# Additions to the knowledge of the land snails of Sabah (Malaysia, Borneo), including 48 new species

**DOI:** 10.3897/zookeys.531.6097

**Published:** 2015-11-02

**Authors:** Jaap J. Vermeulen, Thor-Seng Liew, Menno Schilthuizen

**Affiliations:** 1 JK.art and science, Lauwerbes 8, 2318 AT Leiden, the Netherlands; 2 Institute for Tropical Biology and Conservation, Universiti Malaysia Sabah, Jalan UMS, 88400 Kota Kinabalu, Malaysia; 3 Naturalis Biodiversity Center, Darwinweg 2, 2333 CR Leiden, the Netherlands

**Keywords:** Malaysia, Sabah, Borneo, terrestrial Mollusca, Mount Kinabalu

## Abstract

We present reviews of the Sabah (Malaysia, on the island of Borneo) species of the following problematical genera of land snails (Mollusca, Gastropoda): *Acmella* and *Anaglyphula* (Caenogastropoda: Assimineidae); *Ditropopsis* (Caenogastropoda: Cyclophoridae); *Microcystina* (Pulmonata: Ariophantidae); *Philalanka* and *Thysanota* (Pulmonata: Endodontidae); *Kaliella*, *Rahula*, (Pulmonata: Euconulidae); *Trochomorpha* and *Geotrochus* (Pulmonata: Trochomorphidae). Next to this, we describe new species in previously revised genera, such as *Diplommatina* (Diplommatinidae); *Georissa* (Hydrocenidae); as well as some new species of genera not revised previously, such as *Japonia* (Cyclophoridae); *Durgella* and *Dyakia* (Ariophantidae); *Amphidromus*, and *Trachia* (Camaenidae); *Paralaoma* (Punctidae); *Curvella* (Subulinidae). All descriptions are based on the morphology of the shells. We distinguish the following 48 new species: *Acmella cyrtoglyphe*, *Acmella umbilicata*, *Acmella ovoidea*, *Acmella nana*, *Acmella subcancellata*, *Acmella striata*, and *Anaglyphula sauroderma* (Assimineidae); *Ditropopsis davisoni*, *Ditropopsis trachychilus*, *Ditropopsis constricta*, *Ditropopsis tyloacron*, *Ditropopsis cincta*, and *Japonia anceps* (Cyclophoridae); *Diplommatina bidentata* and *Diplommatina tylocheilos* (Diplommatinidae); *Georissa leucococca* and *Georissa nephrostoma* (Hydrocenidae); *Durgella densestriata*, *Dyakia chlorosoma*, *Microcystina microrhynchus*, *Microcystina callifera*, *Microcystina striatula*, *Microcystina planiuscula*, and *Microcystina physotrochus* (Ariophantidae); *Amphidromus psephos* and *Trachia serpentinitica* (Camaenidae); *Philalanka tambunanensis*, *Philalanka obscura*, *Philalanka anomphala*, *Philalanka rugulosa*, and *Philalanka malimgunung* (Endodontidae); *Kaliella eurytrochus*, *Kaliella sublaxa*, *Kaliella phacomorpha*, *Kaliella punctata*, *Kaliella microsoma*, *Rahula delopleura*, (Euconulidae); *Paralaoma angusta* (Punctidae); *Curvella hadrotes* (Subulinidae); *Trochomorpha trachus*, *Trochomorpha haptoderma*, *Trochomorpha thelecoryphe*, *Geotrochus oedobasis*, *Geotrochus spilokeiria*, *Geotrochus scolops*, *Geotrochus kitteli*, *Geotrochus subscalaris*, and *Geotrochus meristorhachis* (Trochomorphidae).

## Introduction

Apart from a few revised groups (Vermeulen 1991, 1993, 1994, 1996; Liew et al. 2009), the taxonomic structure supporting recent research on the terrestrial molluscs of Sabah, Malaysia (the Northeast part of the Island of Borneo), such as Clements et al. (2008); Liew et al. (2010); Schilthuizen et al. (2013), consists of an unpublished working guide assembled by the first author in 2005. It includes an outdated checklist of all the species occurring in Sabah, with short descriptions where necessary to distinguish between species, and illustrations of the most critical groups. The working guide contains numerous undescribed species, with informal species names. Recently, some of these informal names have found their way into the scientific literature and on to the electronic taxonomic media. This is undesirable. Therefore, we have decided to formally describe the new species present in the working guide. This should be seen as a first step in a process that will culminate into a comprehensive guide to the land snail fauna of Sabah. We present the new species of several critical genera within the framework of a concise review of those genera, in order to provide present researchers with an identification tool with our latest insights into the taxonomy of the local land snail fauna. We divide the species of the larger genera reviewed in informal groups, to facilitate the use of the reviews.

At present, the material available to us largely consists of dry specimens, generally extracted from soil samples gathered by the authors since 1986. The samples from Sabah have yielded close to 8000 lots in our combined collections. Therefore, the species are distinguished by shell characters only. We are aware of the shortcomings of these proceedings, but we feel that investigations into the Sabah snail fauna are better helped by describing the species now, rather than by waiting until we have preserved animals of a representative selection of the species.

In the enumerations of localities, words are used from the Malay language: *batu* (= rock), *bukit* (= hill), *gua* (= cave), *gunung* (= mountain), *pulau* (= island), and *sungai* (= river).

The material studied derives from the following collections, indicated with abbreviations: BOR/MOL (*BORNEENSIS* collection, Institute for Tropical Biology and Conservation, Universiti Malaysia Sabah), RMNH (Naturalis, the natural history museum of the Netherlands), and V (private collection J.J. Vermeulen, Leiden, the Netherlands).

The drawings were made by the first author, with the aid of a Wild M8 stereo microscope with a Camera Lucida device; the SEM images were made by the second author. Also, all BOR/MOL samples were identified by the second author.

## Systematics

### Clade: CAENOGASTROPODA Cox

#### Family ASSIMINEIDAE

Taxon classificationAnimaliaLittorinimorphaAssimineidae

H. & A. Adams

##### Short description.

Shell very small to medium-sized, dextral, globose, ellipsoid, ovoid or conical, rarely almost discoid. Last whorl without a pore near the suture; on the inside usually without a constriction, rarely with a constriction (*Anaglyphula*). Sculpture absent, or fine spiral and/or radial sculpture present, sometimes with more distinct ribs and tubercles. Aperture usually angular above; peristome not or slightly thickened. Umbilicus closed or open, usually narrow. Operculum corneous, sometimes calcareous, usually paucispiral with an excentrical nucleus (Family description adapted from Fukuda and Ponder 2003 and Miranda et al. 2014).

##### Habitat and distribution.

In marine and intertidal environments, in the tropics also terrestrial. Globally distributed in temperate and tropical regions.

##### Remarks.

We provide a review of the terrestrial Assimineidae native to Sabah.

#### Genus Acmella

Taxon classificationAnimaliaLittorinimorphaAssimineidae

Blanford, 1869

Acicula subgenus *Acmella* W.T. Blanford, 1869: 178. *Acmella* (W.T. Blanford) Nevill, 1878: 251.

##### Diagnosis for the Sabah species.

Land snails. Spire without constriction. Umbilicus often with a slight periomphalic thread starting on the columellar side of the peristome, close to the columellar corner, and spiralling (steeply) upwards. Peristome usually not or hardly hardly thickened (except sometimes in gerontic shells).

##### Remarks.

Within several species long and slender shells occur next to shorter and wider ones. The periomphalic thread is sometimes inconspicuous and difficult to see, a mere strip of somewhat rougher sculpture that betrays its presence by the dirt that accumulates around it.

The family typically inhabits intratidal and supratidal environments. Only few genera live in fresh water or on land.

We divide the genus into two informal groups.

#### Group 1. Radial sculpture predominant.

##### Acmella cyrtoglyphe

Taxon classificationAnimaliaLittorinimorphaAssimineidae

Vermeulen, Liew & Schilthuizen sp. n.

http://zoobank.org/8D19118B-5CE0-469A-8AAA-30945C0611FB

Figure 1

Acmella cyrtoglyphe nomen nudum: Clements et al. 2008: 2761–2762.

###### Holotype.

Malaysia, Sabah, Interior Province, Sepulut valley, Gua Sanaron (leg. J.J. Vermeulen & M. Schilthuizen, RMNH.5003948).

###### Examined material from Sabah.

*Interior Province.* Pinangah valley, Batu Urun (= Bukit Sinobang) (leg. J.J. Vermeulen, V 7977; leg. R. Haegens, V 5635). Pun Batu c. 30 km West of Sepulut (leg. J.J. Vermeulen, V 1271). Sepulut valley, Batu Punggul (leg. J.J. Vermeulen, V 1915); Batu Temurung (leg. J.J. Vermeulen, V 8061); Gua Pungiton (leg. J.J. Vermeulen & M. Schilthuizen, V 7527; leg. M. Schilthuizen, BOR/MOL 609); Gua Sanaron (leg. J.J. Vermeulen & M. Schilthuizen, V 7647; leg. M. Schilthuizen, V 13514, BOR/MOL 601); Gua Sanaron, inside cave (leg. M. Schilthuizen, V 13558). *Sandakan Province*. Kinabatangan valley, Batu Keruak (leg. M. Salverda, H. van Oosten, BOR/MOL 1775; leg. T.S. Liew & B. Elahan, BOR/MOL 1867); Batu Mawas (leg. T.S. Liew & M. Schilthuizen, BOR/MOL 2013, BOR/MOL 3592); Batu Tomanggong Kecil (leg. T.S. Liew & B. Elahan, BOR/MOL 2045); Batu Pangi (leg. J.J. Vermeulen & M. Schilthuizen, V 9644). Pinangah valley, Batu Melikop (leg. R. Kiew, V 7718). Segama valley, North end of limestone ridge on East bank Tabin River (leg. J.J. Vermeulen & M. Schilthuizen, V 7754; leg. J.J. Vermeulen & M. Schilthuizen, V 7757). *Tawau Province*. Batu Baturong c. 50 km W.S.W. of Lahad Datu (leg. J.J. Vermeulen & H. Duistermaat, V 1844); Batu Baturong, North slope (leg. J.J. Vermeulen, V 7571); Gua Madai c. 40 km S.S.W. of Lahad Datu (leg. J.J. Vermeulen & H. Duistermaat, V 1735); Gua Madai c. 40 km S.S.W. of Lahad Datu, N.E.-end (leg. J.J. Vermeulen, V 7677). Segama valley, ‘Kirk’s Cave’ 8 km North of Lahad Datu (leg. J.J. Vermeulen, V 1230). Semporna area, Segarong Hills, Batu Tengar, 25 km E.S.E. Of Kunak (leg. J.J. Vermeulen & H. Duistermaat, V 1811); Bukit Pababola, 25 km E.S.E. of Kunak (leg. J.J. Vermeulen & H. Duistermaat, V 1783).

###### Description.

Shell minute, thin, somewhat translucent, white. Surface shiny. Spire conical with approx. flat sides, apex obtuse, whorls convex, sometimes slightly shouldered. *Sculpture.* Radial sculpture predominant: densely placed and regularly spaced, prosocline riblets which are distinctly sinuous around the periphery, and which below the periphery are about as strong as above or weaker, some bifurcating from the periphery downwards. Spiral threads usually present, visible in between the radial riblets, inconspicuous to rather distinct, (rather) densely placed at regular intervals. *Aperture* approx. obliquely elliptic in outline, with a straight parietal side, transition from parietal to basal side rounded to obtusely angular. *Umbilicus* open, (rather) wide. *Dimensions.* Height 1.00–1.50 mm; width 0.85–1.10 mm; h/w 1.10–1.36; number of whorls 4 1/8–5 7/8; height aperture 0.35–0.50 mm; width aperture 0.37–0.50 mm.

**Figure 1–2.**
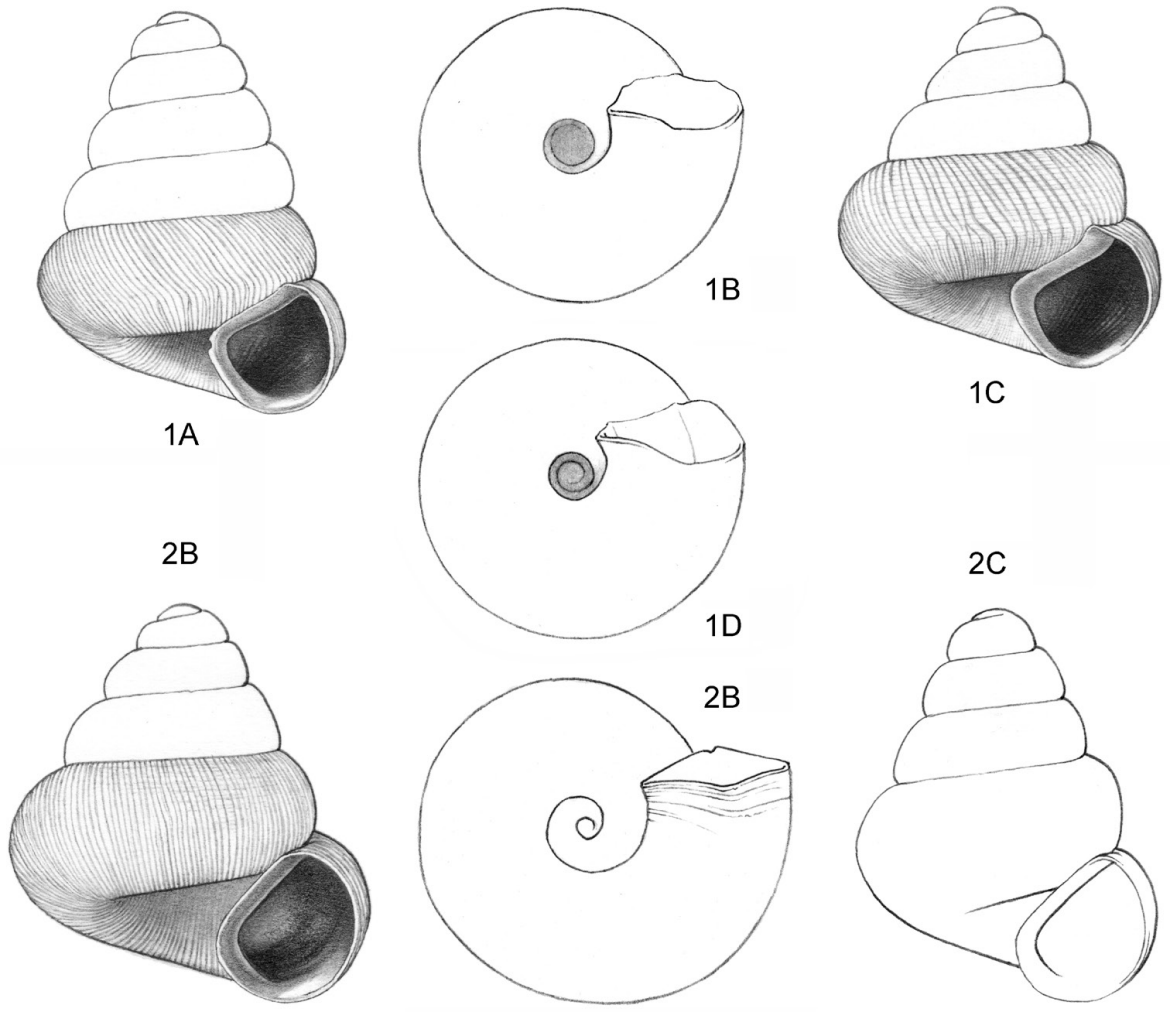
**1** *Acmella cyrtoglyphe* sp. n. Frontal view, shell 1.4 mm high **1B** Same shell, umbilical view **1C** Frontal view, shell 1.1 mm high **1D** Same shell, umbilical view (**1A, 1B** Malaysia, Sabah, Interior Province, Sepulut valley, Gua Sanaron, RMNH.5003948, holotype **1C, 1D** Malaysia, Sabah, Sandakan Province, Kinabatangan valley, Batu Pangi, V 9644) **2** *Acmella umbilicata* sp. n. **2A** Frontal view, shell 1.3 mm high **2B** Same shell, umbilical view **2C** Frontal view, shell 1.3 mm high (Malaysia, Sabah, Interior Province, Pinangah valley, Batu Urun (= Bukit Sinobang) **2A, 2B** RMNH.5003946, holotype; **2C** V 9644).

###### Habitat in Sabah and distribution.

Primary and secondary forest on limestone bedrock, at 0–700 m alt. **Sabah: Interior Province, South part; East coast: from Kinabatangan River valley and further South.** Also in Sarawak, Kalimantan. Endemic to Borneo.

###### Cross diagnosis.

Shares the prominent, prosocline radial sculpture with *Acmella roepstoffiana* Godwin Austen & Nevill, 1879, from the Nicobar Islands. *Acmella cyrtoglyphe* differs by the more convex whorls and the wider umbilicus, which is not at all covered by the peristome.

###### Remarks.

The material of this species displays variability in two characters but we are not able to divide it into discrete units. The ratio height/width varies, and with it the width of the umbilicus. The spacing of the radial ribs above the periphery also varies; specimens with moderately spaced radial ribs above the periphery have the radial ribs bifurcating more frequently below the periphery. Next to this, the whorls are more depressed in some specimens than in others.

###### Etymology.

The name refers to the shape of the riblets [*kurtos* (Gr.) = bent; *glupho* (Gr.) = to engrave].

##### Acmella umbilicata

Taxon classificationAnimaliaLittorinimorphaAssimineidae

Vermeulen, Liew & Schilthuizen sp. n.

http://zoobank.org/2FCADD8A-7097-4D09-AD09-A276DCF03BA8

Figure 2

Acmella umbilicata nomen nudum: Clements et al. 2008: 2761–2762.

###### Holotype.

Malaysia, Sabah, Interior Province, Pinangah valley, Batu Urun (= Bukit Sinobang) (leg. J.J. Vermeulen, RMNH.5003946).

###### Examined material from Sabah.

*Interior Province.* Pinangah valley, Batu Urun (= Bukit Sinobang) (leg. J.J. Vermeulen, V 8016, MOL/BOR 610; leg. M. Schilthuizen, V 13508, MOL/BOR 611). Sepulut valley, Gua Sanaron, inside cave (leg. M. Schilthuizen, V 13559).

###### Description.

Shell minute, thin, somewhat translucent, white. Surface shiny. Spire conical with flat or slightly concave sides, apex obtuse, whorls convex, sometimes slightly shouldered. *Sculpture.* Radial sculpture predominant: densely placed and regularly spaced, almost orthocline riblets which are about straight with a few slightly sinuous around the periphery in between, and which below the periphery are about as strong as above, rarely bifurcating. Spiral threads inconspicuous or almost absent. *Aperture* approx. obliquely elliptic in outline, with a straight parietal side, transition from parietal to basal side rounded to obtusely angular. *Umbilicus* open, very wide. *Dimensions.* Height 1.15–1.50 mm; width 0.95–1.15 mm; h/w 1.00–1.37; number of whorls 4 1/8–5; height aperture 0.45–0.55 mm; width aperture 0.45–0.50 mm.

###### Habitat in Sabah and distribution.

(Disturbed) primary forest on limestone bedrock, at c. 400 m alt. **Sabah: Interior Province, South part only**. Endemic to Sabah.

###### Cross diagnosis.

Differs from *Acmella cyrtoglyphe* by the almost orthocline radial ribs.

###### Remarks.

Just as in *Acmella cyrtoglyphe*, we observe variability in the ratio height/width of the shell, and with it in the width of the umbilicus.

###### Etymology.

The name refers to the wide umbilicus.

#### Group 2. Spiral sculpture predominant, or radial and spiral sculpture about equally strong, or virtually no sculpture present.

##### Acmella polita

Taxon classificationAnimaliaLittorinimorphaAssimineidae

Von Moellendorff, 1887

Figure 3

Acmella polita Von Moellendorff, 1887b: 301; Schilthuizen and Vermeulen 2003: 95; Clements et al. 2008: 2761–2762. Type from Philippines, Luzon, Montalban near Manilla.

###### Examined material from Sabah.

*Interior Province*. Pinangah valley, Batu Urun (= Bukit Sinobang) (leg. J.J. Vermeulen, V 7979). Pun Batu c. 30 km West of Sepulut (leg. J.J. Vermeulen, V 1270). Sepulut valley, Batu Punggul (leg. J.J. Vermeulen, V 1914); Batu Temurung (leg. J.J. Vermeulen, V 8018; leg. M. Schilthuizen, BOR/MOL 531; leg. J.J. Vermeulen, BOR/MOL 524); Bukit Tinahas, East end of Batu Punggul limestone (leg. J.J. Vermeulen & M. Schilthuizen, V 7608); Gua Pungiton (leg. J.J. Vermeulen & M. Schilthuizen, V 7526; leg. M. Schilthuizen, BOR/MOL 523); Gua Sanaron (leg. J.J. Vermeulen & M. Schilthuizen, V 7646; leg. M. Schilthuizen & A.S. Cabanban, BOR/MOL 1745; leg. M. Schilthuizen, BOR/MOL 530). *Sandakan Province*. Kinabatangan valley, Batu Keruak 2 near Sukau (leg. J.J. Vermeulen & M. Schilthuizen, V 9784, BOR/MOL 1744; leg. T.S. Liew & B. Elahan, BOR/MOL 3586, BOR/MOL 1834, BOR/MOL 1871, BOR/MOL 3585; leg. M. Salverda & H. van Oosten, BOR/MOL 1749, BOR/MOL 1750); Batu Mawas (leg. T.S. Liew & M. Schilthuizen, BOR/MOL 1980); Batu Pangi (leg. J.J. Vermeulen & M. Schilthuizen, V 9636); Batu Tai (not Bod Tai) near Gomantong (leg. J.J. Vermeulen & M. Schilthuizen, V 9602; leg. T.S. Liew & B. Elahan, BOR/MOL 1900, BOR/MOL 1935); Batu Tomanggong Kecil (leg. J.J. Vermeulen & M. Schilthuizen, V 9697; leg. M. Salverda & H. van Oosten, BOR/MOL 1748); Batu Tulug (Batu Putih) along road Lahad Datu-Sandakan, North of bridge over Kinabatangan River (leg. J.J. Vermeulen & H. Duistermaat, V 1505); Batu Tomanggong Besar (leg. G.E. Wilford, loc. 66, V 13412; leg. M. Schilthuizen, BOR/MOL 3587, BOR/MOL 1742, BOR/MOL 1743); Gomantong Hill 30 km South of Sandakan (leg. J.J. Vermeulen & H. Duistermaat, V 1622); Tandu Batu (leg. J.J. Vermeulen & M. Schilthuizen, V 9624); Unnamed hill near Sukau Police Station (, BOR/MOL 1747, BOR/MOL 1739; leg. T.S. Liew, BOR/MOL 2140; leg. T.S. Liew & B. Elahan, BOR/MOL 2174).Segama valley, North end of limestone ridge on East bank Tabin River (leg. J.J. Vermeulen & M. Schilthuizen, V 7762, BOR/MOL 522). *Tawau Province*. Gua Madai c. 40 km S.S.W. of Lahad Datu (leg. J.J. Vermeulen & H. Duistermaat, V 1734). Segama valley, ‘Kirk’s Cave’ 8 km North of Lahad Datu (leg. J.J. Vermeulen, V 1242). Semporna area, Segarong Hills, Batu Tengar, 25 km E.S.E. Of Kunak (leg. J.J. Vermeulen & H. Duistermaat, V 1810); Bukit Pababola, 25 km E.S.E. Of Kunak (leg. J.J. Vermeulen & H. Duistermaat, V 1784).

###### Description.

Shell very small, rather thick, hardly translucent, white. Surface dull or somewhat shiny. Spire conical with approx. flat or slightly convex sides, apex obtuse, whorls moderately convex. *Sculpture.* Radial sculpture: scattered, inconspicuous growth lines only, at irregular intervals. Spiral sculpture (almost) absent, or locally a very fine, dense spiral striation, just visible at 40 times magn. *Aperture* obliquely elliptic to broadly ovate in outline, with a straight or slightly curved parietal side, transition from parietal to basal side rounded to obtusely angular. *Umbilicus* open, (rather) narrow. *Dimensions.* Height 1.95–4.50 mm; width 1.40–3.00 mm; h/w 1.18–1.50; number of whorls 4 3/4–6 1/2; height aperture 0.85–2.00 mm; width aperture 0.90–1.70 mm.

**Figure 3–5.**
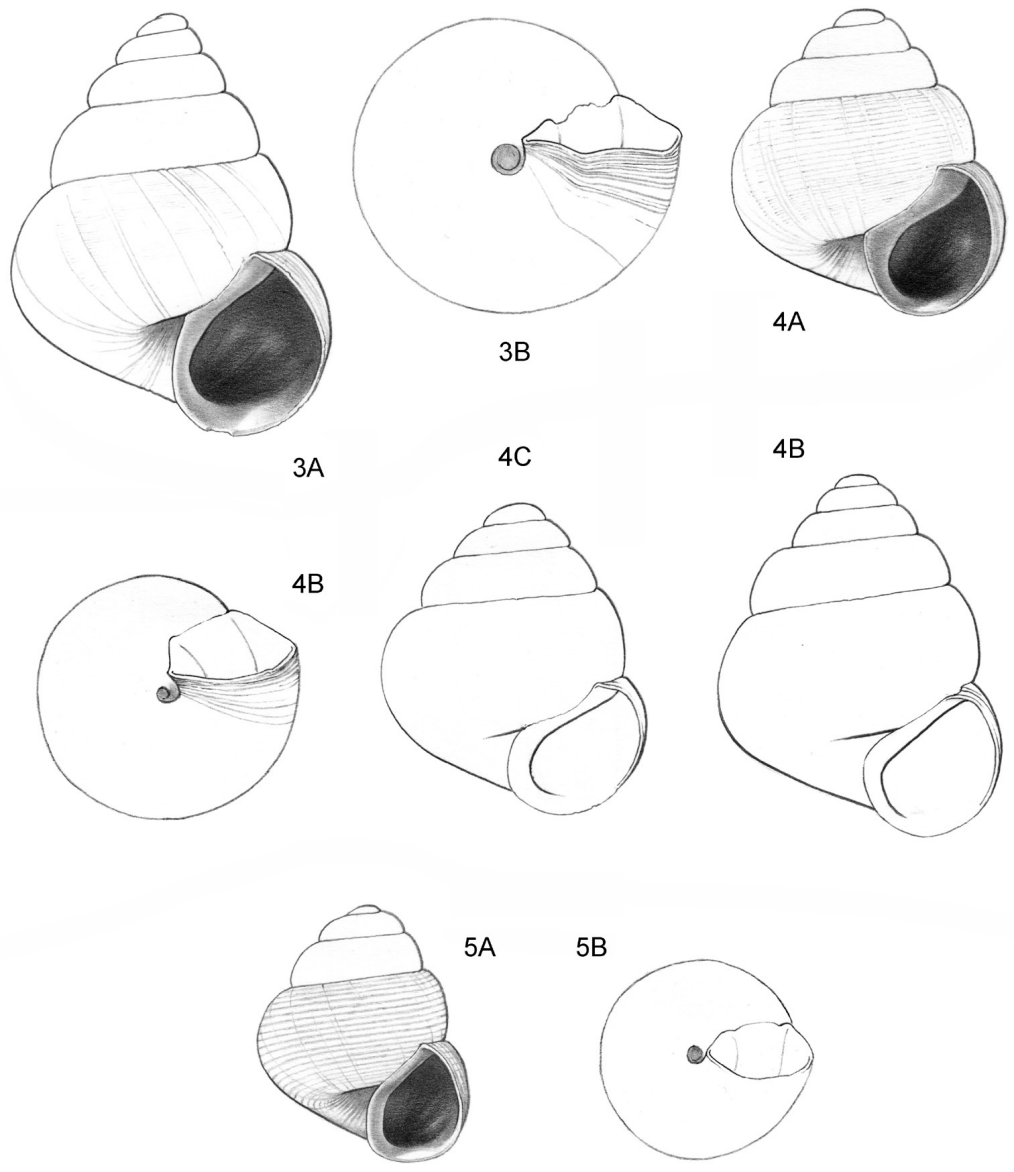
**3** *Acmella polita* Von Moellendorff, 1887. **3A** Frontal view, shell 2.3 mm high **3B** Umbilical view (Malaysia, Sabah, Sandakan Province, Segama valley, North end of limestone ridge on East bank Tabin River, V 7762). **4** *Acmella ovoidea* sp. n. **4A** Frontal view, shell 1.2 mm high **4B** Same shell, umbilical view **4C** Frontal view, shell 1.15 mm high **4D** Frontal view, shell 1.35 mm high (**4A, 4B** Malaysia, Sabah, Interior Province, Pinangah valley, Batu Urun (= Bukit Sinobang), RMNH.5003949, holotype; **4C, 4D** Malaysia, Sabah, Interior Province, Sepulut valley, Batu Temurung, V 8019) **5** *Acmella nana* sp. n. **5A** Frontal view, shell 0.7 mm high **5B** Same shell, umbilical view (Malaysia, Sarawak, 4^th^ Division, Niah Caves, South side of limestone area, West side of quarry, soil-filled crevice opened in quarry, RMNH.5003950, holotype).

###### Habitat in Sabah and distribution.

Primary and secondary forest on limestone bedrock, often in caves. Alt. 0–500 m. **Sabah: Interior; East coast**. Also in Kalimantan Timur. Distribution: Philippines.

###### Cross diagnosis.

The largest *Acmella* species in Sabah.

##### Acmella ovoidea

Taxon classificationAnimaliaLittorinimorphaAssimineidae

Vermeulen, Liew & Schilthuizen sp. n.

http://zoobank.org/0A197662-10ED-4464-A8C2-989A68262930

Figure 4

Acmella ovoidea nomen nudum: Clements et al. 2008: 2761–2762.
Acmella sp. “bor-01 [V 7980]”: Schilthuizen and Vermeulen 2003: 95.
Acmella minutissima auct.: Schilthuizen et al. 2013: online supplementary data.

###### Holotype.

Malaysia, Sabah, Interior Province, Pinangah valley, Batu Urun (= Bukit Sinobang) (leg. J.J. Vermeulen, RMNH.5003949).

###### Examined material from Sabah.

*Interior Province*. Pinangah valley, Batu Urun (= Bukit Sinobang) (leg. J.J. Vermeulen, V 7980, BOR/MOL 526; leg. M. Schilthuizen, BOR/MOL 528). Pun Batu c. 30 km West of Sepulut (leg. J.J. Vermeulen, V 1269). Sepulut valley, Batu Punggul (leg. J.J. Vermeulen, V 1913; leg. M. Schilthuizen, BOR/MOL 600); Batu Temurung (leg. J.J. Vermeulen, V 8019, BOR/MOL 525); Gua Pungiton (leg. J.J. Vermeulen & M. Schilthuizen, V 7528); Gua Sanaron (leg. J.J. Vermeulen & M. Schilthuizen, V 7645; leg. J.J. Vermeulen & M. Schilthuizen, V 7664); Batu Tinagas (leg. M. Schilthuizen, BOR/MOL 527). *Sandakan Province*. Kinabatangan valley, Batu Keruak near Sukau (leg. J.J. Vermeulen & M. Schilthuizen, V 9785; leg. T.S. Liew & B. Elahan, BOR/MOL 1872, BOR/MOL 1835); Batu Batangan (leg. M. Schilthuizen, BOR/MOL 1737); Batu Mawas (leg. M. Schilthuizen, BOR/MOL 1732; leg. T.S. Liew & M. Schilthuizen, BOR/MOL 1946, BOR/MOL 1981, BOR/MOL 1945, BOR/MOL 3588); Batu Pangi (leg. J.J. Vermeulen & M. Schilthuizen, V 9639, BOR/MOL 1730); Batu Tai (not Bod Tai) near Gomantong (leg. J.J. Vermeulen & M. Schilthuizen, V 9577, BOR/MOL 1736; leg. T.S. Liew & B. Elahan, BOR/MOL 3590, BOR/MOL 3589, BOR/MOL 1901); Batu Tai (leg. M. Schilthuizen, BOR/MOL 1733); Batu Tomanggong Besar (leg. M. Schilthuizen, BOR/MOL 607, BOR/MOL 1738; leg. T.S. Liew & B. Elahan, BOR/MOL 2270, BOR/MOL 2243); Batu Tomanggong Kecil (leg. J.J. Vermeulen & M. Schilthuizen, V 9708, BOR/MOL 1734; leg. T.S. Liew & B. Elahan, BOR/MOL 2048, BOR/MOL 2016; leg. M. Salverda & H. van Oosten, BOR/MOL 1740); Batu Tulug (Batu Putih) along road Lahad Datu-Sandakan, North of bridge over Kinabatangan River (leg. J.J. Vermeulen & H. Duistermaat, V 1504); Gomantong Hill 30 km South of Sandakan (leg. J.J. Vermeulen & H. Duistermaat, V 1621; leg. T.S. Liew & J.P. King, BOR/MOL 3625; leg. A. van Til, BOR/MOL 3275, BOR/MOL 3336, BOR/MOL 3272; leg. M. Schilthuizen, BOR/MOL 603); Tandu Batu (leg. J.J. Vermeulen & M. Schilthuizen, V 9622, BOR/MOL 1731); Batu Materis (leg. M. Schilthuizen, BOR/MOL 1735; leg. T.S. Liew & B. Elahan, BOR/MOL 2104, BOR/MOL 2074); Unnamed hill near Sukau Police Station (leg. T.S. Liew & B. Elahan, BOR/MOL 2207, BOR/MOL 2175; leg. T.S. Liew, BOR/MOL 2141, BOR/MOL 3591; leg. M. Schilthuizen, BOR/MOL 1757, BOR/MOL 1741). Pinangah valley, Batu Melikop (leg. R. Kiew, V 7717). Segama valley, North end of limestone ridge on East bank Tabin River (leg. J.J. Vermeulen & M. Schilthuizen, V 7761). *Tawau Province*. Batu Baturong c. 50 km W.S.W. of Lahad Datu (leg. J.J. Vermeulen & H. Duistermaat, V 1846); North slope (leg. J.J. Vermeulen, V 7573). Gua Madai c. 40 km S.S.W. of Lahad Datu (leg. J.J. Vermeulen & H. Duistermaat, V 1733). Semporna area, Segarong Hills, Batu Tengar, 25 km E.S.E. Of Kunak (leg. J.J. Vermeulen & H. Duistermaat, V 1809); Bukit Pababola, 25 km E.S.E. Of Kunak (leg. J.J. Vermeulen & H. Duistermaat, V 1785).

###### Description.

Shell minute, rather thin, translucent, white. Surface shiny. Spire conical with slightly convex sides to ovoid, apex (broadly) obtuse, whorls slightly to moderately convex, sometimes slightly shouldered. *Sculpture.* Spiral sculpture predominant. Radial sculpture: scattered, inconspicuous growth lines only, in some shells locally grading into slight, densely placed and regularly spaced riblets on the crests of the spiral threads. Spiral sculpture: densely placed and regularly spaced, very fine, low and wide threads with only very narrow grooves in between. *Aperture* obliquely ovate in outline, with a concave to slightly convex parietal side, transition from parietal to basal side rounded. *Umbilicus* open, narrow. *Dimensions.* Height 0.85–1.35 mm; width 0.80–1.00 mm; h/w 1.00–1.35; number of whorls 3 1/2–5; height aperture 0.40–0.55 mm; width aperture 0.40–0.50 mm.

###### Habitat in Sabah and distribution.

Primary and secondary forest on limestone bedrock, at 0–700 m alt. **Sabah: Interior Province, South part; along the East coast from the Kinabatangan River valley Southwards.** Also in Sarawak; Kalimantan Timur. Endemic to Borneo.

###### Cross diagnosis.

Characterized among the Sabah species by the more or less ovoid outline of the shell.

Differs from *Acmella minutissima* (Maassen, 2000) (= *Anaglyphula minutissima* Maassen), from Sumatra, by the same character. In addition, the sculpture on the teleoconch is different: the spiral threads are much wider, leaving only very narrow grooves in between. The radial sculpture cuts into the crests of the spiral grooves.

###### Remarks.

The index h/w is highly variable, and the umbilicus may be wider than in the illustrated shell.

###### Etymology.

The name refers to the shell outline [*ovoideus* (L.) = egg-shaped].

##### Acmella nana

Taxon classificationAnimaliaLittorinimorphaAssimineidae

Vermeulen, Liew & Schilthuizen sp. n.

http://zoobank.org/A2287C13-AE75-4E08-B3D9-BC093116E64A

Figure 5

Acmella nana nomen nudum: Clements et al. 2008: 2761–2762.

###### Holotype.

Malaysia, Sarawak, 4^th^ Division, Niah Caves, South side of limestone area, West side of quarry, soil-filled crevice opened in quarry (leg J.J. Vermeulen, RMNH.5003950).

###### Examined material from Sabah.

*Interior Province*. Pinangah valley, Batu Urun (= Bukit Sinobang) (leg. J.J. Vermeulen, V 7978). Sepulut valley, Gua Pungiton (leg. J.J. Vermeulen & M. Schilthuizen, V 8075, BOR/MOL 529); Gua Sanaron (leg. J.J. Vermeulen & M. Schilthuizen, V 8063). *Sandakan Province*. Kinabatangan valley, Gomantong Hill 30 km South of Sandakan (leg. J.J. Vermeulen & H. Duistermaat, V 13268).

###### Description.

Shell minute (one of the smallest Borneo snail species), thin, translucent, white. Surface shiny. Spire conical with almost flat to distinctly convex sides, apex broadly obtuse, whorls moderately convex, sometimes slightly shouldered. *Sculpture.* Spiral sculpture predominant. Radial sculpture: scattered, inconspicuous growth lines only, grading into somewhat coarser riblets at irregular intervals. Spiral sculpture: rather conspicuous, densely placed and regularly spaced, fine, low and wide threads with only very narrow grooves in between. *Aperture* obliquely ovate in outline, with a slightly concave to slightly convex parietal side, transition from parietal to basal side rounded to obtusely angular. *Umbilicus* open, narrow. *Dimensions.* Height 0.60–0.79 mm; width 0.50–0.60 mm; h/w 1.00–1.35; number of whorls 2 7/8–3 7/8; height aperture 0.30–0.37 mm; width aperture 0.26–0.30 mm.

###### Habitat in Sabah and distribution.

(Disturbed) primary forest on limestone bedrock. Alt. 0–500 m. **Sabah: Interior Province, South part; East coast: Kinabatangan River valley, 1 locality.** Also in Sarawak and East Kalimantan. Endemic to Borneo.

###### Cross diagnosis.

Most similar to *Acmella caelata* Vermeulen & Junau, 2007, from Sarawak. This species is consistently wider (c. 0.7 mm in *Acmella caelata*, 0.50–0.60 mm in *Acmella nana*). If specimens with the same number of whorls are compared, the whorls of *Acmella caelata* are less depressed (at 3 1/8–3 3/8 whorl *Acmella caelata* is 0.80–0.95 mm high, *Acmella nana* only 0.60–0.75 mm high).

Also similar to *Acmella ovoidea* but smaller, with a narrower spire than juvenile *Acmella ovoidea* of the same size. It also has coarser spiral sculpture.

###### Etymology.

The name refers to the size [*nanus* (L.) = dwarf].

##### Acmella subcancellata

Taxon classificationAnimaliaLittorinimorphaAssimineidae

Vermeulen, Liew & Schilthuizen sp. n.

http://zoobank.org/DBE780D0-BB7D-46BD-8481-26EE88831083

Figure 6

###### Holotype.

Malaysia, Sabah, Tawau Province, Batu Baturong c. 50 km W.S.W. of Lahad Datu (leg. J.J. Vermeulen & H. Duistermaat, RMNH.5003945).

###### Examined material from Sabah.

Malaysia, Sabah, *Tawau Province*. Batu Baturong c. 50 km W.S.W. of Lahad Datu, North slope (leg. J.J. Vermeulen, V 7572).

###### Description.

Shell minute, rather thin, somewhat translucent, white. Surface shiny. Spire conical with flat to slightly convex sides, apex somewhat narrowly obtuse, whorls (moderately) convex, sometimes slightly shouldered. *Sculpture.* Spiral sculpture predominant. Radial sculpture: scattered, inconspicuous growth lines, grading into inconspicuous, rather densely placed and regularly spaced riblets on the crests of the spiral threads. Spiral sculpture: densely placed and regularly spaced, very fine, low and wide threads with only a very narrow groove in between. *Aperture* obliquely ovate in outline, with a slightly concave to approx. straight parietal side, transition from parietal to basal side rounded. *Umbilicus* open, narrow. *Dimensions.* Height 1.19–1.30 mm; width 0.95–1.10 mm; h/w 1.11–1.26; number of whorls 4 1/8–4 1/2; height aperture 0.58–0.69 mm; width aperture 0.49–0.59 mm.

**Figure 6–8.**
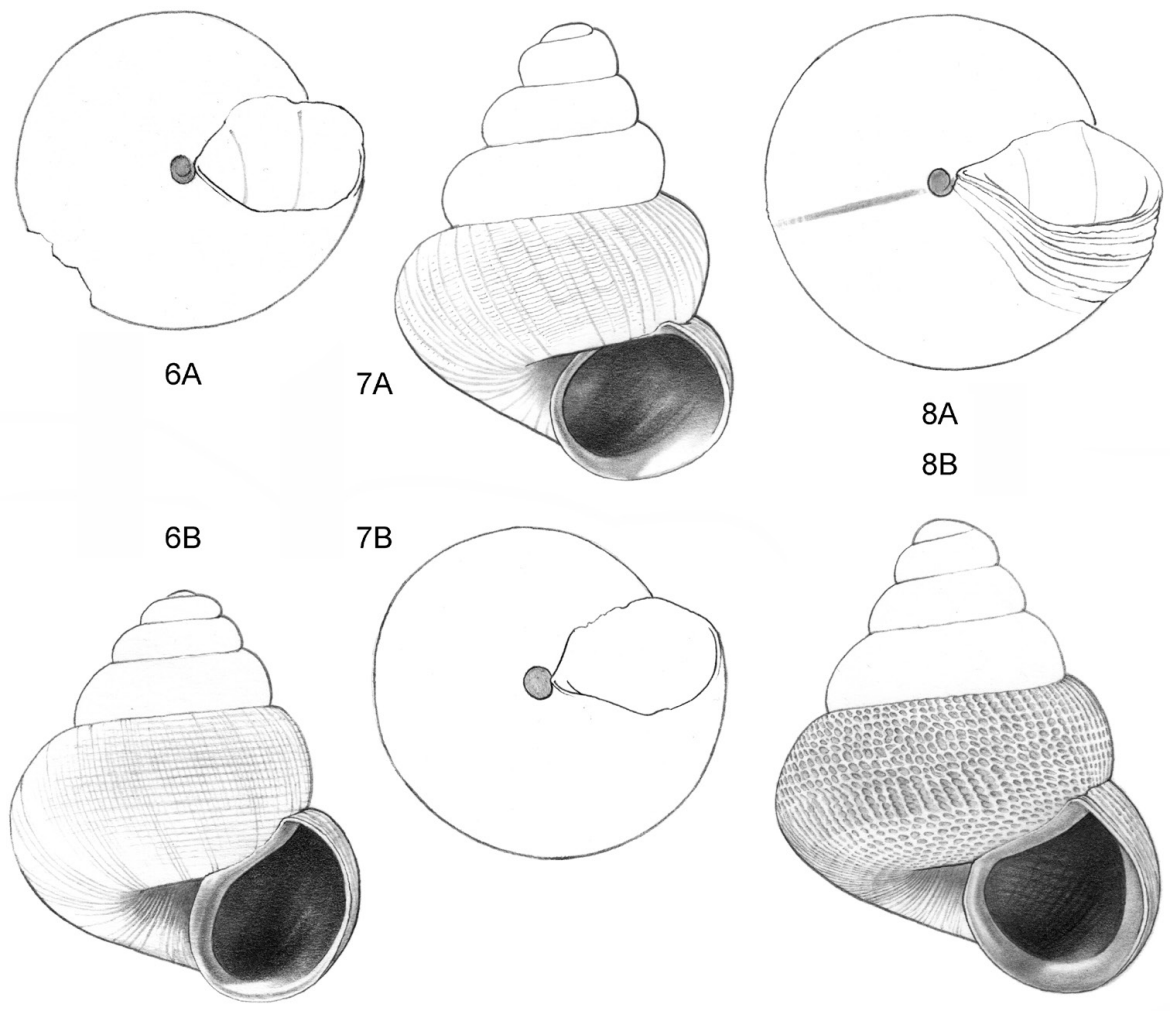
**6** *Acmella subcancellata* sp. n. **6A** Frontal view, shell 1.4 mm high **6B** Same shell, umbilical view (Malaysia, Sabah, Sabah, Tawau Province, Batu Baturong c. 50 km W.S.W. of Lahad Datu, RMNH.5003945, holotype) **7** *Acmella striata* sp. n. **7A** Frontal view, shell 1.6 mm high **7B** Same shell, umbilical view (Malaysia, Sabah, Kudat Province, Balambangan Island, South end, Batu Sireh, RMNH.5003947, holotype) **8** *Anaglyphula sauroderma* sp. n. **8A** Frontal view, shell 1.4 mm high **8B** Same shell, umbilical view (Malaysia, Sabah, Sabah, Tawau Province, Batu Baturong c. 50 km W.S.W. of Lahad Datu, RMNH.50030952, holotype).

###### Habitat in Sabah and distribution.

(Disturbed) primary forest on limestone bedrock, at c. 100 m alt. **Sabah: East coast, Baturong Hill only**. Endemic to Sabah.

###### Cross diagnosis.

Most similar in size and shell sculpture to *Acmella ovoidea*; differs by its conical shell. The sculpture is somewhat more prominent than in *Acmella ovoidea*.

The general shell shape is virtually identical to *Acmella minutissima* (Maassen, 2000), from Sumatra. *Acmella subcancellata* differs by its teleochonch sculpture, consisting of much wider spiral threads, leaving only narrow grooves in between, as well as by the radial sculpture cutting into the crest of the spural threads.

###### Etymology.

The name refers to the shell surface [*sub-cancellata* (L.) = with an inconspicuous, trellis-like structure].

##### Acmella striata

Taxon classificationAnimaliaLittorinimorphaAssimineidae

Vermeulen, Liew & Schilthuizen sp. n.

http://zoobank.org/CBBA3618-1650-44BE-8080-2A5BA127BA5A

Figure 7

Acmella striata nomen nudum: Clements et al. 2008: 2761–2762; Schilthuizen et al. 2013: online supplementary data.

###### Holotype.

Malaysia, Sabah, Kudat Province, Balambangan Island, South end, Batu Sireh (leg. J.J. Vermeulen & M. Schilthuizen, RMNH.5003947).

###### Examined material from Sabah.

*Interior Province*. Pinangah valley, Batu Urun (= Bukit Sinobang) (leg. J.J. Vermeulen, V 8017, BOR/MOL 608; leg. R. Haegens, V 5630). Pun Batu c. 30 km West of Sepulut (leg. J.J. Vermeulen, V 1272). Sepulut valley, Batu Temurung (leg. J.J. Vermeulen, V 12682); Gua Sanaron (leg. J.J. Vermeulen & M. Schilthuizen, V 8062). *Kudat Province*. Balambangan Island, Kok Simpul (leg. J.J. Vermeulen & M. Schilthuizen, V 9518); South end, Batu Sireh (leg. J.J. Vermeulen & M. Schilthuizen, V 9563). *Sandakan Province*. Kinabatangan valley, Batu Tomanggong Kecil (leg. J.J. Vermeulen & M. Schilthuizen, V 9695); Tandu Batu (leg. J.J. Vermeulen & M. Schilthuizen, V 9621). *Tawau Province*. Batu Baturong c. 50 km W.S.W. of Lahad Datu (leg. J.J. Vermeulen & H. Duistermaat, V 1845). Tawau Hills N.P., waterfalls near Headquarters area (leg. J.J. Vermeulen, V 13199). *West Coast Province*. Mantanani Group, Pulau Mantanani Besar (leg. M. Schilthuizen, V 9846; leg. T.H. Liew, BOR/MOL 3726).

Description: Shell minute, rather thin, slightly translucent, corneous to white. Surface shiny. Spire conical with approx. flat sides, apex somewhat narrowly obtuse, whorls convex, often somewhat shouldered. *Sculpture.* Spiral sculpture predominant. Radial sculpture: growth lines, grading into irregularly and widely spaced riblets locally. Spiral sculpture: densely placed to moderately spaced, thin, narrow, very fine, distinctly wavy spiral threads which are occasionally interrupted or bifurcated, which have shallow and wide furrows in between and which run either approx. parallel to the suture or run slightly obliquely downwards. *Aperture* approx. elliptic in outline, with a slightly convex parietal side, transition from parietal to basal side rounded. *Umbilicus* open, narrow. *Dimensions.* Height 1.40–1.80 mm; width 1.05–1.25 mm; h/w 1.33–1.44; number of whorls 4 1/8–5 1/4; height aperture 0.50–0.70 mm; width aperture 0.55–0.65 mm.

###### Habitat in Sabah and distribution.

Primary forest, primary coastal woodland and secondary vegetation on limestone bedrock, also found in soil over sandstone and volcanic bedrock, at 0–1400 m alt. **Sabah: scattered localities, including islands on the North and West coast**. Also in Sarawak and Kalimantan. Distribution: Ambon, Ceram, Tanimbar Islands (see note below).

###### Cross diagnosis.

Characterized among Sabah *Acmella* by the comparatively high spire with distinctly convex whorls, and the teleoconch with fine, inconspicuous to rather distinct, but always very wavy spiral sculpture.

Also similar to *Acmella sutteri* Van Benthem Jutting, 1958, from Sumba. *Acmella striata* differs by the parietal side of the aperture, which is attached to the previous whorl over slightly more than half the distance between the periphery and the umbilicus of the latter. In *Acmella sutteri* it is only attached to the previous whorl over a very short distance near the angular corner.

###### Remarks.

Most Sabah samples have a slightly oblique spiral striation. In Sarawak shells with a spiral striation parallel to the suture predominate in most localities.

Samples from Mantanani and Balambangan Islands contain relatively large shells of dark colour. Similar shells are found locally in the Kinabatangan River valley; but towards the West populations grade into a smaller form with white shells, as is found in Gunung Mulu N.P., Sarawak. A sample from Kalimantan contains medium-sized and white shells.

The samples V 4721 (Indonesia, Tanimbar Islands, Yamdena, near Kampong Makatian), V 5052 (Indonesia, Ambon, 1 km from Poka), and V 5248 (Indonesia, Ceram, Moso) contain shells identical to the Borneo material. We regard these as the same species, and conclude that *Acmella striata* is a rather widespread species. It may well occur in Philippines, but we cannot find any described species identical to it.

###### Etymology.

The name refers to the shell surface [*striata* (L.) = striate].

#### Genus Anaglyphula

Taxon classificationAnimaliaLittorinimorphaAssimineidae

Rensch, 1932

Anaglyphula Rensch, 1932: 122.

##### Diagnosis for the Sabah species.

Land snails. Spire with a slight constriction about 1/2 whorl back of the aperture, consisting of a circular rim on the inner surface, on the outside this rim shines through as a thin, white line. Umbilicus without periomphalic thread. Peristome thickened.

##### Cross diagnosis.

The presence of a constriction distinguishes *Anaglyphula* from *Acmella*. If this is not visible because the shell has turned opaque, the thickened peristome may serve as a distinguishing character.

##### Remarks.

Originally included in the Valloniidae, a Pulmonate family. Its position in the Caenogastropoda was established by Vermeulen (1996) and Vermeulen and Whitten (1998), who found a thin, paucispiral operculum in *Anaglyphula whitteni* Vermeulen, 1996, as well as in *Anaglyphula tiluana* (Von Moellendorff, 1897) (= *Acanthinula tiluana* Von Moellendorff).

#### Anaglyphula sauroderma

Taxon classificationAnimaliaLittorinimorphaAssimineidae

Vermeulen, Liew & Schilthuizen sp. n.

http://zoobank.org/4E829B05-3170-460C-91E1-04C1BA47A017

Figure 8

##### Holotype.

Malaysia, Sabah, Tawau Province, Batu Baturong c. 50 km W.S.W. of Lahad Datu (leg. J.J. Vermeulen & H. Duistermaat, RMNH.5003952).

##### Description.

Shell minute, rather thick, about opaque, pale corneous. Surface shiny. Spire conical with approx. flat or slightly concave sides, apex somewhat narrowly obtuse, whorls moderately convex. *Sculpture* consisting of radial ribs intersecting with about equally strong spiral threads, creating a pattern of rounded, sharply delineated depressions on the shell surface; locally this pattern is distorted, and around the periphery and below the suture the depressions more or less line up parallel to the growth lines; close to the aperture radial riblets and growth lines predominate. *Aperture* widely and obliquely ovate in outline, with a slightly concave parietal side, transition from parietal to basal side obtusely angular. Peristome thickened, with a slight lip on the inner side. *Umbilicus* open, narrow. *Dimensions.* Height c. 1.75 mm; width c. 1.45 mm; h/w c. 1.25; number of whorls c. 5; height aperture c. 0.80 mm; width aperture c. 0.70 mm.

##### Habitat in Sabah and distribution.

Primary forest on limestone soil, at 100–300 m alt. **Sabah: East coast, Baturong Hill.** Also in Kalimantan Timur. Endemic to Borneo.

##### Cross diagnosis.

The sculpture uniquely identifies the species among the Sabah snail fauna.

*Anaglyphula sauroderma* shares the irregularly cancellate sculpture, with shallow pits, with *Anaglyphula cancellata* Rensch, 1932, from Flores. It differs in having a higher spire (distinctly higher than wide) and evenly rounded whorls.

##### Etymology.

The name refers to the shell surface [*sauros* (Gr.) = lizard; *derma* (Gr.) = skin].

#### Family CYCLOPHORIDAE

Taxon classificationAnimaliaArchitaenioglossaCyclophoridae

Gray

##### Short description.

Shell small to very large, dextral, discoid to ovoid to conical. Last whorl with or without a pore near the aperture; on the inside with (Alycaeinae) or without a constriction. Sculpture absent, or inconspicuous to distinct spiral and/or radial sculpture present; periostracal sculpture often present. Aperture rounded to somewhat angular above; peristome often (distinctly) thickened. Umbilicus usually open, narrow to wide. Operculum corneous or calcareous, multispiral, nucleus approx. central (Family description adapted from Abbott 1989; Herbert and Kilburn 2004).

##### Habitat and distribution.

Terrestrial; ground- or tree-dwellers. Asia, Africa, Australia, and Oceania; fewer species in temperate regions.

#### Genus Ditropopsis

Taxon classificationAnimaliaArchitaenioglossaCyclophoridae

E.A. Smith, 1897

Ditropopsis E.A. Smith, 1897: 416; Fukuda 2000: 1.
Cyclophorus subgenus *Ditropis* Blanford, 1869: 126. Genus *Ditropis* Hanley & Theobald, 1875: xiv.
Ditropopsis [Not *Ditropis* Kirschbaum (Delphacidae, Hemiptera)]

##### Diagnosis for the Sabah species.

Shell very small, white to pale corneous or pale greenish, without any colour patterns. Surface shiny or glossy. Spire rather high-conical, to approx. flat with a protruding apex. Radial sculpture absent or inconspicuous. Spiral sculpture prominent, consisting of distinct cords and keels. Peristome simple or double, expanded or not, without a notch in the angular corner. Height up to 2.3 mm. Umbilicus wide.

##### Cross diagnosis.

Borneo species of *Japonia* Gould, 1859 (s.l., including *Pilosphaera* Lee et al., 2008), have larger shells: usually more than 4 mm high or more. *Japonia* also has a notch in the angular corner of the peristome, and many have shells with brown colour markings. Only *Japonia hyalina* Vermeulen & Junau, 2007, and *Japonia ditropis* Vermeulen & Junau, 2007, from Sarawak, have whitish shells 2.0–3.5 mm high. These two differ from *Ditropopsis* by having a more distinct radial sculpture.

The two Borneo species of *Craspedotropis* Blanford, 1864, see Vermeulen 1999, differ by having a narrower umbilicus and more prominent radial sculpture.

We provide a review of the Sabah species of the genus.

##### Remarks.

The cyclophorid fauna of Borneo includes a small number of species with small shells with conspicuous spiral cords: *Jerdonia* (?) *borneensis* Godwin Austen, 1889, *Cyathopoma everetti* E.A. Smith, 1895, *Craspedotropis andrei* Vermeulen, 1999 and *Craspedotropis juvenilis* Vermeulen, 1999. Except for the second species, all were included in *Craspedotropis* Blanford, 1864 by Vermeulen (1999) with due expression of doubt. His concern was the general similarity of *Craspedotropis* with two largely continental Asiatic genera *Cyathopoma* Blanford, 1864, and *Jerdonia* Blanford & Blanford, 1861. Diagnostic differences between the three are mainly sought in the operculum morphology, a part unfortunately missing in the material of his two new species. The matter is still unresolved, and further complicated when, as we do here, we include Bornean species that show similarity with a fourth genus *Ditropopsis* E.A. Smith, 1897 = *Ditropis* Blanford, 1869. Operculum morphology, again, is not decisive, although the operculum of *Ditropis koperbergi* (Zilch, 1955), see below, differs from that of *Craspedotropis borneensis* (Godwin Austen, 1889) in lacking the erect and outwards folded whorl edges. However, *Ditropis aenigmatica* (Van Benthem Jutting, 1963) (Biak Island, Indonesia) and *Ditropis heterospirifera* (Van Benthem Jutting, 1958) (from Misool Island, Indonesia) are similar to our Bornean species in general terms, but have an operculum with very distinct, erect, calcareous whorl edges.

For the Borneo material we conclude that the species listed below generally differ from the ones included in *Craspedotropis* by Vermeulen, 1999 by having a wider umbilicus and a less distinct radial sculpture. Their shell morphology agrees well with species generally included in *Ditropopsis*, and we include them in that genus. Below, we list all the Sabah species present in the collections available to us.

We divide the genus into three informal groups.

#### Group 1. Teleoconch whorl entirely detached

##### Ditropopsis davisoni

Taxon classificationAnimaliaArchitaenioglossaCyclophoridae

Vermeulen, Liew & Schilthuizen sp. n.

http://zoobank.org/5E4902B2-AD74-403D-8604-2E872638CE04

Figure 9

###### Holotype.

Malaysia, Sabah, Upper Padas valley, Matang River South of Long Pasia (leg. J.J. Vermeulen, RMNH.5003917).

###### Description.

Shell very small, rather thick, somewhat translucent, whitish. Surface shiny. Teleoconch whorl entirely detached, more or less in one plane; apex protruding from the plane, distinctly oblique. *Sculpture.* Radial sculpture: fine growth lines, locally grading into fine, densely placed riblets. Spiral sculpture on the last whorl: 8 cords (position as on the first part of the teleoconch, close to the protoconch): 1 supraperipheral, 1 peripheral, 1 basal, these three distinct, and 5 umbilical, the outer 2 distinct, the inner 3 more inconspicuous; next to these a fine spiral striation on the lower surface. Sculpture continuing up to the peristome. *Aperture.* Peristome simple, not expanded, angular where the spiral cords meet the peristome. *Dimensions.* Height c. 0.7 mm; width c. 1.5 mm (both measured along the axis of the teleoconch); height and width aperture c. 0.5 mm.

**Figure 9–11.**
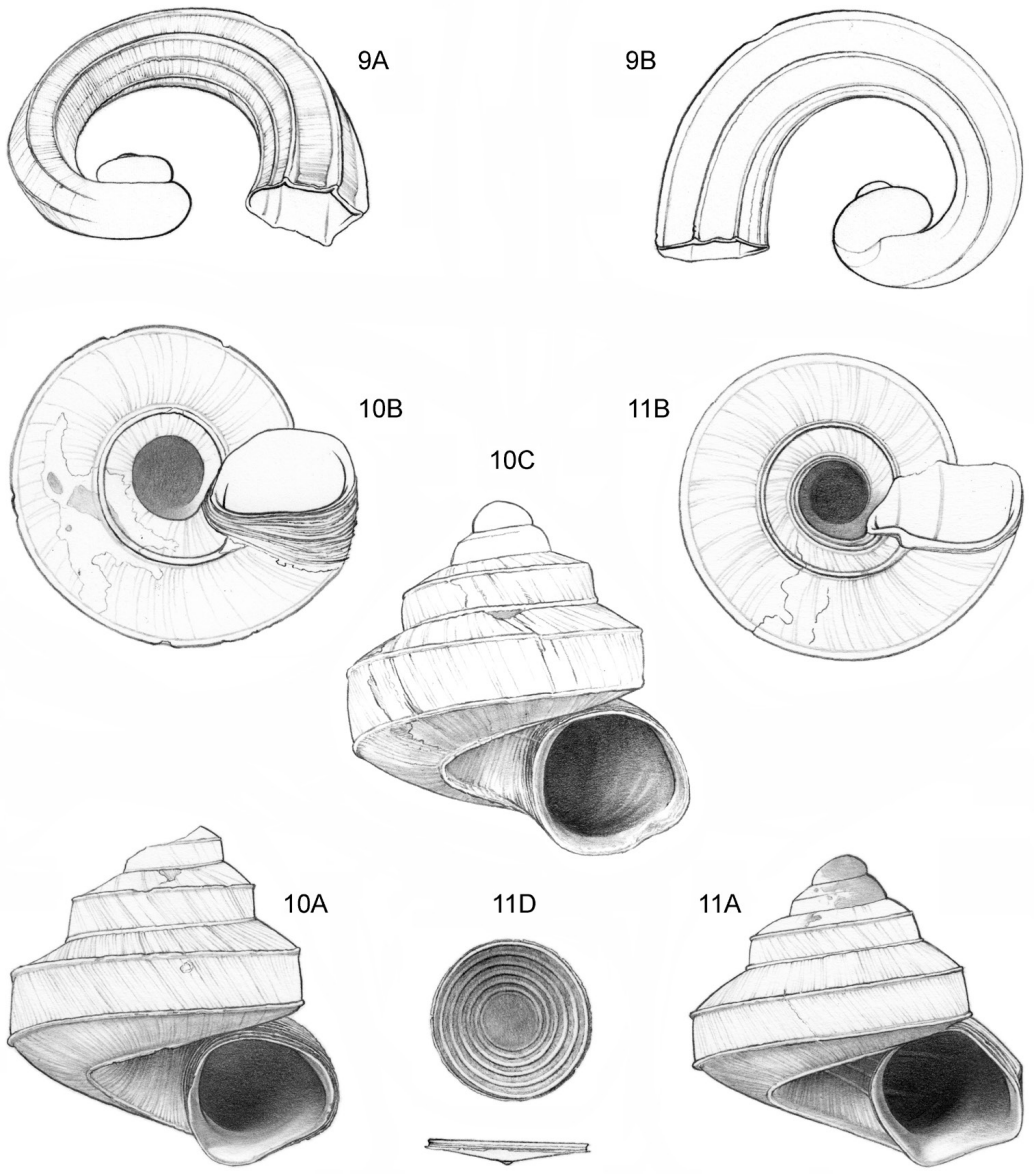
**9** *Ditropopsis davisoni* sp. n. **9A** Frontal view, shell 1.5 mm wide **9B** Back view (Malaysia, Sabah, Upper Padas valley, Matang River South of Long Pasia, RMNH.5003917, holotype) **10** *Ditropopsis trachychilus* sp. n. **10A** Frontal view, shell 2.3 mm high **10B** Umbilical view **10C** Frontal view, shell 2.7 mm high (**10A, 10B** Malaysia, Sabah, Crocker Range N.P., near the km 54 marker on the road Kota Kinabalu-Tambunan, Gunung Mas, RMNH.5003918, holotype, shell partly reconstructed; **10C** Malaysia, Sabah, Kinabalu N.P., Kotal’s route between 2924–3048 m) **11** *Ditropopsis koperbergi* (Zilch, 1955). **11A** Frontal view, shell 1.9 mm high **11B** Umbilical view **11C** Operculum, frontal view, diameter 0.65 mm **11D** Operculum, lateral view (Malaysia, Sabah, Danum Valley Conservation Area, V 9881).

###### Habitat in Sabah and distribution.

Found in montane forest, in a sandstone rock crevice near a small stream in thick leaf litter, at 1100 m alt. **Sabah: Upper Padas River.** Endemic to Sabah.

###### Cross diagnosis.

Uniquely identified among the Sabah species by the tubular, detached teleochonch.

Species from elsewhere with a detached teleoconch are *Ditropopsis adesmospira* Moellendorff, 1895, *Ditropopsis mira* Moellendorff, 1891, (both from the Philippines) *Ditropopsis spiralis* Boettger, 1891 (from Maluku) and *Ditropopsis biroi* Soós, 1911 (from Papua New Guinea). All have a protoconch which protrudes from the teleoconch, and a conical spire.

###### Etymology.

Named in honour of Dr. Geoffrey Davison, tireless advocate of nature conservation in Southeast Asia.

#### Group 2. Teleoconch whorls (almost) entirely attached. Supraperipheral usually cord present.

##### Ditropopsis trachychilus

Taxon classificationAnimaliaArchitaenioglossaCyclophoridae

Vermeulen, Liew & Schilthuizen sp. n.

http://zoobank.org/DBF5C5C5-B845-4479-B2FE-44D0940DCD7D

Figure 10

###### Holotype.

Malaysia, Sabah, West Coast Province, Crocker Range N.P., near the km 54 marker on the road Kota Kinabalu-Tambunan, Gunung Mas (leg. J.J. Vermeulen & M. Schilthuizen, RMNH.5003918).

###### Examined material from Sabah.

*West Coast Province*, Kinabalu N.P., summit trail, between 2308–2775 m (leg. T.S. Liew, BOR/MOL 4121, BOR/MOL 4113); Bowen route at 3376 m (leg. T.S. Liew & Jasilin, BOR/MOL 4111); Mesilau trail between 2092–2356 m (leg. T.S. Liew, BOR/MOL 4119, BOR/MOL 4114); Kotal’s route between 2924–3048 m (leg. T.S. Liew, J. Lapidin, Safrie & Jasilin, BOR/MOL 4117, BOR/MOL 4115; leg. T.S. Liew & Jasilin, BOR/MOL 4118); Sayap-Nunuhon trail between 2640–2944 m (leg. T.S. Liew, Dominik, J. Lapidin & Jasilin, BOR/MOL 4116, BOR/MOL 4112, BOR/MOL 4120, BOR/MOL 4123, BOR/MOL 4124).

###### Description.

Shell very small, rather thick, hardly translucent, pale greenish. Surface shiny. Spire rather high-conical. Radial sculpture: fine growth lines, locally grading into fine, densely placed riblets. *Sculpture.* Spiral sculpture on the last whorl: 4 cords: 1 supraperipheral and 1 peripheral, about equally wide, 1 basal; these all rather distinct; 1 umbilical, inconspicuous and located deep inside the umbilicus; next to these a fine spiral striation on the lower surface. This sculpture continuing up to about 0.8 mm from the peristome, to be replaced by densely and irregularly placed radial riblets with a periostracal crest. *Aperture.* Peristome simple, not expanded, parietal side slightly detached from the penultimate whorl, basal side rounded, hardly drawn out. *Dimensions.* Height c. 2.3 mm; width c. 2 mm; h/w c. 1.15; umbilicus measured over the basal spiral cord c. 25 % of the shell width; number of whorls c. 4 1/8; height aperture c. 0.8 mm; width aperture c. 0.8 mm.

###### Habitat in Sabah and distribution.

Primary montane oak forest on sandstone bedrock, in leaf litter, sub-alpine forest on ultrabasic and granodioritic bedrock, 1300–1400 m (Crocker Range), 2000–3400 m (Mount Kinabalu) alt. **Sabah: Mount Kinabalu, Crocker Range.** Endemic to Sabah.

###### Cross diagnosis.

Similar to *Ditropopsis koperbergi*, but shell higher conical. The distinctive sculpture near the aperture develops only when fully adult, as in other species of the genus.

*Ditropopsis gradata* Quadras & Moellendorff, 1896 and *Ditropopsis pusilla* Quadras & Moellendorff, 1895, from the Philippines, have a similarly high spire and spiral cord distribution. Both species have a more rounded profile to the whorls.

###### Etymology.

The name refers to the peristome [*trachus* (Gr.) rough; *cheilos* (Gr.) = lip].

##### Ditropopsis koperbergi

Taxon classificationAnimaliaArchitaenioglossaCyclophoridae

(Zilch, 1955)

Figure 11

Ditropopsis koperbergi (Zilch) Schilthuizen, 2004: 94. *Ditropis koperbergi* Zilch, 1955: 193. Type from Indonesia, Kalimantan, Landak.

###### Examined material from Sabah.

*West Coast Province*, Kinabalu N.P., Monggis-Tambuyukon trail at 1144 m (leg. T.S. Liew, BOR/MOL 4122). *Interior Province*. Gunung Trusmadi slopes, Gua Loloposon (leg. J.J. Vermeulen, V 13226). *Sandakan Province*. Segama valley, North end of limestone ridge on East bank Tabin River (leg. J.J. Vermeulen & M. Schilthuizen, V 14490). *Tawau Province*. Danum Valley Conservation Area (leg. UMS students, V 9881; leg. H.A. Rutjes, BOR/MOL 312).

###### Description.

Shell very small, rather thick, somewhat translucent, white to pale corneous. Surface shiny. Spire conical, apex not protruding, not oblique. *Sculpture.* Radial sculpture: fine growth lines, locally grading into fine, densely placed riblets. Spiral sculpture on the last whorl: 6 rather distinct cords: 1 supraperipheral and 1 peripheral, about equally wide, 1 basal and 3 umbilical; next to these a fine spiral striation on the lower sureface. Sculpture continuing up to the peristome. *Aperture.* Peristome simple, not expanded, parietal side attached to the penultimate whorl, basal side moderately angular but not drawn out. *Dimensions.* Height 1.7–2.1 mm; width 1.9–2.2 mm; h/w 0.85–1.00; umbilicus measured over the basal spiral cord 19–27 % of the shell width; number of whorls 4–4 3/4; height aperture 0.7–0.8 mm; width aperture 0.8–0.9 mm.

###### Habitat in Sabah and distribution.

Primary forest on sandstone and limestone soil, 400–1200 m alt. **Sabah: scattered localities.** Also in Kalimantan. Endemic to Borneo.

###### Cross diagnosis.

*Ditropopsis koperbergi* is characterized by its simple peristome: the aperture is not constricted, and there is no distinctive, rough sculpture close to it.

Elsewhere in Borneo, the conical spire and the angular whorl profile identify the species.

##### Ditropopsis tyloacron

Taxon classificationAnimaliaArchitaenioglossaCyclophoridae

Vermeulen, Liew & Schilthuizen sp. n.

http://zoobank.org/4BF26EBE-935F-4FF1-9BAD-0F62543E18AF

Figure 12

Ditropopsis sp. “BO-02”, Schilthuizen et al. 2002: 257–258.

###### Holotype.

Malaysia, Sabah, Tawau Province, Danum Valley Conservation Area (RMNH5003920).

###### Examined material from Sabah.

*Tawau Province*. Danum Valley Conservation Area (leg. UMS students, V 9880, BOR/MOL 313).

###### Description.

Shell very small, rather thick, somewhat translucent, white to pale (yellowish) green. Surface shiny or glossy. Spire almost flat, but apex protruding, slightly oblique. *Sculpture.* Radial sculpture: fine growth lines, locally grading into fine, densely placed riblets. Spiral sculpture on the last whorl: 6 very distinct cords: 1 supraperipheral and 1 peripheral, the latter widest and widely projecting, 1 basal and 3 umbilical; next to these a fine spiral striation on the lower surface. Sculpture continuing up to the peristome, but close to the peristome distorted and partly obliterated by densely placed, low radial riblets and increasingly coarse spiral threads. *Aperture.* Peristome simple, not expanded, distinctly constricted in fully adult shells, parietal side attached to the penultimate whorl in fully adult shells, basal side angular, slightly to distinctly drawn out. *Dimensions.* Height 1.4–1.65 mm; width 2.5–2.8 mm; h/w 0.52–0.56; umbilicus measured over the basal spiral cord 33–37 % of the shell width; number of whorls c. 4; height aperture 0.7–0.9 mm; width aperture 0.8–0.9 mm.

**Figure 12–13.**
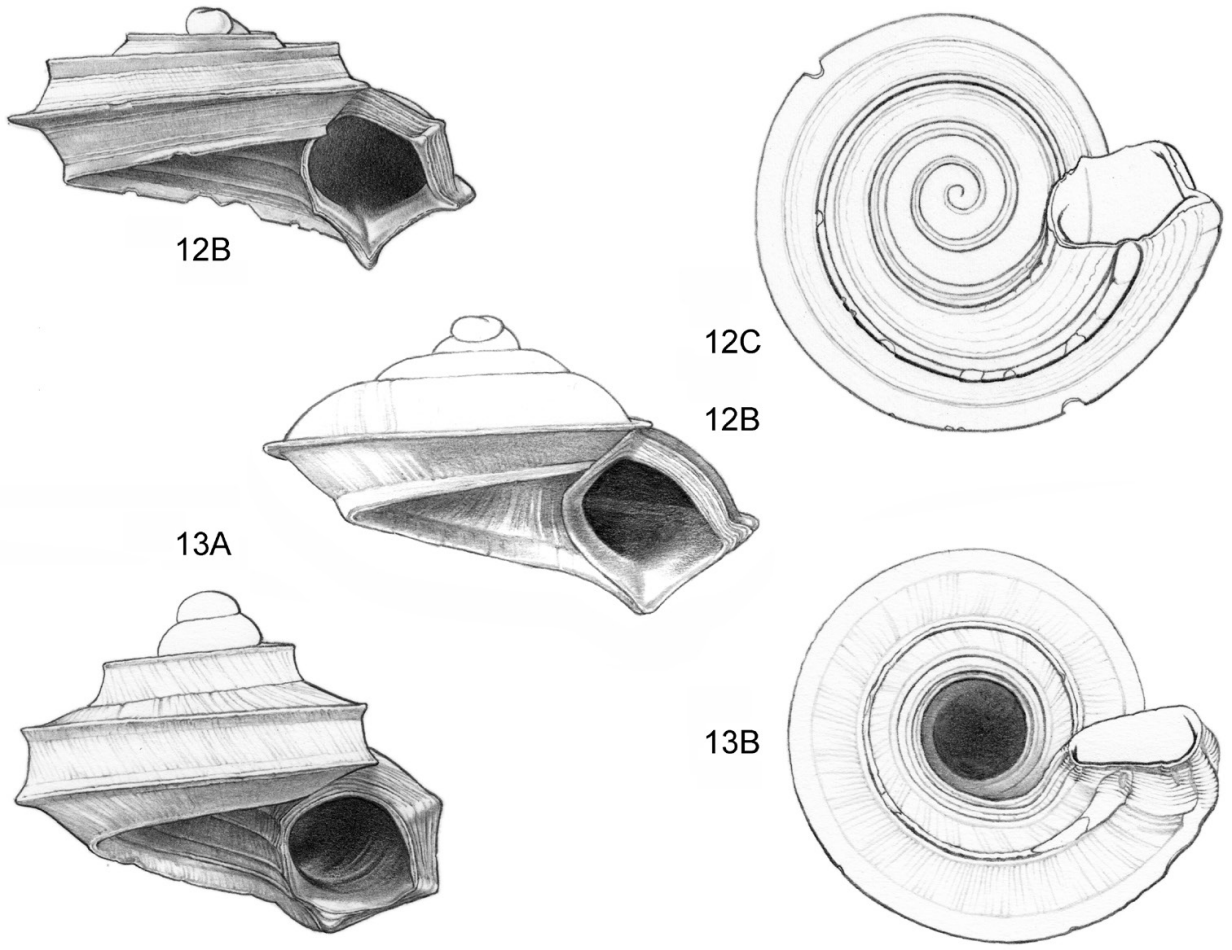
**12** *Ditropopsis tyloacron* sp. n. **12A** Frontal view, shell 1.6 mm high **12B** Frontal view, shell 1.5 mm high **12C** Umbilical view (**12A, 12C** Malaysia, Sabah, Tawau Province, Danum Valley Conservation Area, RMNH.5003920, holotype; **12B** Indonesia, Kalimantan Selatan, Meratus Mountains, c. 18 Km East of Barabai, Nateh near Batu Tangga, V 3003) **13** *Ditropopsis constricta* sp. n. **13A** Frontal view, shell 1.5 mm high **13B** Umbilical view (Malaysia, Sabah, Interior Province, Sepulut valley, Gua Sanaron, RMNH.5003919, holotype).

###### Habitat in Sabah and distribution.

Primary forest on limestone soil, rarely on sandstone, 0–600 m alt. **Sabah: S.E. part.** Also in Kalimantan, East part. Endemic to Borneo.

###### Cross diagnosis.

Well characterized among Sabah *Ditropopsis* by the low, almost flat spire with only the apex protruding. In Kalimantan the Sabah form (illustrated) locally grades into series with relatively smooth, large shells and with the supraperipheral and umbilical ridges partly or entirely missing. Such shells look a little like *Ditropopsis imadatei* and *Ditropopsis cincta*, but have a flatter spire and a wider umbilicus.

Elsewhere, *Ditropopsis moellendorfii* Boettger, 1891 and *Ditropopsis papuana* E.A. Smith, 1897 have similarly flat shells, but the first has a double peristome, and the second a more rounded spire.

###### Etymology.

The name refers to the shape of the apex [*tulos* (Gr.) = knob; *akros* (Gr.) = at the top; a knobhead, therefore].

##### Ditropopsis constricta

Taxon classificationAnimaliaArchitaenioglossaCyclophoridae

Vermeulen, Liew & Schilthuizen sp. n.

http://zoobank.org/AEC7D5EC-503D-4EF9-BC43-E44A360486AF

Figure 13

Ditropopsis constricta nomen nudum, Clements et al. 2008: 2761–2762.
Ditropopsis sp. “BO-01 [= V8027]”, Schilthuizen et al. 2002: 257–258; Schilthuizen et al. 2002b: 37, 41.

###### Holotype.

Malaysia, Sabah, Interior Province, Sepulut valley, Gua Sanaron (RMNH.5003919).

###### Examined material from Sabah.

*Interior Province*. Crocker Range N.P., near the km 59 marker on the road Kota Kinabalu-Tambunan (leg. J.J. Vermeulen, V 1183). Sepulut valley, Batu Temurung (leg. J.J. Vermeulen, V 8027); Gua Pungiton (leg. J.J. Vermeulen & M. Schilthuizen, V 8077); Gua Sanaron (leg. J.J. Vermeulen & M. Schilthuizen, V 7650). *Sandakan Province*. Danum Valley Field Centre (c. 60 km West of Lahad Datu): Clive Marsh grid W11/N0 (leg. Noel Tawatao, V 13483; leg. M. Schilthuizen, BOR/MOL 314, BOR/MOL 316). *Tawau Province*. Batu Baturong c. 50 km W.S.W. of Lahad Datu (leg. J.J. Vermeulen & H. Duistermaat, V 1850).

###### Description.

Shell very small, rather thick, somewhat translucent, off-white to pale greenish yellow. Surface shiny. Spire rather low-conical, apex slightly protruding, slightly oblique. *Sculpture.* Radial sculpture: fine growth lines, locally grading into fine, densely placed riblets. Spiral sculpture on the last whorl: 4–7 rather inconspicuous to very distinct cords: 1 supraperipheral and 1 peripheral, about equally wide, 1 basal and 1–4 umbilical, if fewer than 4 umbilicals then those closest to the basal cord absent; next to these a fine spiral striation on the lower surface. Sculpture continuing up to the peristome, but close to the peristome distorted and partly obliterated by densely placed, low radial riblets in fully adult shells. *Aperture.* Peristome simple, not expanded, distinctly constricted in fully adult shells, parietal side slightly detached from the penultimate whorl in fully adult shells, basal side slightly angular but not drawn out. *Dimensions.* Height 1.4–1.6 mm; width 1.8–2.1 mm; h/w 0.78–0.83; umbilicus measured over the basal spiral cord 31–32% of the shell width; number of whorls 3 7/8–4; height aperture 0.5–0.7 mm; width aperture 0.5–0.75 mm.

###### Habitat in Sabah and distribution.

Primary and secondary forest. Predominantly on limestone soil, scattered records from sandstone, 0–1000 m alt. **Sabah: South and S.E. part.** Also in Sarawak; Brunei; Kalimantan (East part). Endemic to Borneo.

###### Cross diagnosis.

Differs from *Ditropopsis koperbergi* by the constricted aperture, with a distinct radial sculpture. Juvenile shells of *Ditropopsis constricta*, without a fully grown aperture, have lower conical shells than *Ditropopsis koperbergi*, with a wider umbilicus.

###### Remarks.

The drawn specimen has relatively thick spiral cords, specimens with thinner cords occur.

###### Etymology.

The name refers to the constricted peristome.

#### Group 3. Teleoconch whorls (almost) entirely attached. Supraperipheral cord absent (check also *Ditropopsis tyloacron*)

##### Ditropopsis cincta

Taxon classificationAnimaliaArchitaenioglossaCyclophoridae

Vermeulen, Liew & Schilthuizen sp. n.

http://zoobank.org/4AB1D401-8A93-437D-8A6F-87EE13557DA2

Figure 14

###### Holotype.

Indonesia, Kalimantan Timur, Sangkulirang Peninsula, Liang Belana near Merabu (RMNH.5003921).

###### Examined material from Sabah.

*Sandakan Province*. Meliau Range: Southern slope of Gunung Meliau (leg. M. Schilthuizen, T.S. Liew & A. van Til, V 14344).

###### Description.

Shell very small, rather thick, somewhat translucent, corneous to brown. Surface shiny or glossy. Spire low-conical with convex sides, apex protruding, slightly oblique. *Sculpture.* Radial sculpture: fine growth lines, locally grading into fine, densely placed riblets. Spiral sculpture on the last whorl: 2 very distinct cords: 1 peripheral, widest and widely projecting, 1 basal; next to these a fine and inconspicuous spiral striation, particularly below the suture and around the basal cord. Sculpture continuing up to the peristome, but close to the peristome somewhat distorted because crossed by a few slightly more distinct growth lines. *Aperture.* Peristome simple, not expanded, parietal side attached to the penultimate whorl, basal side moderately angular and somewhat drawn out. *Dimensions.* Height 1.9–2.0 mm; width 2.5–2.7 mm; h/w 0.70–0.76; umbilicus measured over the basal spiral cord 40–48 % of the shell width; number of whorls 4–4 1/2; height aperture 0.8–0.9 mm; width aperture c. 1.0 mm.

**Figure 14–15.**
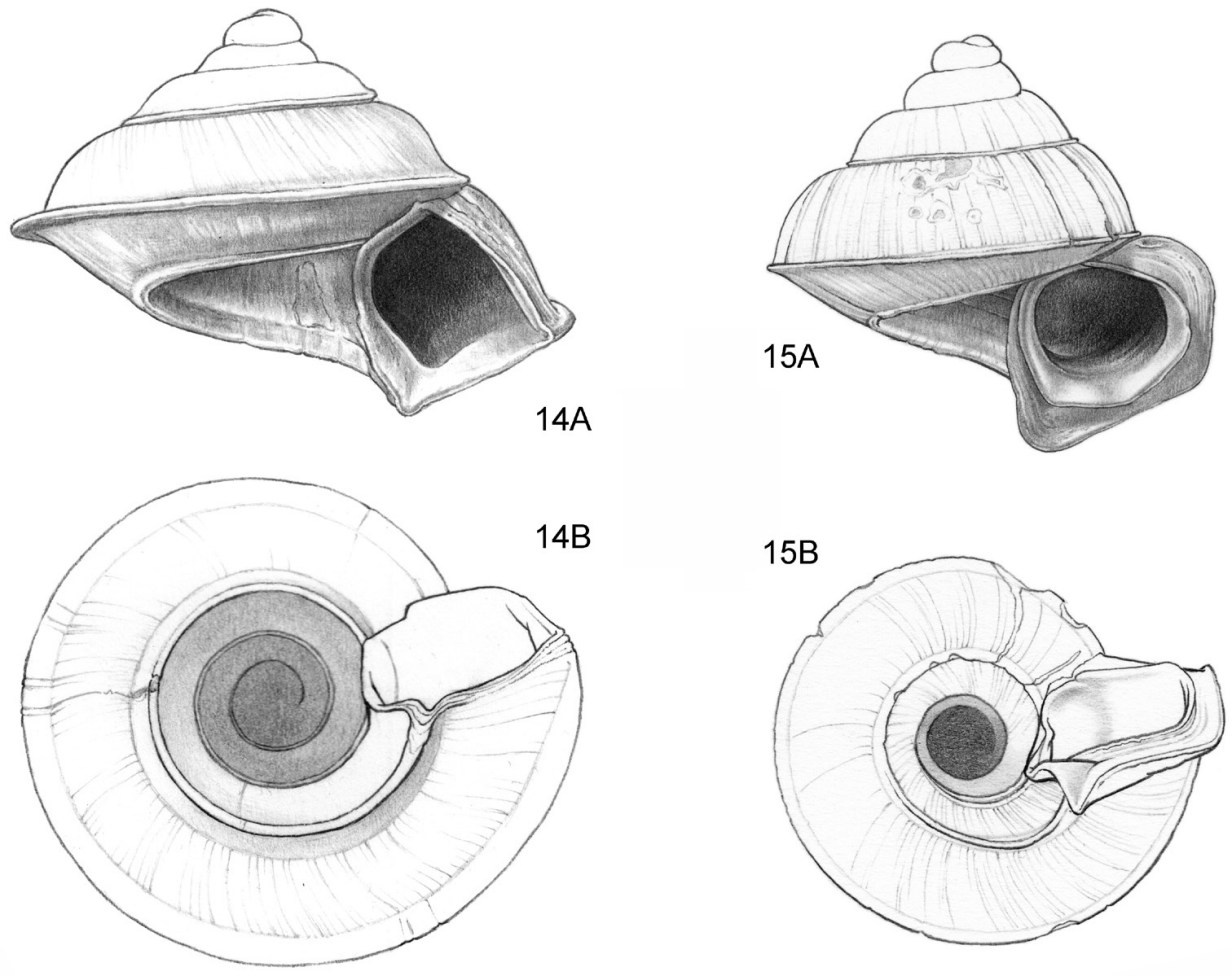
**14** *Ditropopsis cincta* sp. n. **14A** Frontal view, shell 2.0 mm high **14B** Umbilical view (Indonesia, Kalimantan Timur, Sangkulirang Peninsula, Liang Belana near Merabu, RMNH.5003921, holotype) **15** *Ditropopsis imadatei* (Habe, 1965) **15A** Frontal view, shell 2.1 mm high **15B** Umbilical view. (Malaysia, Sarawak, First Division, limestone hill near Bau, V 2621, shell partly reconstructed).

###### Habitat in Sabah and distribution.

Found in forest on ultrabasic bedrock. Elsewhere also on limestone bedrock, 400–500 m alt. **Sabah: Meliau Range.** Also in Kalimantan, East part. Endemic to Borneo.

###### Cross diagnosis.

The spire height of *Ditropopsis cincta* is intermediate between *Ditropopsis imadatei* and specimens of *Ditropopsis tyloacron* without a supraperipheral cord. Next to this, it differs from *Ditropopsis imadatei* by lacking the flaring outer peristome, and from *Ditropopsis tyloacron* by its narrower umbilicus.

###### Etymology.

The name refers to the peripheral keel [*cinctus* (L.) = girded].

##### Ditropopsis imadatei

Taxon classificationAnimaliaArchitaenioglossaCyclophoridae

(Habe, 1965)

Figure 15

Ditropis imadatei Habe, 1965: 117. Type from Brunei, Bandar Seri Begawan.
Ditropopsis sp. “nov.”, Schilthuizen 2004: 94.

###### Examined material from Sabah.

*Interior Province*. Crocker Range N.P., Ulu Kimanis, trail 5 of Crocker Range Scientific Expedition (leg. UMS Students, V 12747).

###### Description.

Shell very small, rather thick, somewhat translucent, white to pale greenish. Surface shiny or glossy. Spire conical with convex sides, apex somewhat protruding, hardly oblique. *Sculpture.* Radial sculpture: fine growth lines, locally grading into fine, densely placed riblets. Spiral sculpture on the last whorl: 5 cords: 1 peripheral, 1 basal, very distinct, and 2 much thinner and more inconspicuous umbilical cords; next to these a fine spiral striation locally present. Sculpture continuing up to the peristome. *Aperture.* Peristome double, the outer thickened and distinctly expanded, parietal side attached to the penultimate whorl in fully adult shells, basal side angular, distinctly drawn out; the inner peristome slightly protruding from the outer, slightly expanded, basal side only slightly angular and slightly drawn out. *Dimensions.* Height c. 2 mm; width 2.0–2.2 mm; h/w c. 0.9; umbilicus measured over the basal spiral cord 25–31% of the shell width; number of whorls c. 4 1/8; height aperture c. 1 mm; width aperture 1.0–1.2 mm.

###### Habitat in Sabah and distribution.

Primary forest on sandstone and limestone soil, 200–800 m alt. **Sabah: Crocker Range.** Also in Sarawak; Brunei; Kalimantan, East part. Endemic to Borneo.

###### Cross diagnosis.

Uniquely identified among the Sabah species by the double peristome.

Elsewhere, *Ditropopsis moellendorfi* Boettger, 1891 (Maluku) has a double peristome, but a much flatter shell with more numerous spiral cords.

#### Genus Japonia

Taxon classificationAnimaliaArchitaenioglossaCyclophoridae

Gould, 1859

Japonia Gould, 1859: 426.

##### Diagnosis for the Sabah species.

Shell small, white to pale corneous or pale greenish, usually marked with brown. Surface dull or shiny. Spire low to high-conical. Radial sculpture usually consisting of growth lines, locally grading into riblets. Spiral sculpture usually consisting of inconspicuous to distinct threads, sometimes with a peripheral keel. Peristome simple or double, expanded or not, with a notch in the angular corner. Height 4–12 mm. Umbilicus narrow to wide.

##### Cross diagnosis.

The genus *Leptopoma* Pfeiffer, 1847 (Borneo species revised by Vermeulen 1999), is most similar. *Japonia* is distinguished by the presence of a small notch in the angular corner of the peristome.

##### Remarks.

The Borneo species, numbering about 25, have not yet been revised. Nonetheless, we feel confident to describe one, highly distinctive, new species.

#### Japonia anceps

Taxon classificationAnimaliaArchitaenioglossaCyclophoridae

Vermeulen, Liew & Schilthuizen sp. n.

http://zoobank.org/C34702F6-1728-4681-8785-81892CB0BBD9

Figure 16

##### Holotype.

Malaysia, Sabah, Interior Province, Crocker Range N.P., Gua Laing c. 12 km North of Keningau (leg. J.J. Vermeulen, RMNH.5003957).

##### Examined material from Sabah.

*Interior Province*. Crocker Range N.P., Gua Laing c. 12 km North of Keningau (leg. J.J. Vermeulen, V 1104; M. Schilthuizen, BOR/MOL 200).

##### Description.

Shell small, rather solid, opaque, white to yellowish, with vaguely outlined pale brown radial blotches on the upper surface. Surface slightly shiny. Spire conical, top distinctly protruding, periphery sharply angular, whorls almost flat to slightly convex (in large specimens) above the periphery, slightly convex below; transition from basal to umbilical area somewhat less distinctly angular than the periphery. Whorls not channeled towards the suture. *Sculpture.* Radial sculpture: rather distinct, irregular growth lines, grading into similar radial riblets. Spiral sculpture on the last whorl: 1 distinct peripheral thread, 1 distinct basal thread, above the peripheral thread slight and inconspicuous spiral striation, below slightly more distinct striation, in the umbilical region still more distinct striation, grading into very fine, densely placed, thin threads; penultimate whorl with 1 distinct peripheral thread, just above the suture. *Aperture* subcircular, slightly angular on the palatal side. Peristome double, the inner hardly reflected, a rim which slightly protrudes from the outer; the outer peristome moderately reflected, somewhat concave. *Dimensions.* Height 4.7–6.2 mm; width 5.6–7.1 mm; h/w 0.84–0.88; umbilicus 0.7–1.0 mm wide, u/w 0.11–0.13; number of whorls 4 7/8–5 5/8; height aperture 2.5–2.8 mm; width aperture 2.7–3.5 mm. Periostracum without hairs.

**Figure 16–18.**
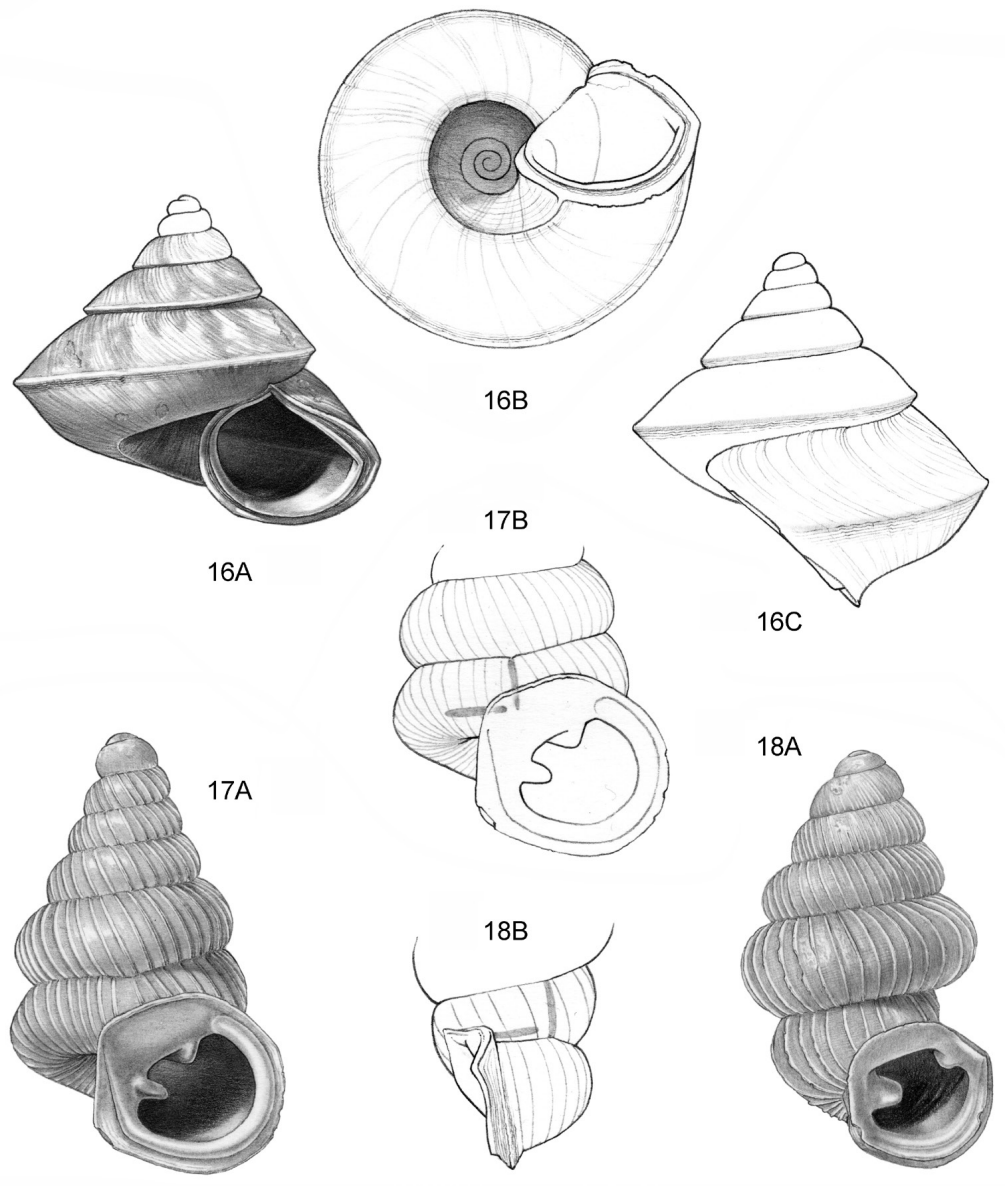
**16** *Japonia anceps* sp. n. **16A** Frontal view, shell 6.0 mm high **16B** Same shell, umbilical view **16C** Same shell, right lateral view (Malaysia, Sabah, Interior Province, Crocker Range N.P., Gua Laing c. 12 km North of Keningau, RMNH.5003957, holotype) **17** *Diplommatina bidentata* sp. n. **17A** Frontal view, shell 2.3 mm high **17B** Same shell, frontal view, with position of internal teeth indicated (Malaysia, Sabah, Sabah, Tawau Province, Batu Baturong c. 50 km W.S.W. of Lahad Datu, RMNH.5003954, holotype) **18** *Diplommatina tylocheilos* sp. n. **18A** Frontal view, shell 1.8 mm high **18B** Same shell, right lateral view, with position of internal teeth indicated (Malaysia, Sabah, Gunung Trusmadi slopes, Gua Loloposon, RMNH.5003953, holotype).

##### Habitat in Sabah and distribution.

Secondary forest on limestone bedrock. Alt. c. 500 m. Sabah: Crocker Range, Laing Cave near Keningau only. Endemic to Sabah.

##### Cross diagnosis.

Shares the angular periphery and the whorls which are only slightly convex above and below with *Japonia hyalina* Vermeulen & Junau, 2007, and *Japonia ditropis* Vermeulen & Junau, 2007, both from Sarawak. It differs from both by its larger size (shell height 4.7–6.2 mm, versus 2.0–3.5 mm), the presence of brown blotches on on the shell, as well as by the less conspicuous spiral sculpture apart from the peripheral and basal thread.

##### Etymology.

The name refers to the fact that the upper and lower surface of the last whorl are about equally convex [*anceps* (L.) = with two faces].

#### Family DIPLOMMATINIDAE

Taxon classificationAnimaliaArchitaenioglossaDiplommatinidae

Pfeiffer

##### Short description.

Shell (very) small, dextral or less frequently sinistral, ellipsoid to ovoid to conical, or cylindrical. Last whorl without a pore near the aperture; on the inside with a constriction. Coiling axis changing direction one to several times during development, often resulting in (partly) detached whorls. Sculpture: usually a distinct radial sculpture, spiral sculpture subordinate; rarely with spiral sculpture only, or without sculpture. Aperture rounded to somewhat angular above; peristome (distinctly) thickened. Umbilicus closed, or open, narrow. Operculum corneous, paucispiral (Family description based on Webster et al. 2012; Vermeulen 1991; Yamazaki 2013).

##### Habitat and distribution.

Rock and soil foragers, frequently associated with limestone substrates. Asia (from India, China, Japan Southwards) to Australia, and Oceania.

#### Genus Diplommatina

Taxon classificationAnimaliaArchitaenioglossaDiplommatinidae

Benson, 1849

Diplommatina Benson 1849: 193.

##### Remarks.

The Borneo species of *Diplommatina* have been revised in Vermeulen (1993). We add two new species. Both display a very unusual character in the genus apertural teeth on the parietal side and in the angular corner.

#### Diplommatina bidentata

Taxon classificationAnimaliaArchitaenioglossaDiplommatinidae

Vermeulen, Liew & Schilthuizen sp. n.

http://zoobank.org/F899243D-677B-410B-91ED-8227C3410E2A

Figure 17

##### Holotype.

Malaysia, Sabah, Tawau Province, Batu Baturong c. 50 km W.S.W. of Lahad Datu (leg. J.J. Vermeulen & H. Duistermaat, RMNH.5003954).

##### Examined material from Sabah.

*Tawau Province*. Batu Baturong c. 50 km W.S.W. of Lahad Datu (leg. J.J. Vermeulen & H. Duistermaat, V 7581).

##### Description.

Shell dextral, conical, with the last whorl or the last two whorls widest; sides flat or slightly convex. Suture impressed. *Constriction* level with the parietal side of the peristome, with 1 parietalis which continues as a lamella down to the aperture, 1 longitudinal palatalis slightly to the left of the parietal peristome, 1 transversal palatalis, 1 columellaris which continues as a lamella down to the aperture. Tuba 7/8 whorl. *Sculpture.* Radial ribs straight, distinct, rather low, rather wide, rather densely placed (7–11 ribs/0.5 mm on the penultimate whorl). Spiral striation present, inconspious, fine and dense. *Aperture* hardly tilted with regard to the coiling axis; columellaris large, slightly downwards directed; the distal end of the parietalis visible on the parietal side as a tooth. Peristome double, expanding; palatal side only slightly sinuous, without edge; basal side without edge; basal edge not sinuous; outer peristome not or hardly expanding beyond the inner; inner peristome with a palatal lip, free and erect on the columellar side, expanding on the parietal side. *Umbilicus* open, narrow. *Dimensions.* Height 1.9–2.3 mm; width 1.00–1.25 mm; umbilicus 0.05–0.20 mm wide; number of whorls 6–6 3/4; height and width aperture 0.25–0.30 mm.

##### Habitat in Sabah and distribution.

(Disturbed) primary forest on limestone bedrock, at c. 100 m alt. **Sabah: East coast, Baturong Hill only**. Endemic to Sabah.

##### Cross diagnosis.

Uniquely identified by the parietal lamella, which runs over the full length of the tuba and which is visible as a pearietal tooth deep in the aperture.

In Vermeulen (1993) it keys out next to *Diplommatina cacuminulus* Vermeulen, 1993. Yet it seems most similar to *Diplommatina toretos* Vermeulen, 1993; it shares the open umbilicus with this species.

##### Etymology.

The name refers to the two teeth in the aperture [*bidentata* (L.) = with two teeth].

#### Diplommatina tylocheilos

Taxon classificationAnimaliaArchitaenioglossaDiplommatinidae

Vermeulen, Liew & Schilthuizen sp. n.

http://zoobank.org/8EDBC334-75B1-4603-A1F0-8540D903321F

Figure 18

##### Holotype.

Malaysia, Sabah, Interior Province, Gunung Trusmadi slopes, Gua Loloposon (RMNH.5003953).

##### Examined material from Sabah.

*Interior Province*. Gunung Trusmadi slopes, Gua Loloposon (leg. J.J. Vermeulen, V 13227).

##### Description.

Shell dextral, fusiform, with the penultimate whorl widest. Suture impressed. *Constriction* level with the angular edge of the peristome, or up to 1/8 whorl beyond this point, towards the apex, with 1 parietalis, 1 longitudinal palatalis, 1 transversal palatalis, 1 columellaris which continues as a lamella down to the aperture. Tuba 1–1 1/8 whorl. *Sculpture.* Radial ribs straight, distinct, rather low, narrow, rather densely placed (6–9 ribs/0.5 mm on the penultimate whorl). Spiral striation present, rather distinct, moderately spaced. *Aperture* hardly tilted with regard to the coiling axis; columellaris large, patent; a second conical, obtuse tooth present on the palatal side, just below the angular edge. Peristome double, expanding; palatal side sinuous, with or without a slight edge; basal side without edge; basal edge not sinuous; outer peristome somewhat expanding beyond the inner; inner peristome with a palatal lip, free and erect on the columellar side, somewhat expanding on the parietal side. *Umbilicus* closed. *Dimensions.* Height 1.75–2.05 mm; width 0.95–1.15 mm; number of whorls 6–6 1/4; height aperture 0.30–0.40 mm; width aperture 0.35–0.45 mm.

##### Habitat in Sabah and distribution.

Found in primary forest on limestone soil. Alt. 900–1000 m. **Sabah: Gunung Trusmadi.** Endemic to Sabah.

##### Cross diagnosis.

Uniquely identified among Sabah *Diplommatina* species by the distinct, conical, obtuse tooth on the palatal peristome, close to the angular edge.

*Diplommatina unicrenata* Godwin Austen, 1897 [1897–1914], from India, Naga Hills is the only known species with a similar structure on the palatal peristome; it differs in having two teeth with a distinct furrow in between.

##### Etymology.

The name refers to the diagnostic character [*tulos* (Gr.) = knob; *cheilos* (Gr.) = lip].

### Unranked clade: NERITOMORPHA

#### Family HYDROCENIDAE

Taxon classificationAnimaliaArchaeogastropodaHydrocenidae

Troschel

##### Short description.

Shell (very) small, dextral, ellipsoid to ovoid to conical. Last whorl without a pore near the aperture, on the inside without a constriction. Sculpture absent, or inconspicuous to distinct spiral and/or radial sculpture present, often fused to nodular structures. Aperture rounded to somewhat angular above; peristome usually not thickened. Umbilicus usually covered by a thickened callus, an extension of the peristome. Operculum calcareous, paucispiral, with a central calcareous peg projecting from the inner surface (Family description adapted from Barker 2001; Kobelt 1876; Suter 1913; Herbert and Kilburn 2004; Thompson and Dance 1983).

##### Habitat and distribution.

Widespread in humid terrestrial environments, often associated with limestone habitats. Palearctis, Africa, but most diverse in Southeast Asia to Australia and Oceania.

##### Remarks.

The family is poorly known and has not been revised in the past 140 years and as a consequence, the status of the various genus names (including *Georissa*) is uncertain.

#### Genus Georissa

Taxon classificationAnimaliaArchaeogastropodaHydrocenidae

Blanford, 1864

Georissa Blanford, 1864: 463.

##### Remarks.

Thompson and Dance (1983) revised the Borneo species. We add two more.

#### Georissa leucococca

Taxon classificationAnimaliaArchaeogastropodaHydrocenidae

Vermeulen, Liew & Schilthuizen sp. n.

http://zoobank.org/DD72B156-C3BD-4537-A581-61D3A7A96222

Figure 19

##### Holotype.

Malaysia, Sabah, Interior Province, Sepulut valley, Gua Pungiton (RMNH.5003956).

##### Examined material from Sabah.

*Interior Province*. Sepulut valley, Gua Pungiton (leg. J.J. Vermeulen & M. Schilthuizen, V 8081, BOR/MOL 61); Gua Sanaron (leg. J.J. Vermeulen & M. Schilthuizen, V 8068). *Tawau Province*. Gua Madai c. 40 km S.S.W. of Lahad Datu (leg. J.J. Vermeulen & H. Duistermaat, V 1736).

##### Description.

Shell minute, rather solid, translucent, white; spire conical with convex sides to approx. ovoid; apex rounded. Surface with a silky luster. Whorls moderately convex, rounded; last whorl somewhat more narrowly rounded to slightly angular at the periphery, rounded below. *Protoconch* minutely rugulose. *Teleoconch*: radial sculpture consisting of growth lines, locally grading into low, thin riblets at irregular intervals. Spiral sculpture: rather distinct, somewhat spaced, fine, low, thin, rounded spiral threads, on the last whorl present above the periphery, but absent in a narrow area just below the suture. Aperture semi-elliptic, with the parietal side approx. straight or slightly convex; peristome widely extended on the parietal side, not reflected elsewhere, free on the columellar side. *Umbilicus* open, rimate, a narrow slit underneath the reflected columellar peristome. *Dimensions*: Height 0.70–1.05 mm; width 0.65–1.00 mm; number of whorls 2 1/4–3; height aperture 0.35–0.50 mm; width aperture 0.35–0.45 mm.

**Figure 19–20.**
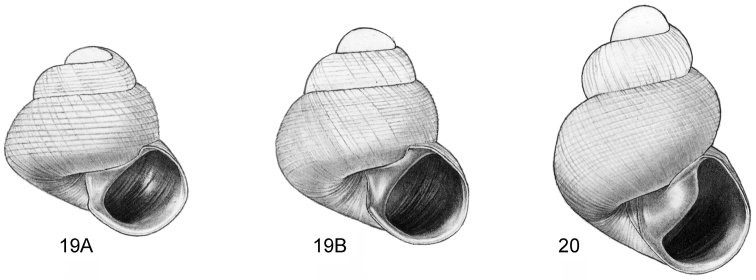
**19** *Georissa leucococca* sp. n. **19A** Frontal view, shell 0.85 mm high **19B** Frontal view, shell 1.05 mm high (**19A** Malaysia, Sabah, Interior Province, Sepulut valley, Gua Pungiton, RMNH.5003956, holotype **19B** Malaysia, Sabah, Interior Province, Sepulut valley, Gua Sanaron, V 8068) **20** *Georissa nephrostoma* sp. n. Frontal view, shell 1.35 mm high (Malaysia, Sabah, Sandakan Province, Kinabatangan valley, Batu Keruak 2 near Sukau, RMNH.5003955, holotype).

##### Habitat in Sabah and distribution.

Primary and secondary forest on limestone bedrock. Alt. up to c. 500 m. **Sabah: infrequent, a few localities only.** Also in Sarawak. Endemic to Borneo.

##### Cross diagnosis.

The small size, hardly over 1 mm high at 3 whorls, characterizes the species. *Georissa gomantongensis* E.A. Smith, 1893, has a similar spiral sculpture. Apart from being larger, it has orange to yellow shells and a nodular uppermost spiral thread, situated close to the suture.

The name refers to the white shell [*leucos* (Gr.) = white; *coccos* (Gr.) = kernel].

#### Georissa nephrostoma

Taxon classificationAnimaliaArchaeogastropodaHydrocenidae

Vermeulen, Liew & Schilthuizen sp. n.

http://zoobank.org/0D87FDD2-03F9-466F-9804-AA8948A99BA1

Figure 20

##### Holotype.

Malaysia, Sabah, Sandakan Province, Kinabatangan valley, Batu Keruak 2 near Sukau (RMNH.5003955).

##### Examined material from Sabah.

*Sandakan Province*. Kinabatangan valley, Batu Keruak 2 near Sukau (leg. J.J. Vermeulen & M. Schilthuizen, V 9795; leg. M. Salverda & H. van Oosten, BOR/MOL 1449, BOR/MOL 1450; leg. T.S. Liew & B. Elahan, BOR/MOL 1845); Batu Pangi (leg. J.J. Vermeulen & M. Schilthuizen, V 9833, BOR/MOL 1452); Batu Tai (not Bod Tai) near Gomantong (leg. J.J. Vermeulen & M. Schilthuizen, V 9832); Tandu Batu (leg. J.J. Vermeulen & M. Schilthuizen, V 9834); Unnamed limestone hills near Sukau Police Station (leg. T.S. Liew & B. Elahan, BOR/MOL 2186; leg. T.S. Liew, BOR/MOL 2153; leg. M. Schilthuizen, BOR/MOL 1451).

##### Description.

Shell minute, solid, opaque or nearly so, orange to white; spire conical with convex sides; apex rounded. Surface with a silky luster. Whorls distinctly convex, rounded; last whorl rounded at the periphery. *Protoconch* minutely rugulose. *Teleoconch*: radial sculpture in some specimens consisting of growth lines, only locally somewhat raised, in others consisting of densely placed, inconspicuous to rather distinct, low and rather flat ridglets with narrow, shallow grooves in between. Spiral sculpture: virtually absent in some specimens, in others consisting of densely placed to somewhat spaced, very fine, shallow grooves cutting into the raised elements of the radial sculpture, but sometimes most conspicuous in between the radial riblets on the apical whorls. Aperture reniform, with the parietal side distinctly concave; peristome extended on the parietal side, not reflected elsewhere, embedded in the shell surface on the columellar side. *Umbilicus* closed. *Dimensions*: Height 1.35–1.60 mm; width 0.90–1.15 mm; number of whorls 2 3/4–3 1/2; height aperture 0.60–0.65 mm; width aperture 0.50–0.55 mm.

##### Habitat in Sabah and distribution.

Primary and secondary forest on limestone bedrock. Alt. up to c. 100 m. **Sabah: Lower Kinabatangan valley only.** Endemic to Sabah.

##### Cross diagnosis.

Most similar to *Georissa similis* E.A. Smith 1893; distinguished by the deeply concave parietal peristome, giving the aperture a kidney-shaped outline. In *Georissa similis* the parietal side of the aperture is usually straight or slightly convex, seldomly a little concave. The sculpture of *Georissa nephrostoma* is on average less distinct than in *Georissa similis*, but both species include series of almost smooth specimens.

##### Etymology.

The name refers to the shape of the aperture [*nephros* (Gr.) = kidney; *stoma* (Gr.) = mouth].

### Clade: PULMONATA Cuvier

#### Family ARIOPHANTIDAE

Taxon classificationAnimaliaStylommatophoraAriophantidae

Godwin Austen

##### Short description.

Snails or semi-slugs. Shell (very) small to very large, dextral or sinistral, (low-)conical, ellipsoid, lenticular or discoid; semi-slugs with a (partly) reduced shell. Sculpture absent, or inconspicuous (rarely more distinct) spiral and/or radial sculpture present. Aperture without teeth or lamellae, peristome usually thin and not reflected. Umbilicus closed or open, narrow (Family description adapted from Baker (1941), and Solem (1966). In contrast to our earlier paper, Liew et al. 2009, we here include the Dyakiinae in Ariophantidae, rather than treating them as a separate family).

##### Habitat and distribution.

Generally forest species found on leaf litter and vegetation. Throughout South, East, and Southeast Asia, and Oceania, with a few species circumtropically introduced.

#### Genus Durgella

Taxon classificationAnimaliaStylommatophoraAriophantidae

Blanford, 1863

Durgella Blanford, 1863: 81.

#### ‘Durgella’ densestriata

Taxon classificationAnimaliaStylommatophoraAriophantidae

Vermeulen, Liew & Schilthuizen sp. n.

http://zoobank.org/72AE997E-70F9-4B26-A983-AC590D1D1A7F

Figure 21

##### Holotype.

Malaysia, Sabah, West Coast Province, Kinabalu N.P., summit trail, near Layang-layang at 2641 m (Leg. Liew T.S., BOR/MOL 6035).

Examined material from Sabah: *West Coast Province*, Kinabalu N.P., summit trail (Leg. Liew T.S., RMNH.5003941, BOR/MOL 6036); Kinabalu N.P., Kiau-Spurs route at 2416 m (Leg. T.S. Liew, J. Lapidin & Safrie, BOR/MOL 6034).

##### Description.

Shell small, membranous, leathery with only a thin calcareous layer on the inside, somewhat translucent, greenish or brownish, low-conical with slightly concave sides; apex narrowly rounded. Surface shiny. *Whorls* convex, rounded, suture impressed. *Protoconch* approx. smooth. *Teleoconch*, radial sculpture: irregularly spaced, somewhat raised growth lines, locally grading into some very fine, inconspicuous, densely placed riblets. Spiral sculpture: above the periphery with very fine, wavy, densely placed, shallow grooves. *Peristome* not reflected. *Umbilicus* closed. *Dimensions*: Height up to 5.5 mm; width up to 6.2 mm; diameter of the first three whorls c. 0.85 mm, c. 1.75 mm, c. 3.7 mm respectively; number of whorls up to c. 3 3/4, height aperture up to 3.8 mm; width aperture up to 3.5 mm.

**Figure 21.**
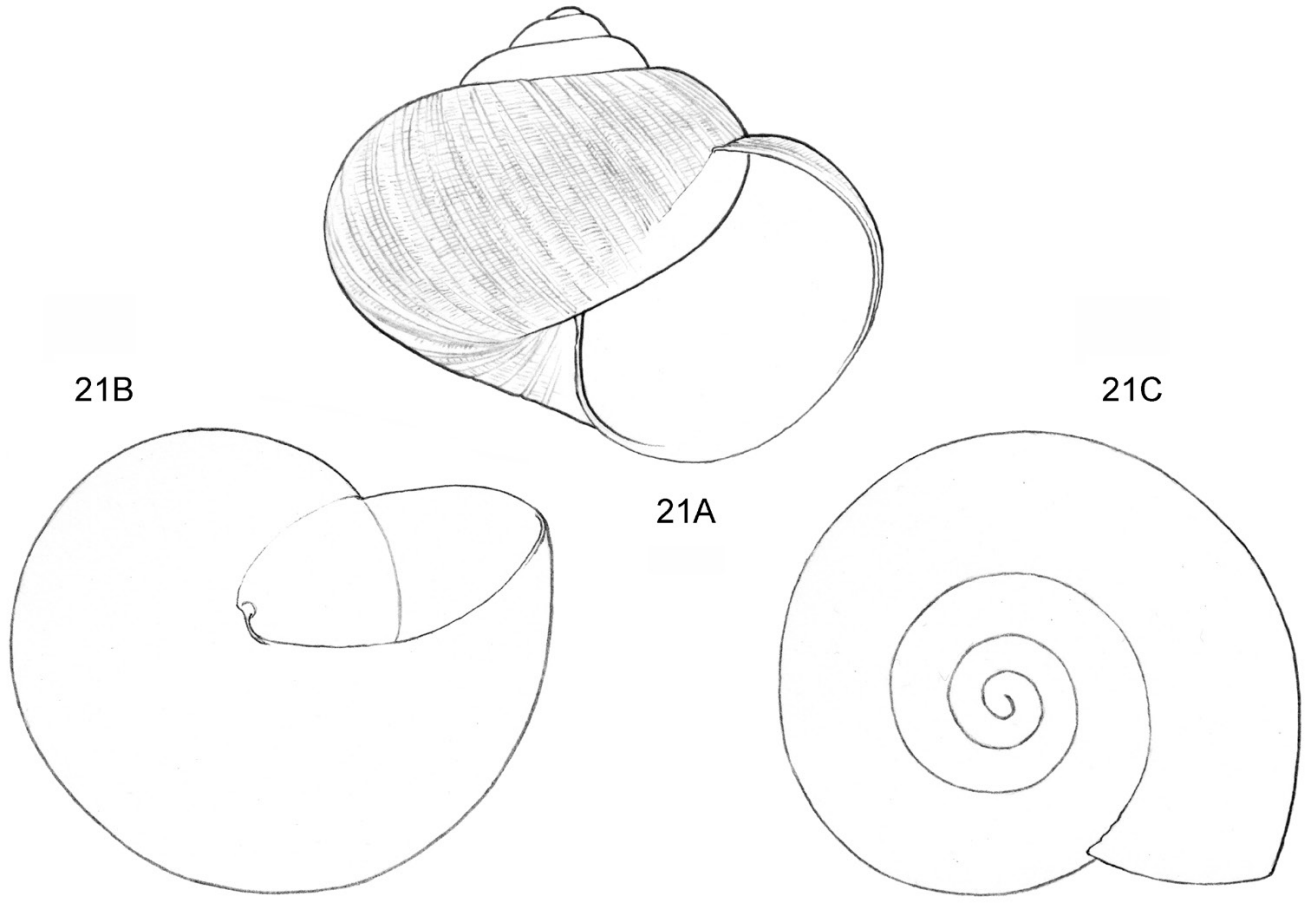
‘Durgella’ densestriata sp. n. **A** Frontal view, shell 5.5 mm high **B** Umbilical view **C** Apical view (Malaysia, Sabah, West Coast Province, Kinabalu N.P., summit trail, (BOR/MOL 6035, holotype, umbilical region reconstructed).

##### Habitat in Sabah and distribution.

Primary forest on sandstone or granodiorite bedrock. Alt. 2400–2700 m. **Sabah: Mount Kinabalu.** Endemic to Sabah.

##### Cross diagnosis.

We assume this is the shell of an ariophantid semi-slug. Among the genera already reported from Borneo, it fits best in *Durgella*, Blanford, with regard to the general shell shape and the number of whorls. It differs from *Durgella hosei* Godwin Austen, 1891, from Sarawak, as well as from continental Asiatic species listed in Blanford and Godwin Austen (1908: 213), Godwin Austen (1916: 555), and Solem (1966: 49) by having a low-conical spire with slightly concave sides, as well as by the fine and dense spiral striation.

##### Remarks.

Only damaged material was available to us, with some dried remnants of the animal.

##### Etymology.

The name refers to the shell sculpture [*densus* (L.) = dense; *striatus* (L.) = striated].

#### Genus Dyakia

Taxon classificationAnimaliaStylommatophoraAriophantidae

Godwin Austen, 1891

Dyakia Godwin Austen, 1891: 29.

#### Dyakia chlorosoma

Taxon classificationAnimaliaStylommatophoraAriophantidae

Vermeulen, Liew & Schilthuizen sp. n.

http://zoobank.org/AEBF9AF6-0F0F-43FA-B7E8-F58B145A1E27

Figure 22

##### Holotype.

Malaysia, Sabah, West Coast Province, Crocker Range N.P., Ulu Kimanis, along Keningau-Kimanis road (BOR/MOL 1364).

##### Examined material from Sabah.

*West Coast Province*. Crocker Range N.P., Ulu Kimanis, along Keningau-Kimanis road (leg. M. Schilthuizen, BOR/MOL 1363, BOR/MOL 1364), (leg. M. Schilthuizen & A.S. Cabanban, BOR/MOL 3006). Kinabalu N.P., Headquarters area, Liwagu Trail (leg. M. Schilthuizen, BOR/MOL 1362). Gunung Trusmadi slopes (leg. M. Schilthuizen & P. Koomen, BOR/MOL 919).

##### Description.

Shell medium-sized, very thin, translucent, pale yellowish green, conical with slightly concave (juveniles) to slightly convex (adults) sides; apex narrowly rounded. Surface glossy. *Whorls*: top whorls moderately convex, outer whorls flat above and convex below the periphery, periphery pinched and sharply keeled. *Protoconch* sculpture: a few flat radial folds, no other sculpture. *Teleoconch*, radial sculpture: first whorls slightly crenellated immediately below the suture, all whorls with more or less regularly spaced, slightly raised, weak growth lines, shell surface, particularly on the last whorl, distinctly undulated following the growth lines. Spiral sculpture absent, some shells with a inconspicuous spiral undulation on the last whorl. *Peristome* not reflected, not drawn out into a spur at the periphery. *Umbilicus* closed. *Dimensions*: Height up to 14 mm; width up to 32 mm; diameter of the first three whorls 1.9–2.1 mm, 3.5–4 mm, 8.0–8.3 mm respectively; number of whorls up to c. 4, height aperture up to 10.5 mm; width aperture up to 14.0 mm. *Animal* (pale) green including the tentacles, shining through the shell a bright green.

**Figure 22–23.**
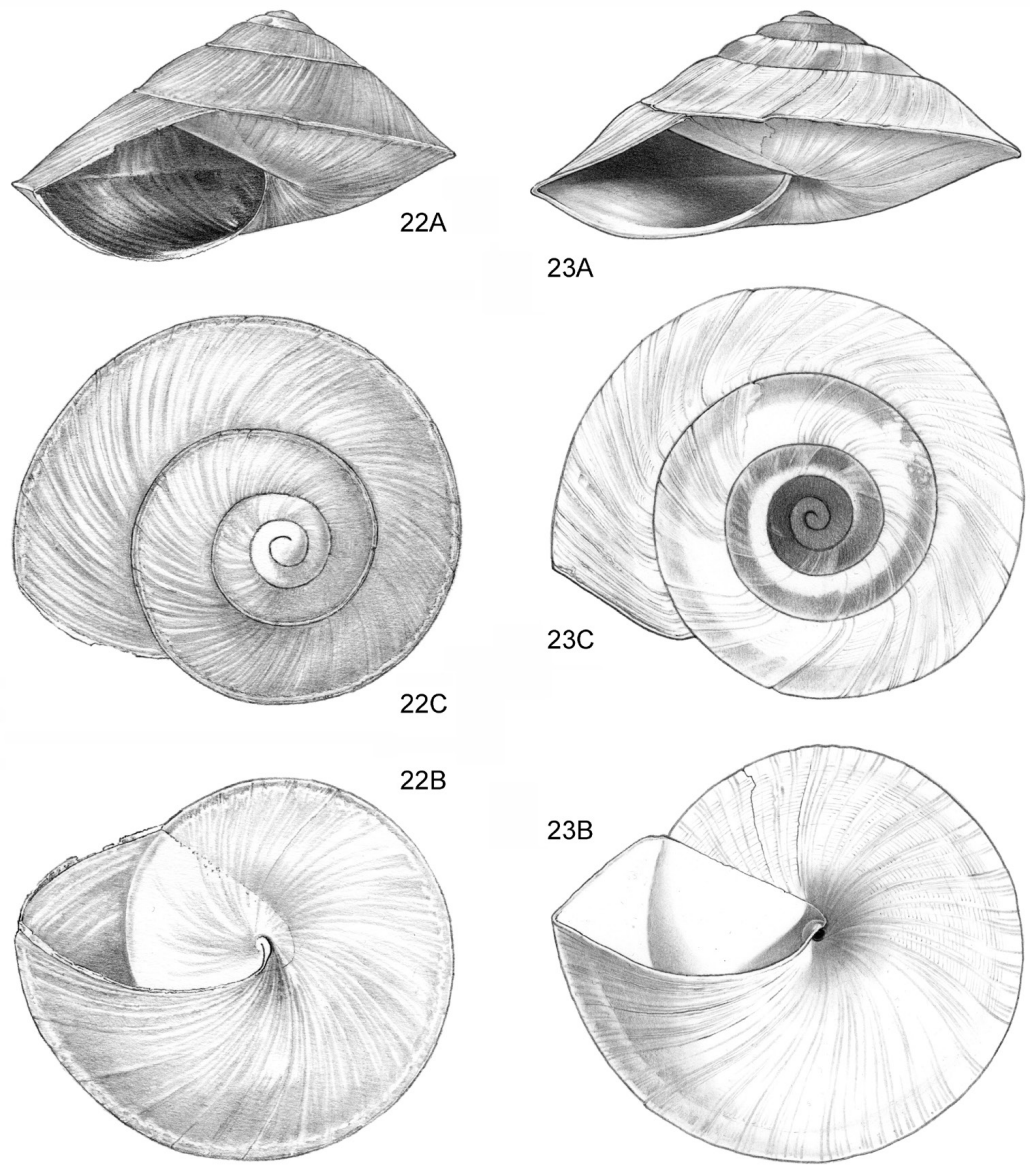
**22** *Dyakia chlorosoma* sp. n. **22A** Frontal view, shell 14 mm high **22B** Umbilical view **22C** Apical view (Malaysia, Sabah, West Coast Province, Crocker Range N.P., Ulu Kimanis, along Keningau-Kimanis road, BOR/MOL 1364, holotype) **23** *Dyakia moluensis* Godwin Austen, 1891 **23A** Frontal view, shell 12.5 mm high **23B** Umbilical view **23C** Apical view (Malaysia, Sarawak, Gunung Mulu National Park, V 10464).

##### Habitat in Sabah and distribution.

Primary forest on sandstone bedrock. Alt. 1200–1500 m. **Sabah: Crocker Range, Mount Kinabalu, Mount Trusmadi.** Endemic to Sabah.

##### Cross diagnosis.

Very similar to *Dyakia moluensis* Godwin Austen, 1891, the shell differs by being very thin, by having a somewhat higher spire, and a markedly undulate upper surface, by absence of any colour markings, and by absence of a fine, somewhat spaced spiral striation on the upper and lower surface. For comparison, we also provide illustrations of *Dyakia moluensis* (fig. 23).

Adult shells of *Rhinocochlis nasuta* (Metcalfe, 1851) differ by having the palatal side of the peristome drawn out into a tapering, slightly backwards curved beak. Juvenile shells without the beak are very similar to *Dyakia chlorosoma*, but differ by the presence of a fine, somewhat spaced, spiral striation. The animal of *Rhinocochlis nasuta* is green, with somewhat yellowish to orange red tentacles.

##### Etymology.

The name refers to the green body of the living animal [*chloros* (Gr.) = green; *soma* (Gr.) = body].

#### Genus Microcystina

Taxon classificationAnimaliaStylommatophoraAriophantidae

Mörch, 1872

Nanina subgenus *Microcystina* Mörch, 1872: 311. *Microcystina* (Mörch) Godwin Austen, 1882 (1882–1888): 11.

##### Diagnosis for the Sabah species.

Shell minute or very small, thin, lenticular, inflated-lenticular to depressed-ovoid; spire almost flat to moderately elevated. Surface shiny or glossy. Sculpture very fine, hardly prominent. Umbilicus open but very narrow, to closed, often partly or entirely covered by a minute, triangular spur protruding from the columellar corner of the peristome; this spur lacking or inconspicuous in juveniles. Dimensions: Width of adult shells 1.7–3.6 mm.

##### Remarks.

The genus *Microcystina* includes small (up to 3.6 mm wide) species with lenticular (depressed ovoid in *Microcystina physotrochus*) shells. Invariably, the shell surface seems smooth at a first impression, any sculpture present on the shells is very fine and inconspicuous. This distinguishes the smaller species of *Microcystina* (particularly *Microcystina sinica*) from sympatric, yet undescribed *Charopa* species (Charopidae), which have lenticular, minute shells (width of adult shells 0.9–1.9 mm) with a much more conspicuous sculpture. In several *Microcystina* species the umbilicus is partly or entirely covered by a minute, triangular spur protruding from the columellar corner of the peristome.

Borneo *Macrochlamys* and *Everettia* (Ariophantidae), and *Helicarion* (Helicarionidae) may have similarly shaped shells, including the inconspicuous sculpture, but they are larger (width of adult shells 8 mm or more).

Placement of *Microcystina* into the family Ariophantidae follows Schileyko (2003).

We provide a review of the Sabah species of *Microcystina*. We divide the genus into two informal groups.

#### Group 1. Shell white.

##### Microcystina microrhynchus

Taxon classificationAnimaliaStylommatophoraAriophantidae

Vermeulen, Liew & Schilthuizen sp. n.

http://zoobank.org/5192A533-FC42-49F4-8FE5-A354D53EB8AF

Figure 24

Microcystina microrhynchus nomen nudum, Clements et al. 2008: 2761–2762; Schilthuizen et al. 2013: online supplementary data.
Microcystina sp. “BO-01”, Schilthuizen et al. 2002a: 258; Schilthuizen et al. 2002b: 41–42; Schilthuizen and Vermeulen 2003: 96; Schilthuizen 2004: 94; Schilthuizen et al. 2011: 5.

###### Holotype.

Malaysia, Sabah, Interior Province, Gua Pungiton (RMNH.5003933).

###### Examined material from Sabah.

*Interior Province*. Upper Padas valley, Matang River South of Long Pasia (leg. J.J. Vermeulen, V 9815); Matang River South of Long Pasia, ridge on West bank (leg. J.J. Vermeulen, V 9824); Long Pasia (leg. T.S. Liew & Meckson, BOR/MOL 4355). Tenom (leg. M. Schilthuizen, BOR/MOL 3348). Crocker Range N.P., Gua Laing c. 12 km North of Keningau (leg. J.J. Vermeulen, V 1109, V 1114); West of the km 10 marker on the road Tambunan-Ranau, Mahua Waterfall (leg. J.J. Vermeulen & M. Schilthuizen, V 9749; leg. M. Schilthuizen, BOR/MOL 3137; leg. J. Schilthuizen, BOR/MOL 1081). Gunung Trusmadi slopes: Forestry chalet (leg. J.J. Vermeulen, V 13211); Gua Loloposon (leg. J.J. Vermeulen, V 13244). Pinangah valley, Batu Urun (= Bukit Sinobang) (leg. J.J. Vermeulen, V 7997, V 1143, V 1168, BOR/MOL 1067; leg. R. Haegens, V 5633; leg. M. Schilthuizen, BOR/MOL 1085). Pun Batu c. 30 km West of Sepulut (leg. J.J. Vermeulen, V 1284. Sepulut valley, Batu Punggul (leg. J.J. Vermeulen, V 1971; leg. M. Schilthuizen, BOR/MOL 1099); Batu Temurung (leg. J.J. Vermeulen, V 8041, BOR/MOL 1071; leg. M. Schilthuizen, BOR/MOL 1091); Bukit Tinagas, East end of Batu Punggul limestone (leg. J.J. Vermeulen & M. Schilthuizen, V 7629; leg. M. Schilthuizen, BOR/MOL 1096); Gua Pungiton (leg. J.J. Vermeulen & M. Schilthuizen, V 7548, BOR/MOL 1069); Gua Sanaron (leg. J.J. Vermeulen & M. Schilthuizen, V 7678; leg. M. Schilthuizen, BOR/MOL 1082, BOR/MOL 1098). *Kudat Province*. Balambangan Island, Kok Simpul (leg. J.J. Vermeulen & M. Schilthuizen, V 9530); South end, Batu Sireh (leg. J.J. Vermeulen & M. Schilthuizen, V 9544; leg. T.H. Liew, Sazilin & Ramlan, BOR/MOL 3689). Banggi Island, South end (leg. J.J. Vermeulen, V 1429; leg. M.A. Rahman, BOR/MOL 1094); Karakit Hill (leg. J.J. Vermeulen, V 1466; leg. J.J. Vermeulen & M. Schilthuizen, V 9470, V 9498); Pitas, Gua Mundau (leg. T.S. Liew & M. Schilthuizen, BOR/MOL 4370); Kg. Magnin (leg. T.S. Liew & M. Schilthuizen, BOR/MOL 4365). *Sandakan Province*. Meliau Range between 355–470 m (leg. T.S. Liew, M. Schilthuizen & A. van Til, BOR/MOL 3196, BOR/MOL 3197, BOR/MOL 3221). Kinabatangan valley, Batu Batangan (leg. M. Schilthuizen, BOR/MOL 1328); Batu Keruak 2 near Sukau (leg. J.J. Vermeulen & M. Schilthuizen, V 9790; leg. T.S. Liew & B. Elahan, BOR/MOL 1858, BOR/MOL 1892; leg. M. Salverda & H. van Oosten, BOR/MOL 1322, BOR/MOL 1319); Hill Resang River (leg. M. Schilthuizen, BOR/MOL 1324); Batu Mawas (leg. T.S. Liew & M. Schilthuizen, BOR/MOL 1969, BOR/MOL 2004; leg. M. Schilthuizen, BOR/MOL 1325); Batu Materis (; leg. T.S. Liew & B. Elahan, BOR/MOL 2094, BOR/MOL 2126); Batu Pangi (leg. J.J. Vermeulen & M. Schilthuizen, V 9641; leg. J.P. King, BOR/MOL 1326); Batu Tai (not Bod Tai) near Gomantong (leg. J.J. Vermeulen & M. Schilthuizen, V 9582; leg. M. Schilthuizen, BOR/MOL 1321; leg. T.S. Liew & B. Elahan, BOR/MOL 1926); Batu Tomanggong Kecil (leg. J.J. Vermeulen & M. Schilthuizen, V 9713); Batu Tulug (Batu Putih) along road Lahad Datu-Sandakan, North of bridge over Kinabatangan River (leg. J.J. Vermeulen & H. Duistermaat, V 1500); Gomantong Hill 30 km South of Sandakan (leg. J.J. Vermeulen & H. Duistermaat, V 1631; leg. M. Schilthuizen, BOR/MOL 1080; leg. A. van Til, BOR/MOL 3122, BOR/MOL 3276, BOR/MOL 3292, BOR/MOL 3302, BOR/MOL 3309, BOR/MOL 3321, BOR/MOL 3344; leg. T.S. Liew & J.P. King, BOR/MOL 3649); Tandu Batu (leg. J.J. Vermeulen & M. Schilthuizen, V 9628); Batu Tomanggong Besar (leg. M. Schilthuizen, BOR/MOL 1074, BOR/MOL 1317; leg. T.S. Liew & B. Elahan, BOR/MOL 2263, BOR/MOL 2294); Batu Tomanggong Kecil (leg. M. Salverda & H. van Oosten, BOR/MOL 1327; leg. T.S. Liew & B. Elahan, BOR/MOL 2036, BOR/MOL 2064); Unnamed hill near Sukau Police Station (leg. M. Schilthuizen, BOR/MOL 1320; leg. T.S. Liew, BOR/MOL 2166; leg. T.S. Liew & B. Elahan, BOR/MOL 2197). Segama valley, North end of limestone ridge on East bank Tabin River (leg. J.J. Vermeulen & M. Schilthuizen, V 7780, BOR/MOL 1087); Tabin Wildlife Reserve (leg. T. Kimsin, BOR/MOL 735; leg. M. Schilthuizen, BOR/MOL 1086). *Tawau Province*. Batu Baturong c. 50 km W.S.W. Lahad Datu (leg. J.J. Vermeulen & H. Duistermaat, V 1853); North slope (leg. J.J. Vermeulen, V 7588). Danum Valley (leg. M. Schilthuizen, BOR/MOL 1068, BOR/MOL 1073, BOR/MOL 1077; leg. UMS students, BOR/MOL 1075; leg. H.A. Rutjes, BOR/MOL 1079, BOR/MOL 1083;; leg. T. Kimsin & H.N. Chai, BOR/MOL 1084). Gua Madai c. 40 km S.S.W. of Lahad Datu (leg. J.J. Vermeulen & H. Duistermaat, V 1724); N.E. End (leg. J.J. Vermeulen, V 7700, BOR/MOL 1093). Segama valley, hill N.W. of crossing road Sandakan-Lahad Datu with the Segama River (leg. J.J. Vermeulen & H. Duistermaat, V 1679); ‘Kirk’s Cave’ 8 km North of Lahad Datu (leg. J.J. Vermeulen, V 1214); limestone hill on North bank Segama River, near bridge of road Sandakan to Lahad Datu (leg. J.J. Vermeulen, V 7501); Sabahmas Cave (leg. J.J. Vermeulen, V 7458, BOR/MOL 1089). Tawau Hills Park (leg. J.P. King, BOR/MOL 1316). Semporna area, Segarong Hills, Bukit Pababola, 25 km E.S.E. of Kunak (leg. J.J. Vermeulen & H. Duistermaat, V 1767); Bod Gaya Island (leg. T.S. Liew, Abdul & Ladja, BOR/MOL 4761, BOR/MOL 4775, BOR/MOL 4789, BOR/MOL 4897, BOR/MOL 4908). Tawau Hills N.P., path up to Bukit Bombalai (leg. J.J. Vermeulen, V 13156). *West Coast Province*. Crocker Range N.P., km 54 marker on the road Kota Kinabalu-Tambunan, Gunung Mas (leg. J.J. Vermeulen & M. Schilthuizen, V 9766; leg. M. Schilthuizen, BOR/MOL 1090). Kinabalu N.P., Headquarters area near entrance (leg. J.J. Vermeulen, V 9775; leg. M. Schilthuizen, BOR/MOL 1092); Sayap (leg. M. Schilthuizen, J. Schilthuizen & F. Schilthuizen, BOR/MOL 1323); Summit trail between 1616–3089 m (leg. T.S. Liew & J. Lapidin, BOR/MOL 4040; leg. T.S. Liew, BOR/MOL 4063, BOR/MOL 4031, BOR/MOL 4035, BOR/MOL 4066, BOR/MOL 4051, BOR/MOL 4055); Kiau-Spurs route between 3024 and 3088 m (leg. T.S. Liew, J. Lapidin & Safrie, BOR/MOL 4034, BOR/MOL 4069, BOR/MOL 4029); Kotal’s route between 1952 and 3416 m (leg. T.S. Liew & Jasilin, BOR/MOL 4032, BOR/MOL 4033, BOR/MOL 4059, BOR/MOL 4062, BOR/MOL 4038, BOR/MOL 4039, BOR/MOL 4043); Mesilau trail between 1948 and 2356 m (leg. T.S. Liew & J. Lapidin, BOR/MOL 4054, BOR/MOL 4050, BOR/MOL 4056, BOR/MOL 4058, BOR/MOL 4067; leg. T.S. Liew, BOR/MOL 4028, BOR/MOL 4048, BOR/MOL 4053); Sayap – Nunuhon trail between 960 and 2944 m (leg. T.S. Liew, Dominik, J. Lapidin & Jasilin, BOR/MOL 4037, BOR/MOL 4041, BOR/MOL 4042, BOR/MOL 4046, BOR/MOL 4064); Serinsim (leg. M. Schilthuizen, BOR/MOL 3070); Serinsim-Numbuyukon trail between 700 and 1680 m (leg. T.S. Liew, BOR/MOL 4044, BOR/MOL 4049, BOR/MOL 4057, BOR/MOL 4065, BOR/MOL 4068); Monggis-Tambuyukon trail between 1016 and 2360 m (leg. T.S. Liew, BOR/MOL 4030, BOR/MOL 4045), BOR/MOL 4052). Kota Kinabalu, Kiansom (leg. UMS students, BOR/MOL 1095). Mantanani Group, Pulau Mantanani Besar (leg. T.H. Liew, BOR/MOL 3714); Pulau Mantanani Kecil (leg. T.H. Liew, BOR/MOL 3733).

###### Description.

Shell very small, thin, translucent, white, lenticular; spire almost flat or slightly elevated. Surface glossy. Whorls slightly convex. *Protoconch*: smooth, sometimes with a few inconspicuous, scattered radial riblets only. *Teleoconch*: Spiral sculpture absent. Radial sculpture teleoconch: growth lines inconspicuous, next to these widely but irregularly spaced, shallow grooves, often alternating with areas of a much finer, more dense but irregularly spaced striation; sometimes the latter striation is predominant. *Umbilicus* partly or entirely covered by a minute, triangular spur protruding from the columellar corner of the peristome; this spur lacking or inconspicuous in juveniles; umbilical region moderately concave. *Dimensions*: Height up to 1.7 mm; width up to 3 mm; diameters of the first three whorls 0.5–0.8 mm, 0.9–1.5 mm, 1.5–2.5 mm respectively; number of whorls up to 4 1/8; height aperture up to 1.4 mm; width aperture up to 1.7 mm.

**Figure 24–26.**
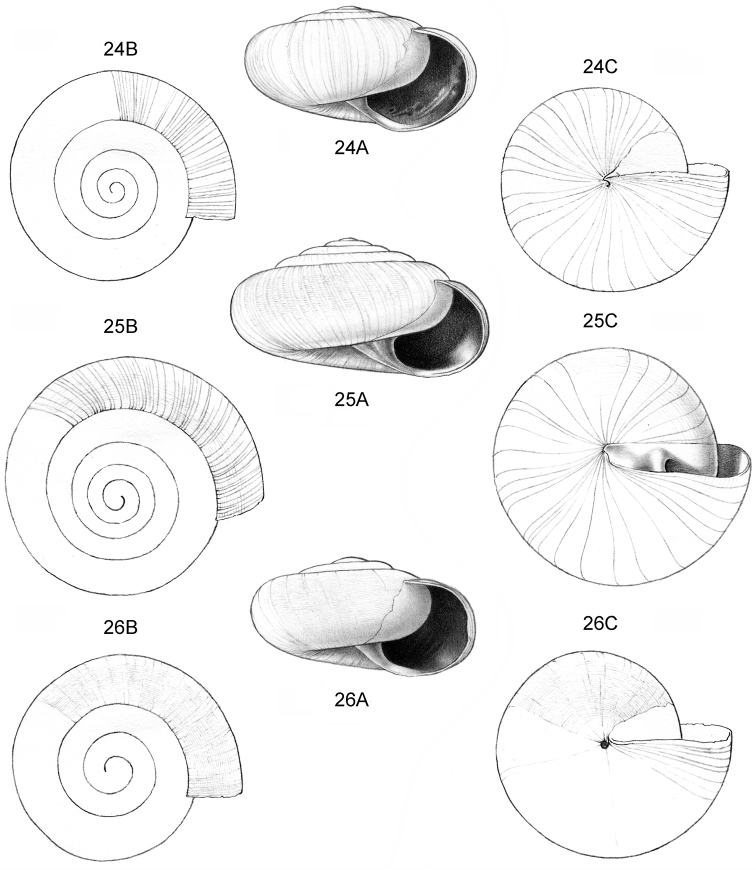
**24** *Microcystina microrhynchus* sp. n. **24A** Frontal view, shell 1.6 mm high **24B** Apical view **24C** Umbilical view (Malaysia, Sabah, Sepulut valley, Gua Pungiton; **24A** RMNH.5003933, Holotype; **24B, 24C** V 7548) **25** *Microcystina callifera* sp. n. **25A** Frontal view, shell 1.4 mm high **25B** Apical view **25C** Umbilical view (Malaysia, Sabah, Mantanani Group, Pulau Lungisan, RMNH.5003934, holotype) **26** *Microcystina striatula* sp. n. **26A** Frontal view, shell 0.8 mm high **26B** Apical view **26C** Umbilical view (Malaysia, Sabah, Segama valley, North end of limestone ridge on East bank Tabin River, RMNH.5003935, holotype).

###### Habitat in Sabah and distribution.

Rainforest, periodically dry, shrubby woodland, coastal woodland, secondary forest and other degraded vegetation; on limestone, sandstone and granitic soil, up to 3500 m alt. **Sabah**: **common and widespread.** Also in Brunei; Sarawak; Kalimantan. Endemic to Borneo.

###### Cross diagnosis.

Characterized among the Borneo *Microcystina* species with white shells by a complete lack of spiral striation. Usually it is also larger and has more rapidly expanding whorls.

Elsewhere, *Lamprocystus vitreiformis* Von Moellendorff, 1897, from Sumatra, Java, and Bali (see Vermeulen and Whitten 1998: 118) is most similar; *Microcystina microrhynchus* is characterized by the minute spur protruding from the columellar corner of the peristome and covering the umbilicus. *Microcystina chionodiscus* Vermeulen, 1996, from Bali (see Vermeulen and Whitten 1998: 115), has the first whorl consistently smaller (diameter 0.4–0.5 mm) than *Microcystina microrhynchus*.

###### Etymology.

The name refers to the spur covering the umbilicus [*mikros* (Gr.) = small; *rhynchos* (Gr.) = beak].

##### Microcystina callifera

Taxon classificationAnimaliaStylommatophoraAriophantidae

Vermeulen, Liew & Schilthuizen sp. n.

http://zoobank.org/0F22A242-E2FA-424B-B0F5-D9F980A0B1C1

Figure 25 , 29

Microcystina callifera nomen nudum, Clements et al. 2008: 2761–2762; Schilthuizen et al. 2013: online supplementary data.

###### Holotype.

Malaysia, Sabah, Mantanani Group, Pulau Lungisan (RMNH.5003934).

###### Examined material from Sabah.

*Kudat Province*. Banggi Island, South end, Karakit Hill (leg. J.J. Vermeulen & M. Schilthuizen, V 9499). *West Coast Province*. Mantanani Group, Pulau Lungisan (leg. M. Schilthuizen, V 9861; leg. M. Schilthuizen & T.H. Liew, BOR/MOL 3746); Pulau Mantanani Besar (leg. M. Schilthuizen, V 9849; leg. T.H. Liew, BOR/MOL 3715); Pulau Mantanani Kecil (leg. M. Schilthuizen, V 9856; leg. T.H. Liew, BOR/MOL 3732).

###### Description.

Shell very small, (rather) thin, almost opaque or slightly translucent, white, lenticular; spire almost flat to moderately elevated. Surface with a silky luster. Whorls slightly convex. *Protoconch* with inconspicuous, widely spaced, continuous, shallow, vaguely outlined spiral grooves, as well as with fine, widely spaced, shallow radial riblets towards the teleoconch. *Teleoconch*: upper surface with very fine, widely spaced, continuous, moderately raised, sharply outlined spiral threads; lower surface with equally fine, widely spaced, shallow grooves. This sculpture is sometimes rather distinct, sometimes inconspicuous and patchy. Radial sculpture teleoconch: growth lines inconspicuous, in some specimens locally grading into irregularly and rather densely placed to widely spaced, shallow grooves. *Umbilicus* closed by a callous extension of the columellar corner of the peristome, the same callus also narrowing the aperture on the columellar side. *Dimensions*: Height up to 1.5 mm; width up to 2.5 mm; diameters of the first three whorls 0.5–0.6 mm, 0.9–1.0 mm, 1.2–1.4 mm respectively; number of whorls up to 4 1/2; height aperture up to 1.0 mm; width aperture up to 1.4 mm.

###### Habitat in Sabah and distribution.

Coastal woodland on limestone soil. **Sabah**: **Islands: Mantanani Group and Banggi Island.** Endemic to Sabah.

###### Cross diagnosis.

Uniquely identified within *Microcystina* by the callus on the columellar side of the aperture. Substantial series of shells all have this callus, rendering the possibility of malformation unlikely. The spiral sculpture is usually more distinct, and more widely spaced than in *Microcystina striatula*.

###### Etymology.

The name refers to the callus in the aperture.

##### Microcystina striatula

Taxon classificationAnimaliaStylommatophoraAriophantidae

Vermeulen, Liew & Schilthuizen sp. n.

http://zoobank.org/A046ED7F-5388-496A-9A80-47BF1913A714

Figure 26 , 30

Microcystina striatula nomen nudum, Clements et al. 2008: 2761–2762.
Microcystina sp. “BO-02”, Schilthuizen and Vermeulen 2003: 96.

###### Holotype.

Malaysia, Sabah, Tabin Valley (RMNH.5003935).

###### Examined material from Sabah.

*Interior Province*. Crocker Range N.P., Gua Laing c. 12 km North of Keningau (leg. J.J. Vermeulen, V 1112). Gunung Trusmadi slopes: Gua Loloposon (leg. J.J. Vermeulen, V 13245). Pinangah valley, Batu Urun (= Bukit Sinobang) (leg. J.J. Vermeulen, V 1159). Pun Batu c. 30 km West of Sepulut (leg. J.J. Vermeulen, V 1287). Sepulut valley, Batu Punggul (leg. J.J. Vermeulen, V 1970); Batu Temurung (leg. M. Schilthuizen, BOR/MOL 730); Gua Pungiton (leg. J.J. Vermeulen & M. Schilthuizen, V 7550). *Kudat Province*. Banggi Island, South end (leg. J.J. Vermeulen, V 2515). *Sandakan Province*. Kinabatangan valley, Batu Materis (leg. M. Schilthuizen, BOR/MOL 1315); Batu Mawas (leg. T.S. Liew & M. Schilthuizen, BOR/MOL 2005); Batu Tomanggong Besar (leg. M. Schilthuizen, BOR/MOL 3611); Batu Tulug (Batu Putih) along road Lahad Datu-Sandakan, North of bridge over Kinabatangan River (leg. J.J. Vermeulen & H. Duistermaat, V 1499). Segama valley, North end of limestone ridge on East bank Tabin River (leg. J.J. Vermeulen & M. Schilthuizen, V 7779). *Tawau Province*. Batu Baturong c. 50 km W.S.W. Lahad Datu (leg. J.J. Vermeulen & H. Duistermaat, V 1854); North slope (leg. J.J. Vermeulen, V 7590). Gua Madai c. 40 km S.S.W. of Lahad Datu (leg. J.J. Vermeulen & H. Duistermaat, V 1723). Segama valley, hill N.W. of crossing road Sandakan-Lahad Datu with the Segama River (leg. J.J. Vermeulen & H. Duistermaat, V 1678); ‘Kirk’s Cave’ 8 km North of Lahad Datu (leg. J.J. Vermeulen, V 1228). Semporna area, Segarong Hills, Batu Tengar, 25 km E.S.E. of Kunak (leg. J.J. Vermeulen & H. Duistermaat, V 1822); Bukit Pababola, 25 km E.S.E. of Kunak (leg. J.J. Vermeulen & H. Duistermaat, V 1768); Bod Gaya Island (leg. T.S. Liew, Abdul & Ladja, BOR/MOL 4750); Sebangkat Island (leg. T.S. Liew & Abdul, BOR/MOL 4968). *West Coast Province*. Pulau Tiga in Kimanis Bay (leg. J.J. Vermeulen, V 11343). Kota Kinabalu, Kiansom (leg. UMS Tropical Malacology Course participants, BOR/MOL 3610).

###### Description.

Shell very small, thin, almost (slightly) translucent, white, lenticular; spire almost flat to slightly elevated. Surface with a silky luster. Whorls slightly to moderately convex. *Protoconch* with a very fine (hardly visible at 40 times magnification), densely placed, continuous, shallow, vaguely outlined spiral striation, and sometimes with fine, moderately spaced, shallow radial grooves towards the teleoconch only. *Teleoconch* with very fine (just visible at 40 times magnification) only slightly spaced, continuous, shallow, rather vaguely outlined spiral grooves on the upper surface; similar, but more densely placed grooves on the lower surface. This spiral sculpture is inconspicuous and patchy in some shells. Radial sculpture teleoconch: inconspicuous growth lines mainly, sometimes a few scattered, slight grooves. *Umbilicus* open, narrow, inner wall with an obtuse periomphalic edge; umbilical region distinctly concave. *Dimensions*: Height up to 1.1 mm; width up to 1.9 mm; diameters of the first three whorls 0.4–0.5 mm, 0.8–0.9 mm, 1.4–1.5 mm respectively; number of whorls up to 3 3/4; height aperture up to 0.8 mm; width aperture up to 1.0 mm.

###### Habitat in Sabah and distribution.

Rainforest, seasonally dry coastal forest and shrubby forest on limestone bedrock, up to 1000 m alt. **Sabah: scattered localities; less common than *Microcystina microrhynchus*.** Also in Kalimantan. Endemic to Borneo.

###### Cross diagnosis.

Differs at first sight from *Microcystina microrhynchus* by the presence of a fine spiral striation, giving the shell a soft, silky shine rather than a high gloss. Next to that, the umbilical area is more distinctly concave.

Elsewhere, *Microcystina chionodiscus* Vermeulen, 1996, from Bali, is similar, but *Microcystina striatula* has a more distinct spiral striation.

###### Remarks.

SEM images at 400 times magnification show that the areas in between the spiral grooves on the upper surface of the teleoconch have a ‘welded’ appearance.

###### Etymology.

The name refers to the sculpture [*striatula* (L.) = finely striated].

#### Group 2. Shell pale corneous, to yellowish green or brown

##### Microcystina sinica

Taxon classificationAnimaliaStylommatophoraAriophantidae

Von Moellendorff, 1885

Figure 27 , 31

Microcystina sinica Von Moellendorff, 1885: 386; Clements et al. 2008: 2761–2762; Liew et al., 2010: Appendix S1 in online Supporting Information; Schilthuizen et al., 2011: 5; Schilthuizen et al. 2013: online supplementary data. Type from China, Guangdong Province, Shiu Heng Hap.

###### Examined material from Sabah.

*Interior Province*. Upper Padas valley, Matang River South of Long Pasia (leg. J.J. Vermeulen, V 9813). Tenom (leg. M. Schilthuizen, BOR/MOL 3349). Crocker Range N.P., West of the km 10 marker on the road Tambunan-Ranau, Mahua Waterfall (leg. J.J. Vermeulen & M. Schilthuizen, V 9745). Sepulut valley, Gua Pungiton (leg. J.J. Vermeulen & M. Schilthuizen, V 7549); Gua Sanaron (leg. J.J. Vermeulen & M. Schilthuizen, V 8071). *Kudat Province*. Balambangan Island, Kok Simpul (leg. J.J. Vermeulen & M. Schilthuizen, V 9527, V 9515); South end, Batu Sireh (leg. J.J. Vermeulen & M. Schilthuizen, V 9565; leg. T.H. Liew, Sazilin & Ramlan, BOR/MOL 3690). Banggi Island, South end, Karakit Hill (leg. J.J. Vermeulen, V 1465; leg. J.J. Vermeulen & M. Schilthuizen, V 9501, V 9466; leg. M.A. Rahman, BOR/MOL 1127). *Sandakan Province*. Kinabatangan valley, Batu Batangan (leg. M. Schilthuizen, BOR/MOL 2347, BOR/MOL 2348); Batu Tai (not Bod Tai) near Gomantong (leg. J.J. Vermeulen & M. Schilthuizen, V 9579). *Tawau Province*. Gua Madai c. 40 km S.S.W. of Lahad Datu (leg. J.J. Vermeulen & H. Duistermaat, V 13457); N.E. End (leg. J.J. Vermeulen, V 7699; leg. M. Schilthuizen & A.S. Cabanban, BOR/MOL 3574). Segama valley, ‘Kirk’s Cave’ 8 km North of Lahad Datu (leg. J.J. Vermeulen, V 1220). Semporna area, Segarong Hills, Batu Tengar, 25 km E.S.E. of Kunak (leg. J.J. Vermeulen & H. Duistermaat, V 13460); Bukit Pababola, 25 km E.S.E. of Kunak (leg. J.J. Vermeulen & H. Duistermaat, V 1769). Semporna Islands Park: Pulau Bod Gaya (leg. M. Schilthuizen & A.S. Cabanban, V 12744) (leg. T.S. Liew, Abdul & Ladja, BOR/MOL 4723, BOR/MOL 4741, BOR/MOL 4751, BOR/MOL 4762, BOR/MOL 4776, BOR/MOL 4790, BOR/MOL 4803, BOR/MOL 4816, BOR/MOL 4841, BOR/MOL 4857, BOR/MOL 4876, BOR/MOL 4886, BOR/MOL 4898, BOR/MOL 4927, BOR/MOL 4937, BOR/MOL 4949, BOR/MOL 4958); Pulau Bohay Dulang (leg. M. Schilthuizen & A.S. Cabanban, V 12743, BOR/MOL 3535; leg. T.S. Liew, BOR/MOL 4608, BOR/MOL 4621, BOR/MOL 4645, BOR/MOL 4656, BOR/MOL 4669; leg. T.S. Liew & M. Ruf, BOR/MOL 4696, BOR/MOL 4710); Sebangkat Island (leg. T.S. Liew, Abdul & Ladja, BOR/MOL 4993); Selakan Island (leg. T.S. Liew, BOR/MOL 5005, BOR/MOL 5340); Tetagan Island (leg. T.S. Liew, Abdul & Ladja, BOR/MOL 5021, BOR/MOL 5029). *West Coast Province*. Kinabalu N.P., Poring Hot Springs, along path to waterfall (leg. J.J. Vermeulen, V 13005); Serinsim (leg. M. Schilthuizen, BOR/MOL 3102). Pulau Tiga in Kimanis Bay (leg. J.J. Vermeulen, V 11347). Mantanani Kecil Island (leg. T.H. Liew, BOR/MOL 3734).

###### Description.

Shell minute, thin, somewhat translucent, pale yellowish to pale corneous, lenticular; spire moderately elevated, conical with a rounded apex. Surface moderately glossy, or with a silky shine. Whorls moderately convex. *Protoconch* with a fine, moderately spaced spiral striation consisting of rows of minute pits (barely visible at 40 times magnification) which are arranged in a reticulate pattern, sometimes traces of radial riblets (barely visible at 40 times magnification). *Teleoconch*: Spiral sculpture sometimes approx. absent or very fine (just visible at 40 times magnification), rather widely spaced, continuous, shallow, rather sharply outlined grooves present locally or over the entire shell. Radial sculpture teleoconch: inconspicuous growth lines, next to these inconspicuous to distinct, well-spaced to densely placed shallow grooves, often at irregular intervals. *Umbilicus* closed, or open and very narrow; columellar side of the peristome somewhat thickened but not covering the umbilicus; umbilical region slightly concave. *Dimensions*: Height up to 1.1 mm; width up to 1.7 mm; diameters of the first three whorls 0.3–0.5 mm, 0.5–0.8 mm, 0.8–1.3 mm respectively; number of whorls up to 4 18; height aperture up to 0.9 mm; width aperture up to 0.9 mm.

**Figure 27–28.**
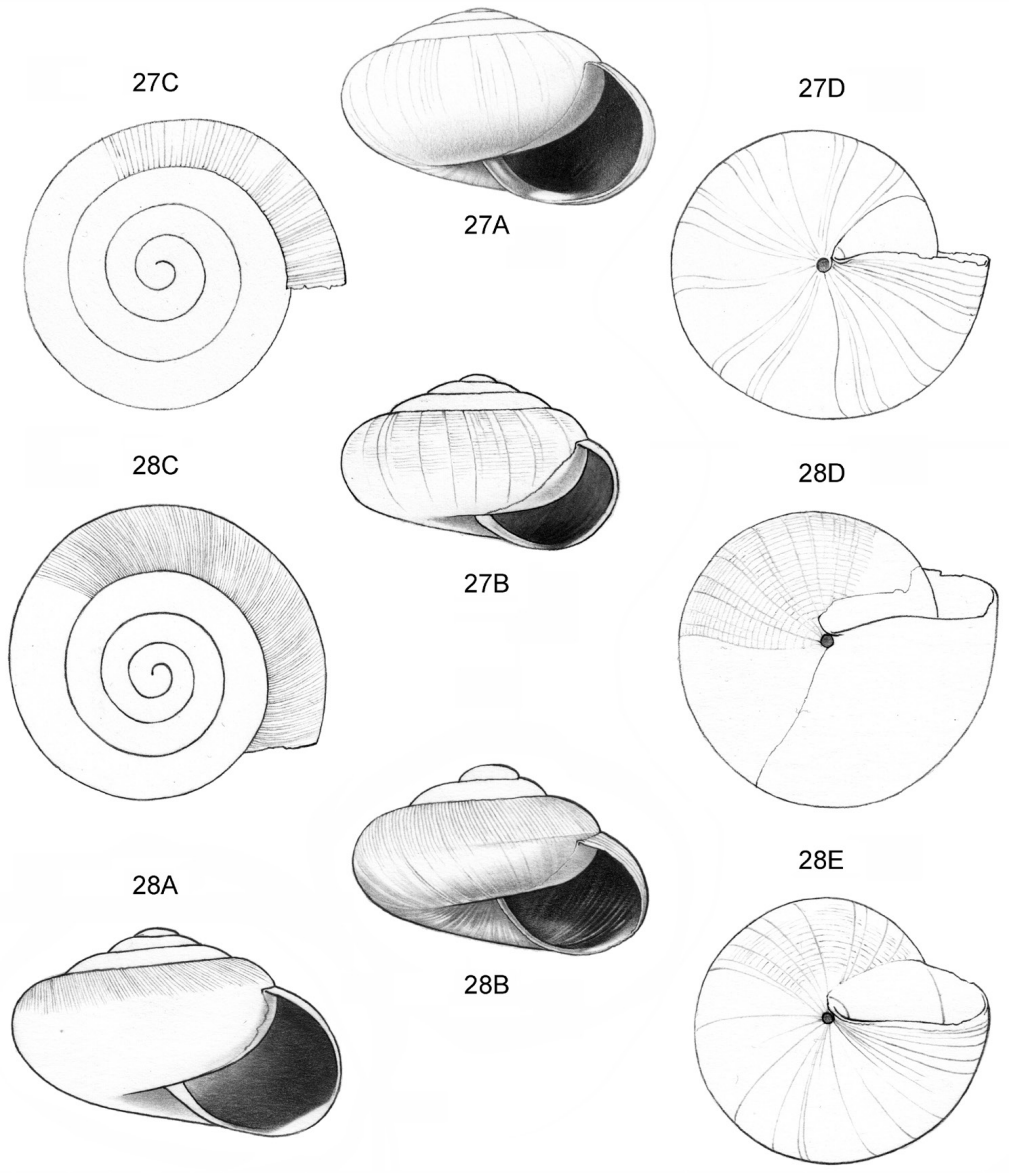
**27** *Microcystina sinica* Von Moellendorff, 1885. **27A** Frontal view, shell 0.6 mm high **27B** Frontal view, shell 1.0 mm high **27C** Apical view **27D** Umbilical view (**27A, 27C, 27D** Malaysia, Sabah, Sepulut valley, Gua Sanaron, V 8071 **27B** Indonesia, Kalimantan Selatan, Meratus Mts., West flank, Nateh near Batu Tangga, c. 18 km E Barabai, V 3061) **28** *Microcystina consobrina* Van Benthem Jutting, 1959. **28A** Frontal view, shell 1.4 mm high **28B** Frontal view, shell 1.3 mm high **28C** Apical view **28D** Umbilical view **28E** Umbilical view (**28A, 28C, 28D** Indonesia, Sumatra, Berastagi, ZMA, s.n., holotype **28B, 28E** Malaysia, Sabah, Kinabalu N.P., near Kotal route, V 14325).

**Figure 29–32.**
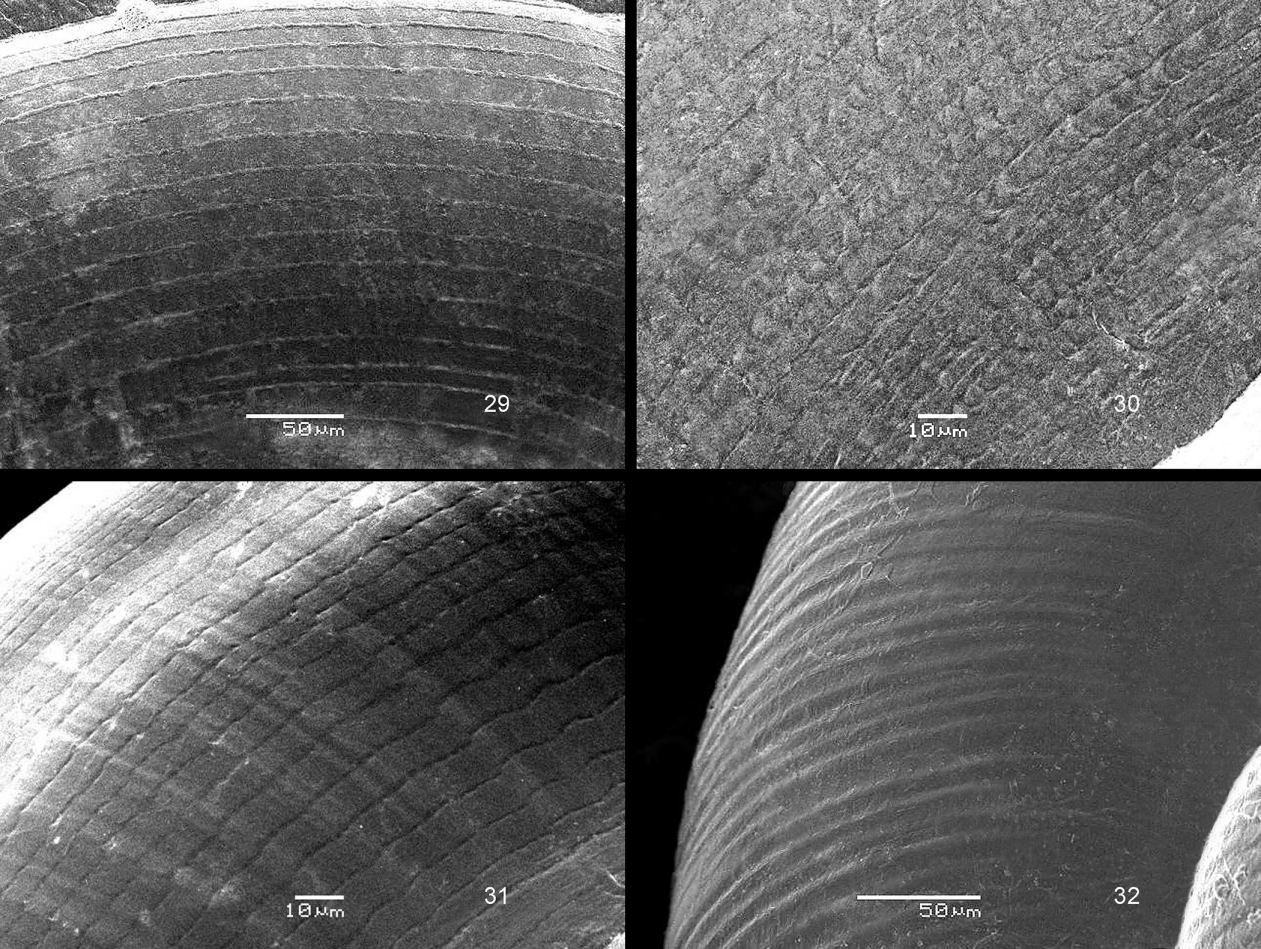
**29** *Microcystina callifera* sp. n. SEM image of the third whorl (Malaysia, Sabah, Mantanani Islands, Pulau Lungisan, BOR/MOL 3746) **30** *Microcystina striatula* sp. n. SEM image of the third whorl (Malaysia, Sabah, Pulau Tiga, BOR/MOL 1100, lost) **31** *Microcystina sinica* Von Moellendorff, 1885. SEM image of the third whorl (material and data lost) **32** *Microcystina consobrina* Van Benthem Jutting, 1959. SEM image of the third whorl (Malaysia, Sabah, Kinabalu N.P., Kotal route between 2292 and 3112 m alt., BOR/MOL 4003).

###### Habitat in Sabah and distribution.

Primary and secondary forest, coastal woodland on limestone, sandstone and volcanic bedrock, up to 1100 m alt. Elsewhere also in more severely degraded environments such as *Imperata* grass fields. **Sabah: rather common in East Sabah, scattered localities in West Sabah.** Also in Sarawak; Kalimantan. Distribution: widespread, from South China to Java; Eastwards to Irian Jaya.

###### Cross diagnosis.

Shares the small size, slowly expanding whorls, and pitted protoconch with *Microcystina muscorum*; it differs by having a continuous spiral striation on the teleoconch. Shells entirely without spiral striation can be distinguished by the colour, as well as by the absence of shallow, widely spaced radial grooves, as occur in *Microcystina muscorum*.

*Microcystina striatula* and *Microcystina gratilla* have more rapidly expanding whorls, and have a flatter shell; *Microcystina striatula* also has a white shell.

##### Microcystina consobrina

Taxon classificationAnimaliaStylommatophoraAriophantidae

Van Benthem Jutting, 1959

Figure 28 , 32

Microcystina consobrina Van Benthem Jutting, 1959: 145; Liew et al. 2010: Appendix S1 in online Supporting Information. Type from Indonesia, Sumatra, Brastagi.

###### Examined material from Sabah.

*West Coast Province*. Kinabalu N.P., Kotal route between 2292 and 3112 m (leg. T.S. Liew, J. Lapidin, Safrie & Jasilin V 14326; V 14325, BOR/MOL 4003).

###### Description.

Shell very small, thin, translucent, pale yellowish green to yellowish brown, approx. lenticular; spire moderately elevated, conical with a rounded apex. Surface shiny, glossy below. Whorls moderately to distinctly convex. *Protoconch* without spiral sculpture; with fine, densely placed, low radial riblets towards the teleoconch, but slightly coarser than on the teleoconch. *Teleoconch*: Spiral sculpture above the periphery absent or inconspicuous, below the periphery with numerous fine, well-spaced, continuous, shallow, rather vaguely outlined grooves. Radial sculpture teleoconch: above the periphery with very fine, very densely and regularly placed, low riblets; below the periphery with some irregularly spaced growth lines only. *Umbilicus* open, narrow; columellar side of the peristome somewhat thickened but not covering the umbilicus. *Dimensions*: Height up to 1.4 mm; width up to 2.3 mm; diameters of the first three whorls 0.5–0.6 mm, 0.8–1.0 mm, 1.4–1.6 mm respectively; number of whorls up to 4 1/4; height aperture up to 1 mm; width aperture up to 1.2 mm.

###### Habitat in Sabah and distribution.

Primary forest on granodiorite soil, 2200–3200 m alt., elsewhere at 0–1700 m alt. **Sabah: Mt. Kinabalu.** Distribution: Sumatra; Bali.

###### Cross diagnosis.

Uniquely characterized within Group 2 by the fine and dense radial ribbing above the periphery.

Elsewhere, *Lamprocystis exigua* Von Moellendorff, 1897, from Java to Flores, (see Van Benthem Jutting 1950: 447), is most similar. It differs by having a larger first whorl (diameter 0.7–0.8 mm).

###### Remarks.

The description includes extralimital material as well. The Kinabalu material has slightly coarser radial riblets than the type.

##### Microcystina planiuscula

Taxon classificationAnimaliaStylommatophoraAriophantidae

Vermeulen, Liew & Schilthuizen sp. n.

http://zoobank.org/6A1CB406-5356-4ABA-800C-732FB1DFF02A

Figure 33

###### Holotype.

Malaysia, Sabah, Mt. Trusmadi, Gua Dawaras (RMNH.5003936).

###### Examined material from Sabah.

*Interior Province*. Gunung Trusmadi slopes: Gua Loloposon (leg. J.J. Vermeulen, V 13220); Gua Dawaras (leg. M. Schilthuizen, V 12742; leg. M. Schilthuizen, V 9868).

###### Description.

Shell very small, thin, translucent, yellowish brown, discoid-lenticular; spire almost flat. Surface glossy. Whorls moderately convex. *Protoconch* with 7–10 very fine, widely and regularly spaced spiral grooves. *Teleoconch*: very fine, widely and sometimes irregularly spaced spiral grooves on the upper and lower surface; these sometimes (partly) absent on the outer whorls. Radial sculpture teleoconch: scattered, inconspicuous growth lines; very slight, irregularly spaced folds just below the suture. *Umbilicus* open, narrow; columellar side of the peristome somewhat thickened but not covering the umbilicus. *Dimensions*: Height up to 1.5 mm; width up to 2.6 mm; diameters of the first three whorls 0.7–0.8 mm, 1.2–1.3 mm, c. 2.3 mm respectively; number of whorls up to 3 1/8; height aperture up to 1.2 mm; width aperture up to 1.4 mm.

**Figure 33–34.**
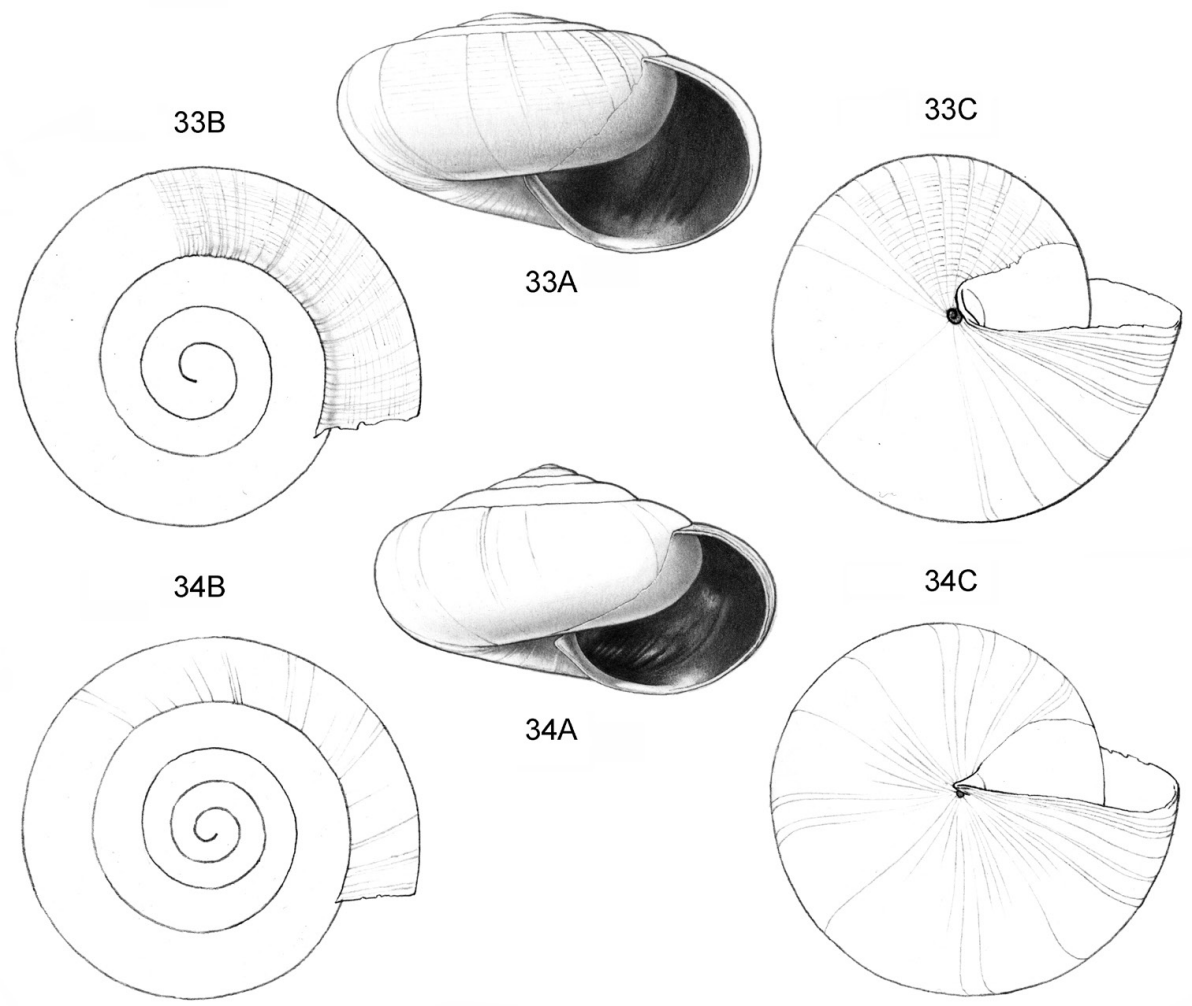
**33** *Microcystina planiuscula* sp. n. **33A** Frontal view **33B** Apical view **33C** Umbilical view (Malaysia, Sabah, Gunung Trusmadi, Gua Dawaras, RMNH.5003936, holotype) **34** *Microcystina appendiculata* (Von Moellendorff, 1893). **34A** Frontal view, shell 1.5 mm high **34B** Apical view **34C** Umbilical view (Malaysia, Sabah, Segama valley, North end of limestone ridge on East bank Tabin River, V 7781).

###### Habitat in Sabah and distribution.

Primary forest on limestone soil, 1600–1700 m alt. **Sabah: Mt. Trusmadi only.** Endemic to Sabah.

###### Cross diagnosis.

Identified by the distinctly more rapidly expanding whorls than in any other species in Group 2.

###### Etymology.

The name refers to the shell shape [*planiusculus* (L.) = somewhat flattened].

##### Microcystina appendiculata

Taxon classificationAnimaliaStylommatophoraAriophantidae

(Von Moellendorff, 1893)

Figure 34

Lamprocystis appendiculata Von Moellendorff, 1893: 72. Type from Philippines, Leyte.
Microcystina lissa nomen nudum, Clements et al. 2008: 2761–2762; Schilthuizen et al. 2013: online supplementary data.
Microcystina sp. “BO-03”, Schilthuizen et al. 2002a: 257–258; Schilthuizen et al. 2002b: 37, 41–42; Schilthuizen and Vermeulen 2003: 96.

###### Examined material from Sabah.

*Interior Province*. Sepulut valley, Batu Punggul (leg. J.J. Vermeulen, V 1972); Batu Temurung (leg. J.J. Vermeulen, V 8042); Bukit Tinagas, East end of Batu Punggul limestone (leg. J.J. Vermeulen & M. Schilthuizen, V 7628; leg. M. Schilthuizen, BOR/MOL 1102). *Sandakan Province*. Kinabatangan valley, Batu Keruak 2 near Sukau (leg. J.J. Vermeulen & M. Schilthuizen, V 9789; leg. M. Salverda & H. van Oosten, BOR/MOL 1348); Batu Mawas (leg. M. Schilthuizen, BOR/MOL 1333; leg. T.S. Liew & M. Schilthuizen, BOR/MOL 1970, BOR/MOL 2006); Batu Materis (leg. M. Schilthuizen, BOR/MOL 1330; leg. T.S. Liew & B. Elahan, BOR/MOL 2095, BOR/MOL 2127); Batu Pangi (leg. J.J. Vermeulen & M. Schilthuizen, V 9669); Batu Tai (not Bod Tai) near Gomantong (leg. J.J. Vermeulen & M. Schilthuizen, V 9596; leg. T.S. Liew & B. Elahan, BOR/MOL 1927); Batu Tomanggong Besar (leg. M. Schilthuizen, BOR/MOL 1125, BOR/MOL 1332; leg. T.S. Liew & B. Elahan, BOR/MOL 2264, BOR/MOL 2295); Batu Tomanggong Kecil (leg. J.J. Vermeulen & M. Schilthuizen, V 9687; leg. T.S. Liew & B. Elahan, BOR/MOL 2037, BOR/MOL 2065); Gomantong Hill 30 km South of Sandakan (leg. J.J. Vermeulen & H. Duistermaat, V 1630; leg. A. van Til, BOR/MOL 3267, BOR/MOL 3282; leg. T.S. Liew & J.P. King, BOR/MOL 3648); Hill Sg. Resang (leg. M. Schilthuizen, BOR/MOL 1335, BOR/MOL 3612); Unnamed hill near Sukau Police Station (leg. T.S. Liew & B. Elahan, BOR/MOL 2198). Segama valley, North end of limestone ridge on East bank Tabin River (leg. J.J. Vermeulen & M. Schilthuizen, V 7781). *Tawau Province*. Batu Baturong c. 50 km W.S.W. Lahad Datu (leg. J.J. Vermeulen & H. Duistermaat, V 1856); North slope (leg. J.J. Vermeulen, V 7599). Danum Valley (leg. H.A. Rutjes, BOR/MOL 1105; leg. M. Schilthuizen, BOR/MOL 1103, BOR/MOL 1110; leg. UMS students, BOR/MOL 1108; leg. T. Kimsin & H.N. Chai, BOR/MOL 1212). Gua Madai c. 40 km S.S.W. of Lahad Datu (leg. J.J. Vermeulen & H. Duistermaat, V 1722); N.E. End (leg. J.J. Vermeulen, V 7698). Segama valley, hill N.W. of crossing road Sandakan-Lahad Datu with the Segama River (leg. J.J. Vermeulen & H. Duistermaat, V 1695); ‘Kirk’s Cave’ 8 km North of Lahad Datu (leg. J.J. Vermeulen, V 1221); limestone hill on North bank Segama River, near bridge of road Sandakan to Lahad Datu (leg. J.J. Vermeulen, V 7502); Sabahmas Cave (leg. J.J. Vermeulen, V 7457, BOR/MOL 1109); Tabin Wildlife Reserve (leg. M. Schilthuizen, BOR/MOL 1106; leg. J.J. Vermeulen & M. Schilthuizen, BOR/MOL 1104). Semporna area, Bukit Tengkorak, 5 km South of Semporna (leg. M. Schilthuizen & A.S. Cabanban, V 12745, BOR/MOL 3563); Segarong Hills, Batu Tengar, 25 km E.S.E. of Kunak (leg. J.J. Vermeulen & H. Duistermaat, V 1821); Segarong Hills, Bukit Pababola, 25 km E.S.E. of Kunak (leg. J.J. Vermeulen & H. Duistermaat, V 1766). Tawau Hills N.P., path up to Bukit Bombalai (leg. J.J. Vermeulen, V 13163). *West Coast Province*. Crocker Range N.P., km 54 marker on the road Kota Kinabalu-Tambunan, Gunung Mas (leg. J.J. Vermeulen & M. Schilthuizen, V 9760).

###### Description.

Shell very small, thin, somewhat translucent, brown, lenticular; spire slightly to moderately elevated, conical with a rounded apex. Surface glossy. Whorls slightly convex. *Protoconch* with 6–10 very fine, well-spaced, continuous, shallow spiral grooves. *Teleoconch*: very fine, moderately to widely spaced, continuous, shallow, rather vaguely outlined spiral grooves on the top whorls, in adults usually absent on the last whorl above the periphery, but more frequently present, more densely placed, below the periphery. Spiral sculpture rarely entirely absent. Radial sculpture teleoconch: inconspicuous growth lines, a few irregularly spaced, very slight grooves. *Umbilicus* partly or entirely covered by a minute, semi-elliptic to triangular spur protruding from the columellar corner of the peristome; this spur often less conspicuous in juveniles. *Dimensions*: Height up to 1.9 mm; width up to 3.3 mm; diameters of the first three whorls 0.5–0.6 mm, 0.9–1.2 mm, 1.5–1.9 mm respectively; number of whorls up to 4 3/4; height aperture up to 1.3 mm; width aperture up to 1.9 mm.

###### Habitat in Sabah and distribution.

Primary and secondary forest on limestone and sandstone soil, up to 1400 m alt. **Sabah: common in East Sabah, scattered localities in West Sabah.** Distribution: Philippines (Palawan, Leyte).

###### Cross diagnosis.

The only Sabah species within group 2 of *Microcystina* with a peristome extension covering the umbilicus. Similar to *Microcystina planiuscula*, but with less rapidly expanding whorls, and with a finer spiral striation. Juveniles with an inconspicuous spur on the columellar side of the peristome can be distinguished from *Microcystina physotrochus* by the lack of radial riblets on the protoconch and by with the flat, not convex, sides of the spire.

*Microcystina cavernae* Godwin Austen, 1891, from Sarawak, has a shell of similar shape and colour; it differs in lacking the columellar spur.

##### Microcystina muscorum

Taxon classificationAnimaliaStylommatophoraAriophantidae

Van Benthem Jutting, 1959

Figure 35 , 38

Microcystina muscorum Van Benthem Jutting, 1959: 146; Clements et al. 2008: 2761–2762; Schilthuizen et al. 2013: online supplementary data. *Wilhelminaia muscorum* (Van Benthem Jutting) Maassen, 2001: 97. Type from Indonesia, Sumatra, Berastagi.
Microcystina sp. “BO-05”, Schilthuizen and Vermeulen 2003: 96; Schilthuizen 2004: 94.

###### Examined material from Sabah.

*Interior Province*. Upper Padas valley, Matang River South of Long Pasia, ridge on West bank (leg. J.J. Vermeulen, V 9823). *Kudat Province*. Balambangan Island, South end, Batu Sireh (leg. J.J. Vermeulen & M. Schilthuizen, V 9551). Banggi Island, South end, Karakit Hill (leg. J.J. Vermeulen & M. Schilthuizen, V 9465). *Sandakan Province*. Kinabatangan valley, Batu Batangan (leg. M. Schilthuizen, BOR/MOL 1338); Batu Keruak 2 near Sukau (leg. J.J. Vermeulen & M. Schilthuizen, V 12727; leg. M. Salverda & H. van Oosten, BOR/MOL 1336, BOR/MOL 3614, BOR/MOL 3616; leg. T.S. Liew & B. Elahan, BOR/MOL 1859); Batu Mawas (leg. M. Schilthuizen, BOR/MOL 1341); Batu Materis (leg. T.S. Liew & B. Elahan, BOR/MOL 3615, BOR/MOL 2128; leg. T.S. Liew & M. Schilthuizen, BOR/MOL 1972, BOR/MOL 2007); Batu Pangi (leg. J.J. Vermeulen & M. Schilthuizen, V 12731); Bod Tai (leg. M. Schilthuizen, BOR/MOL 1342); Batu Tai (not Bod Tai) near Gomantong (leg. J.J. Vermeulen & M. Schilthuizen, V 12730) (leg. T.S. Liew & B. Elahan, BOR/MOL 2643, BOR/MOL 1944); Batu Tomanggong Besar (leg. M. Schilthuizen, BOR/MOL 1350, BOR/MOL 3613); Batu Tomanggong Kecil (leg. J.J. Vermeulen & M. Schilthuizen, V 12729); Batu Tulug (Batu Putih) along road Lahad Datu-Sandakan, North of bridge over Kinabatangan River (leg. J.J. Vermeulen & H. Duistermaat, V 1498); Gomantong Hill 30 km South of Sandakan (leg. J.J. Vermeulen & H. Duistermaat, V 1633; leg. T.S. Liew & J.P. King, BOR/MOL 3650); Tandu Batu (leg. J.J. Vermeulen & M. Schilthuizen, V 9616). Segama valley, North end of limestone ridge on East bank Tabin River (leg. J.J. Vermeulen & M. Schilthuizen, V 7782); Unnamed hill near Sukau Police Station (leg. T.S. Liew & B. Elahan, BOR/MOL 2234, BOR/MOL 2199; leg. T.S. Liew, BOR/MOL 2167; leg. M. Schilthuizen, BOR/MOL 1337). *Tawau Province*. Batu Baturong c. 50 km W.S.W. Lahad Datu (leg. J.J. Vermeulen & H. Duistermaat, V 1857). Gua Madai c. 40 km S.S.W. of Lahad Datu (leg. J.J. Vermeulen & H. Duistermaat, V 1727). Segama valley, hill N.W. of crossing road Sandakan-Lahad Datu with the Segama River (leg. J.J. Vermeulen & H. Duistermaat, V 1696); ‘Kirk’s Cave’ 8 km North of Lahad Datu (leg. J.J. Vermeulen, V 14346); Sabahmas Cave (leg. J.J. Vermeulen, V 7459, V 12728, BOR/MOL 1126). Semporna, Bohey Dulang Island (leg. T.S. Liew, BOR/MOL 4607, BOR/MOL 4620, BOR/MOL 4655; leg. T.S. Liew & M. Ruf, BOR/MOL 4697, BOR/MOL 4709); Bod Gaya Island (leg. T.S. Liew, Abdul & Ladja, BOR/MOL 4749, BOR/MOL 4760, BOR/MOL 4801, BOR/MOL 4831, BOR/MOL 4856, BOR/MOL 4864, BOR/MOL 4875, BOR/MOL 4885, BOR/MOL 4896, BOR/MOL 4917); Sibuan Island (leg. T.S. Liew & Abdul, BOR/MOL 5058). *West Coast Province*. Kinabalu N.P., Poring Hot Springs, along path to waterfall (leg. J.J. Vermeulen, V 13004); Serinsim (leg. M. Schilthuizen, BOR/MOL 3078).

###### Description.

Shell very small, thin, translucent, straw yellow to brown, approx. lenticular; spire moderately elevated, conical with a rounded apex. Surface shiny. Whorls moderately convex. *Protoconch* with a fine, moderately spaced spiral striation consisting of rows of minute, rather sharply outlined pits which are arranged in a reticulate pattern towards the teleoconch; with or without patches of fine, densely placed riblets. *Teleoconch*: above the periphery with a very fine (just visible at 40 times magnification), dense and regularly spaced spiral striation, the striae consisting of rows of disconnected or partially connected, minute, rather deep, sharply outlined pits (best visible in tangential light); below the periphery the pits are sometimes more or less connected to continuous grooves. Spiral sculpture sometimes absent on the teleoconch. Radial sculpture teleoconch: inconspicuous growth lines, next to these with inconspicuous to distinct, well-spaced to densely placed, very shallow grooves, often at irregular intervals. *Umbilicus* open, narrow, columellar side of the peristome somewhat thickened but not covering the umbilicus. *Dimensions*: Height up to 1.9 mm; width up to 3.1 mm; diameters of the first three whorls 0.3–0.5 mm, 0.6–0.9 mm, 1.1–1.5 mm respectively; number of whorls up to 5 1/4; height aperture up to 1.2 mm; width aperture up to 1.5 mm.

**Figure 35–37.**
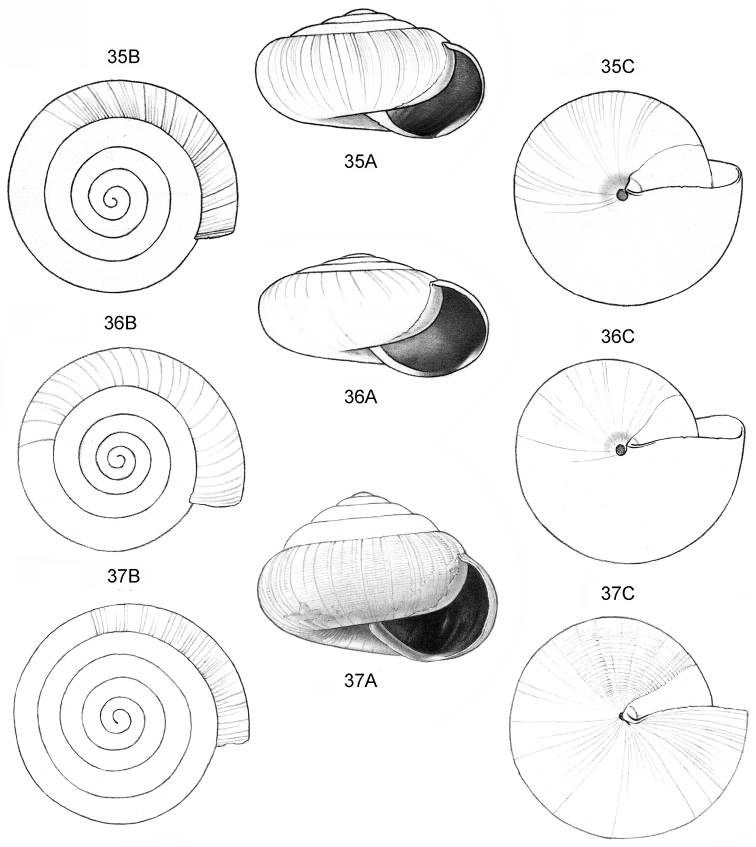
**35** *Microcystina muscorum* Van Benthem Jutting, 1959 **35A** Frontal view, shell 1.2 mm high **35B** Apical view **35C** Umbilical view (Indonesia, Bali, Danau Buyan, V 3974) **36** *Microcystina gratilla* Van Benthem Jutting, 1950. **36A** Frontal view, shell 2.0 mm high **36B** Apical view **36C** Umbilical view (Indonesia, Java, limestone mountains near Padalarang, ZMA s.n.) **37** *Microcystina physotrochus* sp. n. **37A** Frontal view, shell 2.0 mm high **37B** Apical view **37C** Umbilical view (Malaysia, Sabah, Kinabatangan valley, Batu Keruak 2 near Sukau, RMNH.5003937, holotype).

**Figure 38–39.**
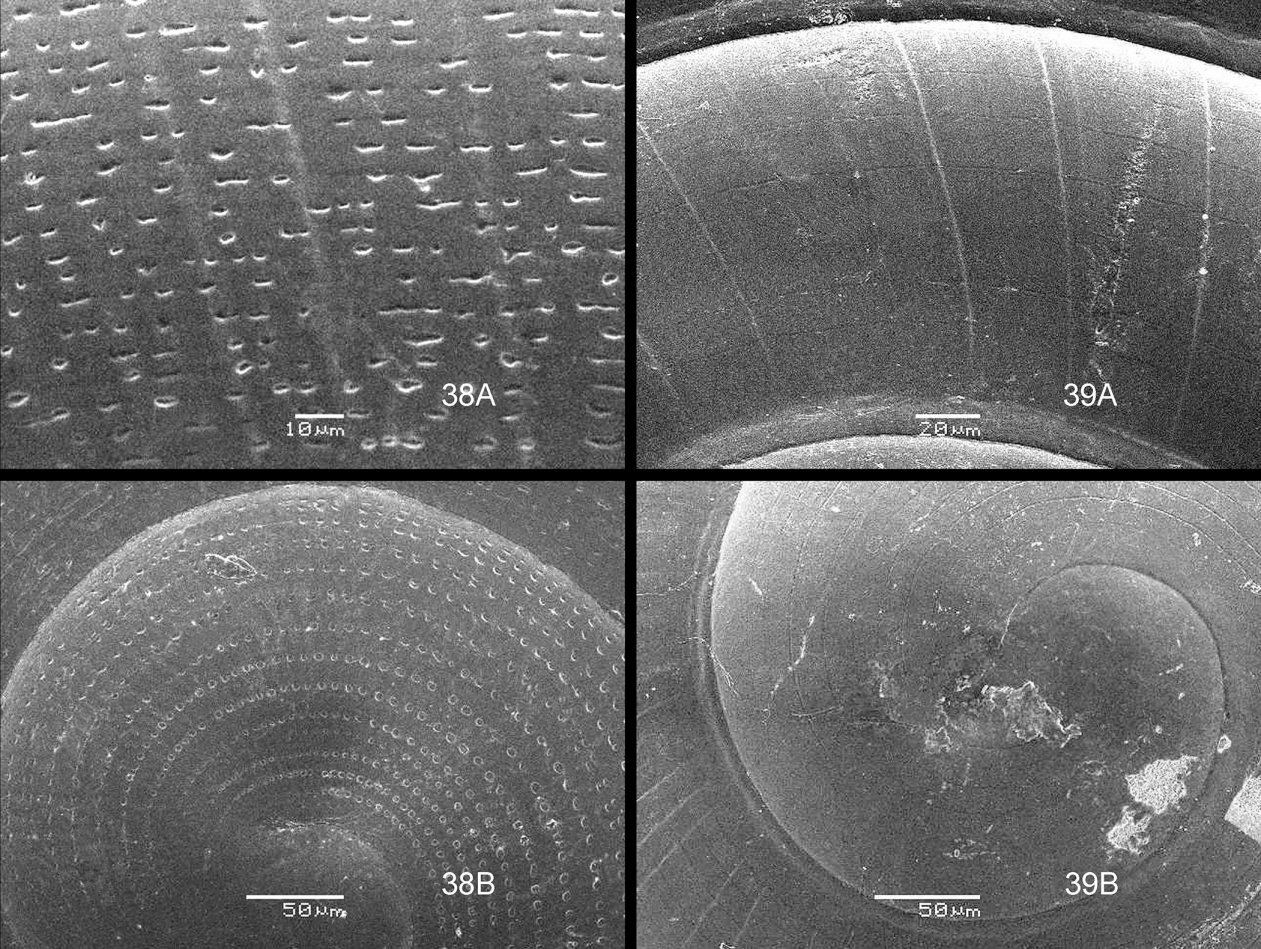
**38** *Microcystina muscorum* Van Benthem Jutting, 1959 **38A** SEM image of the third whorl **38B** SEM image of apex (Malaysia, Sabah, Kinabatangan river valley, Batu Mawas, leg. M. Schilthuizen, BOR/MOL 1341) **39** *Microcystina gratilla* Van Benthem Jutting, 1950 **39A** SEM image of the third whorl **39B** SEM image of apex (Malaysia, Sabah, Kinabalu N.P., Monggis-Tambuyukon trail between 896 and 1016 m alt., BOR/MOL 4141).

###### Habitat in Sabah and distribution.

Primary and secondary forest, coastal woodland on limestone and sandstone soil, up to 2100 m alt. **Sabah: rather common in East Sabah, only few localities in West Sabah.** Distribution: Laos; Peninsular Malaysia; Sumatra; Java; Bali.

###### Cross diagnosis.

Usually well-characterized by the spiral striation consisting of minute pits. Rare populations (one in Sabah) lack this striation on the teleoconch. On the protoconch, however, the pits are always present, which serves to distinguish *Microcystina muscorum* from *Microcystina gratilla*.

*Microcystina sinica* has a similarly pitted protoconch, see under that species.

##### Microcystina gratilla

Taxon classificationAnimaliaStylommatophoraAriophantidae

Van Benthem Jutting, 1950

Figure 36 , 39

Microcystina gratilla Van Benthem Jutting, 1950: 448; Liew et al. 2010: Appendix S1 in online Supporting Information. Type from Indonesia, Java, near Bogor. *Wilhelminaia gratilla* (Van Benthem Jutting) Maassen, 2001: 96.
Microcystina sp. “BO-04”, Schilthuizen et al. 2002a: 257; Schilthuizen et al. 2002b: 42; Schilthuizen and Vermeulen 2003: 96; Schilthuizen 2004: 94.

###### Examined material from Sabah.

*Interior Province*. Crocker Range N.P., West of the km 10 marker on the road Tambunan-Ranau, Mahua Waterfall (leg. J.J. Vermeulen, V 12741). Gunung Trusmadi (leg. M. Schilthuizen & P. Koomen, V 12740). *West Coast Province*. Kinabalu N.P., Poring Hot Springs, along path to waterfall (leg. J.J. Vermeulen, V 13006); Monggis-Tambuyukon trail between 896 and 1016 m (leg. T.S. Liew, BOR/MOL 4139, BOR/MOL 4141); Serinsim-Nombuyukon trail between 548 and 877 m (leg. T.S. Liew, BOR/MOL 4140, BOR/MOL 4143).

###### Description.

Shell very small, thin, translucent, yellowish brown to brown, lenticular; spire almost flat to moderately elevated, conical with a rounded apex. Surface glossy. Whorls moderately convex. *Protoconch*: spiral striation absent, with or without a few inconspicuous, scattered radial riblets towards the teleoconch. *Teleoconch*: with or without traces of very fine (only just visible at 40 times magnification), widely and somewhat irregularly spaced, shallow, rather sharply outlined grooves which are somewhat interrupted towards the protoconch, but which are continuous on the outer whorls. Radial sculpture teleoconch: inconspicuous growth lines, next to these inconspicuous to distinct, well-spaced to densely placed, shallow grooves, often at irregular intervals. *Umbilicus* open, narrow, columellar side of the peristome somewhat thickened but not covering the umbilicus. *Dimensions*: Height up to 2.2 mm; width up to 3.6 mm; diameters of the first three whorls 0.4–0.5 mm, 0.8–1.0 mm, 1.2–1.6 mm respectively; number of whorls up to 4 7/8; height aperture up to 1.3 mm; width aperture up to 1.3 mm.

###### Habitat in Sabah and distribution.

Primary and secondary forest on sandstone bedrock, up to 900 m alt., elsewhere up to 2400 m alt. **Sabah: Mount Kinabalu, Mount Tambuyukon; Mount Trusmadi, Crocker Range.** Also in Kalimantan. Distribution: Java; Madura, Bali; Flores.

###### Cross diagnosis.

Very similar to *Microcystina muscorum*, but the sculpture, at least on the protoconch, is different; see below *Microcystina muscorum*. Juvenile *Microcystina appendiculata* in which the spur protruding from the columellar corner of the peristome has not yet developed can be distinguished by the absence of well-spaced radial grooves on the teleoconch. Juvenile *Microcystina physotrochus* has a protoconch with more distinct radial riblets.

###### Remarks.

Most Sabah shells have a comparatively high spire.

##### Microcystina physotrochus

Taxon classificationAnimaliaStylommatophoraAriophantidae

Vermeulen, Liew & Schilthuizen sp. n.

http://zoobank.org/5F34B811-5434-4A0E-B170-648669309E55

Figure 37

Microcystina physotrochus nomen nudum, Clements et al. 2008: 2761–2762; Schilthuizen et al. 2013: online supplementary data.

###### Holotype.

Malaysia, Sabah, Sandakan Province, Kinabatangan Valley, Batu Keruak 2, near Sukau (RMNH.5003937).

###### Examined material from Sabah.

*Interior Province*. Upper Padas valley, Long Pasia (leg. T.S. Liew & Meckson, BOR/MOL 4354). Gunung Trusmadi slopes: Gua Loloposon (leg. J.J. Vermeulen, V 13243); Gua Dawaras (leg. M. Schilthuizen, V 9873). Pun Batu c. 30 km West of Sepulut (leg. J.J. Vermeulen, V 2643). Sepulut valley, Batu Punggul (leg. J.J. Vermeulen, V 1969); Bukit Tinahas, East end of Batu Punggul limestone (leg. J.J. Vermeulen & M. Schilthuizen, V 7456); Gua Pungiton (leg. J.J. Vermeulen & M. Schilthuizen, V 8082). *Sandakan Province*. Kinabatangan valley, Batu Mawas (leg. T.S. Liew & M. Schilthuizen, BOR/MOL 1971; leg. M. Schilthuizen, BOR/MOL 1345); Batu Keruak 2 near Sukau (leg. J.J. Vermeulen & M. Schilthuizen, V 12726; leg. M. Salverda & H. van Oosten, BOR/MOL 1347); Hill on Resang river (leg. M. Schilthuizen, BOR/MOL 1346). Segama valley, North end of limestone ridge on East bank Tabin River (leg. J.J. Vermeulen & M. Schilthuizen, V 7500). *Tawau Province*. Batu Baturong, North slope (leg. J.J. Vermeulen, V 7587). Danum Valley Conservation Area (leg. UMS students, V 9882; leg. M. Schilthuizen, BOR/MOL 1107). Semporna area, Segarong Hills, Batu Tengar, 25 km E.S.E. of Kunak (leg. J.J. Vermeulen & H. Duistermaat, V 2644). Tawau Hills N.P., waterfalls near Headquarters area (leg. J.J. Vermeulen, V 13203). *West Coast Province*. Kinabalu N.P., plot near Mesilau trail (leg. T.S. Liew et al.; locality 2006.S043, V 14342); Poring (leg. M. Schilthuizen & P. Koomen, BOR/MOL 1114); Sayap-Nunuhon trail between 960 and 1152 m (leg. T.S. Liew, Dominik, J. Lapidin & Jasilin, BOR/MOL 4070, BOR/MOL 4071); Monggis-Tambuyukon trail between 880 and 1144 m (leg. T.S. Liew, BOR/MOL 4072, BOR/MOL 4074, BOR/MOL 4077); Kiau-Spurs route between 2248 and 2624 m (leg. T.S. Liew, J. Lapidin & Safrie, BOR/MOL 4079, BOR/MOL 4085, BOR/MOL 4082); Mesilau trail between 2560 and 2680 m (leg. T.S. Liew, BOR/MOL 4083, BOR/MOL 4084); Serinsim-Numbuyokon trail at 1001 m (leg. T.S. Liew, BOR/MOL 4078); Summit trail between 1616 and 2526 m (leg. T.S. Liew, BOR/MOL 4075, BOR/MOL 4076, BOR/MOL 4080; leg. T.S. Liew & David, BOR/MOL 4081). Crocker Range N.P., Ulu Kimanis (leg. M. Schilthuizen, BOR/MOL 1343); Raflesia Park (leg. M. Schilthuizen, BOR/MOL 1119). Mantanani Group, Pulau Lungisan (leg. M. Schilthuizen, V 9862).

###### Description.

Shell very small, thin, slightly translucent, brown; inflated-lenticular to depressed-ovoid; spire (moderately) elevated (more distinctly elevated in some adults) conical with convex sides or depressed-ovoid, with a rounded apex. Surface glossy. Whorls moderately convex. *Protoconch*: with or without very fine (only just visible at 40 times magnification), well-spaced, somewhat interrupted, shallow, rather sharply outlined spiral grooves; also with patches of fine, densely placed radial riblets, particularly below the suture. *Teleoconch*: fine, well-spaced, shallow spiral grooves on the upper and lower surface; sometimes only present on part of the shell, or (almost) entirely absent. Radial sculpture teleoconch: inconspicuous growth lines, next to these rather distinct, well-spaced to densely placed shallow grooves, often at irregular intervals. *Umbilicus* open, narrow; columellar side of the peristome somewhat thickened but not covering the umbilicus. *Dimensions*: Height up to 2.3 mm; width up to 2.9 mm; diameters of the first three whorls 0.5–0.7 mm, 0.9–1.2 mm, 1.3–1.8 mm respectively; number of whorls up to 4 7/8; height aperture up to 1.5 mm; width aperture up to 1.6 mm.

###### Habitat in Sabah and distribution.

Rainforest, seasonally dry forest, coastal forest, secondary woodland; on limestone and sandstone bedrock, up to 2600 m alt. **Sabah: rather common. Usually found in small numbers.** Also in Sarawak. Endemic to Borneo.

###### Cross diagnosis.

Generally identified among Sabah *Microcystina* by the somewhat inflated shell. Relatively flat specimens differ from *Microcystina appendiculata* because they lack the spur on the columellar side of the peristome.

Elsewhere, the following species have a similar inflated-lenticular or depressed-ovoid shell of comparable size. *Sitala infantilis* E.A. Smith, 1895, from Palawan, has a corneous shell with a comparatively larger aperture, and a smooth surface, *Microcystina seclusa* Godwin Austen, 1891, from Sarawak, has the last whorl more narrowly rounded around the periphery. *Lamprocystis ambonica* Boettger, 1891, from the Moluccas, has a larger aperture. *Sitala amussitata* E.A. Smith, 1895, from Sarawak, and *Lamprocystis subglobosa* Von Moellendorff, 1897 (see Van Benthem Jutting 1950: 452), from Java, have the whorls are slightly laterally compressed, resulting in a somewhat shouldered last whorl.

*Philalanka anomphala* (see Endodontidae) is characterized by the thinner shell, with much coarser, raised growth lines.

###### Remarks.

Juveniles and some adults have the basal edge of the peristome more angular than in the illustrated specimen. A small callus (not protruding beyond the rim of the peristome, as in *Microcystina appendiculata*) may be present on the columellar peristome in such shells. Shells from Mount Kinabalu tend to be relatively small, with a distinctly elevated spire.

###### Etymology.

The name refers to the inflated shell shape [*phusa* (Gr.) = bellows; *trokhos* (Gr.) = wheel, a word often used for the gastropod spire].

#### Family CAMAENIDAE

Taxon classificationAnimaliaStylommatophoraCamaenidae

Pilsbry

##### Short description.

Snails. Shell often colourful, medium-sized to very large, dextral, sinistral or chirally dimorphic, (narrowly-)conical, ellipsoid, ovoid, lenticular discoidal or globose. Sculpture absent, or inconspicuous; often with minute, geometrically arranged scars, each with a periostracal hair on top. Aperture usually without teeth or lamellae, peristome usually thickened and/or reflected. Umbilicus closed or open, narrow (Family description adapted from Abbott 1989; Solem 1992).

##### Habitat and distribution.

Tree and rock dwellers. Widespread and species-rich throughout Southeast Asia, Australia, and Oceania.

##### Remarks.

Although the family is well-studied, and some groups have been confidently excluded because of polyphyly, such as the American taxa (Scott 1996; Wade et al. 2007), Solem (1992) refrains from a family diagnosis, as its relation with Bradybaenidae and other members of the helicoid clade remains unclear.

#### Genus Amphidromus

Taxon classificationAnimaliaStylommatophoraCamaenidae

Albers, 1850

Amphidromus Albers 1850: 138.

#### Amphidromus psephos

Taxon classificationAnimaliaStylommatophoraCamaenidae

Vermeulen, Liew & Schilthuizen sp. n.

http://zoobank.org/28FA32A4-0987-4D08-B869-32DB9AE85E65

Figure 40

##### Holotype.

Malaysia, Sabah, Interior Province, Pun Batu c. 30 km West of Sepulut (RMNH.5003951).

##### Examined material from Sabah.

*Interior Province*. Pun Batu c. 30 km West of Sepulut (leg. J.J. Vermeulen, V 1311).

##### Description.

Shell sinistral, rather large, rather thin but rather solid, opaque, white, top whorls with or without some oblique, pale brownish-purple markings, spire high-conical with convex sides to approx. narrowly ovoid; apex narrowly rounded. Surface shiny. Whorls: top whorls convex, other whorls slightly convex, flat or slightly concave just below the suture; last whorl rounded at the periphery, slightly convex below the periphery. *Protoconch* whorls rounded, minutely punctate. *Teleoconch*: few growth lines at irregular intervals, locally slightly raised. Spiral sculpture: a faint, very fine, dense stration locally present. Aperture slightly obliquely elliptic, peristome distinctly reflected on the palatal, basal and columellar side. *Umbilicus* closed, or rimate, a narrow pore underneath the reflected columellar peristome. *Dimensions*: Height 24–25 mm; width 11–12 mm; number of whorls 5–5 1/2; height aperture 11.0–11.5 mm; width aperture 6.5–7.5 mm.

**Figure 40–41.**
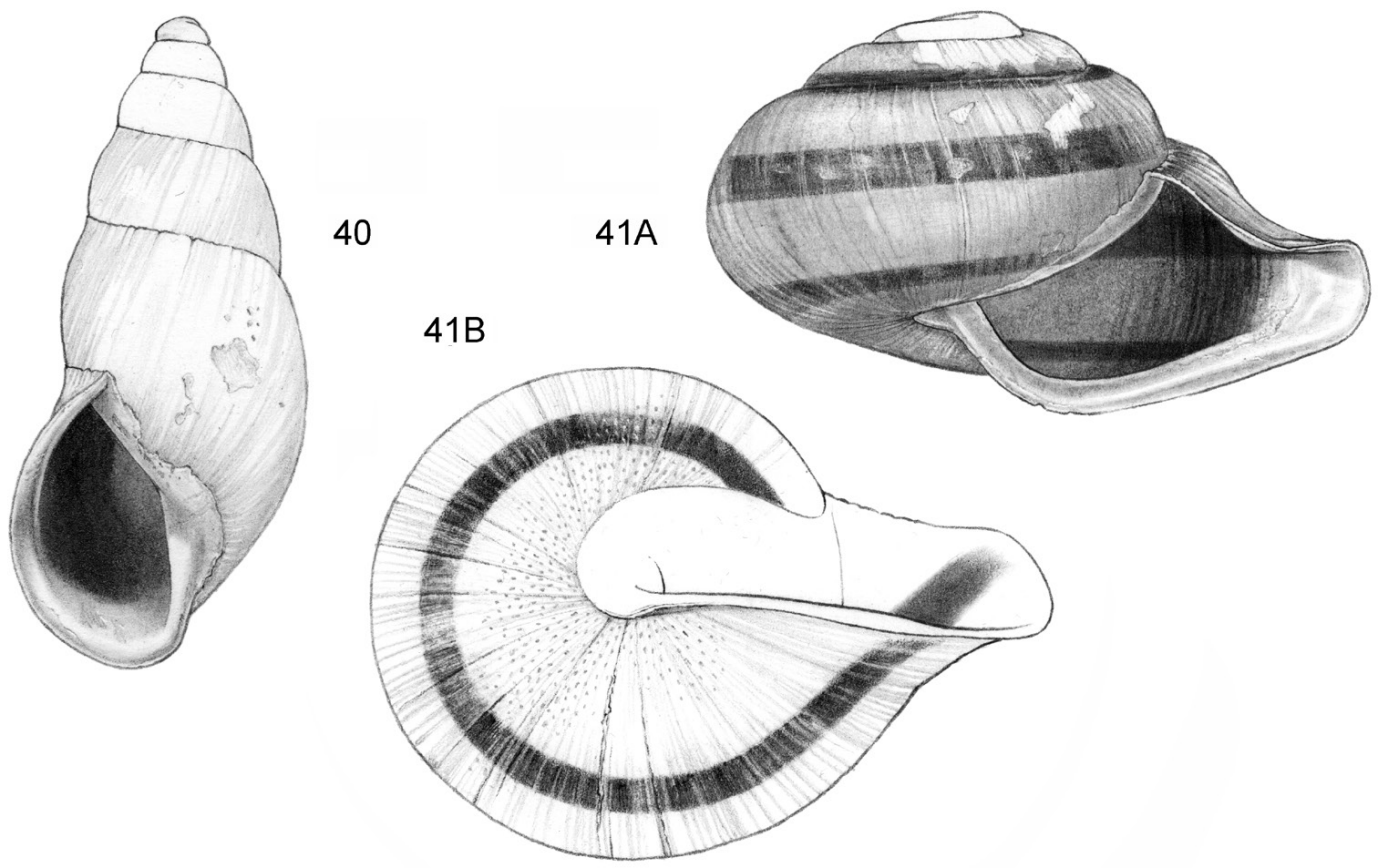
**40** *Amphidromus psephos* sp. n. Frontal view, shell 24 mm high (Malaysia, Sabah, Interior Province, Pun Batu c. 30 km West of Sepulut, RMNH.5003951, holotype) **41** *Trachia serpentinitica* sp. n. **41A** Frontal view, shell 11.5 mm high **41B** Same shell, umbilical view (Malaysia, Sabah, Sandakan Province, Gunung Meliau, South flank, BOR/MOL 3201, holotype).

##### Habitat in Sabah and distribution.

Found in shrubby forest on limestone bedrock. Alt. c. 500 m. **Sabah: Interor, Pun Batu only.** Endemic to Sabah.

##### Cross diagnosis.

The small size, combined with the narrow spire uniquely identifies this species within in the genus *Amphidromus*.

##### Etymology.

The name refers to the size of the shell [*psephos* (Gr.) = pebble].

#### Genus Trachia

Taxon classificationAnimaliaStylommatophoraCamaenidae

Albers, 1860

Trachia Albers in Albers & Von Martens, 1860: 160.

##### Remarks.

The classification of this genus is still unresolved (Solem 1964). We include the species below on account of the similarity of its shell to that of *Trachia pudica* Godwin Austen, 1891, a species native to Sabah. With its drawn-out peristome it would also fit in *Planispira* Beck, 1838, but all species included in this genus have an open umbilicus.

#### Trachia serpentinitica

Taxon classificationAnimaliaStylommatophoraCamaenidae

Vermeulen, Liew & Schilthuizen sp. n.

http://zoobank.org/0743AD7F-0D2D-4F22-9CB0-32AC923023C9

Figure 41

##### Holotype.

Malaysia, Sabah, Sandakan Province, Gunung Meliau, South flank (BOR/MOL 3201).

##### Description.

Shell dextral, medium-sized, thin, opaque, whitish with 3 rather narrow, brown bands: one slightly above the periphery, one below the suture and one basal, peristome white, spire low-conical with slightly convex sides; apex almost flat. Surface dull. Periostracum rather thick, easily peeling off in dead shells, translucent, greenish-corneous, with regularly spread, short hairs. Whorls convex and not shouldered, last whorl rounded at the periphery, convex below the periphery. *Protoconch* whorls rounded. *Teleoconch*: growth lines at irregular intervals, locally raised to inconspicuous, rather densely placed, flat riblets. No spiral sculpture. Aperture obliquely rhombiform, distinctly drawn out into a rounded beak on the paratal side, peristome only slightly reflected above the beak, distinctly so below the beak. *Umbilicus* closed, covered by an extension of the peristome. *Dimensions*: Height c. 11.5 mm; width c. 18.0 mm; diameters of the first three whorls c. 3.5 mm, c. 7.0 mm, c. 12.0 mm respectively; number of whorls c. 3 7/8; height aperture c. 7.5 mm; width aperture c. 11.5 mm.

##### Habitat in Sabah and distribution.

Found in primary forest on ultrabasic bedrock. Alt. 600–700 m. **Sabah: Gunung Meliau.** Endemic to Sabah.

##### Cross diagnosis.

Uniquely identified among Sabah Camaenidae by its beak-like extension of the palatal side of the aperture. The only Sabah species with a similar beak is the ariophantid *Rhinocochlis nasuta* (Metcalfe, 1851), which has a flat, sharply keeled, entirely white shell without hairs on the periostracum.

##### Remarks.

This species may be endemic to the Sabah areas on ultrabasic bedrock.

##### Etymology.

The name refers that the species is found on serpentinite bedrock.

#### Family ENDODONTIDAE

Taxon classificationAnimaliaStylommatophoraEndodontidae

Pilsbry

##### Short description.

Snails. Shell small to medium-sized, dextral, conical, lenticular, or discoidal. Sculpture often rather distinct, consisting of spiral striation and/or radial ribs, sculpture sometimes inconspicuous or absent. Aperture with or without teeth or lamellae, peristome neither thickened nor reflected. Umbilicus closed or open, narrow or wide (Family description adapted from Solem 1976; Schileyko 2001).

##### Habitat and distribution.

Generally found in litter and on the vegetation. Worldwide, but particularly species-rich on the islands of and around the Pacific.

##### Remarks.

The status of several genera often included in this family, including the ones we discuss here (*Philalanka* and *Thysanota*), has been debated. Raheem et al. (2014) follow Godwin-Austen (1907) in placing these two genera in the Thysanotinae and follow Bouchet and Rocroi (2005) in placing this subfamily in the Charopidae, whereas we have here retained the endodontid placement of Godwin-Austen (1907).

The endodontid genera *Philalanka* and *Thysanota* generally include small species with more or less conical shells, and often with one or more distinct spiral threads. Particularly *Philalanka* displays a wide range of shell shapes, which makes it impossible to find a diagnostic set that unequivocally distinguishes between the genera. However, we feel that including *Philalanka*, *Kaliella* and the various satellite genera into a single genus would be unjustified. This is in spite of the fact that we are in favour of genera of convenience, by necessity because in most cases our taxonomy is largely based on shells. The best we can do is to compare groups within *Philalanka* with groups within *Kaliella* which share one or two characters.

We provide a review of the Sabah species of the Endodontidae.

#### Genus Philalanka

Taxon classificationAnimaliaStylommatophoraEndodontidae

Godwin Austen, 1898

Endodonta subgenus *Philalanka* Godwin Austen, 1898: 11. *Philalanka* (Godwin Austen) Gude, 1914: 14; Naggs and Raheem 1999: 9.

##### Diagnosis for the Sabah species.

Shell conical to conical-ovoid, whorls somewhat depressed or not. Teleoconch: Radial sculpture prosocline, indistinct to coarse growth lines only, or with fine growth lines grading into rather densely placed, rather distinct riblets. Last whorl without spiral threads, or with 1–6 distinct spiral threads (next to a number of much finer spiral threads), the lowermost around the periphery, the others above the periphery and often interrupted. Umbilicus open, narrow, or closed.

##### Cross diagnosis.

Type of the genus is *Philalanka secessa* Godwin Austen, from Sri Lanka, a species comparable to *Philalanka thienemanni*, with a single peripheral thread on the last whorl. Next to these, species with more than one spiral thread (comparable to *Philalanka kusana*, below) are included by Gude (1914), next to species without any spiral sculpture (comparable to *Philalanka malimgunung*, below). Species with a single, conspicuous spiral thread and species without a spiral thread are difficult to distinguish from *Kaliella* (Helicarionidae). Such species have a wider umbilicus than *Kaliella*. *Philalanka anomphala* is the exception, with a closed umbilicus, but this has rather coarse, irregularly spaced growth lines, unlike any *Kaliella*.

##### Remarks.

On account of anatomical characters, Godwin Austen (1907[1897–1914]: 188), places the genus in the Endodontidae.

We divide the genus into two informal groups.

#### Group 1. Last whorl with 2 or more distinct spiral threads (rare specimens of *Philalanka tambunanensis* and *Philalanka kusana* have 1 spiral thread).

##### Philalanka tambunanensis

Taxon classificationAnimaliaStylommatophoraEndodontidae

Vermeulen, Liew & Schilthuizen sp. n.

http://zoobank.org/1B552A26-6D93-4C9A-A818-CECF4F1961C4

Figure 42

###### Holotype.

Malaysia, Sabah, Interior Province, Gunung Trusmadi slopes, Gua Loloposon (RMNH.5003938).

###### Examined material from Sabah.

*Interior Province*. Upper Padas valley, Long Pasia (leg. T.S. Liew & Meckson, BOR/MOL 4340). Crocker Range N.P., West of the km 10 marker on the road Tambunan-Ranau, Mahua Waterfall (leg. J.J. Vermeulen & M. Schilthuizen, V 14250; leg. M. Schilthuizen, BOR/MOL 2317). Gunung Trusmadi slopes, Gua Loloposon (leg. J.J. Vermeulen, V 13221).

###### Description.

Shell very small, thin, translucent to opaque, pale brown- or yellow-corneous to white, conical with flat or slightly convex sides; apex widely rounded. Surface with a silky luster. *Whorls* convex, rounded. *Protoconch* whorls convex; sculpture: 10–15 rather distinct, thin spiral threads; radial sculpture approx. absent or some very fine, subordinate wrinkles. *Teleoconch*: Last whorl with (1-)2 inconspicuous to distinct, narrow spiral threads (1 above the periphery, sometimes absent; 1 below the periphery, coinciding with the suture of the penultimate whorl); next to these a distinct, fine, rather dense spiral striation present, sometimes less conspicuous just below the suture; below the lowermost spiral thread the spiral striation is (somewhat) more widely spaced and gradually disappears towards the umbilicus. Radial sculpture most distinct below the suture: fine growth lines grading into rather densely placed, rather distinct riblets; the interstices between these riblets cut into the crests of the spiral striation. *Umbilicus* open, rather narrow. *Dimensions*: Height up to 2.3 mm; width up to 2.5 mm; diameters of the first four whorls 0.55–0.80 mm, 0.95–1.20 mm, 1.45–1.80 mm, 2.0–2.3 mm respectively; number of whorls up to 5; height aperture up to 1.0 mm; width aperture up to 1.3 mm.

**Figure 42–43.**
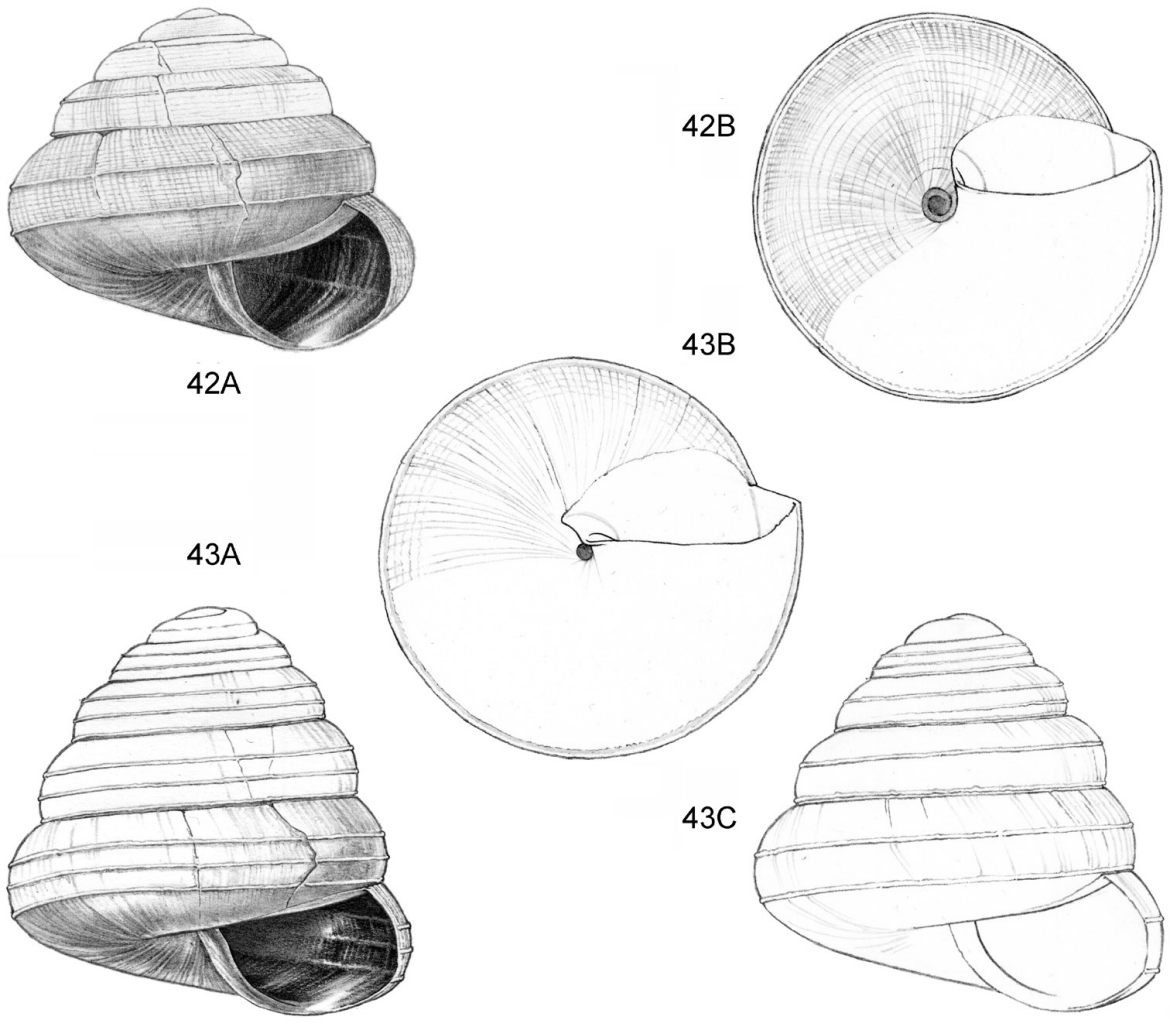
**42** *Philalanka tambunanensis* sp. n. **42A** Frontal view, shell 1.9 mm high **42B** Umbilical view (Malaysia, Sabah, Interior Province, Gunung Trusmadi slopes, Gua Loloposon, RMNH.5003938, holotype) **43** *Philalanka kusana* (Aldrich, 1889). **43A** Frontal view, shell 3.4 mm high **43B** Umbilical view of same shell **43C** Frontal view, shell 3.4 mm high (Malaysia, Sabah, Interior Province, Gunung Trusmadi slopes, Gua Loloposon, V 13223).

###### Habitat in Sabah and distribution.

Damp primary forest around streams, on limestone and sandstone bedrock, 500–1000 m alt. **Sabah: Upper Padas River, Tambunan valley.** Endemic to Sabah.

###### Cross diagnosis.

Differs from *Philalanka kusana* by the more distinct spiral striation next to the two threads, as well as a more distinct radial sculpture. As a result, the shell is not shiny, but has a silky luster. *Philalanka tambunanensis* usually has two spiral threads; *Philalanka kusana* usually has three, sometimes two.

*Philalanka carinifera* (Stoliczka), from Peninsular Malaysia, is more densely coiled (diameters of the first four whorls 0.4–0.5 mm, 0.70–0.85 mm, 1.05–1.20 mm, 1.4–1.6 mm respectively), and has a shiny surface with inconspicuous sculpture only, apart from the spiral threads.

###### Etymology.

The name refers to the area where the species is found.

##### Philalanka kusana

Taxon classificationAnimaliaStylommatophoraEndodontidae

(Aldrich, 1889)

Figure 43

Trochomorpha kusana Aldrich, 1889: 24. *Sitala kusana* (Aldrich) Godwin Austen, 1891: 40. *Philalanka kusana* (Aldrich) Schilthuizen & Rutjes, 2001: 419–420; Schilthuizen et al. 2002: 256–258; Schilthuizen et al. 2002b: 37, 41–42; Schilthuizen and Vermeulen 2003: 96; Schilthuizen 2004: 94; Clements et al. 2008: 2762; Schilthuizen et al. 2011: 5; Schilthuizen et al. 2013: online supplementary data. Type from Indonesia, S.E. Kalimantan.
Sitala quadricarinata Gude, 1917: 315. Type from Borneo.
Philalanka kusana [Not *Philalanka kusana* auct. Vermeulen & Whitten, 1998: 92, 148; = *Philalanka tjibodasensis* (Leschke)].

###### Examined material from Sabah.

*Interior Province*. Long Pa Sia (leg. Meckson & T.S. Liew, BOR/MOL 4341). Tenom (leg. M. Schilthuizen, BOR/MOL 3359). Crocker Range N.P., Gua Laing c. 12 km North of Keningau (leg. J.J. Vermeulen, V 1115; leg. M. Schilthuizen, BOR/MOL 755); West of the km 10 marker on the road Tambunan-Ranau, Mahua Waterfall (leg. J.J. Vermeulen & M. Schilthuizen, V 9730; leg. J. Schilthuizen, BOR/MOL 763; leg. M. Schilthuizen, BOR/MOL 3140). Gunung Trusmadi slopes, Gua Loloposon (leg. J.J. Vermeulen, V 13223; leg. M. Schilthuizen & P. Koomen, BOR/MOL 760; leg. M. Suleiman & M. Schilthuizen, BOR/MOL 2401). Pinangah valley, Batu Urun (= Bukit Sinobang) (leg. J.J. Vermeulen, V 1165, V 8000; leg. M. Schilthuizen, BOR/MOL 747). Pun Batu c. 30 km West of Sepulut (leg. J.J. Vermeulen, V 1281). Sepulut valley, Batu Punggul (leg. J.J. Vermeulen, V 1984; leg. T.S. Liew, BOR/MOL 4327; leg. M. Schilthuizen, BOR/MOL 745); Batu Temurung (leg. J.J. Vermeulen, V 8045, V 8046; BOR/MOL 767, BOR/MOL 768; leg. M. Schilthuizen, BOR/MOL 746); Bukit Tinagas, East end of Batu Punggul limestone (leg. J.J. Vermeulen & M. Schilthuizen, V 7630; leg. J.J. Vermeulen, BOR/MOL 753; leg. M. Schilthuizen, BOR/MOL 740); Gua Pungiton (leg. J.J. Vermeulen & M. Schilthuizen, V 7553, V 8084, BOR/MOL 749); Gua Sanaron (leg. J.J. Vermeulen & M. Schilthuizen, V 7666; leg. M. Schilthuizen, BOR/MOL 739, BOR/MOL 744). *Kudat Province*. Balambangan Island, Kok Simpul (leg. J.J. Vermeulen & M. Schilthuizen, V 9531; leg. T.H. Liew, BOR/MOL 3695); Forest near Kampung Magnin (leg. M. Schilthuizen, BOR/MOL 4359, BOR/MOL 4366). *Sandakan Province*. Kinabatangan valley, Batu Pangi (leg. J.J. Vermeulen & M. Schilthuizen, V 9646); Batu Tomanggong Kecil (leg. J.J. Vermeulen & M. Schilthuizen, V 9689; leg. T.S. Liew, BOR/MOL 2069; leg. J.J. Vermeulen, BOR/MOL 2314); Bod Tai (leg. M. Schilthuizen, BOR/MOL 2316); Batu Tulug (Batu Putih) along road Lahad Datu-Sandakan, North of bridge over Kinabatangan River (leg. J.J. Vermeulen & H. Duistermaat, V 1484); Gomantong Hill 30 km South of Sandakan (leg. J.J. Vermeulen & H. Duistermaat, V 1618; leg. A. van Til, BOR/MOL 3279; leg. A. van Til, BOR/MOL 3280, BOR/MOL 3298; leg. M. Schilthuizen, BOR/MOL 761; leg. T.S. Liew, BOR/MOL 3657). Segama Valley, North end of limestone ridge on East bank Tabin River (leg. J.J. Vermeulen & M. Schilthuizen, V 7772); Tabin (leg. M. Schilthuizen, BOR/MOL 743; leg. H.N. Chai, BOR/MOL 751; leg. J.J. Vermeulen, BOR/MOL 766). *Tawau Province*. Batu Baturong c. 50 km W.S.W. of Lahad Datu (leg. J.J. Vermeulen & H. Duistermaat, V 1862); North slope (leg. J.J. Vermeulen, V 7596). Gua Madai c. 40 km S.S.W. of Lahad Datu (leg. J.J. Vermeulen & H. Duistermaat, V 1731); N.E. end (leg. J.J. Vermeulen, V 7703). Danum Valley (leg. M. Schilthuizen, BOR/MOL 748, BOR/MOL 756, BOR/MOL 757, BOR/MOL 759; leg. J.J. Vermeulen, BOR/MOL 741; leg. H.N. Chai, BOR/MOL 754; leg. H.A. Rutjes, BOR/MOL 764; leg. N. Tawatao, BOR/MOL 2318; leg. UMS students, BOR/MOL 765). Segama valley, hill N.W. of crossing road Sandakan-Lahad Datu with the Segama River (leg. J.J. Vermeulen & H. Duistermaat, V 1673); ‘Kirk’s Cave’ 8 km North of Lahad Datu (leg. J.J. Vermeulen, V 1235); limestone hill on North bank Segama River, near bridge of road Sandakan to Lahad Datu (leg. J.J. Vermeulen, V 7509); Sabahmas Cave (leg. J.J. Vermeulen, V 7465, BOR/MOL 752). Semporna area, Segarong Hills, Bukit Pababola, 25 km E.S.E. of Kunak (leg. J.J. Vermeulen & H. Duistermaat, V 1773). Tawau Hills N.P., path up to Bukit Bombalai (leg. J.J. Vermeulen, V 13153); waterfalls near Headquarters area (leg. J.J. Vermeulen, V 13198; leg. J.P. King, BOR/MOL 2315). W*est Coast Province*. Kinabalu N.P., Poring Hot Springs, orchid Garden (leg. J.J. Vermeulen, V 13029; leg. P. Koomen, BOR/MOL 762); Serinsim-Nombuyukon trail between 458 and 542 m (leg. T.S. Liew, BOR/MOL 4214, BOR/MOL 4215). Kota Kinabalu, Kiansom Waterfall (leg. UMS students, BOR/MOL 750; leg. M. Schilthuizen, BOR/MOL 758; leg. UMS Tropical Malacology Course participants, BOR/MOL 3466).

###### Description.

Shell very small, thin, translucent to opaque, light brown to pale yellow-corneous to white, conical with slightly convex sides, sometimes conical-ovoid; apex rounded. Surface shiny. *Whorls* convex, rounded or slightly angular because of the presence of strong spiral threads, somewhat flattened below the lowermost spiral thread, sometimes somewhat flattened above the upper spiral thread. *Protoconch* whorls convex; sculpture: (1-)4–6(-9) inconspicuous to distinct, thin spiral threads; radial sculpture absent or some subordinate wrinkles. *Teleoconch*: Last whorl with (2-)3(-4) distinct, narrow spiral threads (when 2: 1 above and 1 below the periphery; when 3–4: 1 at the periphery, 1 well below, and 1–2 above), the lowermost coinciding with the suture of the penultimate whorl; between the spiral threads often (traces of) a fine, rather dense to moderately spaced spiral striation; below the lowermost spiral thread a similar moderately to widely spaced spiral striation which gradually disappears towards the umbilicus. Radial sculpture most distinct below the suture: fine growth lines, locally grading into somewhat spaced riblets. *Umbilicus* open, narrow. *Dimensions*: Height up to 3.2 mm; width up to 3.5 mm; diameters of the first four whorls 0.6–0.8 mm, 1.0–1.3 mm, 1.6–2.0 mm, 2.1–2.7 mm respectively; number of whorls up to 5 1/2; height aperture up to 1.1 mm; width aperture up to 1.6 mm.

###### Habitat in Sabah and distribution.

Primary and secondary forest, shrubby regrowth, rock outcrops, on sandstone and limestone soil, up to 600 m alt. (up to 1200 m alt. in Sarawak). **Sabah: common, widespread.** Also in Sarawak; Kalimantan. Distribution: Peninsular Malaysia; Sumatra; Eastwards to Irian Jaya.

###### Cross diagnosis.

*Philalanka tjibodasensis* (Leschke), from Java, has a comparable size and mode of coiling (diameter of the first four whorls c. 0.6 mm, 1.1 mm, 1.6 mm, 2.4 mm respectively). It differs by having flattened protoconch whorls without spiral sculpture.

*Philalanka carinifera* (Stoliczka), from Peninsular Malaysia, is of similar shape, but smaller (height up to 2 mm, and width up to 2.1 mm, at 5 3/8 whorls). It is also more densely coiled (diameter of the first four whorls 0.4–0.5 mm, 0.70–0.85 mm, 1.05–1.20 mm, 1.4–1.6 mm respectively).

###### Remarks.

Most individuals of *Philalanka kusana* have 3 spiral threads, but large samples usually include a small number of specimens with only 2 spiral threads. In a few samples from the Crocker Range, Kappes (unpublished report) suspects two discrete species within our concept of *Philalanka kusana*, based on molecular data and supported by slight differences in protoconch sculpture. We find a wide variability in protoconch sculpture, and cannot accordingly divide all material available.

Aldrich (1889) describes the shell colour as light brown. All specimens seen by us range from pale yellow-corneous to white.

##### Philalanka moluensis

Taxon classificationAnimaliaStylommatophoraEndodontidae

(E.A. Smith, 1893)

Figure 44

Sitala moluensis E.A. Smith, 1893: 343. *Philalanka moluensis* (E.A. Smith). Schilthuizen et al., 2002: 356–257; Schilthuizen and Vermeulen 2003: 96; Schilthuizen 2004: 94; Clements et al. 2008: 2762. Type from Malaysia, Sarawak, Mulu area).
Sitala inaequisculpta E.A. Smith, 1895: 112. Type from Malaysia, Sarawak, Mount Rabong).

###### Examined material from Sabah.

*Interior Province*. Upper Padas valley, Matang River South of Long Pasia (leg. J.J. Vermeulen, V 9812). Crocker Range N.P., Kalang Waterfalls (leg. J.J. Vermeulen, V 1178); Crocker Range N.P., Gua Laing c. 12 km North of Keningau (leg. J.J. Vermeulen, V 1110, V 1124); West of the km 10 marker on the road Tambunan-Ranau, Mahua Waterfall (leg. J.J. Vermeulen & M. Schilthuizen, V 9728). Gunung Trusmadi slopes, Gua Loloposon (leg. J.J. Vermeulen, V 13232). Pinangah valley, Batu Urun (= Bukit Sinobang) (leg. J.J. Vermeulen, V 8001, V 1166, V 1167, BOR/MOL 782; leg. M. Schilthuizen, BOR/MOL 773); Batu Punggul (leg. J.J. Vermeulen, V 1986; leg. M. Schilthuizen, BOR/MOL 778); Batu Temurung (leg. J.J. Vermeulen, V 8047, BOR/MOL 781; leg. M. Schilthuizen, BOR/MOL 777); Bukit Tinahas, East end of Batu Punggul limestone (leg. J.J. Vermeulen & M. Schilthuizen, V 7631); Gua Pungiton (leg. J.J. Vermeulen & M. Schilthuizen, V 7558, BOR/MOL 770); Gua Sanaron (leg. J.J. Vermeulen & M. Schilthuizen, V 7668; leg. M. Schilthuizen, BOR/MOL 3619, BOR/MOL 775). *Sandakan Province*. Kinabatangan valley, Gomantong Hill 30 km South of Sandakan (leg. J.J. Vermeulen & H. Duistermaat, V 1617). *Tawau Province*. Batu Baturong c. 50 km W.S.W. of Lahad Datu (leg. J.J. Vermeulen & H. Duistermaat, V 1858); North slope (leg. J.J. Vermeulen, V 7595). Segama valley, ‘Kirk’s Cave’ 8 km North of Lahad Datu (leg. J.J. Vermeulen, V 1219), *West Coast Province*. Kinabalu N.P., Poring Hot Springs, along path to waterfall (leg. J.J. Vermeulen, V 13009).

**Figure 44–46.**
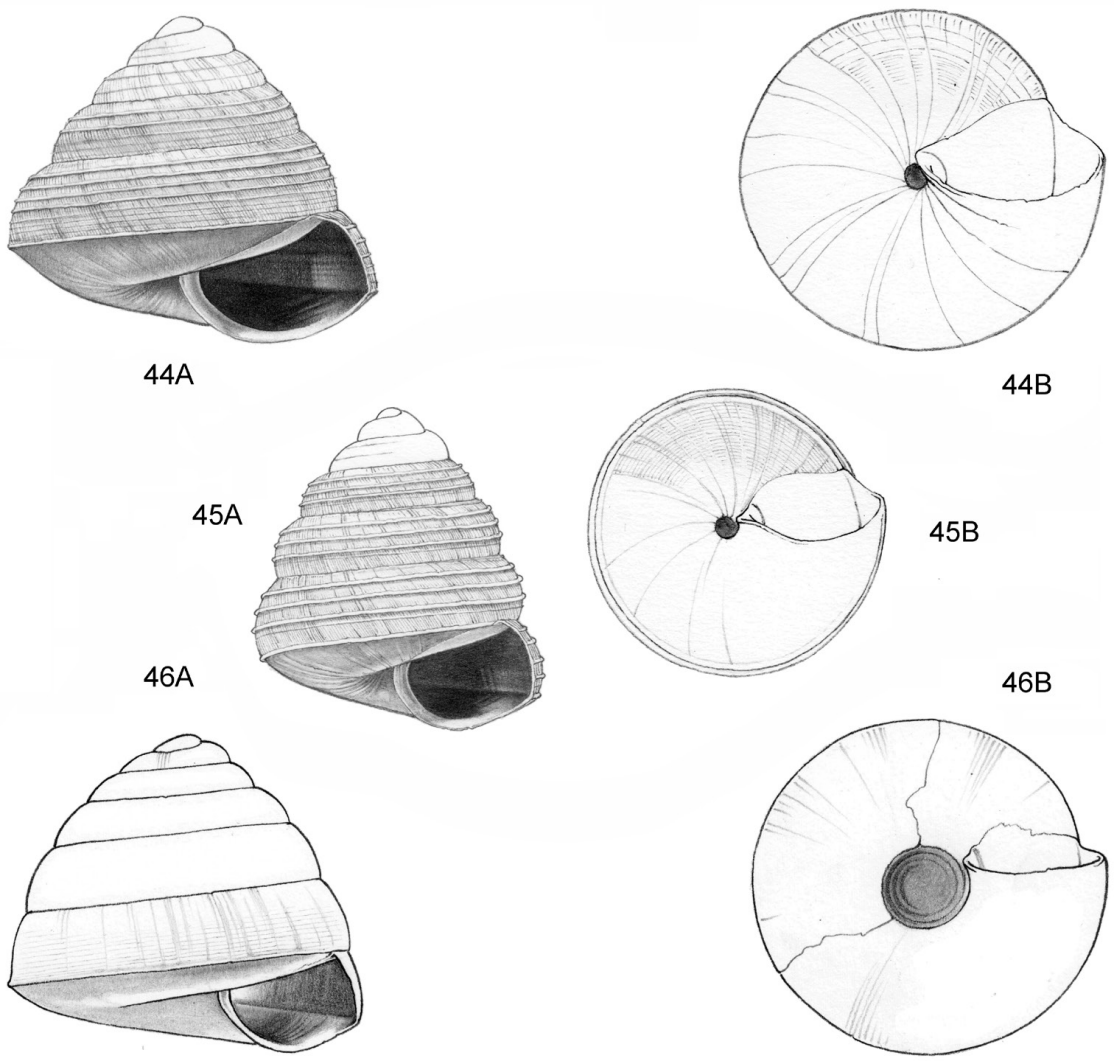
**44** *Philalanka moluensis* (E.A. Smith, 1893). **44A** Frontal view, shell 2.3 mm high **44B** Umbilical view (Malaysia, Sabah, Interior Province, Sepulut valley, Gua Pungiton, V 7558) **45** *Philalanka obscura* sp. n. **45A** Frontal view, shell 2.1 mm high **45B** Umbilical view (Indonesia, Kalimantan Timur, Sangkulirang Peninsula, Meweding Cave near village Tabalar Ulu (RMNH.5003939, holotype) **46** *Philalanka thienemanni* B. Rensch, 1932 **46A** Frontal view, shell 2.7 mm high **46B** Umbilical view (Indonesia, Java, Bremi, V 4019).

###### Description.

Shell very small, rather thin, more or less opaque, brown, very rarely yellowish, conical with approx. flat or slightly convex sides; apex rounded. Surface about dull or with a silky luster above the periphery, glossy below. *Whorls* convex, approx. rounded. *Protoconch* whorls convex; sculpture: 9–12 inconspicuous to distinct, very fine, thin spiral threads; radial sculpture absent or some subordinate wrinkles. *Teleoconch*: Last whorl with a distinct spiral thread coinciding with the suture of the penultimate whorl, above this 2–4 slightly less distinct threads with a very fine (just visible at 40 times magnification) spiral striation in between, particularly towards the periphery; below the periphery up to 4 fine spiral threads close to it, with very fine spiral striation in between. Radial sculpture: densely placed, very fine (just visible at 40 times magnification), narrow riblets that may be inconspicuous locally. *Umbilicus* open, narrow. *Dimensions*: Height up to 2.9 mm; width up to 2.8 mm; diameters of the first four whorls 0.55–0.65 mm, 0.9–1.1 mm, 1.35–1.55 mm, 1.9–2.1 mm respectively; number of whorls up to 5 7/8; height aperture up to 1.2 mm; width aperture up to 1.6 mm.

###### Habitat in Sabah and distribution.

Primary and secondary forest on limestone and sandstone soil, up to 1100 m alt. **Sabah: West part; scattered localities along East coast**. Also in Sarawak. Endemic to Borneo.

###### Cross diagnosis.

Resembles *Kaliella microconus* (Mousson), which also occurs in Sabah, but it has much coarser and less regularly spaced spiral threads, and more convex whorls.

##### Philalanka obscura

Taxon classificationAnimaliaStylommatophoraEndodontidae

Vermeulen, Liew & Schilthuizen sp. n.

http://zoobank.org/48FDA67D-59FA-4A9C-9969-1C8FFFD514D8

Figure 45

Philalanka obscura nomen nudum, Clements et al. 2008: 2762.

###### Holotype.

Indonesia, Kalimantan Timur, Sangkulirang Peninsula, Meweding Cave near village Tabalar Ulu (RMNH.5003939).

###### Examined material from Sabah.

*Interior Province*. Upper Padas valley, Long Pasia (leg. T.S. Liew & Meckson, BOR/MOL 4350). Crocker Range N.P., Gua Laing c. 12 km North of Keningau (leg. J.J. Vermeulen, V 12721; leg. M. Schilthuizen, BOR/MOL 774); Ulu Kimanis (leg. UMS students, BOR/MOL 2329). Pinangah valley, Batu Urun (= Bukit Sinobang) (leg. J.J. Vermeulen, V 12719, BOR/MOL 3605). Pun Batu c. 30 km West of Sepulut (leg. J.J. Vermeulen, V 1280). Sepulut valley, Batu Punggul (leg. J.J. Vermeulen, V 14252); Batu Temurung (leg. J.J. Vermeulen, V 12722; leg. M. Schilthuizen, BOR/MOL 771); Bukit Tinagas, East end of Batu Punggul limestone (leg. J.J. Vermeulen & M. Schilthuizen, V 12720, BOR/MOL 772; leg. M. Schilthuizen, BOR/MOL 776); Gua Pungiton (leg. J.J. Vermeulen & M. Schilthuizen, V 12724, BOR/MOL 3604); Gua Sanaron (leg. J.J. Vermeulen & M. Schilthuizen, V 12725). *Sandakan Province*. Segama Valley, North end of limestone ridge on East bank Tabin River (leg. J.J. Vermeulen & M. Schilthuizen, V 7771). *Tawau Province*. Batu Baturong, North slope (leg. J.J. Vermeulen, V 12723). Danum Valley (leg. H.A. Rutjes, BOR/MOL 769; leg. UMS students, BOR/MOL 779); Gua Madai c. 40 km S.S.W. of Lahad Datu, NE end (leg. J.J. Vermeulen, V 7704). *West Coast Province*. Kinabalu N.P., Poring Hot Springs, along path to waterfall (leg. J.J. Vermeulen, V 13010; leg. M. Schilthuizen & P. Koomen, BOR/MOL 780).

###### Description.

Shell very small, rather thin, more or less opaque, (dark) brown, sometimes yellowish, conical with approx. flat or slightly convex sides; apex rounded. Surface about dull or with a silky luster above the periphery, glossy below. *Whorls* convex, approx. rounded. *Protoconch* whorls convex; sculpture: 9–12 inconspicuous to distinct, very fine, thin spiral threads; radial sculpture absent or some subordinate wrinkles. *Teleoconch*: Last whorl with a distinct spiral thread coinciding with the suture of the penultimate whorl, above this 3–4 about equally distinct threads, and in between up to 2 less distinct ones; below the lowermost thread a very fine spiral striation close to it, sometimes with up to 4 slightly coarser threads interspersed. Radial sculpture: densely placed, very fine (just visible at 40 times magnification), narrow riblets that may be inconspicuous locally. *Umbilicus* open, narrow. *Dimensions*: Height up to 2.7 mm; width up to 2.4 mm; diameters of the first four whorls 0.5–0.65 mm, 0.85–1.00 mm, 1.15–1.35 mm, 1.5–1.8 mm respectively; number of whorls up to 5 3/4; height aperture up to 0.9 mm; width aperture up to 1.3 mm.

###### Habitat in Sabah and distribution.

Primary and secondary forest on limestone and sandstone soil, up to 500 m alt. **Sabah: scattered localities**. Also in Kalimantan. Endemic to Borneo.

###### Cross diagnosis.

Differs from *Philalanka moluensis* by not having a fine spiral striation in between the spiral threads above the periphery, except for a few subordinate threads. Next to this, the shells are usually higher conical, and the spiral threads are thicker.

###### Etymology.

The name refers to the shell colour [*obscurus* (L.) = dark].

#### Group 2. Last whorl with 1 spiral thread, or without a spiral thread

##### Philalanka thienemanni

Taxon classificationAnimaliaStylommatophoraEndodontidae

B. Rensch, 1932

Fig. 46

Philalanka thienemanni B. Rensch, 1932: 105; Schilthuizen 2004: 94. Type from Indonesia, Java.

###### Examined material from Sabah.

*Interior Province*. Crocker Range N.P., West of the km 10 marker on the road Tambunan-Ranau, Mahua Waterfall (leg. J.J. Vermeulen & M. Schilthuizen, V 14251). *West Coast Province*. Crocker Range N.P., 22.5 km road Tambunan to Kota Kinabalu, near Rafflesia Park (leg. M. Schilthuizen, V 13530).

###### Description.

Shell very small, very thin, translucent, pale yellow-corneous to white, conical with convex sides, to almost conical-ovoid; apex rounded. Surface glossy. *Whorls* moderately convex, last whorl rounded or slightly angular because of the presence of a strong spiral thread. *Protoconch* whorls convex, smooth or with 1–6 very inconspicuous, thin spiral threads. *Teleoconch*: last whorl with a distinct, thick peripheral thread coinciding with the suture of the penultimate whorl; next to this thread (traces of) very fine, inconspicuous, well-spaced, very thin spiral threads present except in the umbilical region; with or without (traces of) an even finer (just visible at 40 times magnification), dense spiral striation in between these threads, especially above the peripheral thread. Radial sculpture: locally a few inconspicuous, well-spaced growth lines, locally also very fine (barely visible at 40 times magnification), densely placed riblets. *Umbilicus* open, wide. *Dimensions*: Height up to 2.35 mm; width up to 2.6 mm; diameters of the first four whorls 0.5–0.6 mm, 0.90–1.05 mm, 1.3–1.6 mm, 1.80–2.15 mm respectively; number of whorls 6; height aperture up to 0.8 mm; width aperture up to 1 mm.

###### Habitat in Sabah and distribution.

Found in damp forest on sandstone soil, at 500–1700 m alt. **Sabah**: **Crocker Range**. Distribution: Thailand; Java; Bali; Flores.

###### Remarks.

Shells from Thailand (V5603, from Chiang Mai) have a slightly more distinct spiral sculpture, particularly on the lower surface, than shells from Java. The Borneo shells are intermediate in this respect.

##### Philalanka anomphala

Taxon classificationAnimaliaStylommatophoraEndodontidae

Vermeulen, Liew & Schilthuizen sp. n.

http://zoobank.org/CCCAE19A-42F7-4895-A132-DBA3943968C1

Figure 47

###### Holotype.

Malaysia, Sabah, West Coast Province, Kinabalu N.P., Mesilau trail (RMNH.5003940).

###### Examined material from Sabah.

*West Coast Province*. Kinabalu N.P., near Kotal route at 2376 m (leg. T.S. Liew, J. Lapidin, Safrie & Jasilin, BOR/MOL 4202, V 14335); Mesilau trail between 2112 and 2356 m (leg. T.S. Liew, BOR/MOL 4204, BOR/MOL 4206, V 14334; leg. T.S. Liew & J. Lapidin, BOR/MOL 4205); Summit trail at 2308 m (leg. T.S. Liew, BOR/MOL 4203).

###### Description.

Shell very small, thin, hardly translucent, yellow-corneous, low-conical with slightly convex sides; apex somewhat flattened. Surface glossy. *Whorls* convex, rounded. *Protoconch* whorls convex; surface almost smooth, the slightest traces of a very fine (just visible at 40 times magnification), dense spiral striation, as well as some traces of radial sculpture. *Teleoconch*: Last whorl with a thin, inconspicuous (conspicuous in juveniles) spiral thread following the periphery and coinciding with the suture of the penultimate whorl; above this traces of very fine (just visible at 40 times magnification), rather dense spiral striation, below the periphery with a similar, but widely spaced, striation; this spiral striation subordinate to rather coarse, moderately spaced but irregularly placed, usually somewhat sunk growth lines above the periphery, radial sculpture below the periphery less conspicuous. *Umbilicus* closed, rimate in juveniles. *Dimensions*: Height up to c. 1.75 mm; width up to c. 2.3 mm; diameters of the first three whorls c. 0.75 mm, c. 1.25 mm, c. 1.9 mm respectively; number of whorls up to c. 3 5/8; height aperture up to 1.0 mm; width aperture up to 1.2 mm.

**Figure 47–49.**
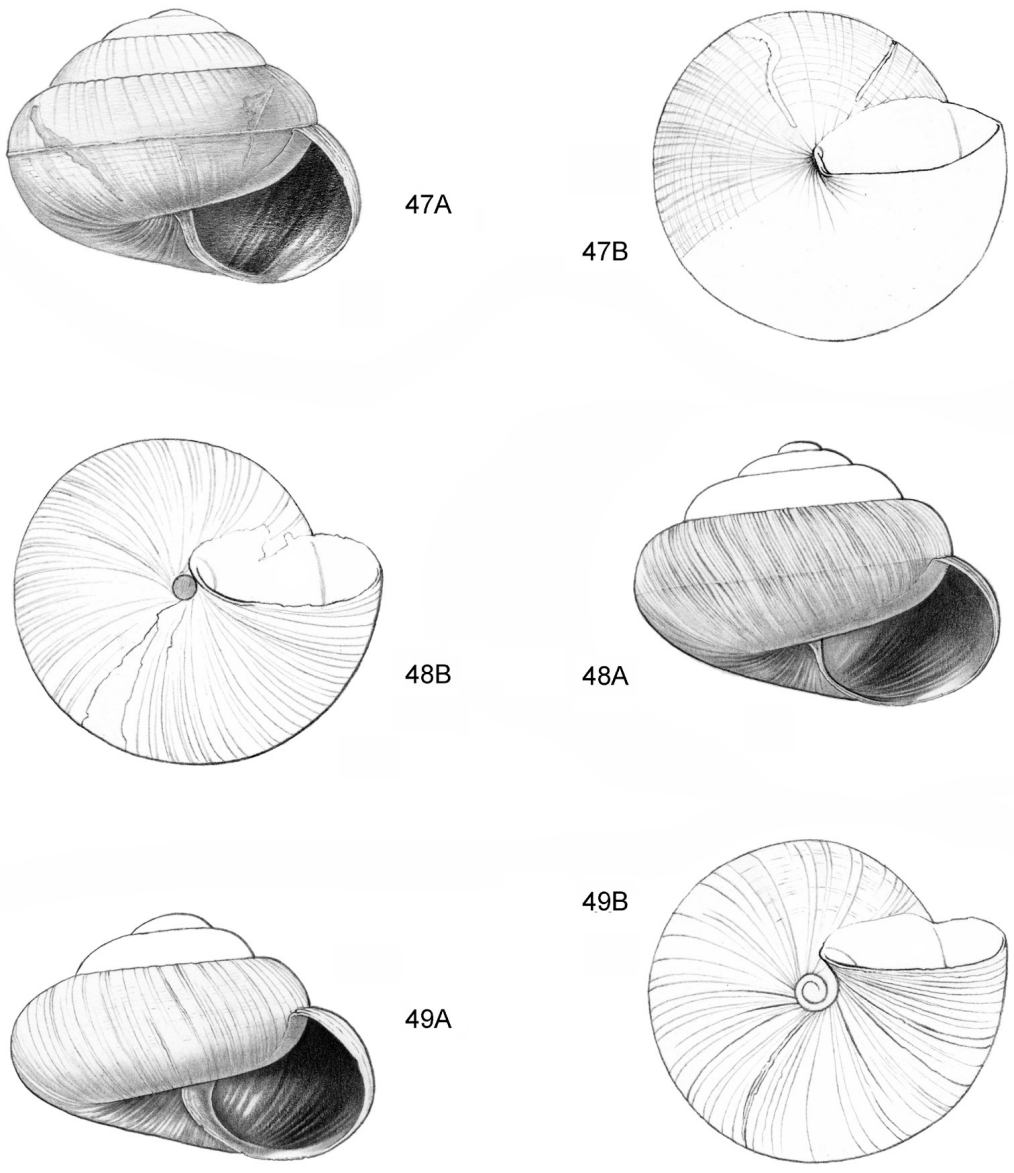
**47** *Philalanka anomphala* sp. n. **47A** Frontal view **47B** Umbilical view (Malaysia, Sabah, West Coast Province, Kinabalu N.P., Mesilau trail, RMNH.5003940, holotype, aperture reconstructed) **48** *Philalanka rugulosa* sp. n. **48A** Frontal view, shell 2.7 mm high **48B** Umbilical view (Malaysia, Sabah, West Coast Province, Kinabalu N.P., summit trail, RMNH.5003941, holotype, shell partly reconstructed) **49** *Philalanka malimgunung* sp. n. **49A** Frontal view, shell 1.4 mm high **49B** Umbilical view (Malaysia, Sabah, West Coast Province, Kinabalu N.P., summit trail, RMNH.5003942, holotype).

###### Habitat in Sabah and distribution.

Montane forest on sandstone soil. Alt. 2100–2400 m. **Sabah: Mount Kinabalu**. Endemic to Sabah.

###### Cross diagnosis.

Differs from *Philalanka rugulosa* and *Philalanka malimgunung*, as well as from the similar looking species *Philalanka lieftincki* Van Benthem Jutting (Indonesia, Ambon), and *Philalanka micromphala* Van Benthem Jutting (Indonesia, Java), by having a closed umbilicus.

###### Etymology.

The name refers to the absence of an umbilicus [*an-omphalos* (Gr.) = without umbilicus].

##### Philalanka rugulosa

Taxon classificationAnimaliaStylommatophoraEndodontidae

Vermeulen, Liew & Schilthuizen sp. n.

http://zoobank.org/3878A617-9773-4C10-8B88-0A4084027838

Figure 48

###### Holotype.

Malaysia, Sabah, West Coast Province, Kinabalu N.P., summit trail (RMNH.5003941).

###### Examined material from Sabah.

*West Coast Province*. Kinabalu N.P., Summit trail between 2984 and 4080 m (leg. T.S. Liew, BOR/MOL 4197, BOR/MOL 4198, BOR/MOL 4199, BOR/MOL 4200, BOR/MOL 4201, V 14339, V 14340, V 14341).

###### Description.

Shell very small, very thin, hardly translucent, yellowish brown, (low) conical with slightly convex sides; apex rounded. Surface with a silky luster. *Whorls* convex above and below the periphery, periphery rounded to slightly angular (in adults, more distinctly angular in juveniles, suture somewhat impressed. *Protoconch* whorls moderately convex; almost smooth with the slightest traces of a radial sculpture, and a very fine, dense spiral striation. *Teleoconch*: last whorl sometimes with a thin, inconspicuous peripheral thread coinciding with the suture of the penultimate whorl; some traces of a fine, dense spiral striation below this thread. Radial sculpture: rather distinct, irregularly spaced, somewhat raised growth lines, grading into more regularly and rather densely placed, low riblets; towards the umbilicus the growth lines tend to become less conspicuous. *Umbilicus* open, narrow. *Dimensions*: Height up to 2.7 mm; width up to 3.4 mm; diameters of the first three whorls 0.65–0.75 mm, 1.2–1.4 mm, 2.25–2.60 mm respectively; number of whorls up to c. 4 1/4; height aperture up to 1.5 mm; width aperture up to 1.75 mm.

###### Habitat in Sabah and distribution.

Subalpine vegetation on granodiorite soil. Alt. 2900–4100 m. **Sabah: Mount Kinabalu (summit trail area).** Endemic to Sabah.

###### Cross diagnosis.

Distinctly larger than *Philalanka anomphala* and *Philalanka malimgunung*; from the first it also differs by having an umbilicate shell.

*Philalanka lieftincki* Van Benthem Jutting, 1953 (Indonesia, Ambon) looks similar but is smaller (shell 1.5–1.9 mm high, at 4–4 1/2 whorls). *Philalanka micromphala* Van Benthem Jutting, 1952 (Indonesia, Java) is of similar shape and size, but has a more angular last whorl with a distinct peripheral thread, a finer radial sculpture and a more depressed aperture (aperture 1.1–1.2 mm high, at a shell height of 2.1–2.7 mm).

*Kaliella scandens* and *Kaliella doliolum* are similar in shape but have a far more regular radial sculpture, consisting of densely placed ribs.

###### Etymology.

The name refers to the shell surface [*rugulosus* (L.) = finely wrinkled].

##### Philalanka malimgunung

Taxon classificationAnimaliaStylommatophoraEndodontidae

Vermeulen, Liew & Schilthuizen sp. n.

http://zoobank.org/33E06180-04E1-4A2B-8C80-41DB4216F799

Figure 49

###### Holotype.

Malaysia, Sabah, West Coast Province, Kinabalu N.P., summit trail (RMNH.5003942).

###### Examined material from Sabah.

*West Coast Province*. Kinabalu N.P., Summit trail between 3119 and 4080 m (leg. T.S. Liew, BOR/MOL 4192, BOR/MOL 4193, BOR/MOL 4195, BOR/MOL 4196,V 14336, V 14337, V 14338).

###### Description.

Shell minute, very thin, hardly translucent, pale yellow-brown, yellow-corneous below, (low-)conical with approx. flat sides; apex rounded. Surface with a silky luster. *Whorls* convex, rounded (angular in juveniles), suture somewhat impressed. *Protoconch* whorls moderately convex; with inconspicuous, very fine (just visible at 40 times magnification) and dense spiral striation. *Teleoconch* with very fine (just visible at 40 times magnification), dense spiral striation above the periphery, which is continuous on the top whorls but more patchy on the last whorl; with a similar but somewhat more widely spaced striation below the periphery; spiral striation somewhat subordinate to the rather distinct, irregularly spaced, slightly raised growth lines, which are less conspicuous towards the umbilicus. *Umbilicus* open, rather narrow. *Dimensions*: Height up to 1.4 mm; width up to 1.9 mm; diameters of the first three whorls 0.45–0.50 mm, 0.85–1.00 mm, 1.40–1.55 mm respectively; number of whorls up to c. 3 1/2; height aperture up to 0.8 mm; width aperture up to 0.9 mm.

###### Habitat in Sabah and distribution.

Subalpine vegetation on granodiorite soil. Alt. 3200–4100 m. **Sabah: Mount Kinabalu (summit trail area).** Endemic to Sabah.

###### Cross diagnosis.

Most similar in size and shape to *Philalanka anomphala*, it differs by having an open umbilicus, and by lacking the peripheral thread. It also differs from the extralimital species listed under *Philalanka rugulosa* by the lack of a peripheral thread.

###### Etymology.

The species is named in recognition of generations of mountain guides (‘*Malim Gunung*’) who assist tourists and scientists on their way to the peaks of Mount Kinabalu every day, and who have aided scientific discoveries on this mountain since the first documented exploration of Mount Kinabalu in 1851. The species name also serves to honour the guides who lost their lives protecting mountain climbers when Mount Kinabalu was struck by earthquake on June 5^th^, 2015.

#### Genus Thysanota

Taxon classificationAnimaliaStylommatophoraEndodontidae

Albers 1860

Nanina section *Thysanota* Albers in Albers & Von Martens, 1860: 63. *Thysanota* (Albers) Godwin Austen, 1907 (1897–1914): 189; Gude 1914: 10; Naggs and Raheem 1999: 23.
Queridomus Iredale, 1937: 322; Solem 1988: 552).

##### Diagnosis for the Sabah species.

Shell conical, with almost flat sides, with depressed whorls. Teleoconch: Radial sculpture prosocline, consisting of densely placed to moderately spaced, fine riblets. Last whorl with 2–3 distinct spiral threads (next to a number of much finer spiral threads), the lowermost around the periphery, the others above the periphery and often interrupted. Umbilicus open, narrow, or closed.

##### Cross diagnosis.

The Sabah species of *Thysanota* differ from *Philalanka* by the combination of a conical spire, with almost flat sides, somewhat depressed whorls and a very narrow, or closed umbilicus.

##### Remarks.

The generic position of the two species listed below is unresolved. Solem (1988: 552) places *Thysanota grenvillei* in the Helicarionidae, although he finds its anatomy different from other Australian helicarionid genera. Godwin Austen (1907 [1897–1914]: 189) dissects an Indian species, *Thysanota carinigera* (Benson), and places the genus in the Endodontidae. Solem also expresses the possibility that *Thysanota conula* (as *Liardetia fimbriosa*), and *Thysanota grenvillei* belong to the same genus. With several more samples of both species at hand from widely scattered localities in S. and S.E. Asia, we conclude that their shells, at least, show striking similarity. The genus *Thysanota* includes more species with similar shells (see Gude 1914: 10; Naggs and Raheem 1999: 23); we feel that the species below are best placed in this genus.

#### Thysanota conula

Taxon classificationAnimaliaStylommatophoraEndodontidae

(Blanford, 1865)

Figure 50

Nanina conula Blanford, 1865: 73. *Helix conulus* (Blanford) Pfeiffer, 1868: 89. *Kaliella conulus* (Blanford) Godwin Austen, 1883 (1882–1888): 71. *Queridomus conulus* (Blanford) Clements et al. 2008: 2762. Type from India, Arakan.
Sitala fimbriosa Quadras & Von Moellendorff, 1894: 89. *Kaliella fimbriosa* (Quadras & Von Moellendorff) Rensch, 1932: 67. *Sitalinopsis fimbriosa* (Quadras & Von Moellendorff) Rensch, 1935: 332. *Liardetia fimbriosa* (Quadras & Von Moellendorff) Van Benthem Jutting, 1950: 410. *Queridomus fimbriosus* (Quadras & Von Moellendorff) Vermeulen & Whitten, 1998: 104. Type from Philippines, Negros.
Sitala elatior Bavay & Dautzenberg, 1908: 232. Type from Vietnam, Phu-Quoc-Oai.
Thysanota elegans Preston, 1909: 135); Naggs and Raheem 2000: iii, 23. Type from Sri Lanka.

##### Examined material from Sabah.

*Interior Province*. Sepulut valley, Batu Temurung (leg. J.J. Vermeulen, V 8044, BOR/MOL 886; leg. M. Schilthuizen, BOR/MOL 885).

##### Description.

Shell very small, rather thin, somewhat translucent or opaque, pale brown to white, high-conical with approx. flat sides; apex rounded. Surface about dull or with a silky luster above the periphery, shiny below. *Whorls* moderately convex, (moderately) angular at the periphery, above the periphery slightly rounded or almost flat, below the periphery with a second, moderate angle at a distance from the periphery, below this almost flat. *Protoconch* whorls convex, with 10–15 distinct, fine, rather densely placed, thin spiral threads; radial sculpture absent or subordinate. *Teleoconch*: Last whorl with a distinct peripheral spiral thread, below the periphery with a second, equally distinct spiral thread at a distance from the periphery, and coinciding with the suture of the penultimate whorl; next to these two with numerous much finer, well-spaced spiral threads, which become finer and more widely spaced towards the suture and towards the umbilicus. Radial sculpture: growth lines above the periphery and particularly below the suture grading into rather densely placed to moderately spaced, fine, narrow riblets at more or less regular distances. which may form slight nodes where they cross the spiral threads. *Umbilicus* closed, or open, narrow. *Dimensions*: Height up to 2.3 mm; width up to 1.6 mm; diameters of the first four whorls 0.40–0.45 mm, 0.60–0.65 mm, 0.80–0.95 mm, 1.05–1.30 mm respectively; number of whorls up to 6 1/2; height aperture up to 0.5 mm; width aperture up to 0.9 mm.

**Figure 50–51.**
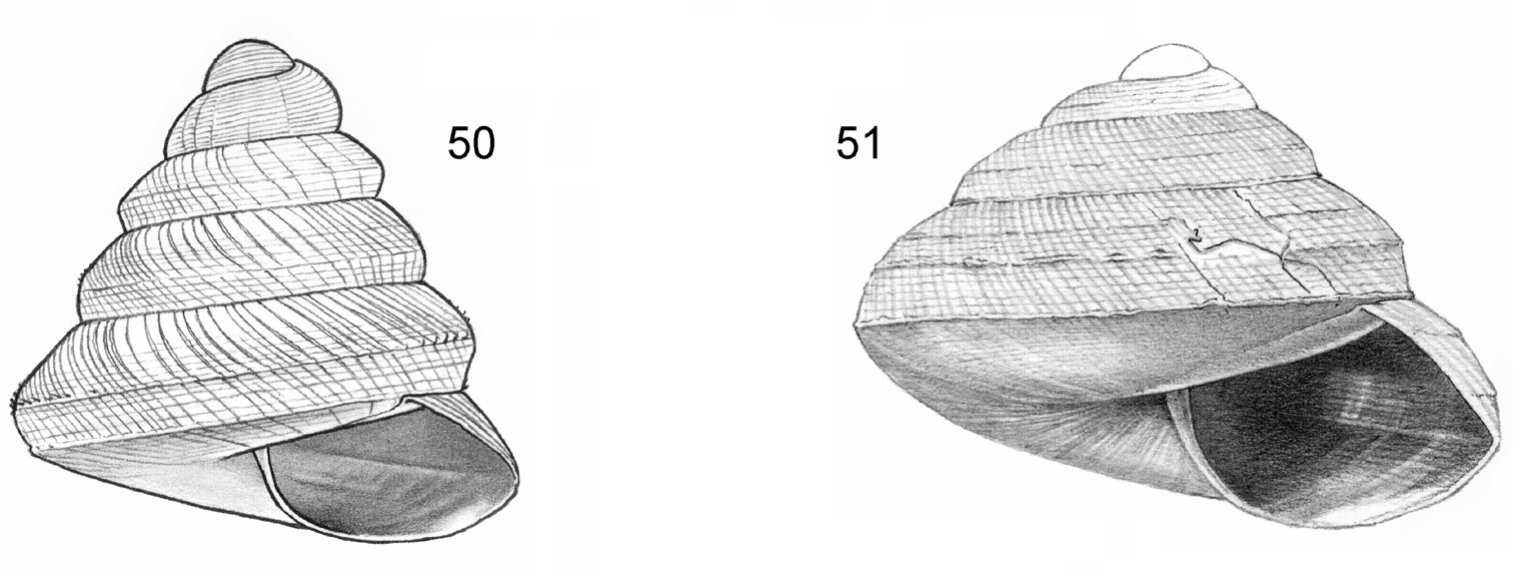
**50** *Thysanota conula* (Blanford, 1865). Frontal view, shell 1.5 mm high (Indonesia, Nusa Penida, Tengasa, V 4086) **51** *Thysanota grenvillei* (Brazier, 1876). Frontal view, shell 1.9 mm high (Vietnam, Quang Ninh Province, Ha Long Bay, Dao Bo Hon, 300 m N.W. Song Sot Cave, V 6935).

##### Habitat in Sabah and distribution.

Found near a limestone cliff in logged forest, at 400–500 m alt. Elsewhere in a range of disturbed environments, drought tolerant. **Sabah: Interior, Sepulut valley.** Distribution elsewhere: India; Vietnam; Peninsular Malaysia; Southwards to Java; Eastwards to Halmahera.

##### Remarks.

The relative width of the shell shows some variability: some shells are slightly higher than wide, others are distinctly higher than wide.

#### Thysanota grenvillei

Taxon classificationAnimaliaStylommatophoraEndodontidae

(Brazier, 1876)

Figure 51

Helix grenvillei Brazier, 1876: 104. *Queridomus grenvillei* (Brazier) Solem, 1988: 552. Type from Australia, Queensland, Cape York Peninsula.

##### Examined material from Sabah.

*Tawau Province*. Gua Madai c. 40 km S.S.W. of Lahad Datu (leg. J.J. Vermeulen & H. Duistermaat, V 1730). Semporna area, Segarong Hills, Batu Tengar, 25 km E.S.E. of Kunak (leg. J.J. Vermeulen & H. Duistermaat, V 1789); Bukit Pababola, 25 km E.S.E. of Kunak (leg. J.J. Vermeulen & H. Duistermaat, V 1775).

##### Description.

Shell very small, rather thin, somewhat translucent or opaque, (pale) brown, somewhat depressed conical with approx. flat sides; apex rounded. Surface about dull or with a silky luster above the periphery, shiny below. *Whorls* moderately convex, (moderately) angular at the periphery, above the periphery rounded or with a second, slight angle at a distance from the periphery, below the periphery rounded. *Protoconch* whorls convex, with 7–10 rather distinct, very fine, well-spaced, thin spiral threads; radial sculpture absent. *Teleoconch*: Last whorl with a distinct peripheral spiral thread coinciding with the suture of the penultimate whorl, above this 1(-2) less distinct threads as well as numerous very fine spiral threads, the latter more widely spaced towards the upper suture; below the peripheral thread a number of finer threads close to it, as well as a fine, continuous, moderately spaced striation over most of the lower surface. Radial sculpture: growth lines, above the periphery grading into densely placed, rather regularly spaced, fine, narrow riblets, which form minute nodes where they cross the spiral threads. *Umbilicus* open, narrow. *Dimensions*: Height up to 2.0 mm; width up to 2.5 mm; diameters of the first four whorls 0.4–0.5 mm, 0.70–0.95 mm, 1.15–1.50 mm, 1.85–2.40 mm respectively; number of whorls up to 4 5/8; height aperture up to 0.85 mm; width aperture up to 1.25 mm.

##### Habitat in Sabah and distribution.

Found on limestone hills near the coast. Elsewhere also found in coastal environments. **Sabah**: **Gunung Madai, Segarong Hills E.N.E. of Semporna.** Distribution elsewhere: Vietnam; Australia.

##### Cross diagnosis.

More depressed conical than *Thysanota conula*, with the shell wider than high.

##### Remarks.

The Borneo material is virtually identical with the Australian shell shown in Solem (1988: 552), and with material collected by the first author in Ha Long Bay, northern Vietnam. Australian shells have periostracal hairs where the more distinct spiral threads cross the radial ribs. In some Vietnam shells these hairs are fused to low, erose periostracal crests on the spiral threads. In the Borneo shells, all periostracum has worn off.

The species is now known from three widely distant coastal areas. It is probably a widespread element of the coastal fauna, but appears very rare. It is not an Australian endemic species, as was assumed so far.

#### Family EUCONULIDAE

Taxon classificationAnimaliaStylommatophoraEuconulidae

Baker

##### Short description.

Snails. Shell (very) small, dextral, conical, ovoid or sometimes lenticular. Sculpture inconspicuous, consisting of very fine spiral striation and/or radial riblets. Aperture without teeth or lamellae, peristome neither thickened nor reflected. Umbilicus closed, or covered by a thin callus extending from the peristome, or open, narrow (Family description adapted from Baker 1941, Solem 1966).

##### Habitat and distribution.

Generally dwelling in litter and low vegetation. Worldwide.

#### Genus Kaliella

Taxon classificationAnimaliaStylommatophoraEuconulidae

Blanford, 1863

Kaliella Blanford, 1863: 83.

##### Diagnosis for the Sabah species.

Teleoconch: Radial sculpture prosocline, consisting of fine growth lines or densely placed to moderately spaced, fine riblets. Last whorl without prominent spiral threads (numerous very fine spiral threads may be present), or with 1 spiral thread or keel at the periphery.

##### Remarks.

The inclusion of the species listed below in *Kaliella* is merely a convenience, as is the inclusion of our concept of *Kaliella* in the Euconulidae. Both are inspired by an overall similarity of the shells. It is certain that our arrangement does not reflect phylogenetic relationships between the species. In fact, anatomical analysis of 5 Sabah species (Tillier and Bouchet 1988) reveals major differences between them, and consequently they are accommodated in two new genera *Gunongia* and *Kionghutania*. These genera are characterized by properties of the shell, radula and anatomy. We prefer not to apply the four generic names mentioned. Approximately half of the species listed below are only known by their shells, and we cannot unequivocally extend the division into four different genera over all the Sabah species. Until more phylogenetic details become apparent, we prefer to re-instate the old genus *Kaliella*. The genus *Kaliella* is typified by a species occurring in Sabah: *Kaliella barrakporensis* (Pfeiffer).

We provide a review of the Sabah species of *Kaliella*. We divide the genus into four informal groups.

#### Group 1. Protoconch with spaced spiral threads which are predominant over the radial sculpture

##### Kaliella microconus

Taxon classificationAnimaliaStylommatophoraEuconulidae

(Mousson, 1865)

Figure 52 , 54

Nanina microconus Mousson, 1865: 192. *Coneuplecta microconus* (Mousson) H. Burrington Baker, 1941: 236; Schilthuizen and Rutjes 2001: 420; Schilthuizen et al. 2002b: 42; Schilthuizen and Vermeulen 2003: 96. *Kaliella microconus* (Mousson); Schilthuizen 2004: 94; Clements et al. 2008: 2762; Schilthuizen et al., 2011: 5; Schilthuizen et al. 2013: online supplementary data. Type from Fiji.
Sitala bandongensis Boettger, 1890: 141. *Coneuplecta bandongensis* (Boettger) Van Benthem Jutting, 1950: 388. Type from Indonesia, Java, Bandung, Gunung Malabar.
Sitala baritensis E.A. Smith, 1893: 343. *Durgellina baritensis* (E.A. Smith) Rensch, 1932: 68. Type from Malaysia, Sarawak, Barit Mountain.
Sitala singularis Godwin Austen, 1891: 39. Type from Malaysia, Borneo.

###### Examined material from Sabah.

*Interior Province*. Crocker Range N.P., Gua Laing c. 12 km North of Keningau (leg. J.J. Vermeulen, V 1094); Mahua (leg. M. Schilthuizen, BOR/MOL 2394). Gunung Trusmadi slopes, Gua Loloposon (leg. J.J. Vermeulen, V 13233). Pinangah valley, Batu Urun (= Bukit Sinobang) (leg. J.J. Vermeulen, V 1142, V 8007). Pun Batu c. 30 km West of Sepulut (leg. J.J. Vermeulen, V 1279). Sepulut valley, Batu Punggul (leg. J.J. Vermeulen, V 1988); Batu Temurung (leg. J.J. Vermeulen, V 8049; leg. M. Schilthuizen, BOR/MOL 808); Bukit Tinagas, East end of Batu Punggul limestone (leg. J.J. Vermeulen & M. Schilthuizen, V 7826; leg. M. Schilthuizen, BOR/MOL 801, BOR/MOL 3620); Gua Pungiton (leg. J.J. Vermeulen & M. Schilthuizen, V 7561); Gua Sanaron (leg. J.J. Vermeulen & M. Schilthuizen, V 7827). *Kudat Province*. Balambangan Island, Kok Simpul (leg. J.J. Vermeulen & M. Schilthuizen, V 9521); Gua Mundau (leg. T.S. Liew & M. Schilthuizen, BOR/MOL 4371). *Sandakan Province*. Kinabatangan valley, Batu Mawas (leg. T.S. Liew & M. Schilthuizen, BOR/MOL 1961, BOR/MOL 1997; leg. M. Schilthuizen, BOR/MOL 2392); Batu Materis (leg. T.S. Liew & B. Elahan, BOR/MOL 2122); Batu Keruak 2 near Sukau (leg. J.J. Vermeulen & M. Schilthuizen, V 9811, BOR/MOL 2389; leg. M. Salverda & H. van Oosten, BOR/MOL 2393); Batu Pangi (leg. J.J. Vermeulen & M. Schilthuizen, V 9664, BOR/MOL 2388); Batu Tai (not Bod Tai) near Gomantong (leg. J.J. Vermeulen & M. Schilthuizen, V 9588, BOR/MOL 2395; leg. T.S. Liew & B. Elahan, BOR/MOL 1919, BOR/MOL 1942); Batu Tomanggong Kecil (leg. J.J. Vermeulen & M. Schilthuizen, V 9706); Batu Tomanggong Besar (leg. T.S. Liew & B. Elahan, BOR/MOL 2288; leg. M. Schilthuizen, BOR/MOL 2391, BOR/MOL 802); Batu Tulug (Batu Putih) along road Lahad Datu-Sandakan, North of bridge over Kinabatangan River (leg. J.J. Vermeulen & H. Duistermaat, V 1486); Gomantong Hill 30 km South of Sandakan (leg. J.J. Vermeulen & H. Duistermaat, V 1620; leg. A. van Til, BOR/MOL 3266, BOR/MOL 3297; leg. T.S. Liew & J.P. King, BOR/MOL 3663); Tandu Batu (leg. J.J. Vermeulen & M. Schilthuizen, V 9620); Hill Ulu Sungai Resang (leg. M. Schilthuizen, BOR/MOL 2390); Unnamed hill near Sukau Police Station (leg. T.S. Liew & B. Elahan, BOR/MOL 2191, BOR/MOL 2227; leg. M. Schilthuizen, BOR/MOL 2387). Segama Valley, North end of limestone ridge on East bank Tabin River (leg. J.J. Vermeulen & M. Schilthuizen, V 7786). *Tawau Province*. Batu Baturong c. 50 km W.S.W. of Lahad Datu (leg. J.J. Vermeulen & H. Duistermaat, V 1860); North slope (leg. J.J. Vermeulen, V 7598). Danum Valley (leg. T. Kimsin & H.N. Chai, BOR/MOL 804; leg. H.A. Rutjes, BOR/MOL 805). Gua Madai c. 40 km S.S.W. of Lahad Datu (leg. J.J. Vermeulen & H. Duistermaat, V 1728); N.E. end (leg. J.J. Vermeulen, V 7709). Segama valley, hill N.W. of crossing road Sandakan-Lahad Datu with the Segama River (leg. J.J. Vermeulen & H. Duistermaat, V 1680); ‘Kirk’s Cave’ 8 km North of Lahad Datu (leg. J.J. Vermeulen, V 1233); Sabahmas Cave (leg. J.J. Vermeulen, V 7825, BOR/MOL 803). Semporna area, Segarong Hills, Batu Tengar, 25 km E.S.E. of Kunak (leg. J.J. Vermeulen & H. Duistermaat, V 1790); Bukit Pababola, 25 km E.S.E. of Kunak (leg. J.J. Vermeulen & H. Duistermaat, V 1776). *West Coast Province*. Kinabalu N.P., Poring Hot Springs, along path to waterfall (leg. J.J. Vermeulen, V 13015); Orchid Garden (leg. J.J. Vermeulen, V 13028); Serinsim (leg. M. Schilthuizen, BOR/MOL 3075). Kota Kinabalu, Kiansom (leg. M. Schilthuizen, BOR/MOL 807).

###### Description.

Shell very small, thin, somewhat translucent, pale brown, conical with flat sides; apex rounded. Surface shiny. Top whorls moderately convex, outer whorls slightly convex, last whorl angular at the periphery, slightly convex below the periphery. *Protoconch* whorls convex, with predominant, well-spaced, very thin spiral threads, and very fine, densely and somewhat irregularly placed radial riblets. *Teleoconch*: Spiral sculpture predominant, above the periphery with fine, well-spaced, very thin spiral threads (usually 10 or fewer on the last whorl); below the periphery and close to it with a few to many more of such threads, towards the umbilicus sometimes with much finer, densely placed grooves. Radial sculpture teleoconch: above the periphery growth lines grading into very fine, densely and somewhat irregularly placed radial riblets; below the periphery with irregularly spaced growth lines only. *Umbilicus* open, very narrow. *Dimensions*: Height up to 3.0 mm; width up to 2.9 mm; diameters of the first four whorls 0.45–0.60 mm, 0.75–1.00 mm, 1.05–1.45 mm, 1.45–2.00 mm respectively; number of whorls up to 6 1/8; height aperture up to 0.9 mm; width aperture up to 1.4 mm. Radula: central 3-cuspid; laterals and marginals similar, serrate with 2 large cones at the tip.

**Figure 52–53.**
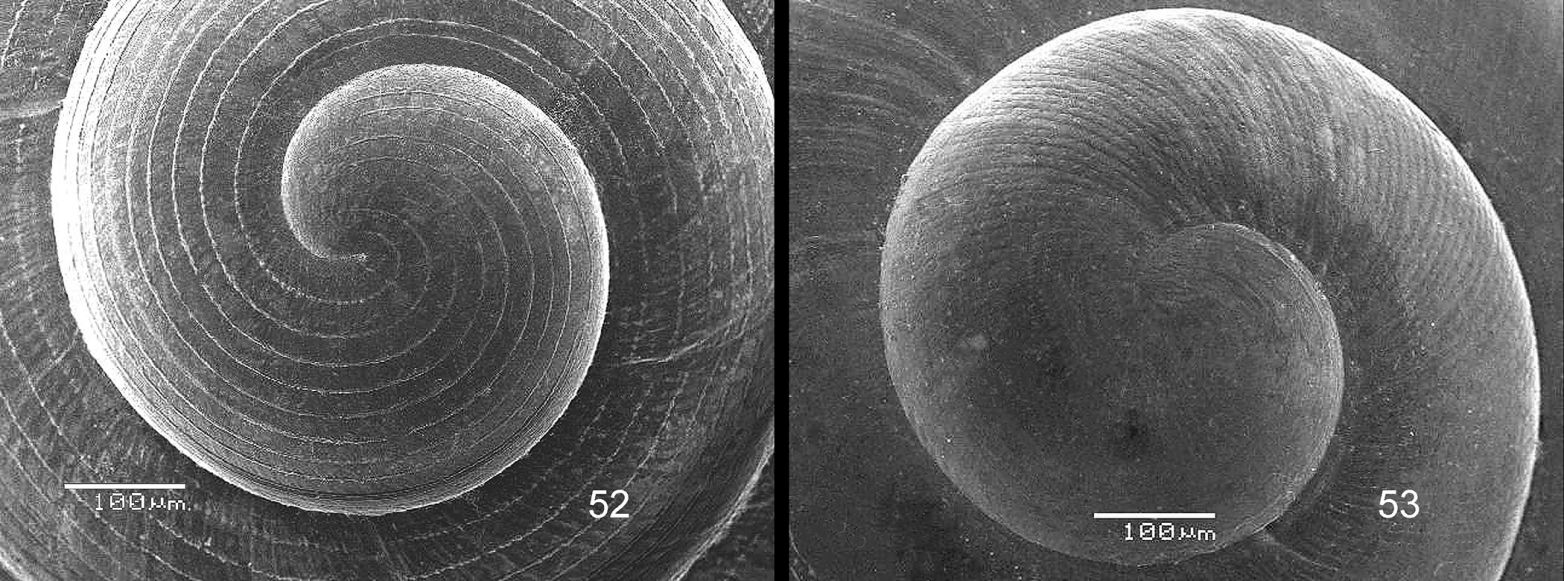
**52** *Kaliella microconus* (Mousson, 1865). SEM image of protoconch (Malaysia, Sabah, Kinabatangan river valley, Batu Mawas, BOR/MOL 1961, lost) **53** *Kaliella accepta* (E.A. Smith, 1895). SEM image of protoconch (Malaysia, Sabah, Kinabatangan river valley, BOR/MOL 1917).

**Figure 54–55.**
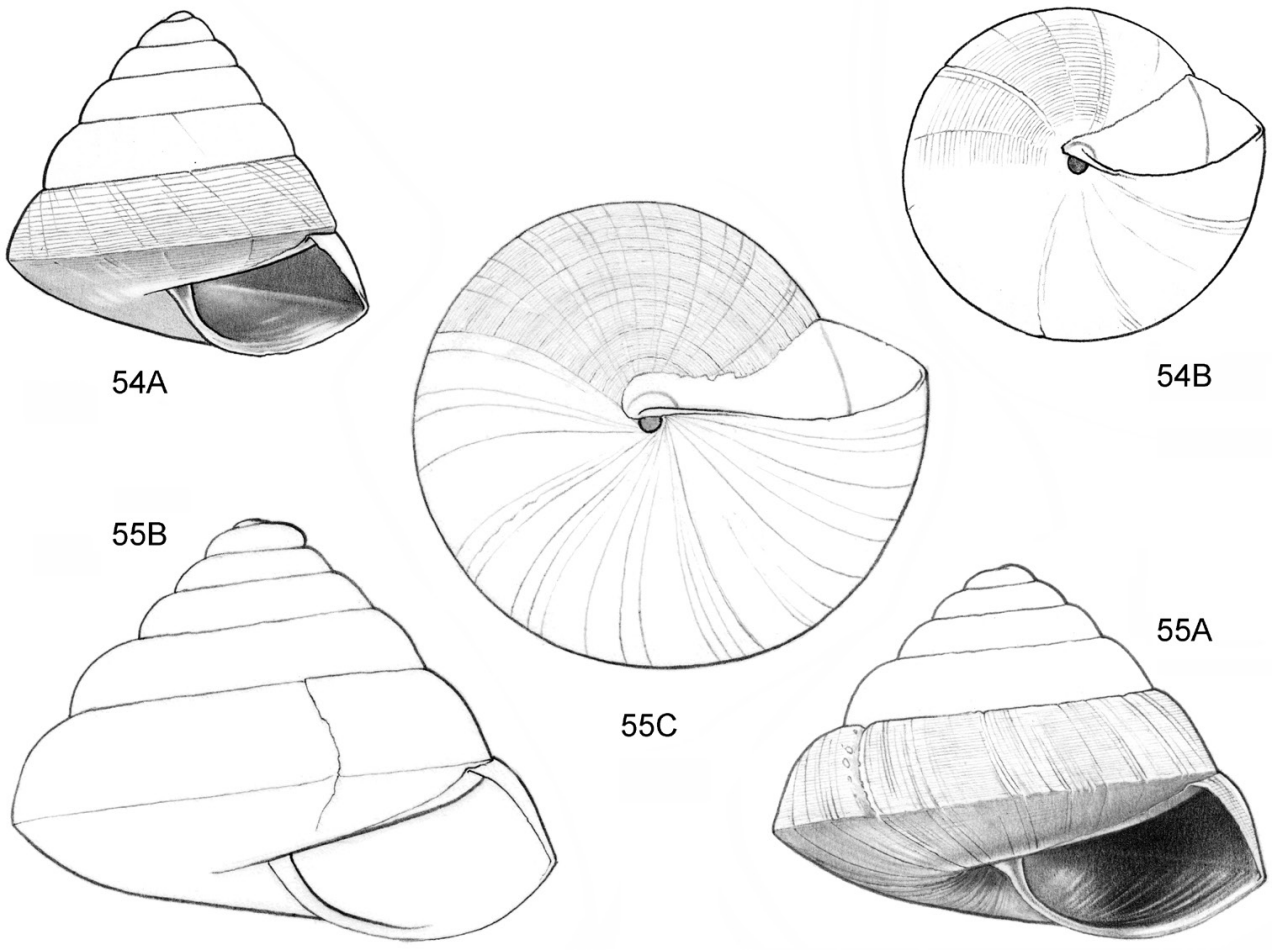
**54** *Kaliella microconus* (Mousson, 1865). **54A** Frontal view, shell 2.3 mm high **54B** Umbilical view (Indonesia, Kalimantan Selatan, between Batulichin and Benualawas, V 3284) **55** *Kaliella humilis* (Tillier & Bouchet, 1988). **55A** Frontal view, shell 2.8 mm high **55B** Frontal view, shell 3.3 mm high **55C** Umbilical view (a, c. Malaysia, Sabah, Kinabalu N.P., V 14330, aperture reconstructed B Malaysia, Sabah, Kinabalu N.P., BOR/MOL 2882).

###### Habitat in Sabah and distribution.

Primary and secondary forest on limestone and sandstone soil. Alt. up to 1000 m, elsewhere up to 1700 m alt. **Sabah: widespread**. Also in Sarawak; Kalimantan. Distribution elsewhere: Vietnam; Peninsular Malaysia to Java; Eastwards to Australia; Fiji; Samoa.

###### Remarks.

Information on the radula is from Van Benthem Jutting (1950). No Sabah material included in *Kaliella microconus* has been checked for radula characters.

##### Kaliella humilis

Taxon classificationAnimaliaStylommatophoraEuconulidae

(Tillier & Bouchet, 1988)

Figure 55

Kionghutania humilis Tillier & Bouchet, 1988: 275. *Kaliella humilis* (Tillier & Bouchet) Liew et al., 2010: Appendix S1 in online Supporting Information. Type from Malaysia, Sabah, Kinabalu N.P.

###### Examined material from Sabah.

*Interior Province*. Crocker Range N.P., West of the km 10 marker on the road Tambunan-Ranau, Mahua Waterfall (leg. M. Schilthuizen, BOR/MOL 2882). *West Coast Province*. Kinabalu N.P., Summit trail between 3080 and 3330 m (leg. T. Narainan, V 13489, V 13480, BOR/MOL 2675, BOR/MOL 2678, BOR/MOL 2683; leg. T.S. Liew, V 14330, BOR/MOL 6037).

###### Description.

Shell very small, thin, about opaque, greenish brown, (low) conical with flat or slightly convex sides; apex rounded. Surface shiny. Top whorls moderately convex, outer whorls moderately convex and somewhat shouldered, last whorl angular at the periphery, slightly convex below the periphery. *Protoconch* whorls convex, with predominant, very fine, well-spaced, thin spiral threads, radial sculpture inconspicuous. *Teleoconch*: Spiral sculpture predominant, above the periphery with fine, very densely placed, somewhat irregularly spaced, thin spiral threads (30 or more on the fifth whorl); below the periphery with very fine, widely spaced threads, with or without numerous still finer, densely placed grooves in between. Radial sculpture teleoconch: above the periphery with raised growth lines at irregular intervals, most distinctly so just below the periphery; below the periphery the growth lines are less distinct. *Umbilicus* open, narrow. *Dimensions*: Height up to 3.3 mm; width up to 4.1 mm; diameters of the first four whorls 0.6–0.7 mm, 1.05–1.15 mm, 1.6–1.7 mm, 2.35–2.50 mm respectively; number of whorls up to 5 7/8; height aperture up to 1.4 mm; width aperture up to 2 mm. Radula: central 3-cuspid; laterals 3-cuspid; marginals serrate with 2 large cones at the tip.

###### Habitat in Sabah and distribution.

Submontane to subalpine forest on granodiorite and sandstone soil. Alt. 1200–3300 m. **Sabah: Mount Kinabalu; Crocker Range.** Endemic to Sabah.

Cross diagnosis: Shares the distinct sculpture of spiral threads above and below the periphery with *Kaliella microconus*, but differs by the wider shell with more convex, slightly shouldered whorls, and the much more numerous and more densely placed spiral threads. The sympatric *Kaliella kinabaluensis* and *Kaliella nephelophila* differ unequivocally by the slightly more concave outline of the spire, whorls without a shoulder, and by the absence of spiral threads on the lower surface (a fine sculpture of spiral grooves may be present).

##### Kaliella nephelophila

Taxon classificationAnimaliaStylommatophoraEuconulidae

(Tillier & Bouchet, 1988)

Fig. 56 , 62

Kionghutania nephelophila Tillier & Bouchet, 1988: 276. *Kaliella nephelophila* (Tillier & Bouchet) Liew et al., 2010: Appendix S1 in online Supporting Information. Type from Malaysia, Sabah, Kinabalu N.P.

###### Examined material from Sabah.

*West Coast Province*. Kinabalu N.P., Paka Cave (leg. Tachaini Narainan, V 13495).

###### Description.

Shell very small, very thin, somewhat translucent, slightly brownish green, conical with flat or slightly concave sides; apex rounded. Surface shiny. Whorls slightly to moderately convex and not shouldered, last whorl angular at the periphery, slightly to moderately convex below the periphery. *Protoconch* whorls rather flat, with predominant, fine, somewhat inconspicuous, well-spaced, thin spiral threads; radial sculpture inconspicuous. *Teleoconch*: first whorls with moderately-spaced, thin spiral threads, outer whorls above the periphery with fine, rather densely placed, somewhat wavy spiral threads (less than 80 on the fifth whorl) which are slightly more distinct towards the periphery; below the periphery without spiral sculpture, or locally with fine spiral striation. Radial sculpture teleoconch: above the periphery with distinctly raised growth lines at irregular intervals; below the periphery these are slightly less distinct. *Umbilicus* almost closed. *Dimensions*: Height up to 3.8 mm; width up to 4.6 mm; diameters of the first four whorls 0.65–0.80 mm, 1.05–1.20 mm, 1.60–1.75 mm, c. 2.7 mm respectively; number of whorls up to 5 5/8; height aperture up to 1.9 mm; width aperture up to 2.4 mm. Radula: central 3-cuspid; laterals 3-cuspid; marginals serrate with 2 large cones at the tip.

**Figure 56–58.**
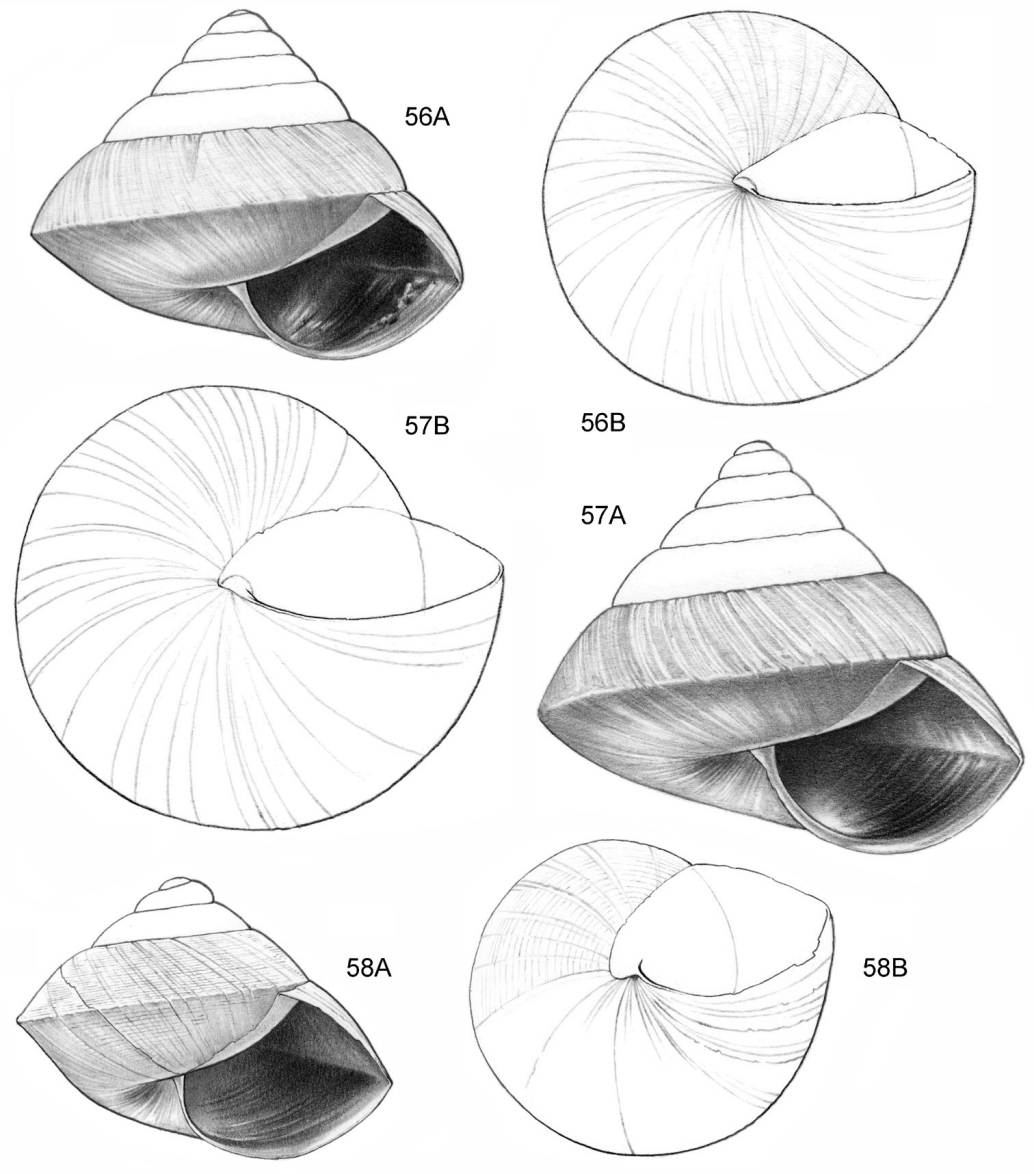
**56** *Kaliella nephelophila* (Tillier & Bouchet, 1988). **56A** Frontal view, shell 3.5 mm high **56B** Umbilical view (Malaysia, Sabah, Kinabalu N.P., V 13495, shell partially reconstructed) **57** *Kaliella kinabaluensis* (Tillier & Bouchet, 1988). **57A** Frontal view, shell 5.3 mm high **57B** Umbilical view (Malaysia, Sabah, Kinabalu N.P., V 13478, last 1/3 whorl reconstructed) **58** *Kaliella dendrophila* (Van Benthem Jutting, 1950). **58A** Frontal view, shell 2.7 mm high **58B** Umbilical view (Malaysia, Sabah, Sandakan Province, Kinabatangan river valley, Batu Pangi, V 9647).

###### Habitat in Sabah and distribution.

Upper montane forest on granodiorite soil.Alt. 3000–3100 m. **Sabah: Mount Kinabalu (summit trail area).** Endemic to Sabah.

###### Cross diagnosis.

*Kaliella nephelophila* is similar to *Kaliella kinabaluensis*. Diagnostic is the spiral sculpture above the periphery: fine but still distinct spiral threads, particularly towards the periphery, in *Kaliella nephelophila*; an indistinct, dense spiral striation in *Kaliella kinabaluensis*. The two species differ anatomically; see Tillier and Bouchet 1988.

##### Kaliella kinabaluensis

Taxon classificationAnimaliaStylommatophoraEuconulidae

(Tillier & Bouchet, 1988)

Figure 57

Kionghutania kinabaluensis Tillier & Bouchet, 1988: 271. *Kaliella kinabaluensis* (Tillier & Bouchet) Liew et al., 2010: Appendix S1 in online Supporting Information. Type from Malaysia, Sabah, Kinabalu N.P.

###### Examined material from Sabah.

*West Coast Province*. Kinabalu N.P., Gunting Lagadan (leg. Tachaini Narainan, V 13478).

###### Description.

Shell small, very thin, somewhat translucent, slightly greenish brown, conical with flat or slightly concave sides; apex rounded. Surface shiny. Whorls moderately convex and not shouldered, last whorl angular at the periphery, moderately convex below the periphery. *Protoconch* whorls somewhat convex, with spiral sculpture predominant but inconspicuous, very fine, well-spaced, thin threads; radial sculpture inconspicuous. *Teleoconch*: subordinate spiral striation, first whorls with traces of very inconspicuous, moderately-spaced striation, outer whorls above the periphery with a very fine, very densely placed, somewhat wavy striation (more than 100 striae on the last whorl); below the periphery almost smooth, with faint traces of an even finer spiral striation only towards the periphery. Radial sculpture teleoconch: Above the periphery with coarse, distinctly raised growth lines at irregular intervals; below the periphery these are slightly less distinct. *Umbilicus* closed. *Dimensions*: Height up to 5.6 mm; width up to 6.2 mm; diameters of the first four whorls 0.8–0.85 mm, 1.2–1.3 mm, 1.80–1.95 mm, c. 2.7 mm respectively; number of whorls up to 6 1/8; height aperture up to 2.4 mm; width aperture up to 3.2 mm. Radula: central 3-cuspid; laterals 3-cuspid; marginals serrate with 2 large cones at the tip.

###### Habitat in Sabah and distribution.

Subalpine forest on granodiorite soil, at 3300–3400 m alt. **Sabah: Mount Kinabalu (summit trail area).** Endemic to Sabah.

###### Cross diagnosis.

Very similar to *Kaliella nephelophila*; see note under the latter.

##### Kaliella dendrophila

Taxon classificationAnimaliaStylommatophoraEuconulidae

(Van Benthem Jutting, 1950)

Figure 58 , 63

Liardetia dendrophila Van Benthem Jutting, 1950: 407; Clements et al. 2008: 2762; Schilthuizen et al. 2013: online supplementary data. Type from Indonesia, Java.

###### Examined material from Sabah.

*Interior Province*. Crocker Range N.P., Gua Laing c. 12 km North of Keningau (leg. J.J. Vermeulen, V 1126). *Sandakan Province*. Kinabatangan valley, Batu Pangi (leg. J.J. Vermeulen & M. Schilthuizen, V 9647); Batu Tomanggong Besar (leg. M. Schilthuizen, BOR/MOL 2357; leg. A. van Til, BOR/MOL 3834).

###### Description.

Shell very small, thin, translucent, very pale greenish to yellowish, somewhat depressed-conical with flat sides; apex rounded. Surface shiny. *Whorls*: Top whorls convex, outer whorls (almost) flat, last whorl sharply angular at the periphery, almost flat or slightly convex above and below. *Protoconch* whorls convex, with distinct, moderately spaced, rounded spiral threads, radial sculpture absent or inconspicuous, subordinate. *Teleoconch*: A slight, obtuse keel present around the periphery; above and below with rather distinct, well-spaced (particularly half-way between suture and periphery, as well as just below the periphery) spiral threads, which consist of rows of densely placed beads which are often elongated in the direction of the growth lines. Radial sculpture: above the periphery with some rather distinct, irregularly spaced, somewhat raised growth lines, next to these locally some very fine, inconspicuous, moderately spaced, low and thin riblets; below the periphery with a few inconspicuous, irregularly spaced growth lines only. *Umbilicus* open, very narrow, or closed. *Dimensions*: Height up to 2.7 mm; width up to 4.1 mm; diameters of the first four whorls 0.55–0.67 mm, 1.1–1. 5 mm, 2.1–2.7 mm, c. 3.6 mm respectively; number of whorls up to 4 1/2; height aperture up to 1.6 mm; width aperture up to 2 mm.

###### Habitat in Sabah and distribution.

Primary and secondary forest on limestone soil. Alt. 0–500 m. **Sabah: Crocker Range; Kinabatangan River valley.**

**River valley.** Distribution elsewhere: Peninsular Malaysia; Java; Sulawesi; Ambon.

###### Cross diagnosis.

Easily recognizable within group 1 by the spiral sculpture on the teleoconch, above the periphery. *Kaliella punctata*, of group 2, has a similar spiral sculpture, but consisting of pits. It also has a higher conical shell with whorls that increase much less rapidly in width. *Kaliella gregaria* and *Kaliella microsoma* have a similar shell but a spiral sculpture not consisting of rows of beads.

###### Remarks.

Van Benthem Jutting (1950: 407) describes the spiral striation as engraved into the shell surface. SEM images show that it consists of interrupted spiral threads.

#### Group 2. Protoconch with fine spiral grooves, usually about as strong as the radial sculpture or weaker, or protoconch without spiral sculpture.

##### Group 2a. Diameter of the third whorl more than 2.3 mm (also check *Kaliella punctata* and *Kaliella calculosa*, with incidental shells having a third whorl of 2.2 mm diameter).

###### Kaliella gregaria

Taxon classificationAnimaliaStylommatophoraEuconulidae

(Tillier & Bouchet, 1988)

Figure 59

Gunongia gregaria Tillier & Bouchet, 1988: 264. *Kaliella gregaria* (Tillier & Bouchet) Liew et al., 2010: Appendix S1 in online Supporting Information. Type from Malaysia, Sabah, Kinabalu N.P.

####### Examined material from Sabah.

*West Coast Province*. Kinabalu N.P., Laban Rata (leg. Schilthuizen & P. Koomen, V 13531); Bowen route between 3320 and 3376 m (leg. T.S. Liew, V 14329, BOR/MOL 6038, BOR/MOL 6039).

####### Description.

Shell small, very thin, somewhat translucent, greenish or brownish, depressed-conical with flat sides; apex rounded. Surface shiny. *Whorls* moderately convex, last whorl angular and slightly compressed at the periphery, rounded above and below the periphery. *Protoconch* whorls convex, with numerous fine, rather densely placed radial riblets; spiral sculpture subordinate and inconspicuous (visible at 40 times magnification), numerous very densely placed grooves. *Teleoconch*: Last whorl with a peripheral spiral thread coinciding with the suture of the penultimate whorl, above and below this numerous continuous, fine, densely and regularly placed spiral grooves. Radial sculpture: above the periphery rather distinct, irregularly spaced, somewhat raised growth lines, next to these fine, densely and regularly placed (about as densely as the spiral grooves) riblets; below the periphery with similar but less distinct radial sculpture. *Umbilicus* closed. *Dimensions*: Height up to 5.2 mm; width up to 6.8 mm; diameters of the first three whorls c. 0.75 mm, c. 1.65 mm, c. 3.25 mm respectively; number of whorls up to c. 4, height aperture up to 3.4 mm; width aperture up to 4 mm.

**Figure 59.**
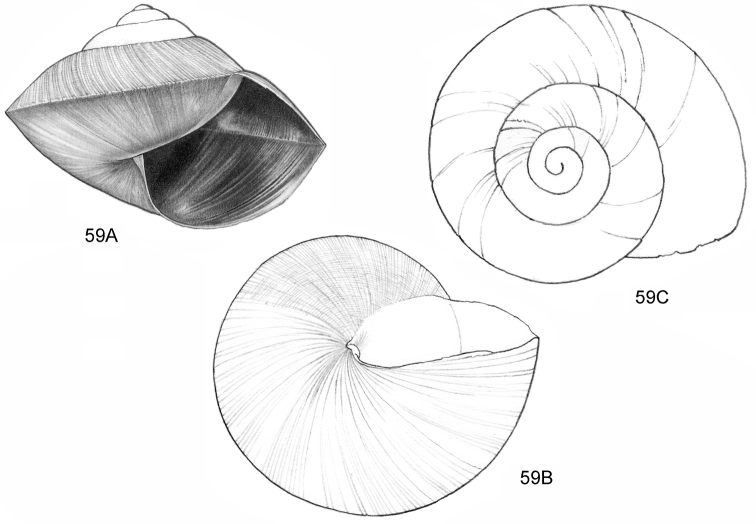
*Kaliella gregaria* (Tillier & Bouchet, 1988). **A** Frontal view **B** Umbilical view **C** Apical view (Malaysia, Sabah, Kinabalu N.P., V 14329, aperture reconstructed).

####### Habitat in Sabah and distribution.

Subalpine forest on diorite bedrock. Alt. 3300–3400 m. **Sabah: Mount Kinabalu (summit trail area).** Endemic to Sabah.

####### Cross diagnosis.

The whorls increase faster in width than in any other Borneo *Kaliella* species. In this respect *Kaliella sublaxa* is most similar.

###### Kaliella eurytrochus

Taxon classificationAnimaliaStylommatophoraEuconulidae

Vermeulen, Liew & Schilthuizen sp. n.

http://zoobank.org/DF754721-4B13-4E3A-8C5A-75A11CC1D8BB

Figure 60

####### Holotype.

Malaysia, Sabah, Interior Province, Gunung Trusmadi slopes, Gua Loloposon (RMNH.5003922).

####### Examined material from Sabah.

*Interior Province*. Gunung Trusmadi slopes, Gua Loloposon (leg. J.J. Vermeulen, V 13237). *Tawau Province*. Danum Valley Conservation Area, 2 km N.W. of Danum Valley Field Centre (leg. H.A. Rutjes, V 13513).

####### Description.

Shell small, rather thin, somewhat translucent or opaque, pale yellow-corneous to white, depressed-conical with approx. flat or slightly convex sides; apex rounded. Surface shiny. *Whorls* moderately convex, last whorl angular at the periphery, rounded above and below the periphery. *Protoconch* whorls convex, with numerous fine, rather densely placed radial riblets, which are most distinct below the suture; numerous approx. equally strong, densely placed spiral grooves cutting into the crests of the radial riblets. *Teleoconch*: Last whorl with a peripheral spiral thread coinciding with the suture of the penultimate whorl, above and below this numerous fine, moderately and regularly spaced, continuous spiral grooves. Radial sculpture: growth lines above the periphery, at somewhat irregular intervals, which are approx. as strong as the spiral sculpture, and which are most distinct just below the suture. *Umbilicus* closed. *Dimensions*: Height up to 2.65 mm; width up to 3.8 mm; diameters of the first four whorls 0.80–0.85 mm, 1.5–1.6 mm, 2.35–2.50 mm, 3.6–3.7 mm respectively; number of whorls up to 4 1/8; height aperture up to 1.5 mm; width aperture up to 2.0 mm.

**Figure 60–61.**
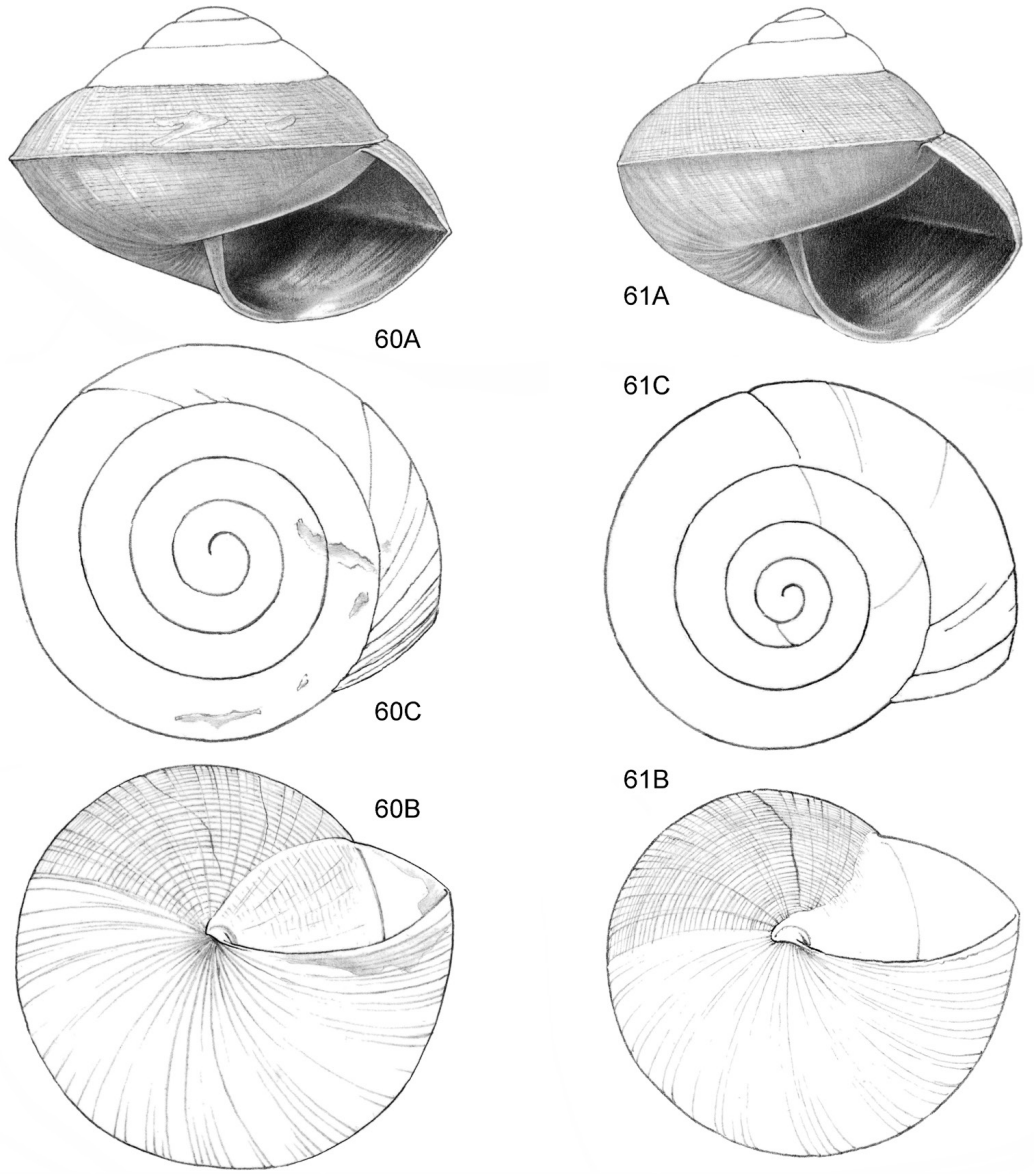
**60** *Kaliella eurytrochus* sp. n. **60A** Frontal view, shell 2.6 mm high **60B** Umbilical view **60C** Apical view (Malaysia, Sabah, Gunung Trusmadi slopes, Gua Loloposon, RMNH.5003922, holotype) **61** *Kaliella sublaxa* sp. n. **61A** Frontal view, shell 3.3 mm high **61B** Umbilical view **61C** Apical view (Malaysia, West Coast Province. Crocker Range N.P., km 54 marker on the road Kota Kinabalu-Tambunan, Gunung Mas, RMNH.5003923, holotype, aperture reconstructed).

**Figure 62–65.**
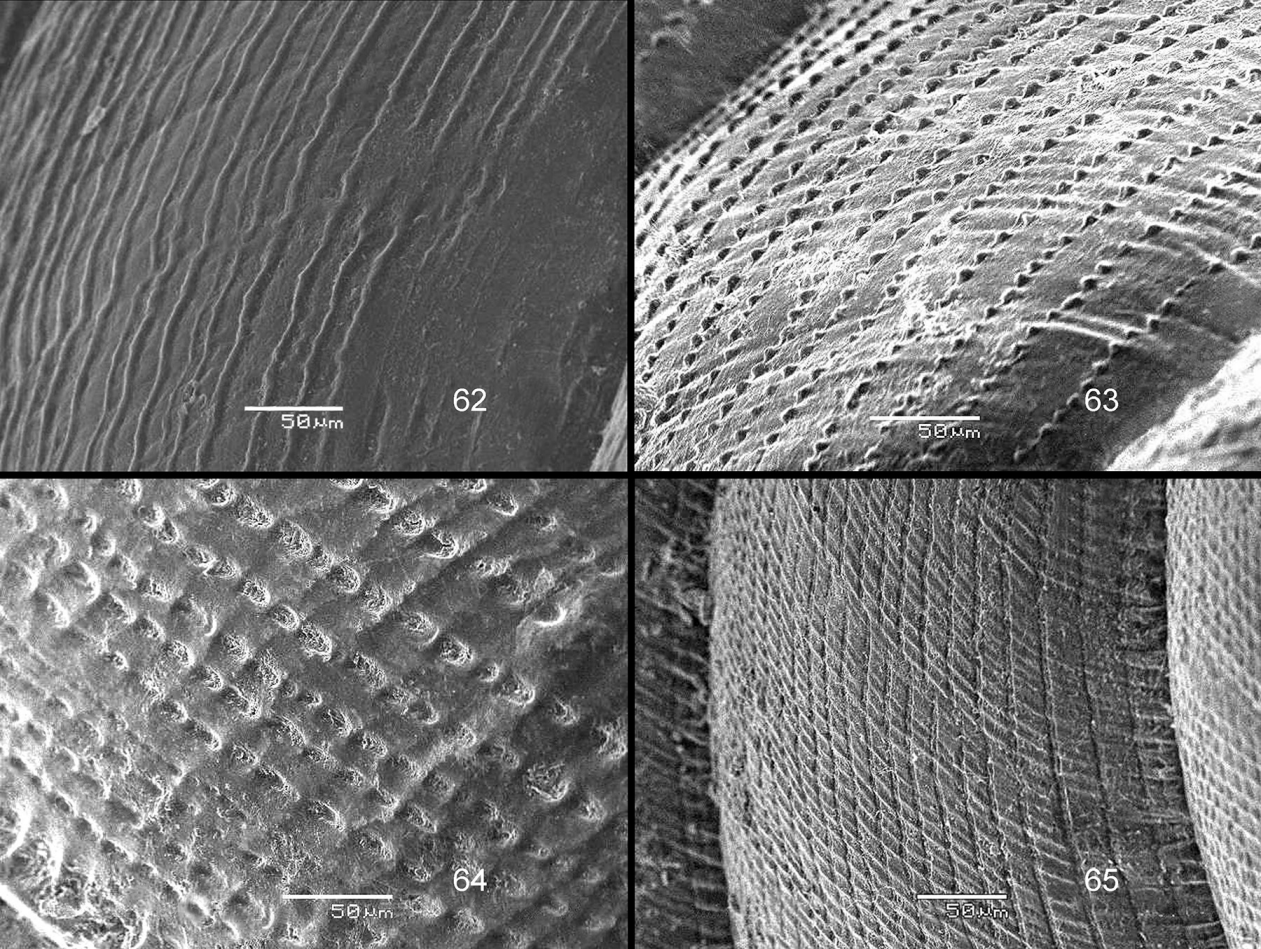
**62** *Kaliella nephelophila* (Tillier & Bouchet, 1988). SEM image of the third whorl (Malaysia, Sabah, Kinabalu N.P., Pakka, 3080 m alt., (BOR/MOL 2873, lost) **63** *Kaliella dendrophila* (Van Benthem Jutting, 1950). SEM image of the third whorl (Malaysia, Sabah, Kinabatangan River valley, Gomantong, BOR/MOL 3661, lost) **64** *Kaliella punctata* sp. n. SEM image of the third whorl (Malaysia, Sabah, Sepulut valley, Batu Punggul, BOR/MOL 4325) **65** *Kaliella calculosa* (Gould, 1852). SEM image of the third whorl (Malaysia, Sabah, Tawau Province, Danum Valley, BOR/MOL 815).

####### Habitat in Sabah and distribution.

Primary forest on limestone and sandstone soil. Alt. c. 400–1000 m. **Sabah: Gunung Trusmadi; Danum Valley. Two localities.** Endemic to Sabah.

####### Cross diagnosis.

Differs from *Kaliella gregaria* by its smaller size, at the same number of whorls. In *Kaliella eurytrochus*, the whorls increase less fast in width; compare the diameters of the third whorl of both species. The shell of *Kaliella eurytrochus* is also distinctly thicker, and less fragile.

####### Etymology.

The name refers to the inflated shell shape [*eurus* (Gr.) = wide; *trokhos* (Gr.) = wheel, a word often used for the gastropod spire].

###### Kaliella sublaxa

Taxon classificationAnimaliaStylommatophoraEuconulidae

Vermeulen, Liew & Schilthuizen sp. n.

http://zoobank.org/CE4B221C-E4DD-4324-8AFB-8F305C6A7196

Figure 61

####### Holotype.

Malaysia, Sabah, West Coast Province, Crocker Range N.P., km 54 marker on the road Kota Kinabalu-Tambunan, Gunung Mas (RMNH.5003923).

####### Examined material from Sabah.

*Interior Province*. Gunung Trusmadi slopes, Gua Loloposon (leg. J.J. Vermeulen, V 13259). *West Coast Province*. Crocker Range N.P., km 54 marker on the road Kota Kinabalu-Tambunan, Gunung Mas (leg. M. Schilthuizen, J.J.M. van Alphen & J. van Alphen, V 13493).

####### Description.

Shell small, very thin, somewhat translucent, pale yellowish-brown, somewhat depressed-conical with flat or slightly concave sides; apex rounded. Surface shiny. *Whorls* moderately convex, last whorl slightly angular, rounded above and below the periphery. *Protoconch* whorls rather flat, with numerous fine, densely placed radial riblets; spiral sculpture virtually absent. *Teleoconch*: Last whorl with a thin peripheral spiral thread coinciding with the suture of the penultimate whorl, above and below this numerous fine, densely and regularly placed, continuous spiral grooves. Radial sculpture: above the periphery with a few growth lines, next to these fine, densely and regularly placed (about as dense and as strong as the spiral grooves) riblets; below the periphery with similar but less distinct radial sculpture. *Umbilicus* closed. *Dimensions*: Height up to 3.5 mm; width up to 4.1 mm; diameters of the first four whorls 0.7–0.8 mm, 1.35–1. 50 mm, 2.40–2.65 mm, c. 3.9 mm respectively; number of whorls up to c. 4; height aperture up to 2.2 mm; width aperture up to 2.4 mm.

####### Habitat in Sabah and distribution.

Primary and secondary forest on limestone and sandstone soil. Alt. 900–1700 m. **Sabah: Crocker Range; Gunung Trusmadi.** Endemic to Sabah.

####### Cross diagnosis.

In general shape most similar to *Kaliella dendrophila*, differs by the protoconch sculpture with predominant radial riblets and the more convex outer whorls. Also similar to *Kaliella calculosa* and *Kaliella microsoma*, differs by the less dense mode of coiling (compare the width of the third and fourth whorl).

It differs from *Kaliella gregaria* by its denser mode of coiling (compare diameters of the third whorl), and from *Kaliella eurytrochus* by the more dense spiral sculpture on the outer whorls, above the periphery, as well as by the larger aperture at the same number of whorls.

####### Etymology.

The name refers to the mode of coiling [*sub-laxa* (L.) = a little loose].

##### Group 2b. Periphery of last whorl angular in adult shells, or with a peripheral spiral thread. Diameter of the third whorl 2.2 mm or less. Spiral sculpture teleoconch fine but distinct above the periphery.

###### Kaliella phacomorpha

Taxon classificationAnimaliaStylommatophoraEuconulidae

Vermeulen, Liew & Schilthuizen sp. n.

http://zoobank.org/362C60FC-DBBC-4DB9-864C-69CF9504577C

Figure 66

####### Holotype.

Malaysia, Sabah, Interior Province, Gunung Trusmadi slopes, Gua Loloposon (RMNH.5003924).

####### Examined material from Sabah.

*Interior Province*. Gunung Trusmadi slopes, Gua Loloposon (leg. J.J. Vermeulen, V 13215).

####### Description.

Shell very small, rather thin, somewhat translucent or opaque, yellow-brown corneous, depressed-conical to almost lenticular with slightly convex sides; apex rounded. Surface shiny. *Whorls* moderately convex, the last whorl angular at the periphery, rounded above and below the periphery. *Protoconch* whorls convex, with numerous fine, densely placed radial riblets; spiral sculpture subordinate, consisting of approx. 12 very fine, densely placed spiral grooves cutting into the crests of the radial riblets. *Teleoconch*: Last whorl with a peripheral spiral thread coinciding with the suture of the penultimate whorl, above and below this numerous continuous, fine, moderately and regularly spaced, continuous spiral grooves. Radial sculpture: growth lines above the periphery only. *Umbilicus* open, narrow. *Dimensions*: Height up to 1.7 mm; width up to 2.6 mm; diameters of the first four whorls 0.55–0.65 mm, 0.95–1.10 mm, 1.45–1.60 mm, 2.05–2.30 mm respectively; number of whorls up to 4 3/4; height aperture up to 1.05 mm; width aperture up to 1.3 mm.

**Figure 66–67.**
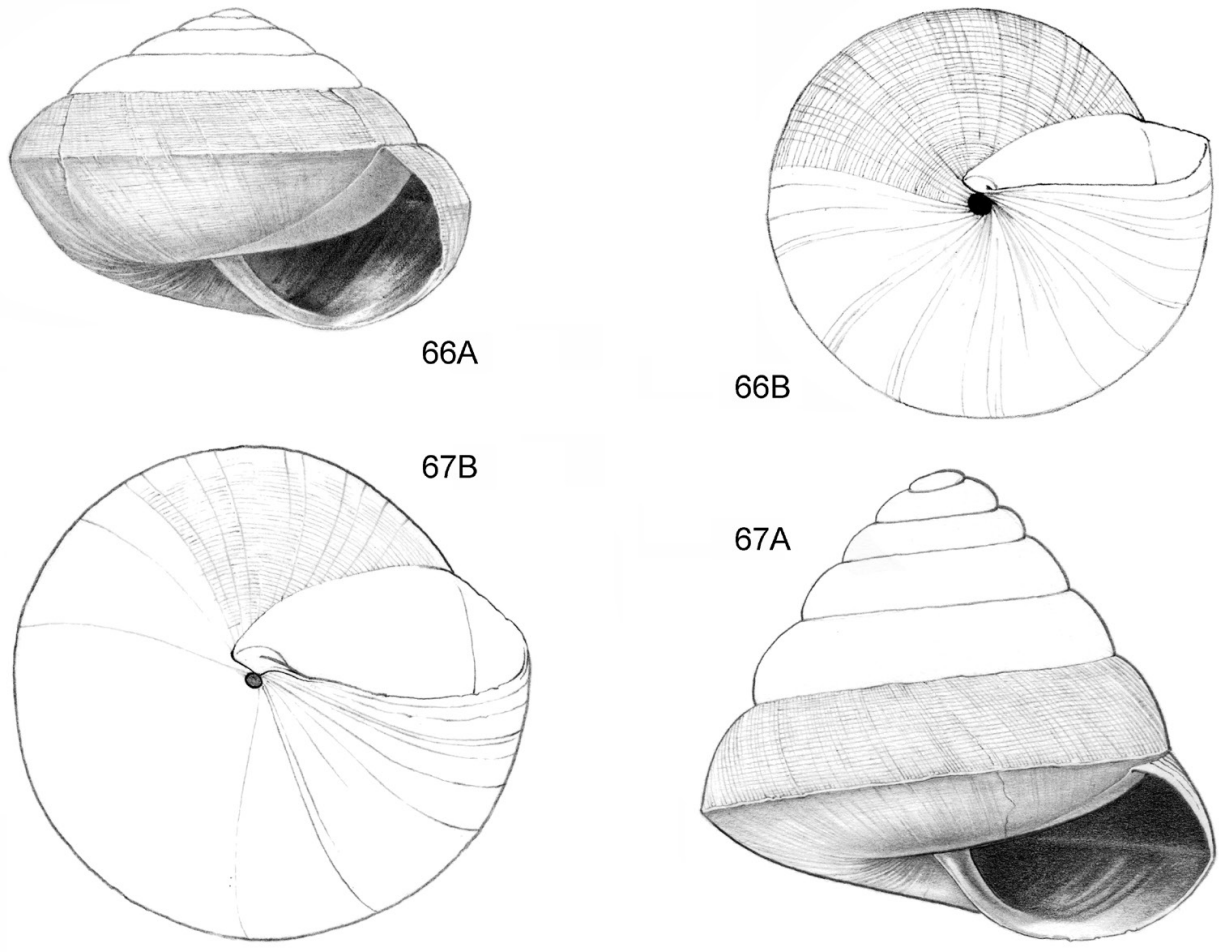
**66** *Kaliella phacomorpha* sp. n. **66A** Frontal view, shell 1.8 mm high **66B** Umbilical view 50C Apical view (Malaysia, Sabah, Gunung Trusmadi slopes, Gua Loloposon, RMNH.5003924, holotype) **67** *Kaliella punctata* sp. n. **67A** Frontal view, shell 4.5 mm high **67B** Umbilical view (Malaysia, Sabah, Gua Pungiton in Sepulot River valley, RMNH.5003925, holotype).

####### Habitat in Sabah and distribution.

Found in primary forest on limestone soil. Alt. 900–1000 m. **Sabah: Gunung Trusmadi.** Endemic to Sabah.

####### Cross diagnosis.

Uniquely identified among Sabah *Kaliella* species by its depressed-conical, almost lenticular spire.

####### Etymology.

The name refers to the shell shape [*phakos* (Gr.) = lentil; *morphe* (Gr.) = shape].

###### Kaliella punctata

Taxon classificationAnimaliaStylommatophoraEuconulidae

Vermeulen, Liew & Schilthuizen sp. n.

http://zoobank.org/1277B7FB-D848-476F-91CA-79D6A770C33B

Figure 64 , 67

Kaliella punctata nomen nudum, Clements et al. 2008: 2762; Schilthuizen et al. 2013: online supplementary data.
Coneuplecta angulata auct., Schilthuizen et al. 2002: 256–257; Schilthuizen et al. 2002b: 37, 41–42; Schilthuizen and Vermeulen 2003: 96; Clements et al. 2008: 2762.

####### Holotype.

Malaysia, Sabah, Interior Province, Sepulut River valley, Gua Pungiton (RMNH.5003925).

####### Examined material from Sabah.

*Interior Province*. Crocker Range N.P., Gua Laing c. 12 km North of Keningau (leg. J.J. Vermeulen, V 1108; leg. M. Schilthuizen, BOR/MOL 821). Gunung Trusmadi slopes, Gua Loloposon (leg. J.J. Vermeulen, V 13235). Pinangah valley, Batu Urun (= Bukit Sinobang) (leg. J.J. Vermeulen, V 8005). Pun Batu c. 30 km West of Sepulut (leg. J.J. Vermeulen, V 1277). Sepulut valley, Batu Punggul (leg. J.J. Vermeulen, V 1987; leg. M. Schilthuizen, BOR/MOL 788; leg. T.S. Liew, M. Schilthuizen & S. Chiba, BOR/MOL 4325); Batu Temurung (leg. J.J. Vermeulen, V 8052; leg. M. Schilthuizen, BOR/MOL 819); Bukit Tinagas, East end of Batu Punggul limestone (leg. J.J. Vermeulen & M. Schilthuizen, V 7632); Gua Pungiton (leg. J.J. Vermeulen & M. Schilthuizen, V 7829, BOR/MOL 786); Gua Sanaron (leg. J.J. Vermeulen & M. Schilthuizen, V 7669; leg. M. Schilthuizen, BOR/MOL 794, BOR/MOL 806). *Sandakan Province*. Kinabatangan valley, Batu Keruak 2 near Sukau (leg. J.J. Vermeulen & M. Schilthuizen, V 9796, BOR/MOL 2364; leg. M. Salverda & H. van Oosten, BOR/MOL 2367); Batu Materis (leg. T.S. Liew & B. Elahan, BOR/MOL 2119; leg. M. Schilthuizen, BOR/MOL 2368); Batu Tai (not Bod Tai) near Gomantong (leg. J.J. Vermeulen & M. Schilthuizen, V 9605, BOR/MOL 2365; leg. T.S. Liew & B. Elahan, BOR/MOL 1918); Bod Tai (leg. M. Schilthuizen, BOR/MOL 2366); Batu Tomanggong Besar (leg. M. Schilthuizen, BOR/MOL 795); Batu Tomanggong Kecil (leg. J.J. Vermeulen & M. Schilthuizen, V 9694; leg. T.S. Liew & B. Elahan, BOR/MOL 2029, BOR/MOL 2059); Batu Tulug (Batu Putih) along road Lahad Datu-Sandakan, North of bridge over Kinabatangan River (leg. J.J. Vermeulen & H. Duistermaat, V 1485; leg. M. Schilthuizen, BOR/MOL 796); Gomantong Hill 30 km South of Sandakan (leg. J.J. Vermeulen & H. Duistermaat, V 1616; leg. A. van Til, BOR/MOL 3314; leg. T.S. Liew & J.P. King, BOR/MOL 3666); Tandu Batu (leg. J.J. Vermeulen & M. Schilthuizen, V 9629). Segama Valley, North end of limestone ridge on East bank Tabin River (leg. J.J. Vermeulen & M. Schilthuizen, V 7783, BOR/MOL 791), Tabin Wildlife Reserve (leg. T. Kimsin & H.N. Chai, BOR/MOL 800; leg. M. Schilthuizen, BOR/MOL 822). *Tawau Province*. Batu Baturong c. 50 km W.S.W. of Lahad Datu (leg. J.J. Vermeulen & H. Duistermaat, V 1859). Segama valley, hill N.W. of crossing road Sandakan-Lahad Datu with the Segama River (leg. J.J. Vermeulen & H. Duistermaat, V 1682); Danum Valley (leg. M. Schilthuizen, BOR/MOL 797, BOR/MOL 798, BOR/MOL 811; leg. H.A. Rutjes, BOR/MOL 3618, BOR/MOL 792; leg. UMS students, BOR/MOL 787; leg. T. Kimsin & H.N. Chai, BOR/MOL 799); ‘Kirk’s Cave’ 8 km North of Lahad Datu (leg. J.J. Vermeulen, V 1241); limestone hill on North bank Segama River, near bridge of road Sandakan to Lahad Datu (leg. J.J. Vermeulen, V 7514); Sabahmas Cave (leg. J.J. Vermeulen, V 7466, BOR/MOL 793). Tawau Hills Park (leg. J.P. King, BOR/MOL 2362). *West Coast Province*. Kinabalu N.P., Headquarters area near entrance (leg. J.J. Vermeulen, V 9774); Poring Hot Spring (leg. M. Schilthuizen & P. Koomen, BOR/MOL 789, BOR/MOL 790).

####### Description.

Shell (very) small, thin, slightly translucent or more or less opaque, pale (yellowish) brown, conical with flat or slightly convex sides; apex rounded. Surface shiny. *Whorls* moderately convex, the last whorl angular at the periphery, rounded above and below the periphery. *Protoconch* whorls convex, with fine, densely but slightly irregularly placed radial riblets; spiral sculpture about equally strong, fine, slightly more spaced grooves which are partly continuous, and partly broken up into minute pits. *Teleoconch*: Last whorl with or without a peripheral spiral thread coinciding with the suture of the penultimate whorl, above the periphery with numerous (rather) fine, well-spaced grooves which consist of rows of densely placed pits which are often elongated in the direction of the growth lines; below the periphery usually with numerous fine, well-spaced, continuous grooves. Radial sculpture: above the periphery with some irregularly spaced, somewhat raised growth lines, these less conspicuous below the periphery. *Umbilicus* open, very narrow. *Dimensions*: Height up to 6 mm; width up to 5.4 mm; diameters of the first four whorls 0.7–1.0 mm, 1.15–1.60 mm, 1.7–2.2 mm, 2.1–3.2 mm respectively; number of whorls up to 7 7/8, height aperture up to 2.1 mm; width aperture up to 3 mm.

####### Habitat in Sabah and distribution.

Primary and secondary forest on limestone and granodiorite soil, up to 1700 m alt. **Sabah: Mount Kinabalu; Interior; East coast.** Also in Sarawak; Kalimantan. Distribution elsewhere: Sulawesi.

####### Cross diagnosis.

Uniquely identified by the pitted spiral sculpture on the teleoconch, in combination with its size. In *Kaliella dendrophila*, of group 1, the spiral sculpture consist of beads on the shell surface, not pits in the shell surface. It is much smaller, has a thinner, more translucent shell with a smaller protoconch with predominant spiral sculpture, and with whorls more rapidly increasing in size.

####### Remarks.

The spiral sculpture above the periphery is fine in some specimens, somewhat coarser in others. The continuous spiral striation below the periphery is absent in some specimens.

####### Etymology.

Refers to the shell sculpture [*punctatus* (L.) = dotted].

###### Kaliella calculosa

Taxon classificationAnimaliaStylommatophoraEuconulidae

(Gould, 1852)

Figure 65 , 68

Helix calculosa Gould, 1852: 48. *Coneuplecta calculosa* (Gould) H. Burrington Baker, 1941: 234; Solem 1988: 544. *Kaliella calculosa* (Gould) Schilthuizen, 2004: 94; Clements et al. 2008: 2762; Liew et al. 2010: Appendix S1 in online Supporting Information; Schilthuizen et al. 2013: online supplementary data. Type from Society Islands, Tahiti.
Sitala demissa E.A. Smith 1895: 110. Type from Malaysia, Sarawak, Mount Mulu and Busau.
Coneuplecta sitaliformis auct. Schilthuizen et al. 2002: 256.

####### Examined material from Sabah.

*Interior Province*. Upper Padas valley, Long Pasia (leg. T.S. Liew & Meckson, BOR/MOL 4357). Crocker Range N.P., West of the km 10 marker on the road Tambunan-Ranau, Mahua Waterfall (leg. J.J. Vermeulen & M. Schilthuizen, V 9736; leg. M. Schilthuizen, BOR/MOL 814). Gunung Trusmadi slopes, Gua Loloposon (leg. J.J. Vermeulen, V 13234). Pun Batu c. 30 km West of Sepulut (leg. J.J. Vermeulen, V 1282). Sepulut valley, Batu Punggul (leg. J.J. Vermeulen, V 1985); Batu Temurung (leg. J.J. Vermeulen, V 8050); Gua Pungiton (leg. J.J. Vermeulen & M. Schilthuizen, V 7560). *Sandakan Province*. Kinabatangan valley, Gomantong (leg. A. Van Til, BOR/MOL 3269); Batu Keruak (leg. M. Salverda & H. van Oosten, BOR/MOL 2358); Batu Materis (leg. T.S. Liew & B. Elahan, BOR/MOL 2121); Batu Tomanggong Besar (leg. T.S. Liew & B. Elahan, BOR/MOL 2258; leg. M. Schilthuizen, A. van Til & B. Elahan, BOR/MOL 2972); Batu Tomanggong Kecil (leg. T.S. Liew & B. Elahan, BOR/MOL 2058); Unnamed hill near Sukau Police Station (leg. T.S. Liew & B. Elahan, BOR/MOL 2225). *Tawau Province*. Batu Baturong c. 50 km W.S.W. of Lahad Datu (leg. J.J. Vermeulen & H. Duistermaat, V 14247); Danum Valley (leg. H.A. Rutjes, BOR/MOL 813, BOR/MOL 815; leg. UMS students, BOR/MOL 818). Segama valley, ‘Kirk’s Cave’ 8 km North of Lahad Datu (leg. J.J. Vermeulen, V 14248). *West Coast Province*. Kinabalu N.P., Poring Hot Springs, Garden near hot springs (leg. J.J. Vermeulen, V 13023); Orchid Garden (leg. J.J. Vermeulen, V 13025); Serinsim (leg. M. Schilthuizen, BOR/MOL 3076); Mesilau trail at 2112 m (leg. T.S. Liew, BOR/MOL 6042). Kota Kinabalu, Kiansom (leg. UMS students, BOR/MOL 816), Papar, Kg. Langsat (leg. T.S. Liew, M. Schilthuizen & F. Disuk, BOR/MOL 3095).

####### Description.

Shell small, very thin, translucent, pale yellowish-green to pale yellowish-brown, conical with about flat sides. Surface with a silky luster, shiny below the periphery. *Whorls* somewhat convex, last whorl almost rounded to angular at the periphery, moderately convex below the periphery. *Protoconch* sculpture: distinct, fine, densely placed radial riblets; spiral sculpture slightly subordinate, equally densely placed or slightly wider spaced grooves cutting into the crests of the radial riblets. *Teleoconch*: Last whorl with or without a thin peripheral spiral thread coinciding with the suture of the penultimate whorl, above and below this with very fine to rather coarse, moderately to widely spaced, continuous, sometimes rather shallow and wide grooves cutting through the radial riblets. Radial sculpture teleoconch subordinate: above the periphery inconspicuous growth lines, next to these very fine, densely and regularly placed riblets, sometimes on the outer whorls locally interrupted by approximately smooth areas; below the periphery with occasional growth lines only. *Umbilicus* open, very narrow. *Dimensions*: Height up to 5.0 mm; width up to 4.6 mm; diameters of the first four whorls 0.65–0.75 mm, 1.15–1.25 mm, 1.75–2.10 mm, 2.6–3.2 mm respectively; number of whorls up to c. 5 3/4; height aperture up to 2.5 mm; width aperture up to 2.7 mm.

**Figure 68–69.**
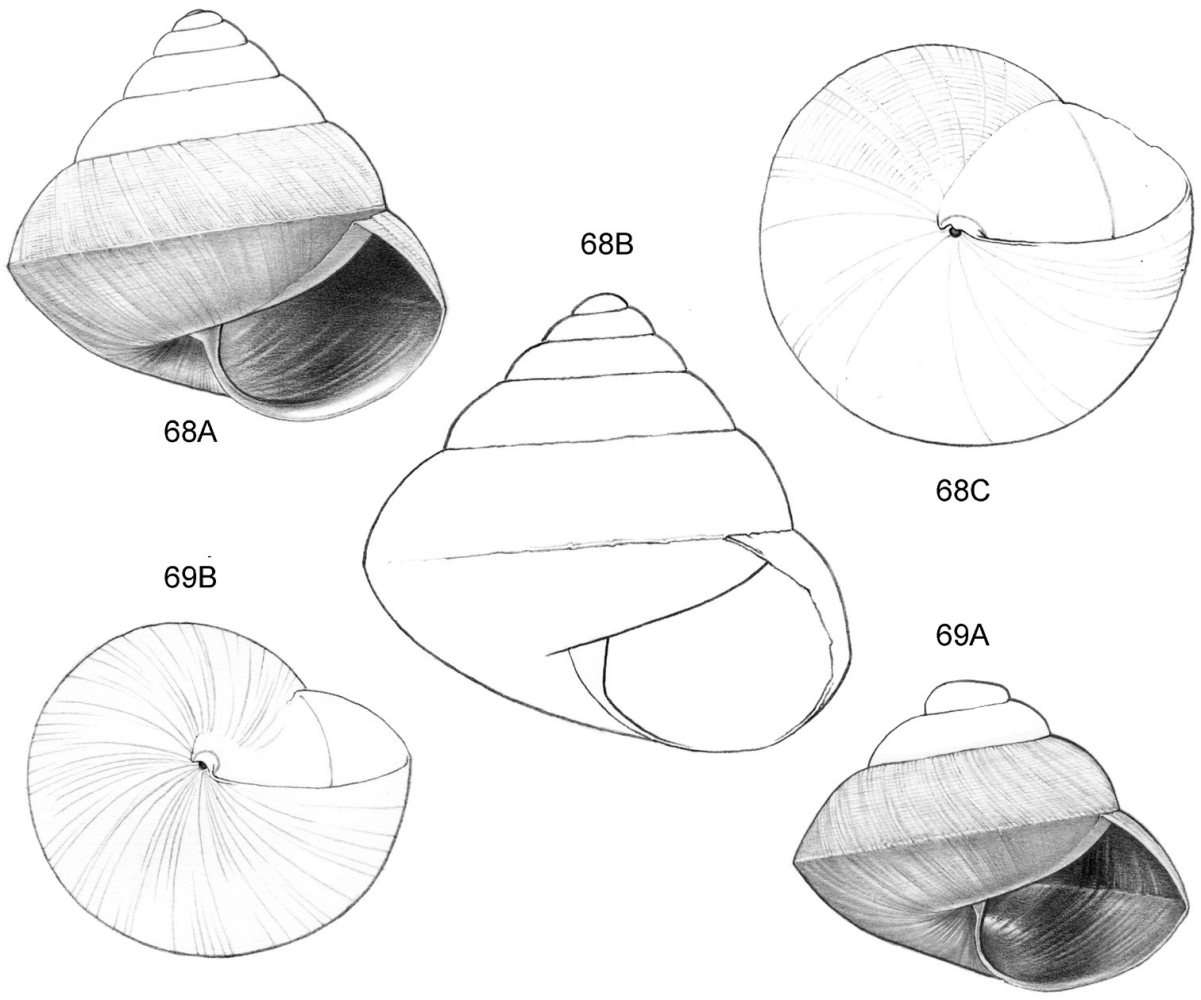
**68** *Kaliella calculosa* (Gould, 1852). **68A** Frontal view, subadult, shell 3,6 mm high **68B** Frontal view, shell 4.0 mm high **68C** Umbilical view, subadult (**68A, 68C** Malaysia, Sarawak, Gunung Mulu N.P., Gunung Api, path from camp to pinnacles, incl. pinnacle area, V 11408 **68B** Malaysia, Sarawak, Upper Penrissen valley, Mount Bra’ang, West flank, about 2 km North of Temurang village, V 13996) **69** *Kaliella microsoma*, sp. n. **69A** Frontal view, shell 1.6 mm high **69B** Umbilical view (Malaysia, West Coast Province. Crocker Range N.P., km 54 marker on the road Kota Kinabalu-Tambunan, Gunung Mas, RMNH.5003926, holotype).

####### Habitat in Sabah and distribution.

Primary and secondary forest on limestone and sandstone soil. Alt. up to 1000 m. **Sabah**: **Crocker Range; Interior; East coast; scattered localities.** Distribution elsewhere: From India, Laos, to Australia; Eastwards to Tahiti.

####### Cross diagnosis.

When fresh, shell thinner and more translucent than the other species in this group, *Kaliella dendrophila* excepted. Juveniles can be distinguished from that species by the different apical sculpture.

Elsewhere, *Kaliella sitaliformis* Von Moellendorff 1897, from Java, Bali, and Sulawesi, differs by having a raised spiral sculpture, visible in between the radial riblets, on the outer whorls.

####### Remarks.

*Kaliella calculosa* shows considerable variability in the prominence and the density of the spiral sculpture on the teleoconch. SEM-images show that the widest spiral grooves have a distinctly convex bottom; it is as if a thin thread is embedded in the groove.

###### Kaliella microsoma

Taxon classificationAnimaliaStylommatophoraEuconulidae

Vermeulen, Liew & Schilthuizen sp. n.

http://zoobank.org/EF21CB0B-C3A6-4349-BBB3-6818E94367E8

Figure 69

####### Holotype.

Malaysia, Sabah, West Coast Province, Crocker Range N.P., km 54 marker on the road Kota Kinabalu-Tambunan, Gunung Mas (RMNH.5003926).

####### Examined material from Sabah.

*Interior Province*. Upper Padas valley, Matang River South of Long Pasia (leg. J.J. Vermeulen, V 9817). *West Coast Province*. Crocker Range N.P., km 54 marker on the road Kota Kinabalu-Tambunan, Gunung Mas (leg. J.J. Vermeulen & M. Schilthuizen, V 9767).

####### Description.

Shell very small, very thin, somewhat translucent, greenish-brown, conical with slightly convex sides; apex rounded. Surface with a silky luster. Top whorls convex, outer whorls only a little less convex, last whorl angular at the periphery, somewhat obtusely so near the aperture in adult specimens, moderately convex below the periphery. *Protoconch* whorls convex, with very fine, densely placed radial riblets; spiral sculpture subordinate and sometimes inconspicous, very densely placed grooves, just visible at 40 times magnification. *Teleoconch*: Last whorl with a peripheral spiral thread coinciding with the suture of the penultimate whorl, above the periphery fine, moderately spaced, narrow spiral grooves that cut into the crests of the radial riblets and that are slightly to distinctly subordinate to these; below the periphery with similar, but slightly more distinct and slightly more spaced grooves. All spiral sculpture varies from rather distinct to inconspicuous and only locally present. Radial sculpture teleoconch: above the periphery rather inconspicuous, irregularly spaced, somewhat raised growth lines, next to these fine, very densely and regularly placed riblets on the inner whorls, less densely placed riblets locally present on the outer whorls; radial sculpture less conspicuous below the periphery. *Umbilicus* closed. *Dimensions*: Height up to 1.6 mm; width up to 1.8 mm; diameters of the first three whorls 0.45–0.55 mm, 1.0–1.2 mm, 1.65–1.90 mm respectively; number of whorls up to c. 3 3/8; height aperture up to 0.85 mm; width aperture up to 1.0 mm.

####### Habitat in Sabah and distribution.

Primary forest on sandstone soil. Alt. 1100–1700 m. **Sabah**: **upper Padas River valley; Crocker Range.** Endemic to Sabah.

####### Cross diagnosis.

General shape as in *Kaliella gregaria*, but much smaller (check the diameters of the first three whorls), slightly higher conical and with more convex whorls. The spiral sculpture is generally coarser then in *Kaliella gregaria*, but may be very inconspicuous in specimens from the Crocker Range (including the illustrated shell which, on the lower surface, has spiral sculpture only near the aperture).

####### Etymology.

The name refers to the small size [*mikros* (Gr.) = small; *soma* (Gr.) = body].

##### Group 2c. Periphery of last whorl angular in adult shells, or with a peripheral spiral thread. Diameter of the third whorl 2.0 mm or less. Spiral sculpture teleoconch absent or inconspicuously present in patches, only just discernible at 40 times magnification.

###### Kaliella barrakporensis

Taxon classificationAnimaliaStylommatophoraEuconulidae

(Pfeiffer, 1852)

Figure 70

Helix barrakporensis Pfeiffer, 1852: 156. *Kaliella barrakporensis* (Pfeiffer) Godwin Austen, 1882 (1882–1888): 2; Schilthuizen 2004: 94; Clements et al. 2008: 2762; Liew et al. 2010: Appendix S1 in online Supporting Information; Schilthuizen et al. 2013: online supplementary data. Type from: India, Barrakpore.
Sitala rumbangensis E.A. Smith, 1895: 110. Type from Malaysia, Sarawak, Rumbang and Mount Rabong.
Sitala cara E.A. Smith, 1895: 111. Type from Malaysia, Sabah, Gomantong hill.
Kaliella angigyra Von Moellendorff, 1897: 60. *Liardetia angigyra angigyra* (Von Moellendorff) Van Benthem Jutting, 1950: 398; Schilthuizen and Rutjes 2001: 420; Schilthuizen et al., 2002: 256–258. Type from Indonesia, Java).

####### Examined material from Sabah.

*Interior Province*. Crocker Range N.P., Gua Laing c. 12 km North of Keningau (leg. J.J. Vermeulen, V 1129; leg. M. Schilthuizen, BOR/MOL 836); Mahua Waterfall (leg. M. Schilthuizen, BOR/MOL 3142). Gunung Trusmadi slopes, Gua Loloposon (leg. J.J. Vermeulen, V 13238; leg. M. Schilthuizen & P. Koomen, BOR/MOL 833); Gua Dawaras (leg. M. Schilthuizen, V 9874; leg. M. Schilthuizen & M. Suleiman, BOR/MOL 2363). Pinangah valley, Batu Urun (= Bukit Sinobang) (leg. J.J. Vermeulen, V 1169, BOR/MOL 844; leg. M. Schilthuizen, BOR/MOL 847). Pun Batu c. 30 km West of Sepulut (leg. J.J. Vermeulen, V 1278). Sepulut valley, Batu Punggul (leg. J.J. Vermeulen, V 1989; leg. T.S. Liew, M. Schilthuizen & S. Chiba, BOR/MOL 4326; leg. M. Schilthuizen, BOR/MOL 851); Batu Sanaron (leg. M. Schilthuizen, BOR/MOL 850, BOR/MOL 852; leg. T.S. Liew, M. Schilthuizen & S. Chiba, BOR/MOL 4313); Batu Temurung (leg. J.J. Vermeulen, V 8051, BOR/MOL 831; leg. M. Schilthuizen, BOR/MOL 846); Bukit Tinagas, East end of Batu Punggul limestone (leg. J.J. Vermeulen & M. Schilthuizen, V 7633; leg. M. Schilthuizen, BOR/MOL 848); Gua Pungiton (leg. J.J. Vermeulen & M. Schilthuizen, V 7559, BOR/MOL 843; leg. M. Schilthuizen, BOR/MOL 845). *Sandakan Province*. Kinabatangan valley, Batu Tai (not Bod Tai) near Gomantong (leg. J.J. Vermeulen & M. Schilthuizen, V 9925; leg. T.S. Liew & B. Elahan, BOR/MOL 1941); Batu Materis (leg. T.S. Liew & B. Elahan, BOR/MOL 2088, BOR/MOL 2120); Batu Mawas (leg. M. Schilthuizen, BOR/MOL 2384; leg. T.S. Liew & M. Schilthuizen, BOR/MOL 1960, BOR/MOL 1996); Batu Tomanggong Besar (leg. T. S. Liew & Badul, V 13528; leg. M. Schilthuizen, BOR/MOL 2415); Batu Tomanggong Kecil (leg. T.S. Liew & B. Elahan, BOR/MOL 2030); Batu Tulug (Batu Putih) along road Lahad Datu-Sandakan, North of bridge over Kinabatangan River (leg. J.J. Vermeulen & H. Duistermaat, V 1487); Gomantong Hill 30 km South of Sandakan (leg. J.J. Vermeulen & H. Duistermaat, V 1619; leg. J.P. King, BOR/MOL 2417; leg. A. van Til, BOR/MOL 3305; leg. T.S. Liew & J.P. King, BOR/MOL 3659); Unnamed hill near Sukau Police Station (leg. T.S. Liew & B. Elahan, BOR/MOL 2226). Pinangah valley, Batu Melikop (leg. R. Kiew, V 7724). *Tawau Province*. Batu Baturong c. 50 km W.S.W. of Lahad Datu (leg. J.J. Vermeulen & H. Duistermaat, V 1861). Gua Madai c. 40 km S.S.W. of Lahad Datu (leg. J.J. Vermeulen & H. Duistermaat, V 1732). Segama valley, hill N.W. of crossing road Sandakan-Lahad Datu with the Segama River (leg. J.J. Vermeulen & H. Duistermaat, V 1681); ‘Kirk’s Cave’ 8 km North of Lahad Datu (leg. J.J. Vermeulen, V 1229); limestone hill on North bank Segama River, near bridge of road Sandakan to Lahad Datu (leg. J.J. Vermeulen, V 7513); Danum Valley (leg. M. Schilthuizen, BOR/MOL 810, BOR/MOL 1230, BOR/MOL 829; leg. H.A. Rutjes, BOR/MOL 830); Tabin Wildlife Reserve (leg. M. Schilthuizen, BOR/MOL 840); Sabahmas Cave (leg. J.J. Vermeulen, V 7468, BOR/MOL 841). Semporna area, Segarong Hills, Batu Tengar, 25 km E.S.E. of Kunak (leg. J.J. Vermeulen & H. Duistermaat, V 1791). *West Coast Province*. Kinabalu N.P., Headquarters area near entrance (leg. J.J. Vermeulen, V 9771, BOR/MOL 2416); Summit trail at 1723 m (leg. T.S. Liew, BOR/MOL 6041); Poring Hot Springs, along path to waterfall (leg. J.J. Vermeulen, V 13014, leg. M. Schilthuizen & P. Koomen, BOR/MOL 832); Serinsim (leg. M. Schilthuizen, BOR/MOL 3077). Kota Kinabalu, Kiansom (leg. UMS students, BOR/MOL 820; leg. M. Schilthuizen, BOR/MOL 839, BOR/MOL 865; leg. UMS Tropical Malacology Course participants, BOR/MOL 3470, BOR/MOL 3477); Papar, Kg. Langsat (leg. T.S. Liew, M. Schilthuizen & F. Disuk, BOR/MOL 3097).

####### Description.

Shell small, rather thin, somewhat translucent or opaque, (pale) brown-corneous, (somewhat elongated) conical with almost flat or slightly convex sides; apex narrowly rounded. Surface shiny or with a silky luster. *Whorls* moderately convex, the last whorl rounded to angular at the periphery, rounded above and below the periphery. *Protoconch* whorls convex, with spaced, inconspicuous radial riblets and usually subordinate, very fine spiral grooves; protoconch sometimes almost smooth. *Teleoconch*: Last whorl usually with a peripheral spiral thread coinciding with the suture of the penultimate whorl, above this with very fine, densely and regularly placed radial riblets, often only locally present, in some specimens virtually absent, and with or without traces of very fine (just visible at 40 times magnification), inconspicuous spiral striation; below the periphery usually with numerous fine, moderately and regularly spaced, continuous spiral grooves. *Umbilicus* open, narrow. *Dimensions*: Height up to 4.6 mm; width up to 4.3 mm; diameters of the first four whorls 0.65–0.80 mm, 1.10–1.25 mm, 1.5–2.0 mm, 2.15–2.70 mm respectively; number of whorls up to 6 1/2; height aperture up to 1.5 mm; width aperture up to 2.0 mm.

**Figure 70–71.**
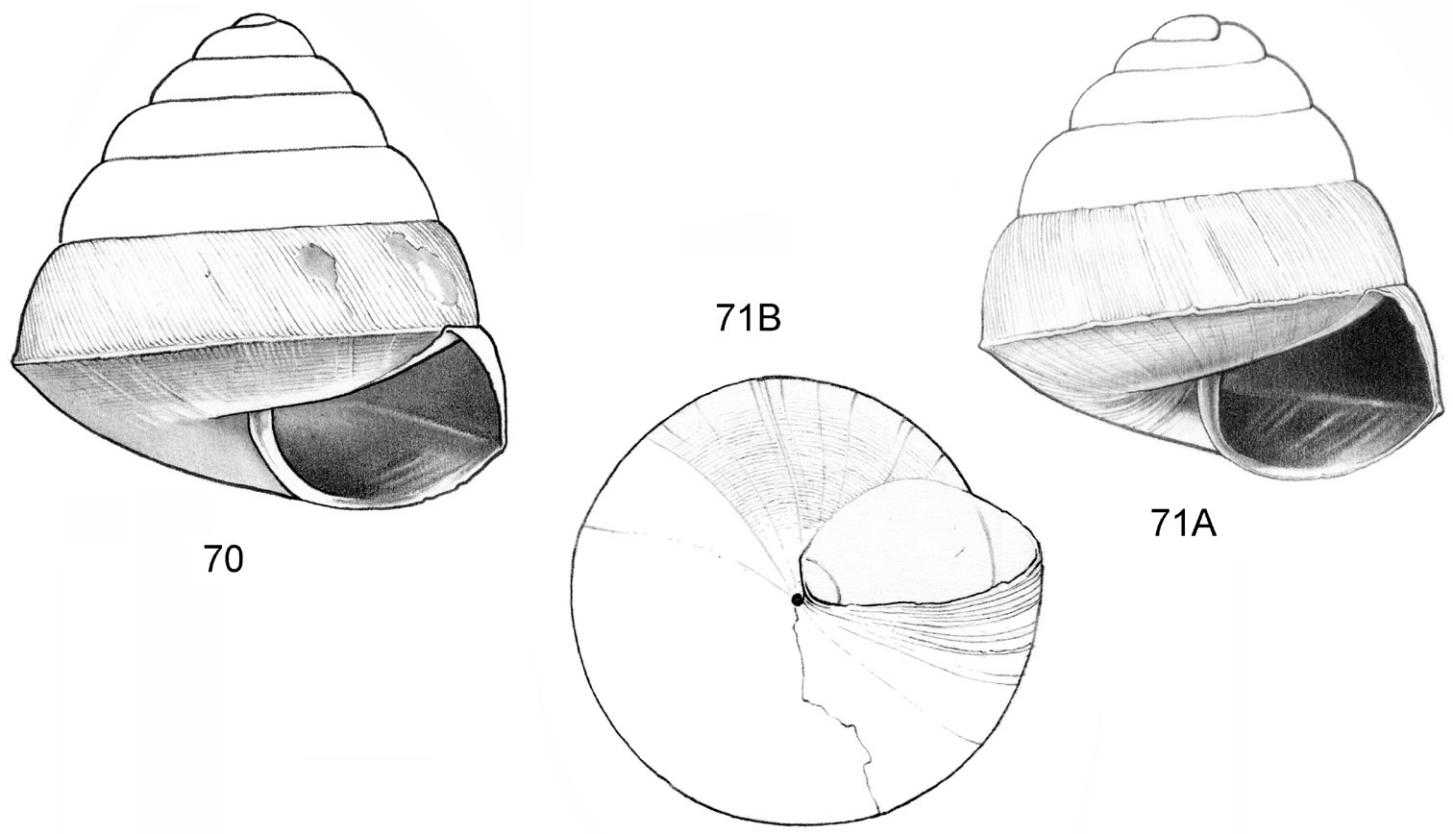
**70** *Kaliella barrakporensis* (Pfeiffer, 1852). Frontal view, shell 3.4 mm high (Indonesia, Bali, Mount Abang, V 3505) **71** *Kaliella accepta* (E.A. Smith, 1895). **71A** Frontal view, shell 2.5 mm high **71B** Umbilical view (Malaysia, Sabah, Sandakan Province, Kinabatangan river valley, Batu Pangi, V 9657).

####### Habitat in Sabah and distribution.

Primary and secondary forest, rocky, vegetated roadsides, on limestone, sandstone, and granodiorite soil, up to 2200 m alt. **Sabah: widespread.** Also in Sarawak; Kalimantan. Distribution elsewhere: Africa. India; Thailand; Laos; Peninsular Malaysia to Java; Eastwards to Sulawesi, Flores.

####### Remarks.

Incidental specimens lack the spiral thread along the periphery of the last whorl. A fine but almost continuous spiral striation may be present on the top whorls.

###### Kaliella accepta

Taxon classificationAnimaliaStylommatophoraEuconulidae

(E.A. Smith, 1895)

Figure 53 , 71

Sitala accepta E.A. Smith, 1895: 111. *Kaliella accepta* (E.A. Smith) Schilthuizen, 2004: 94; Clements et al. 2008: 2762; Schilthuizen et al. 2013: online supplementary data. Type from Malaysia, Sabah, Gomantong Hill.

####### Examined material from Sabah.

*Interior Province*. Crocker Range N.P., West of the km 10 marker on the road Tambunan-Ranau, Mahua Waterfall (leg. J.J. Vermeulen & M. Schilthuizen, V 9746; leg. M. Schilthuizen, BOR/MOL 2386); Gunung Emas (leg. M. Schilthuizen, BOR/MOL 880); Ulu Kimanis at 750 m (leg. UMS students, BOR/MOL 2356). Meliau Range, east of Solonsong River at 650 m (leg. T.S. Liew, M. Schilthuizen & A. van Til, BOR/MOL 3206). Pinangah valley, Batu Urun (= Bukit Sinobang) (leg. J.J. Vermeulen, V 8003, V 8004). Sepulut valley, Batu Temurung (leg. J.J. Vermeulen, V 9924); Bukit Tinagas, East end of Batu Punggul limestone (leg. J.J. Vermeulen & M. Schilthuizen, V 7710); Gua Sanaron (leg. J.J. Vermeulen & M. Schilthuizen, V 7667). *Sandakan Province*. Kinabatangan valley, Batu Batangan (leg. M. Schilthuizen, BOR/MOL 2374); Gomantong cave (leg. A. van Til, BOR/MOL 3324, BOR/MOL 3337; leg. M. Schilthuizen, BOR/MOL 876, leg. T.S. Liew & J.P. King, BOR/MOL 3658); Batu Keruak 2 near Sukau (leg. J.J. Vermeulen & M. Schilthuizen, V 9797, BOR/MOL 2375; leg. M. Salverda & H. van Oosten, BOR/MOL 2371; leg. T.S. Liew & B. Elahan, BOR/MOL 1853, BOR/MOL 1887); Batu Mawas (leg. T.S. Liew & M. Schilthuizen, BOR/MOL 1995); Batu Materis (leg. M. Schilthuizen, BOR/MOL 2372, BOR/MOL 2397; leg. T.S. Liew & B. Elahan, BOR/MOL 2087, BOR/MOL 2117, V 13463); Batu Pangi (leg. J.J. Vermeulen & M. Schilthuizen, V 9657, BOR/MOL 2377); Batu Tai (not Bod Tai) near Gomantong (leg. J.J. Vermeulen & M. Schilthuizen, V 9592, BOR/MOL 2369; leg. T.S. Liew & B. Elahan, BOR/MOL 1917; leg. M. Schilthuizen, BOR/MOL 2378, BOR/MOL 2381); Hill Sg. Resang (leg. M. Schilthuizen, BOR/MOL 2382, BOR/MOL 2385); Batu Tomanggong Kecil (leg. J.J. Vermeulen & M. Schilthuizen, V 9701, BOR/MOL 2376; leg. T.S. Liew & B. Elahan, BOR/MOL 2028, BOR/MOL 2057); Tandu Batu (leg. J.J. Vermeulen & M. Schilthuizen, V 9618, BOR/MOL 2370); Batu Tomanggong Besar (leg. M. Schilthuizen, BOR/MOL 874, BOR/MOL 2383, BOR/MOL 2380; leg. T.S. Liew & B. Elahan, BOR/MOL 2257, BOR/MOL 2287; leg. M. Salverda & H. van Oosten, BOR/MOL 2373); unnamed limestone hill near Sukau Police Station (leg. T. S. Liew & Badul, V 13502; leg. M. Schilthuizen, BOR/MOL 2379; leg. T.S. Liew, BOR/MOL 2157; leg. T.S. Liew & B. Elahan, BOR/MOL 2190, BOR/MOL 2224, V 13502). Segama Valley, North end of limestone ridge on East bank Tabin River (leg. J.J. Vermeulen & M. Schilthuizen, V 7785, BOR/MOL 871; leg. T. Kimsin & H.N. Chai, BOR/MOL 872). *Tawau Province*. Batu Baturong, North slope (leg. J.J. Vermeulen, V 7600). Danum Valley (leg. UMS students, BOR/MOL 828; leg. M. Schilthuizen, BOR/MOL 837, BOR/MOL 875, BOR/MOL 877, BOR/MOL 3617; leg. T. Kimsin & H.N. Chai, BOR/MOL 873; leg. H.A. Rutjes, BOR/MOL 878, BOR/MOL 879). Gua Madai c. 40 km S.S.W. of Lahad Datu, N.E. end (leg. J.J. Vermeulen, V 7708). Segama valley, ‘Kirk’s Cave’ 8 km North of Lahad Datu (leg. J.J. Vermeulen, V 1234); Sabahmas Cave (leg. J.J. Vermeulen, V 7830). Semporna area, Segarong Hills, Bukit Pababola, 25 km E.S.E. of Kunak (leg. J.J. Vermeulen & H. Duistermaat, V 1774); Bod Gaya Island (leg. M. Schilthuizen & A.S. Cabanban, BOR/MOL 3547; leg. T.S. Liew, BOR/MOL 4673; leg. T.S. Liew, Abdul & Ladja, BOR/MOL 4753, BOR/MOL 4780, BOR/MOL 4791, BOR/MOL 4807, BOR/MOL 4846, BOR/MOL 4880). Tawau Hills N.P., path up to Bukit Bombalai (leg. J.J. Vermeulen, V 13154). *West Coast Province*. Crocker Range N.P., km 54 marker on the road Kota Kinabalu-Tambunan, Gunung Mas (leg. J.J. Vermeulen & M. Schilthuizen, V 9765). Kinabalu N.P., Poring Hot Springs, along path to waterfall (leg. J.J. Vermeulen, V 13013); Serinsim (leg. M. Schilthuizen, BOR/MOL 3065).

####### Description.

Shell small, rather thin, somewhat translucent or opaque, (pale) yellow-corneous to brown-corneous, (somewhat depressed) conical with convex sides; apex broadly rounded. Surface glossy or shiny. *Whorls* moderately convex, the last whorl more or less angular at the periphery, rounded above, rounded to almost flat below the periphery. *Protoconch* whorls convex, with spaced radial riblets and usually subordinate (sometimes equally distinct), very fine spiral grooves; usually these are reduced to spiral rows of minute, shallow identations; often the protoconch is almost smooth. *Teleoconch*: Last whorl with a peripheral spiral thread coinciding with the suture of the penultimate whorl, above this with very fine, inconspicuous, densely and regularly placed radial riblets locally, or with a few growth lines only; with or without traces of very fine, inconspicuous spiral striation, below the periphery usually with numerous fine, moderately and regularly spaced, continuous spiral grooves which are most distinct towards the periphery. *Umbilicus* open, narrow. *Dimensions*: Height up to 2.8 mm; width up to 3.2 mm; diameters of the first four whorls 0.5–0.8 mm, 0.90–1.35 mm, 1.5–2.0 mm, 2.0–2.8 mm respectively; number of whorls up to 5; height aperture up to 1.2 mm; width aperture up to 1.6 mm.

####### Habitat in Sabah and distribution.

Primary and secondary forest on limestone, sandstone and volcanic soil, up to 1400 m alt. **Sabah: widespread.** Also in Kalimantan. Endemic to Borneo.

####### Cross diagnosis.

Adult or nearly adult shells differ from *Kaliella barrakporensis* by the lower conical spire with more convex sides and a more broadly rounded apex. Usually, the sculpture is less distinct than in *Kaliella barrakporensis*, and the shells are glossier. Juveniles of both species are sometimes difficult to keep apart.

##### Group 2d. Periphery of last whorl rounded in adult shells (rounded to angular in juveniles), without peripheral thread, (check also *Kaliella calculosa*; occasional specimens of *Kaliella barrakporensis* may lack the peripheral thread).

###### Kaliella scandens

Taxon classificationAnimaliaStylommatophoraEuconulidae

(Cox, 1871)

Figure 72

Helix scandens Cox, 1871: 645. *Liardetia scandens* (Cox) Solem, 1988: 550. *Kaliella scandens* (Cox) Schilthuizen et al., 2002: 256; Schilthuizen 2004: 94; Clements et al. 2008: 2762; Schilthuizen et al. 2011: 5; Schilthuizen et al. 2013: online supplementary data. *Liardetia scandens* (Cox) Schilthuizen et al. 2002b: 37, 41–42; Schilthuizen and Vermeulen 2003: 96. Type from Australia, New South Wales, Port Macquarie.
Kaliella indifferens Boettger, 1891: 256. *Liardetia indifferens* (Boettger) Van Benthem Jutting, 1950: 408. Type from Indonesia, Ambon.
Sitala dulcis E.A. Smith, 1895: 111. Type from Malaysia, Sabah, Gomantong Hill.

####### Examined material from Sabah.

*Interior Province*. Crocker Range N.P., Gua Laing c. 12 km North of Keningau (leg. J.J. Vermeulen, V 1098; leg. M. Schilthuizen, BOR/MOL 858); West of the km 10 marker on the road Tambunan-Ranau, Mahua Waterfall (leg. J.J. Vermeulen & M. Schilthuizen, V 9748; leg. J. Schilthuizen, BOR/MOL 870). Gunung Trusmadi slopes, Gua Loloposon (leg. J.J. Vermeulen, V 13239). Pinangah valley, Batu Urun (= Bukit Sinobang) (leg. J.J. Vermeulen, V 1155; leg. M. Schilthuizen, BOR/MOL 868). Pun Batu c. 30 km West of Sepulut (leg. J.J. Vermeulen, V 1283). Sepulut valley, Batu Punggul (leg. J.J. Vermeulen, V 1983; leg. M. Schilthuizen, BOR/MOL 867); Bukit Tinagas, East end of Batu Punggul limestone (leg. J.J. Vermeulen & M. Schilthuizen, V 7634); Gua Pungiton (leg. J.J. Vermeulen & M. Schilthuizen, V 7557). *Kudat Province*. Kampong Magnin (leg. T.S. Liew & M. Schilthuizen, BOR/MOL 4362); Balambangan Island, Kok Simpul (leg. J.J. Vermeulen & M. Schilthuizen, V 9927, BOR/MOL 2396); South end, Batu Sireh (leg. J.J. Vermeulen & M. Schilthuizen, V 9549; leg. T.H. Liew, Sazilin & Ramlan, BOR/MOL 3697). Banggi Island, South end (leg. J.J. Vermeulen, V 1430); Karakit Hill (leg. J.J. Vermeulen, V 1463). *Sandakan Province*. Kinabatangan valley, Batu Batangan (leg. M. Schilthuizen, BOR/MOL 2410); Batu Keruak 2 near Sukau (leg. J.J. Vermeulen & M. Schilthuizen, V 9799, BOR/MOL 2405; leg. M. Salverda & H. van Oosten, BOR/MOL 2398, BOR/MOL 2402); Batu Mawas (leg. M. Schilthuizen, BOR/MOL 2407; leg. T.S. Liew & M. Schilthuizen, BOR/MOL 1962, BOR/MOL 1998); Batu Materis (leg. T.S. Liew & B. Elahan, BOR/MOL 2118); Batu Pangi (leg. J.J. Vermeulen & M. Schilthuizen, V 9663); Batu Tai (not Bod Tai) near Gomantong (leg. J.J. Vermeulen & M. Schilthuizen, V 9578); Bod Tai (leg. M. Schilthuizen, BOR/MOL 2399; leg. T.S. Liew & B. Elahan, BOR/MOL 1920); Batu Tomanggong Besar (leg. M. Salverda & H. van Oosten, BOR/MOL 2400; leg. T.S. Liew & B. Elahan, BOR/MOL 2645; leg. M. Schilthuizen, BOR/MOL 2409, BOR/MOL 862); Hill on Resang River (leg. M. Schilthuizen, BOR/MOL 2408); Batu Tomanggong Kecil (leg. J.J. Vermeulen & M. Schilthuizen, V 9688, V 9926 BOR/MOL 2403; leg. T.S. Liew & B. Elahan, BOR/MOL 2031); Batu Tulug (Batu Putih) along road Lahad Datu-Sandakan, North of bridge over Kinabatangan River (leg. J.J. Vermeulen & H. Duistermaat, V 1496); Gomantong Hill 30 km South of Sandakan (leg. J.J. Vermeulen & H. Duistermaat, V 1632); Tandu Batu (leg. J.J. Vermeulen & M. Schilthuizen, V 9615); Unnamed hill near Sukau Police Station (leg. M. Schilthuizen, BOR/MOL 2404; leg. T.S. Liew, BOR/MOL 2159; leg. T.S. Liew & B. Elahan, BOR/MOL 2192). Segama Valley, North end of limestone ridge on East bank Tabin River (leg. J.J. Vermeulen & M. Schilthuizen, V 7784, BOR/MOL 859; leg. T. Kimsin & H.N. Chai, BOR/MOL 854; leg. J.J. Vermeulen, BOR/MOL 855; leg. M. Schilthuizen, BOR/MOL 856). *Tawau Province*. Batu Baturong c. 50 km W.S.W. of Lahad Datu (leg. J.J. Vermeulen & H. Duistermaat, V 1864); North slope (leg. J.J. Vermeulen, V 7601). Gua Madai c. 40 km S.S.W. of Lahad Datu (leg. J.J. Vermeulen & H. Duistermaat, V 1729; leg. M. Schilthuizen & A.S. Cabanban, BOR/MOL 3575); N.E. end (leg. J.J. Vermeulen, V 7707). Segama valley, hill N.W. of crossing road Sandakan-Lahad Datu with the Segama River (leg. J.J. Vermeulen & H. Duistermaat, V 1689); ‘Kirk’s Cave’ 8 km North of Lahad Datu (leg. J.J. Vermeulen, V 1225); limestone hill on North bank Segama River, near bridge of road Sandakan to Lahad Datu (leg. J.J. Vermeulen, V 7515); Sabahmas Cave (leg. J.J. Vermeulen, V 7467); Danum Valley (leg. M. Schilthuizen, BOR/MOL 860, BOR/MOL 864; leg. UMS students, BOR/MOL 863). Semporna area, Bukit Tenggorak (leg. M. Schilthuizen & A.S. Cabanban, BOR/MOL 3562); Bohey Dulang Island (leg. M. Schilthuizen & A.S. Cabanban, BOR/MOL 3529; leg. T.S. Liew & M. Ruf, BOR/MOL 4713); Bod Gaya Island (leg. T.S. Liew, Abdul & Ladja, BOR/MOL 4725, BOR/MOL 4808, BOR/MOL 4821,/MOL 4939); Selakan Island (leg. T.S. Liew, BOR/MOL 5333; leg. T.S. Liew, BOR/MOL 5344). *West Coast Province*. Kinabalu N.P., Poring Hot Springs, along path to waterfall (leg. J.J. Vermeulen, V 13016; leg. M. Schilthuizen & P. Koomen, BOR/MOL 869); Orchid Garden (leg. J.J. Vermeulen, V 13027). Mantanani Group, Pulau Mantanani Besar (leg. M. Schilthuizen, V 9840; leg. T.H. Liew, BOR/MOL 3710); Pulau Mantanani Kecil (leg. M. Schilthuizen, V 9855; leg. T.H. Liew, BOR/MOL 3737). Kota Kinabalu, Kiansom (leg. UMS Tropical Malacology Course participants, BOR/MOL 3469).

####### Description.

Shell small, rather thin, somewhat translucent, (pale) brown, sometimes white, rather low-conical with flat to slightly convex sides; apex rounded. Surface with a silky luster. *Whorls* moderately convex, last whorl rounded at the periphery (somewhat angular in juveniles), rounded above and below the periphery. *Protoconch* whorls convex, with numerous fine, rather densely placed radial riblets; spiral sculpture subordinate or approx. equally distinct, very fine (just visible at 40 times magnification), densely placed grooves. *Teleoconch*: above the periphery with fine, slightly spaced, narrow spiral grooves cutting into the crests of the radial riblets and subordinate to these; below the periphery slightly coarser and slightly more spaced spiral grooves. Radial sculpture: above the periphery fine, densely and regularly placed riblets; below the periphery with a few irregularly spaced, inconspicuous growth lines only. *Umbilicus* open, very narrow. *Dimensions*: Height up to 2.8 mm; width up to 3.65 mm; diameters of the first four whorls 0.6–0.7 mm, 1.05–1.30 mm, 1.6–1.9(-2.0) mm, 2.25–2.70(-3.0 mm) respectively; number of whorls up to c. 5 1/2; height aperture up to 0.7 mm; width aperture up to 1.2 mm. Radula: central 3-cuspid; laterals 3-cuspid; marginals 3–5 cuspid.

**Figure 72–74.**
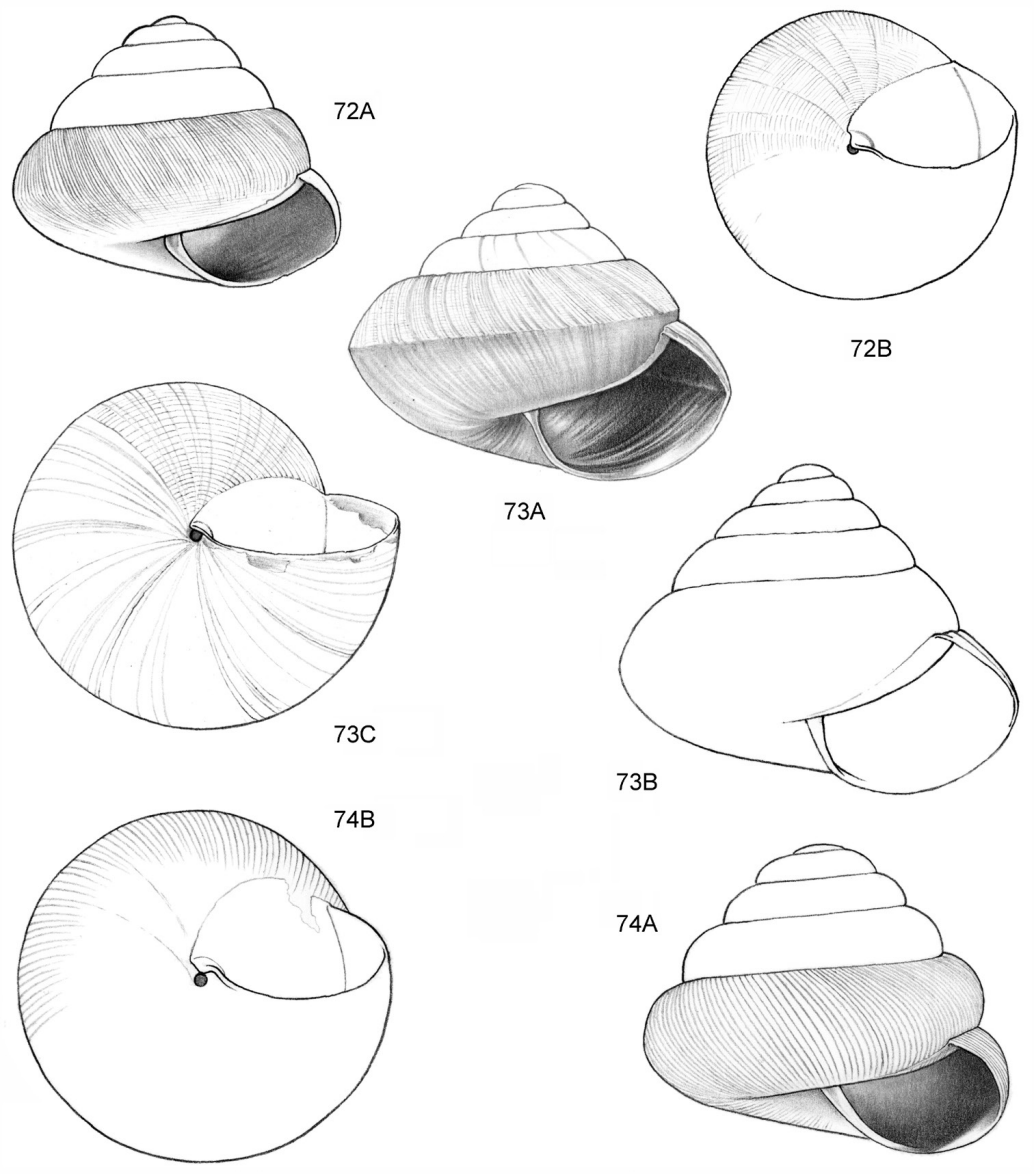
**72** *Kaliella scandens* (Cox, 1871) **72A** Frontal view, shell 2.2 mm high **72B** Umbilical view (Indonesia, Java, Nusa Kembangan, V 3581) **73** *Kaliella dendrobates* (Tillier & Bouchet, 1988). **73A** Frontal view, subadult, shell 2.6 mm high **73B** Frontal view, shell 3.2 mm high **73C** Umbilical view, subadult (**73A, 73C** Malaysia, Sabah, Mount Kinabalu, V 13464 **73B** Malaysia, Mount Kinabalu, after Tillier and Bouchet 1988) **74** *Kaliella doliolum* (Pfeiffer, 1846). **74A** Frontal view, shell 2.5 mm high **74B** Umbilical view (Indonesia, Java, Padalarang Hills, V 3886).

####### Habitat in Sabah and distribution.

Primary and secondary forest, coastal forest, at 0–1000 m alt, elsewhere to 2000 m alt. **Sabah: widespread**. Also in Sarawak; Kalimantan. Distribution elsewhere: Vietnam; Thailand; Peninsular Malaysia to Java; Eastwards to Australia, Pacific.

####### Remarks.

Samples from montane environments (e.g. Poring Hot Springs, V 13027 may include shells with a more loosely coiled spire than usual (diameter of the fourth whorl up to 3.0 mm; in other specimens 2.25–2.70 mm). In this character the shells approach *Kaliella dendrobates*, but the sculpture is typical for *Kaliella scandens*.

Information on the radula is from Rensch (1932). No Sabah material included in *Kaliella scandens* has been checked for radula characters.

###### Kaliella dendrobates

Taxon classificationAnimaliaStylommatophoraEuconulidae

(Tillier & Bouchet, 1988)

Figure 73

Gunongia dendrobates Tillier & Bouchet, 1988: 269. *Kaliella dendrobates* (Tillier & Bouchet) Liew et al., 2010: Appendix S1 in online Supporting Information. Type from Malaysia, Sabah, Kinabalu N.P.

####### Examined material from Sabah.

*Interior Province*. Gunung Trusmadi, Gua Dawaras (leg. M. Schilthuizen, V 13527). *West Coast Province*. Kinabalu N.P., Carson’s Falls (leg. J.J. Vermeulen, V 12712); Gunting Lagadan (leg. Tachaini Narainan, V 13464); Mesilau trail at 2092 m (leg. T.S. Liew, BOR/MOL 6045); Kotal’s route at 2132 m (leg. T.S. Liew, J. Lapidin, Safrie & Jasilin, BOR/MOL 6044).

####### Description.

Shell small, very thin, somewhat translucent, (yellowish) brown, rather low-conical with flat sides; apex rounded. Surface shiny. *Whorls* moderately convex, last whorl rounded at the periphery (angular in juveniles), rounded above and below the periphery. *Protoconch* whorls convex, with numerous fine, densely placed radial riblets; spiral sculpture subordinate, very fine (just visible at 40 times magnification) to rather distinct, very densely placed grooves, locally present. *Teleoconch*: above the periphery locally with fine, slightly spaced, narrow spiral grooves cutting into the crests of the radial riblets and subordinate to these, particularly towards the suture and the periphery; below the periphery slightly coarser and slightly more spaced spiral grooves. Radial sculpture: above the periphery distinct, irregularly spaced and locally crowded, somewhat raised growth lines, next to these areas with fine, densely (slightly more densely than the spiral striation) and regularly placed riblets, on the outer whorls locally interrupted by approximately smooth areas; below the periphery with irregularly spaced, slightly raised growth lines only. *Umbilicus* open, very narrow. *Dimensions*: Height up to 3.2 mm; width up to 3.9 mm; diameters of the first four whorls 0.65–0.75 mm, c. 1.1 mm, 1.7–1.8 mm, c. 2.65 mm respectively; number of whorls up to c. 5 1/2; height aperture up to 1.6 mm; width aperture up to 1.9 mm. Animal with an obtuse caudal horn. Radula: central 1-cuspid; laterals and marginals similar, serrate with 2 large cones at the tip and a small in between.

####### Habitat in Sabah and distribution.

Montane and sub-alpine forest on granodiorite and sandstone soil, at 1600–3400 m alt. **Sabah: Mount Kinabalu, Gunung Trusmadi.** Endemic to Sabah.

####### Cross diagnosis.

In general shape, sculpture and mode of coiling very similar to *Kaliella scandens*. *Kaliella dendrobates* has a much thinner, more shiny shell. The sculpture above the periphery includes irregularly raised growth lines where the fine sculpture of minute radial riblets and even finer spiral grooves is indistinct or entirely absent. In *Kaliella scandens* raised growth lines are usually absent, and the finer sculpture is continuous. Further differences between the two are found in the radula.

*Kaliella micula* (Mousson, 1857) (*Zonites micula* Mousson, 1857), a widespread species which occurs in Sarawak, resembles *Kaliella dendrobates* but has a widely rounded apex and slightly convex sides. It is also less densely coiled than *Kaliella dendrobates* (diameter of the first four whorls c. 1.0 mm, 1.7 mm, 2.8 mm, 4.0 mm respectively in V 13975 from Sarawak, Kuching area, Mount Nambi).

Juveniles of *Kaliella dendrobates* with a peripheral keel differ from *Kaliella calculosa* in having a lower conical spire, and a radial sculpture dominated by rather prominent, irregularly spaced, raised growth lines.

We depict a keeled, juvenile specimen with approx. 1 whorl less than the type specimen, which does not have a keel. The sculpture and general shape is similar in both specimens.

The Gunung Trusmadi record is based on a shell consisting of hardly more than a protoconch. Its identity is somewhat uncertain.

###### Kaliella doliolum

Taxon classificationAnimaliaStylommatophoraEuconulidae

(Pfeiffer, 1846)

Figure 74

Helix doliolum Pfeiffer, 1846: 41. *Kaliella doliolum* (Pfeiffer) Von Moellendorff, 1890: 205; Clements et al. 2008: 2762; Schilthuizen et al. 2011: 5; Schilthuizen et al. 2013: 544, and online supplementary data. *Liardetia doliolum* (Pfeiffer) Van Benthem Jutting, 1950: 410. Type from Philippines, Cebu, Sibonga.
Sitala (?) orchis Godwin Austen, 1891: 40. Type from Malaysia, Sabah, Labuan.

####### Examined material from Sabah.

*Interior Province*. Sepulut valley, Batu Punggul (leg. J.J. Vermeulen, V 1982). *Kudat Province*. Balambangan Island, Kok Simpul (leg. J.J. Vermeulen & M. Schilthuizen, V 9537, BOR/MOL 2413; leg. T.H. Liew, Sazilin & Ramlan, BOR/MOL 3696). Banggi Island, South end, Karakit Hill (leg. J.J. Vermeulen & M. Schilthuizen, V 9500, BOR/MOL 2411). *Sandakan Province*. Gomantong Cave (leg. J.P. King, BOR/MOL 2412; leg. A. van Til, BOR/MOL 3277, BOR/MOL 3299; leg. T.S. Liew & J.P. King, BOR/MOL 3662). Pulau Mataking, Easternmost island of the Semporna-Sulu Chain (leg. G.W.H. Davison, V 11517). *Tawau Province*. Batu Baturong c. 50 km WSW of Lahad Datu (leg. J.J. Vermeulen & H. Duistermaat, V 1863). Semporna area, Bukit Tengkorak, 5 km South of Semporna (leg. M. Schilthuizen & A.S. Cabanban, V 13482); Segarong Hills, Bukit Pababola, 25 km E.S.E. of Kunak (leg. J.J. Vermeulen & H. Duistermaat, V 1771); Bohey Dulang Island (leg. T.S. Liew, BOR/MOL 4634); Tetagan Island (leg. T.S. Liew & Abdul, BOR/MOL 5022; leg. T.S. Liew, Abdul & Ladja, BOR/MOL 5031); Mantabuan Island (leg. T.S. Liew & Abdul, BOR/MOL 5039, BOR/MOL 5050); Sibuan Island (leg. T.S. Liew & Abdul, BOR/MOL 5062, BOR/MOL 5070); Maiga Island (leg. T.S. Liew & Abdul, BOR/MOL 5078; leg. Abdul & Ladja, BOR/MOL 5091). *West Coast Province*. Pulau Tiga in Kimanis Bay (leg. J.J. Vermeulen, V 11340; leg. UMS students, BOR/MOL 853).

####### Description.

Shell small, rather thin, slightly translucent or opaque, (pale) brown, rather low-conical with approx. flat sides; apex broadly rounded. Surface with a silky luster. Whorls (moderately) convex, last whorl rounded at the periphery (somewhat angular in juveniles), rounded above and below the periphery. *Protoconch* whorls convex, with numerous fine, densely placed radial riblets; spiral sculpture subordinate, very fine (just visible at 40 times magnification), densely placed grooves cutting into the crests of the radial riblets. *Teleoconch*: above the periphery with rather densely and somewhat irregularly placed, distinct and rather coarse radial ribs; with or without subordinate, very fine and inconspicuous, rather dense spiral striation in the interstices; below the periphery with a few irregularly spaced, inconspicuous growth lines, and with or without fine, densely placed or somewhat spaced spiral grooves. Umbilicus open, narrow. *Dimensions*: Height up to 2.9 mm; width up to 3.6 mm; diameters of the first four whorls 0.7–0.8 mm, 1.2–1.4 mm, 1.80–2.25 mm, 2.5–3.1 mm respectively; number of whorls up to c. 5 1/8; height aperture up to 0.7 mm; width aperture up to 1.2 mm.

####### Habitat in Sabah and distribution.

Primary forest, secondary forest and coastal forest on limestone bedrock and volcanic bedrock; all localities in the lowlands. **Sabah: Scattered localities along the East coast and Islands around Semporna; one locality in the interior: Sepulut valley.** Also in Sarawak. Distribution elsewhere: Vietnam; Peninsular Malaysia to Java; Eastwards to Australia, Pacific.

####### Cross diagnosis.

Shell shape similar to *Kaliella scandens*; diagnostic is the much coarser radial sculpture. If present, the spiral sculpture below the periphery is much finer.

#### Genus Rahula

Taxon classificationAnimaliaStylommatophoraEuconulidae

Godwin Austen, 1907

Rahula Godwin Austen, 1907 (1897–1914): 216.

##### Diagnosis for the Sabah species.

Teleoconch: Radial sculpture approx. orthocline, consisting of widely spaced, coarse ribs. Last whorl with 1 spiral thread near the periphery.

##### Remarks.

The genus *Rahula* is well characterized by its coarse radial ribs.

#### Rahula delopleura

Taxon classificationAnimaliaStylommatophoraEuconulidae

Vermeulen, Liew & Schilthuizen sp. n.

http://zoobank.org/975C8B6A-ACA8-4553-BFD6-E61722D11A31

Figure 75

Rahula raricostulata auct. Schilthuizen and Vermeulen 2003: 96.
Rahula sp. V9667 auct. Clements et al. 2008: 2762.

##### Holotype.

Malaysia, Sabah, Sandakan Province, Kinabatangan valley, Batu Pangi (RMNH.5003927).

##### Examined material from Sabah.

*Interior Province*. Pinangah valley, Batu Urun (= Bukit Sinobang) (leg. J.J. Vermeulen, V 8006, BOR/MOL 827). Sepulut valley, Gua Pungiton (leg. J.J. Vermeulen & M. Schilthuizen, V 7556; leg. M. Schilthuizen, BOR/MOL 824); Batu Tinagas (leg. M. Schilthuizen, BOR/MOL 826); Gua Sanaron (leg. J.J. Vermeulen & M. Schilthuizen, V 8072). *Sandakan Province*. Kinabatangan valley, Batu Pangi (leg. J.J. Vermeulen & M. Schilthuizen, V 9667, BOR/MOL 2361); Batu Materis (leg. T.S. Liew & B. Elahan, BOR/MOL 2100); Batu Tomanggong Besar (leg. T.S. Liew & B. Elahan, BOR/MOL 2266, BOR/MOL 2299; leg. M. Schilthuizen, BOR/MOL 2360); Batu Tomanggong Kecil (leg. M. Salverda & H. van Oosten, BOR/MOL 2359). Segama Valley, North end of limestone ridge on East bank Tabin River (leg. J.J. Vermeulen & M. Schilthuizen, V 7787, BOR/MOL 825)). *Tawau Province*. Batu Baturong, North slope (leg. J.J. Vermeulen, V 7597). Gua Madai c. 40 km S.S.W. of Lahad Datu, N.E. end (leg. J.J. Vermeulen, V 7706). Segama valley, hill N.W. of crossing road Sandakan-Lahad Datu with the Segama River (leg. J.J. Vermeulen & H. Duistermaat, V 1676); ‘Kirk’s Cave’ 8 km North of Lahad Datu (leg. J.J. Vermeulen, V 1224); limestone hill on North bank Segama River, near bridge of road Sandakan to Lahad Datu (leg. J.J. Vermeulen, V 7512). Semporna area, Segarong Hills, Batu Tengar, 25 km E.S.E. of Kunak (leg. J.J. Vermeulen & H. Duistermaat, V 1820).

##### Description.

Shell small, rather solid, somewhat translucent to opaque, (pale) brown, conical with convex sides to almost ovoid; apex rounded. Surface shiny. *Whorls* convex, rounded, suture somewhat imperessed. *Protoconch* whorls convex, with very fine, densely placed radial riblets starting at some distance from the apex; apex with very fine (just visible at 40 times magnification), inconspicuous spiral striation, which gradually disappears where the radial riblets become more prominent. *Teleoconch*: Last whorl with a distinct spiral ridge slightly below the periphery, which seems to be the edge of a callus covering the lower surface of the shell, and which continues just above the suture of the penultimate whorl; next to this a fine, dense spiral striation on the lower surface of the shell. Radial sculpture above the spiral ridge consisting of well-spaced (26–33 on the last whorl), coarse, orthocline, approx. straight, high and narrow ribs, which reach down to the spiral ridge and are fused to it, interstices smooth or with an occasional, inconspicuous growth line. *Umbilicus* closed. *Dimensions*: Height up to 2.6 mm; width up to 2.1 mm; diameters of the first four whorls 0.5–0.6 mm, 0.8–0.9 mm, 1.1–1.2 mm, 1.35–1.55 mm respectively; number of whorls up to 6 1/2; height aperture up to 0.85 mm; width aperture up to 1.1 mm.

**Figure 75.**
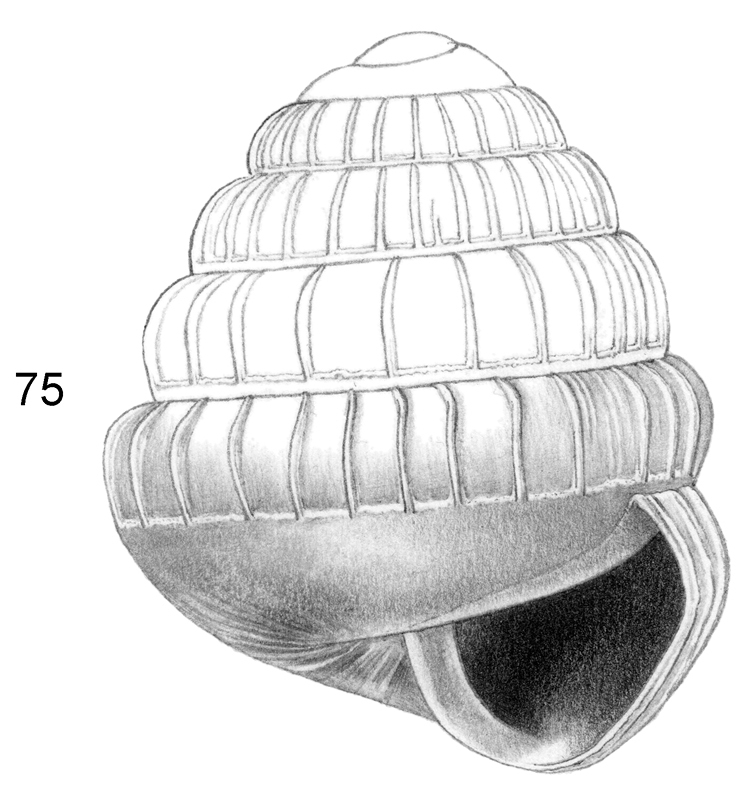
*Rahula delopleura* sp. n. Frontal view, shell 2.7 mm high (Malaysia, Sabah, Sandakan Province, Kinabatangan valley, Batu Pangi, RMNH.5003927, holotype).

##### Habitat in Sabah and distribution.

Primary and secondary forest on limestone soil, up to 500 m alt. **Sabah: Interior (Sepulut valley, upper Pinangah valley); East coast.** Endemic to Sabah.

##### Cross diagnosis.

Differs from *Rahula raricostulata* (E.A. Smith, 1893) (*Sitala raricostulata* E.A. Smith, 1893), from Sarawak, by the absence of a distinct, predominant spiral sculpture on the protoconch. *Rahula raricostulata* has a more conical spire, and fewer radial ribs (11–20) on the last whorl.

All continental Asiatic species of *Rahula* have prosocline radial ribs, see Blanford & Godwin Austen (1908: 249), and Godwin Austen (1918: 597).

##### Etymology.

The name refers to the sculpture [*delos* (Gr.) = manifest; *pleura* (Gr.) = rib].

#### Family PUNCTIDAE

Taxon classificationAnimaliaStylommatophoraPunctidae

Morse

##### Snails.

Shell very small, dextral, lenticular or discoid. Sculpture consisting of inconspicuous radial riblets, spiral sculpture subordinate. Aperture without teeth or lamellae, peristome usually thin and not reflected. Umbilicus open, narrow or wide (Family description adapted from Solem 1976, 1988; Vaught 1989; Herbert and Kilburn 2004).

##### Habitat and distribution.

Soil and litter dwellers. Widely distributed in Africa, Europe, Asia, and Australia.

##### Remarks.

The family is poorly known and although many genera and species have been described, the true diversity may consist of a smaller number of widespread, regionally variable species.

#### Genus Paralaoma

Taxon classificationAnimaliaStylommatophoraPunctidae

Iredale, 1913

Paralaoma Iredale, 1913: 380.

##### Remarks.

Sabah *Paralaoma* species have low-conical to lenticular shells, like *Microcystina*, but with a different sculpture: well-spaced radial ribs with a much finer, reticulate sculpture in between.

#### Paralaoma angusta

Taxon classificationAnimaliaStylommatophoraPunctidae

Vermeulen, Liew & Schilthuizen sp. n.

http://zoobank.org/026E5587-0FEE-48DF-9A8D-CC8301FB8172

Figure 76

##### Holotype.

Malaysia, Sabah, West Coast Province, Crocker Range, Kiansom Waterfall (RMNH.5003958).

##### Examined material from Sabah.

*West Coast Province*. Kiansom Waterfall in foothills of Crocker Range (leg. UMS Tropical Malacology Course, 2005, V 12736).

##### Description.

Shell minute, thin, slightly translucent, yellowish brown, low-conical to lenticular, apex rounded. Surface shiny. *Whorls* well-rounded. *Protoconch* sculpture: densely placed radial riblets which are particularly distinct towards the periphery; fine, rather densely placed spiral grooves cutting into the crests of the radial riblets. Teleoconch, radial sculpture: locally distinct, widely spaced radial ribs, often with a periostracal crest, which gradually disappear towards the umbilicus; in between these very fine, densely placed, low secondary riblets above the periphery. Spiral sculpture teleochonch: spiral threads, as fine and as densely placed as the secondary radial riblets, above the periphery, grading into widely spaced, rather distinct grooves towards the umbilicus. *Umbilicus* narrow, 5–6 % of the shell width. *Dimensions*: height up to 1.1 mm; width up to 1,6 mm; diameter of the first three whorls 0.40–0.45 mm, 0.75–0.80 mm, 1.25–1.30 mm respectively; number of whorls up to 3 3/4, height aperture up to 0.75 mm; width aperture up to 0.90 mm.

**Figure 76–77.**
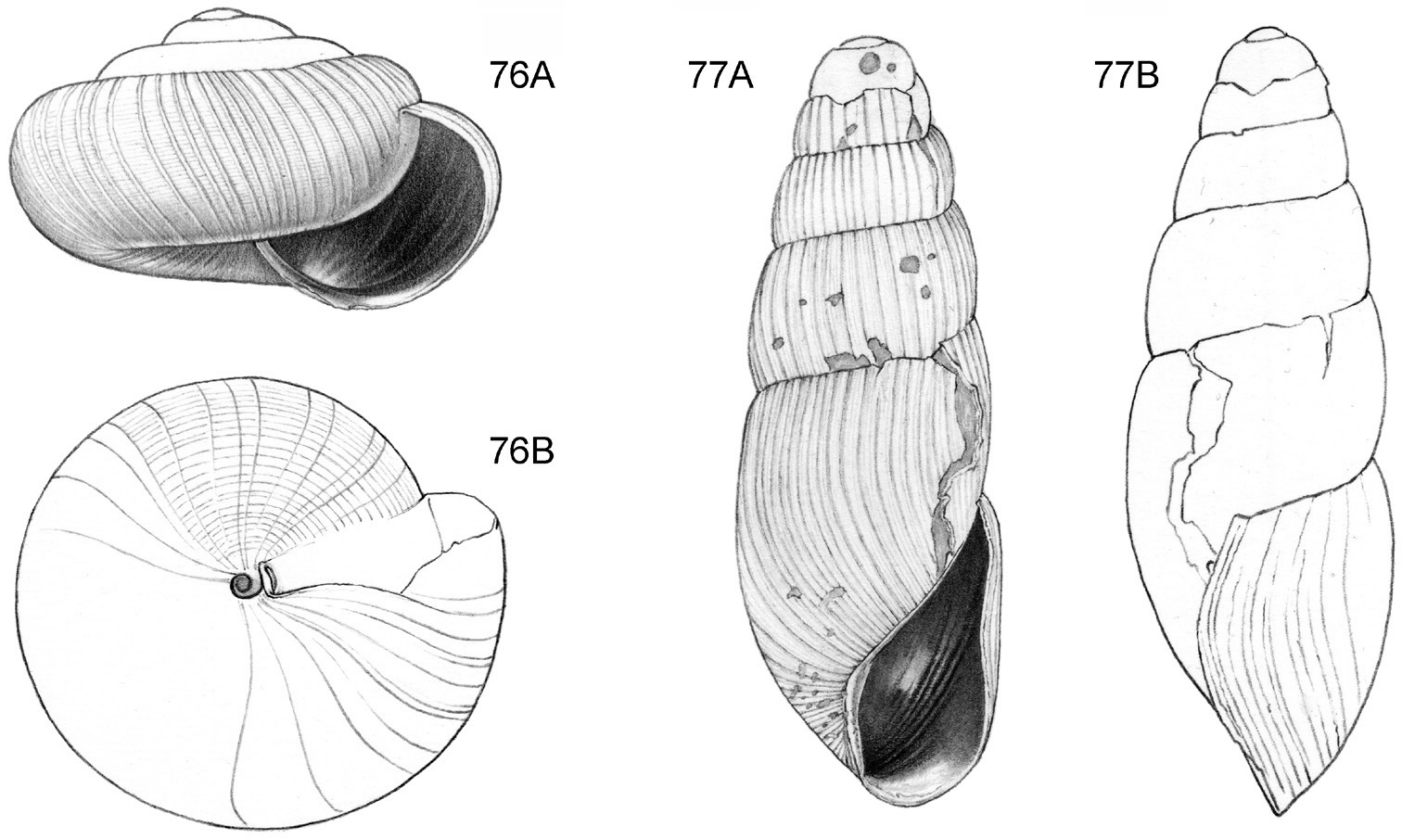
**76** *Paralaoma angusta* sp. n. **76A** Frontal view, shell 1.1 mm high **76B** Umbilical view (Malaysia, Sabah, West Coast Province, Crocker Range, Kiansom Waterfall, RMNH.5003958, holotype, shell partly reconstructed) **77** *Curvella hadrotes* sp. n. **77A** Frontal view, shell 7.8 mm high **77B** Same shell, right lateral view (Malaysia, Sabah, Tawau Province, Danum Valley Conservation Area, 2 km NW of Research Station, BOR/MOL 663, holotype).

##### Habitat in Sabah and distribution.

Secondary forest on sandstone hills, lowland conditions. **Sabah: West Coast, one locality known.** Endemic to Sabah.

##### Cross diagnosis.

Characterized within the genus by the narrow umbilicus, the presence of radial riblets on the protoconch, and the distinct and widely spaced spiral sculpture on the lower surface.

##### Etymology.

The name refers the the narrow umbilicus [*angustus* (L.) = narrow].

#### Family SUBULINIDAE

Taxon classificationAnimaliaStylommatophoraSubulinidae

Fischer & Crosse

##### Short description.

Snails. Shell small to very large, dextral (rarely sinistral), narrowly conical to cylindrical and more or less tapering towards the apex. Sculpture absent, or inconspicuous, radial riblets with usually subordinate spiral striation; rarely spiral keels present. Aperture without teeth or lamellae, peristome thin, not reflected. Umbilicus closed or open, narrow (Family description adapted from on Abbott 1989; Herbert and Kilburn 2004).

##### Habitat and distribution.

Scavengers of soil and litter. Globally distributed in the tropics and subtropics.

#### Genus Curvella

Taxon classificationAnimaliaStylommatophoraSubulinidae

Chaper, 1885

Curvella Chaper, 1885: 49.

##### Remarks.

The generic and specific classification of the Subulinidae are still unresolved. Applying Pilsbry 1906 [1906–1907] leads to inclusion of the species below in the genus *Curvella*, but its position is by no means certain.

#### Curvella hadrotes

Taxon classificationAnimaliaStylommatophoraSubulinidae

Vermeulen, Liew & Schilthuizen sp. n.

http://zoobank.org/768337AA-4268-4C70-9584-E90C9A473C17

Figure 77

##### Holotype.

Malaysia, Sabah, Tawau Province, Danum Valley Conservation Area, 2 km NW of Research Station (leg. H.A. Rutjes, BOR/MOL 663).

##### Examined material from Sabah.

*Interior Province*. Pinangah valley, Batu Urun (= Bukit Sinobang) (leg. J.J. Vermeulen, V 1160). *Sandakan Province*. Segama valley, North end of limestone ridge on East bank Tabin River (leg. J.J. Vermeulen & M. Schilthuizen, V 7778). *Tawau Province*. Segama valley, ‘Kirk’s Cave’ 8 km North of Lahad Datu (leg. J.J. Vermeulen, V 1222).

##### Description.

Shell small, thin, slightly translucent, white, subcylindrical, only slightly tapering towards the apex, with slightly convex sides. Surface glossy. *Whorls*: apical whorls moderately convex, others slightly convex, the last slightly flattened around the periphery; suture moderately impressed. *Protoconch* large, smooth. *Teleoconch*. Radial sculpture predominant, widely and somewhat irregularly spaced, rather fine but narrow and rather deep grooves, in between these a finer, inconspicuous striation present locally. Spiral sculpture: absent or a very fine spiral striation, particularly present locally on the apical whorls, shallowly engraved in the areas in between the radial grooves. Aperture oblique, narrowly ovate, peristome not thickened nor reflected. *Umbilicus* closed. *Dimensions*: Height 7.5–7.8 mm; width 1.4–1.5 mm; number of whorls c. 6 1/8, height aperture 2.8–3 mm; width aperture 1.4–1.5 mm.

##### Habitat in Sabah and distribution.

Primary and secondary forest on limestone, also on other types of bedrock. Alt. 0–400 m. **Sabah: scattered localities. Infrequent.** Endemic to Sabah.

##### Cross diagnosis.

Among Borneo Subulinidae, *Curvella hadrotes* is identified by its subcylindrical shell and the radial sculpture.

##### Etymology.

The name refers to the general shape of the shell [*hadrotes* (Gr.) = thick].

#### Family TROCHOMORPHIDAE

Taxon classificationAnimaliaStylommatophoraTrochomorphidae

Von Möllendorff

##### Short description.

Snails, rarely semi-slugs. Shell medium-sized to very large, dextral or sinistral, (low-)conical, to lenticular or discoid. Sculpture usually inconspicuous to distinct, fine spiral striation and/or radial riblets; in some species coarser sculpture is present, for instance spiral ridges or nodular structures on the intersection of spiral and radial sculpture. Aperture without teeth or lamellae, peristome thin or thick, reflected or not. Umbilicus closed or open, narrow (Family description adapted from Baker 1941; Van Benthem Jutting 1952; Solem 1959; Vaught 1989; Abbott 1989).

##### Habitat and distribution.

Most species are found on understorey vegetation. Widely distributed in Southeast Asia and Oceania.

##### Remarks.

The genera reviewed are *Trochomorpha* Albers, and *Geotrochus* Van Hasselt. Van Benthem Jutting (1952: 407) and Solem (1959: 107) include both genera in the Zonitidae, subfamily Trochomorphinae, in recent literature (Vaught 1989: 98) raised to family level: Trochomorphidae. The Sabah species are generally known by their shell only. An exception is *Trochomorpha rhysa*; dissection by Tillier and Bouchet (1988) confirmed its position in the Trochomorphidae.

Generally, the two genera include species with conical shells, not unlike *Kaliella* Blanford (Helicarionidae) or *Philalanka* Godwin Austen (Endodontidae), but consistently larger. Several species, for instance *Geotrochus conicoides*, *Geotrochus labuanensis*, and *Geotrochus scolops*, have shells of very characteristic appearance, with a prominent, sharp peripheral keel.

#### Genus Trochomorpha

Taxon classificationAnimaliaStylommatophoraTrochomorphidae

Albers, 1850

Trochomorpha Albers, 1850: 116; Albers in Albers & Von Martens, 1860: 60.

##### Diagnosis for the Sabah species.

Shell rather small to medium-sized, (pale) yellowish green to (yellowish) brown, without any colour patterns, or with lighter or darker streaks following the growth lines; low-conical with flat or slightly convex sides, apex protruding or not. Radial sculpture above the periphery distinct, consisting of riblets or irregularly spaced, raised growth lines locally causing a coarse, irregular wrinkling. Spiral sculpture distinct, consisting of threads which are highest or nodular where crossing the radial sculpture, lower or even absent elsewhere. Umbilicus closed, entirely covered by an extension of the parietal callus of the peristome.

##### Cross diagnosis.

Shares the closed umbilicus with Borneo *Geotrochus*. We keep the two separate, although the Sabah representatives of both genera have very similar shells. Sabah *Trochomorpha* is characterized by a coarser radial sculpture on the upper surface of the shell. The spiral sculpture overlies the radial sculpture, forming nodes where the two cross.

##### Remarks.

Possibly, the protoconch with radial riblets can be added to the diagnostic set distinguishing between Borneo *Geotrochus* and *Trochomorpha*. Unfortunately, we could not check this for all Borneo *Trochomorpha* species.

We provide a review of the Sabah species of *Trochomorpha*.

#### Trochomorpha trachus

Taxon classificationAnimaliaStylommatophoraTrochomorphidae

Vermeulen, Liew & Schilthuizen sp. n.

http://zoobank.org/913524B3-224A-4EAB-8E86-284B40603425

Figure 78

##### Holotype.

Malaysia, Sabah, West Coast Province, Crocker Range N.P., near the km 54 marker on the road Kota Kinabalu-Tambunan, Gunung Mas (leg. J.J. Vermeulen & M. Schilthuizen, RMNH.5003928).

##### Examined material from Sabah.

*West Coast Province*, Crocker Range N.P., near the km 54 marker on the road Kota Kinabalu-Tambunan, Gunung Mas (leg. M. Schilthuizen, BOR/MOL 2959).

##### Description.

Shell rather small, thin, opaque, reddish brown with darker brown or whitish streaks following growth lines, slightly paler brown with similar darker streaks below the suture, conical with flat sides; apex not protruding. Surface dull or slightly shiny above the periphery, glossy below. *Whorls* somewhat convex, outer whorls slightly shouldered; suture impressed, coinciding with the periphery; last whorl distinctly angular and slightly compressed at the periphery, moderately rounded below the periphery. *Protoconch* (absent in available material). *Teleoconch*. Radial sculpture: above the periphery irregularly spaced growth lines, developing into locally coarse, irregular wrinkling on the outer whorls, particularly below the suture; below the periphery indistinct growth lines only. Spiral sculpture: last whorl with a peripheral thread; start of fifth whorl above the periphery with 8–9 spiral threads: 3–4 coarse, high and rather narrow threads, with c. 5 minor threads interspersed; all threads highest and most distinct over the raised radial sculpture, low or interrupted in between, at irregular intervals, and with a somewhat erose and locally incised crest; below the periphery a few traces of densely placed, shallow spiral grooves. *Umbilicus* closed. *Peristome* not thickened, nor reflexed (see remarks, below). *Dimensions*: Height c. 8.5 mm; width c. 12.5 mm, h/w c. 0.66; estimated diameters of the first 4 whorls c. 1.2 mm, c. 2.3 mm, c. 4.0 mm, c. 6 mm respectively; number of whorls c. 5 3/4, height aperture c. 4 mm; width aperture c. 7 mm.

**Figure 78.**
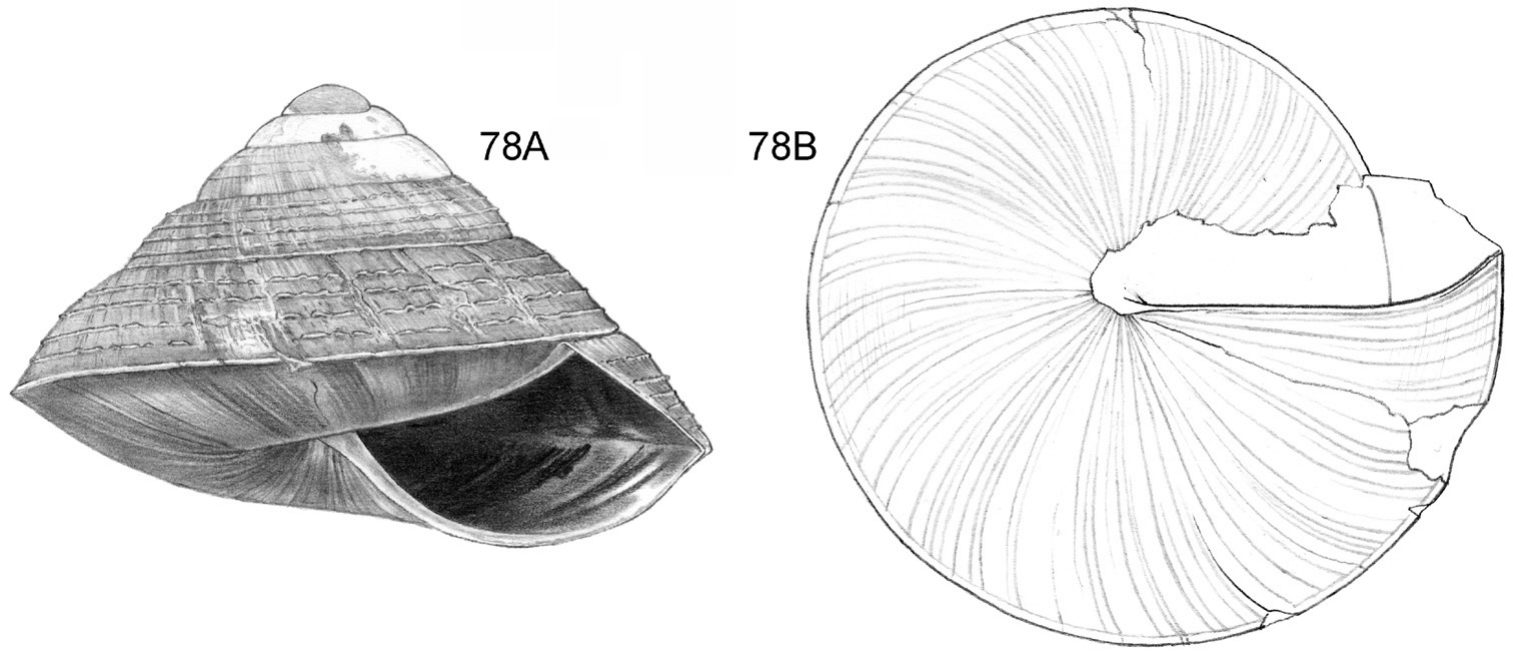
*Trochomorpha trachus* sp. n. **78A** Frontal view, shell 8.5 mm wide **78B** Umbilical view (Malaysia, Sabah, West Coast Province, Crocker Range N.P., near the km 54 marker on the road Kota Kinabalu-Tambunan, Gunung Mas, RMNH.5003928, holotype, apex and aperture reconstructed).

##### Habitat in Sabah and distribution.

Mixed montane forest, c. 1400 m alt. **Sabah: Crocker Range only.** Endemic to Sabah.

##### Cross diagnosis.

Uniquely identified by the very coarse, at irregular intervals interrupted spiral sculpture.

##### Remarks.

The material seen is probably sub-adult, and has the apex damaged. The description of the apex and the peristome will need amendation once more material becomes available.

##### Etymology.

The name refers to the shell surface [*trachus* (Gr.) = rough].

#### Trochomorpha rhysa

Taxon classificationAnimaliaStylommatophoraTrochomorphidae

Tillier & Bouchet, 1988

Figure 79

Trochomorpha rhysa Tillier & Bouchet, 1988: 257. Type from Malaysia, Sabah, Mount Kinabalu.

##### Examined material from Sabah.

*West Coast Province*. Kinabalu N.P., Summit trail between 2526 and 3200 m (leg. T.S. Liew, BOR/MOL 3979, V 14493; leg. K. Kittel,V 4806); near Laban Rata (leg. M. Schilthuizen & P. Koomen, BOR/MOL 2783); Mesilau trail between 2404 and 2680 m (leg. T.S. Liew, BOR/MOL 3983, BOR/MOL 3977, BOR/MOL 3981, BOR/MOL 3978); Kiau-Spurs trail 2688 and 2944 m (leg. T.S. Liew, J. Lapidin & Safrie, BOR/MOL 4403, BOR/MOL 3980); Kotal’s route at 2992 m (leg. T.S. Liew, J. Lapidin, Safrie & Jasilin, BOR/MOL 3982); Sayap-Nunuhon trail between 2480 and 2640 m (leg. T.S. Liew, Dominik, J. Lapidin & Jasilin, BOR/MOL 3984, BOR/MOL 3986, BOR/MOL 4401).

##### Description.

Shell rather small, rather thin, about opaque, (pale) yellowish brown to pale greenish brown, moderately low-conical with approx. flat sides; apex not protruding. Surface with a silky luster above the periphery, shiny below. *Whorls* somewhat convex, outer whorls slightly shouldered; suture impressed, coinciding with the periphery; last whorl distinctly angular but not or hardly compressed at the periphery, rounded below the periphery. *Protoconch* 2 1/8–2 1/4 whorls, with dense radial riblets except at the apex; transition to teleoconch sculpture abrupt. *Teleoconch*. Radial sculpture: above the periphery rather densely placed, more or less regularly spaced riblets, slightly more irregular on the last whorl and fading towards the aperture; below the periphery growth lines only. Spiral sculpture: last whorl with a peripheral thread; start of fifth whorl with 14–19 fine, rather low and wide spiral threads or more or less equal strength, forming nodes over the radial sculpture (less distinctly so on the earlier teleoconch whorls), threads on the last whorl dissolving in rows of granules; no spiral sculpture below the periphery. *Umbilicus* closed. *Peristome* moderately thickened and reflexed, more distinctly so on the columellar side. *Dimensions*: Height 6.0–6.8 mm; width 10–12 mm, h/w 0.57–0.62; diameters of the first 4 whorls 1.4–1.5 mm, 2.3–2.5 mm, 3.7–4.0 mm, 5.5–6 mm respectively; number of whorls 5 3/4–6, height aperture 3.0–3.8 mm; width aperture 5–6 mm.

**Figure 79–81.**
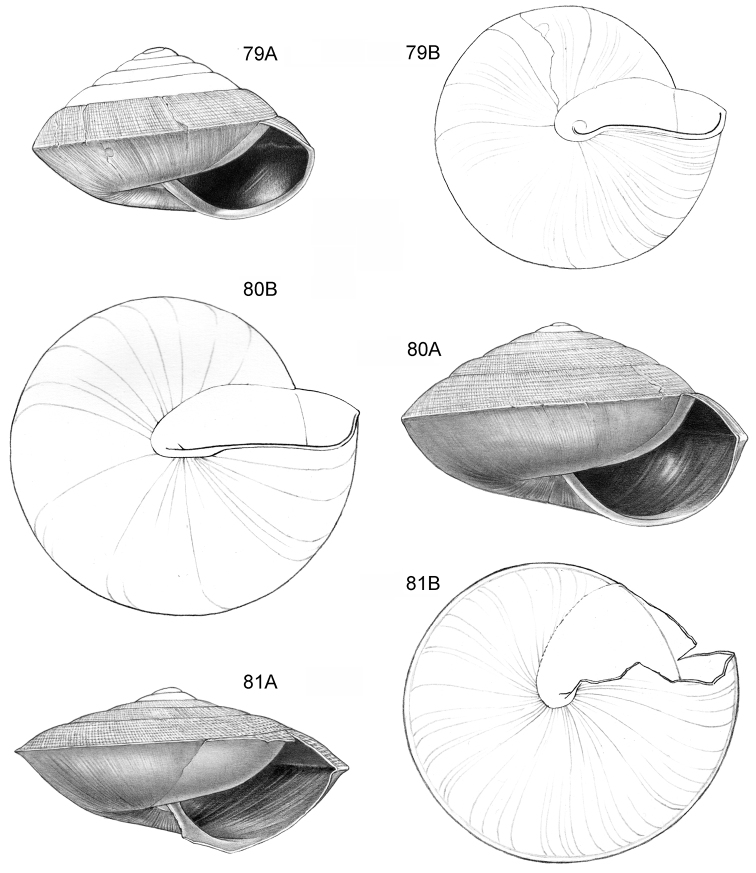
**79** *Trochomorpha rhysa* Tillier & Bouchet, 1988. **79A** Frontal view, shell 5.8 mm high **79B** Umbilical view (Malaysia, Sabah, West Coast Province, Kinabalu N.P., Paka Cave, V 4806) **80** *Trochomorpha haptoderma* sp. n. **80A** Frontal view, shell 10.0 mm high **80B** Umbilical view (Malaysia, Sabah, West Coast Province, Kinabalu N.P., near Summit trail, BOR/MOL 6046, holotype) **81** *Trochomorpha thelecoryphe* sp. n. **81A** Frontal view, shell 5.5 mm high **81B** Umbilical view (Malaysia, Sabah, West Coast Province, Kinabalu N.P., near Summit trail, BOR/MOL 6047, holotype).

##### Habitat in Sabah and distribution.

Montane and sub-alpine forest on sandstone and granodiorite bedrock, 2400–3300 m alt. **Sabah: Mount Kinabalu only.** Endemic to Sabah.

##### Cross diagnosis.

See under *Trochomorpha haptoderma* and *Trochomorpha thelecoryphe*.

#### Trochomorpha haptoderma

Taxon classificationAnimaliaStylommatophoraTrochomorphidae

Vermeulen, Liew & Schilthuizen sp. n.

http://zoobank.org/EA55AD3C-78F2-4DD9-B921-C4F5BF49F9A4

Figure 80

##### Holotype.

Malaysia, Sabah, West Coast Province, Kinabalu N.P., near Summit trail (leg. T.S. Liew et al., BOR/MOL 6046).

##### Examined material from Sabah.

*West Coast Province*. Kinabalu N.P., Summit trail between 2560 and 3330 m (leg. M. Schilthuizen & P. Koomen, BOR/MOL 1205; leg. T. Narainan, BOR/MOL 2673, BOR/MOL 2690, BOR/MOL 2880; leg. T.S. Liew, BOR/MOL 3995; leg. M. Schilthuizen, BOR/MOL 2997); Kotal’s route between 2376 and 3112 m (leg. T.S. Liew, BOR/MOL 3987; (leg. T.S. Liew & Jasilin, BOR/MOL 3988, BOR/MOL 4106, BOR/MOL 3989, BOR/MOL 3990, BOR/MOL 3991); Kiau-Spurs trail between 3024 and 3088 m (leg. T.S. Liew, J. Lapidin & Safrie, BOR/MOL 3994, BOR/MOL 3997, BOR/MOL 3992); Sayap-Nunuhon trail between 2320 and 2944 m (leg. T.S. Liew, Dominik, J. Lapidin & Jasilin, BOR/MOL 4405, BOR/MOL 3993); Mesilau trail between 1948 and 2528 m (leg. T.S. Liew, BOR/MOL 3996); Monggis-Tambuyukon trail at 2360 m (leg. T.S. Liew, BOR/MOL 4108).

##### Description.

Shell medium-sized, rather thin, about opaque, yellowish brown, moderately low-conical with somewhat convex sides; apex slightly protruding. Surface with a silky luster above the periphery, shiny below. *Whorls*: Apical whorls convex, outer slightly convex; suture moderately impressed, coinciding with the periphery; last whorl angular, hardly compressed at the periphery, rounded below the periphery. *Protoconch* 1 7/8–2 1/4 whorls, with dense radial riblets except at the apex, and a spiral striation cutting into the radial riblets towards the teleoconch; transition to teleoconch sculpture gradual. *Teleoconch*. Radial sculpture: above the periphery rather densely placed, more or less regularly spaced riblets; below the periphery growth lines only. Spiral sculpture: last whorl with a peripheral thread; start of fifth whorl with 11–14 spiral threads: 2–8 rather distinct, rather low and wide threads but with a narrow crest, which are highest or form nodes over the radial sculpture, and 5–12 similar but less conspicuous threads interspersed; no spiral sculpture below the periphery. *Umbilicus* closed. *Peristome* slightly thickened and slightly reflexed, more distinctly so on the columellar side. *Dimensions*: Height 7.4–10.0 mm; width 14.0–17.0 mm; h/w 0.53–0.59; diameters of the first 4 whorls 1.7–1.9 mm, 3.0–3.3 mm, 5.0–5.5 mm, 7.5–8.2 mm respectively; number of whorls 5 1/2–6 1/8, height aperture 5.0–6.0 mm; width aperture 6.5–8.5 mm.

##### Habitat in Sabah and distribution.

Montane and sub-alpine forest on sandstone bedrock, 1900–3300 m alt. **Sabah: Mount Kinabalu only.** Endemic to Sabah.

##### Cross diagnosis.

Consistently larger than *Trochomorpha rhysa*, and less densely coiled. The sculpture above the periphery is coarser, particularly the radial riblets on the protoconch, as well as the spiral threads on the teleoconch. The latter are also less regularly spaced, and of more unequal strength, with much more inconspicuous threads interspersed in between the distinct ones.

##### Etymology.

The name refers to the knotted shell surface [*hapto* (Gr.) = to knot; *derma* (Gr.) = skin].

#### Trochomorpha thelecoryphe

Taxon classificationAnimaliaStylommatophoraTrochomorphidae

Vermeulen, Liew & Schilthuizen sp. n.

http://zoobank.org/DAACED0A-AD65-4C3C-B8D8-29A441316F6A

Figure 81

##### Holotype.

Malaysia, Sabah, West Coast Province, Kinabalu N.P., near Summit trail (leg. T.S. Liew et al., BOR/MOL 6047).

##### Examined material from Sabah.

*West Coast Province*. Kinabalu N.P., Kotal’s route between 1992 and 2280 m (leg. T.S. Liew, J. Lapidin, Safrie & Jasilin, BOR/MOL 4395, BOR/MOL 4398); Mesilau trail between 2120–2288 m (leg. T.S. Liew, BOR/MOL 4402, BOR/MOL 4397; leg. T.S. Liew & J. Lapidin, BOR/MOL 4394, BOR/MOL 4234).

##### Description.

Shell rather small, rather thin, about opaque, yellowish brown, low-conical with somewhat convex sides; apex protruding. Surface with a silky luster above the periphery, shiny below. *Whorls*: Apical whorls convex, outer slightly convex; suture hardly impressed, coinciding with the periphery; last whorl acutely angular and compressed at the periphery, rounded below the periphery. *Protoconch* c. 1 7/8 whorls, with dense radial riblets except at the apex, and a spiral striation cutting into the radial riblets towards the teleoconch; transition to teleoconch sculpture gradual. *Teleoconch*. Radial sculpture: above the periphery rather densely placed, more or less regularly spaced riblets; below the periphery growth lines only. Spiral sculpture: last whorl with a peripheral thread; start of fifth whorl with c. 13 spiral threads: c. 10 rather distinct, rather low and wide threads but with a narrow crest, which are highest, or form nodes over the radial sculpture, and c. 3 similar but less conspicuous threads interspersed; no spiral sculpture below the periphery. *Umbilicus* closed. *Peristome* (not present on the material available). *Dimensions*: Height c. 6 mm; width c. 11 mm; h/w 0.54–0.55; diameters of the first 4 whorls c. 1.7 mm, c. 2.8 mm, c. 5 mm, c. 8 mm respectively; number of whorls c. 5 1/8, height aperture c. 3.5 mm; width aperture c. 6 mm.

##### Habitat in Sabah and distribution.

Montane forest on sandstone bedrock, 1900-2300 m alt. **Sabah: Mount Kinabalu only.** Endemic to Sabah.

##### Cross diagnosis.

Similar to *Trochomorpha haptoderma* in coiling density and sculpture; differs by having a flatter spire, with a more protruding apex.

##### Remarks.

The only available shell is a juvenile with the last 1/8 whorl broken. The description needs to be amended once more material becomes available.

##### Etymology.

The name refers to the protruding apex [*thele* (Gr.) = nipple; *coruphe* (Gr.) = summit].

#### Genus Geotrochus

Taxon classificationAnimaliaStylommatophoraTrochomorphidae

Van Hasselt, 1823

Geotrochus Van Hasselt, 1823: 233.

##### Diagnosis for the Sabah species.

Shell rather small to medium-sized, (pale) corneous to (yellowish) brown, without any colour patterns, or with brown spiral bands (*Geotrochus spilokeiria* with additional white spots and stains); (low) conical with concave, flat or convex sides, apex protruding or not. Radial sculpture above the periphery inconspicuous to distinct growth lines, raised at irregular intervals or not. Spiral sculpture consisting of thin threads, at least some spiral threads close to the periphery usually slightly coarser, the others very fine, often inconspicuous or absent; no spiral sculpture below the periphery. Umbilicus closed, entirely covered by an extension of the parietal callus of the peristome.

##### Remarks.

We provide a review of the Sabah species of *Geotrochus*. We divide the genus into three informal groups.

#### Group 1a. Suture between the whorls coinciding with the periphery. Shell 13.5–24.5 mm wide in adult specimens.

##### Geotrochus paraguensis

Taxon classificationAnimaliaStylommatophoraTrochomorphidae

(E.A. Smith, 1893)

Figure 82

Trochonanina paraguensis E.A. Smith, 1893: 349. Type from Philippines, Palawan.
Trochonanina alexis E.A. Smith, 1895: 105, syn. n. Type from Malaysia, Sabah, Banggi Island.

###### Examined material from Sabah.

Kudat Province. Balambangan Island, Kok Simpul (leg. J.J. Vermeulen & M. Schilthuizen, V 9510, BOR/MOL 2433; leg. T.H. Liew, Sazilin & Ramlan, BOR/MOL 3700); Batu Sireh (leg. J.J. Vermeulen & M. Schilthuizen, V 9547). Banggi Island, South end, Karakit Hill (leg. J.J. Vermeulen, V 1462; leg. J.J. Vermeulen & M. Schilthuizen, V 9475).

###### Description.

Shell medium-sized, rather thin, opaque, uniformly pale yellowish brown, low-conical with flat to slightly convex sides; apex not protruding or only slightly so. Surface with a silky luster above the periphery, shiny below. *Whorls*: All whorls moderately convex; suture impressed in the inner whorls, not so in the outer, coinciding with the periphery; last whorl acutely angular, not or hardly compressed at the periphery, moderately rounded below the periphery. *Protoconch* 1 7/8–2 1/8 whorls, smooth. *Teleoconch*. Radial sculpture: above the periphery growth lines, raised locally and developing in patches with inconspicuous, somewhat irregularly spaced, fine, low riblets, particularly towards the periphery; below the periphery irregularly spaced growth lines only. Spiral sculpture: last whorl with a sharp but not pinched peripheral ridge; start of fifth whorl with 13–17 well-spaced, very fine and thin spiral threads covering most of the whorl except a narrow strip below the suture, at least some spiral threads close to the periphery usually slightly coarser; no spiral sculpture below the periphery. *Umbilicus* closed. *Peristome* thickened and reflexed. *Dimensions*: Height 6–8.5 mm; width 14–22.8 mm; h/w 0.37–0.42; peripheral keel of the last whorl at 0.39–0.53 of the shell height, measured from the apex; diameters of the first 4 whorls 1.4–1.8 mm, 2.4–2.7 mm, 4.0–4.6 mm, 5.7–6.9 mm respectively; number of whorls 6 3/4–7 5/8, height aperture 3.2–5.2 mm; width aperture 8–11.6 mm.

**Figure 82–85.**
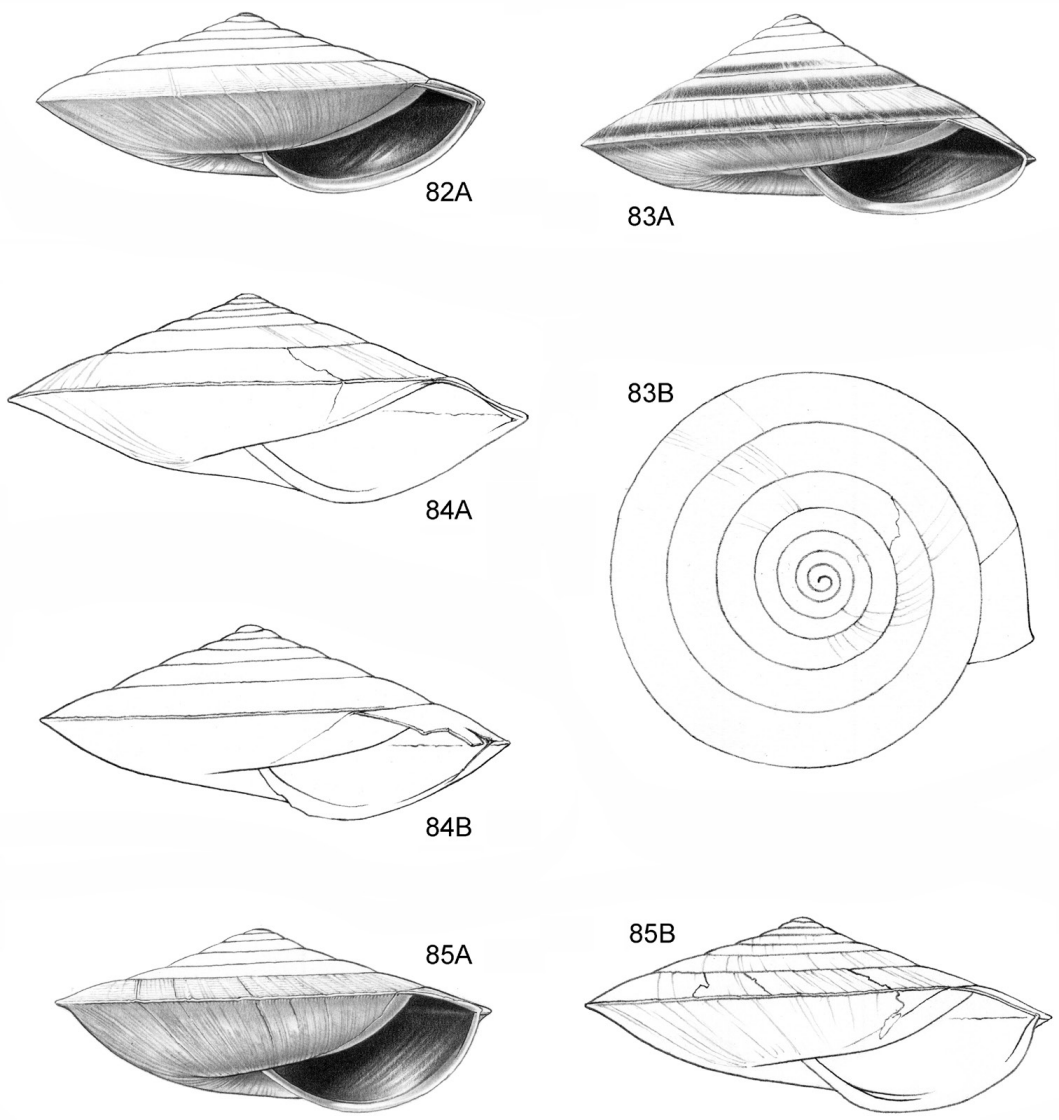
**82** *Geotrochus paraguensis* (E.A. Smith, 1893). Frontal view, shell 7.0 mm high (Malaysia, Sabah, Kudat Province, Balambangan Island, Kok Simpul (leg. J.J. Vermeulen & M. Schilthuizen, V 9510) **83** *Geotrochus labuanensis* (Pfeiffer, 1863) **83A** Frontal view, shell 7.0 mm high **83B** Apical view (Malaysia, Sabah, Tawau Province, Segama valley, hill N.W. of crossing road Sandakan-Lahad Datu with the Segama River,V 1656) **84** *Geotrochus kinabaluensis* (E.A. Smith, 1895) **84A** Frontal view, shell 10 mm high **84B** Frontal view, shell 7.4 mm high, aperture damaged (**84A** Malaysia, Sabah, Mount Kinabalu, NHMUK 1893.6.8.28-29, syntype of *Trochonanina kinabaluensis* **84B** Malaysia, Sabah, Interior Province, Crocker Range N.P., West of the km 10 marker on the road Tambunan-Ranau, Mahua Waterfall, BOR/MOL 3372) **85** *Geotrochus oedobasis* sp. n. **85A** Frontal view, shell 6.0 mm high **85B** Frontal view, shell 8.5 mm high (**85A** Malaysia, Sabah, West Coast Province, Kinabalu N.P., Headquarters area, RMNH.5003929, holotype; **85B** Malaysia, Sabah, *Interior Province*, Gunung Trusmadi slopes, Gua Loloposon, V 13258).

###### Habitat in Sabah and distribution.

Dry primary lowland forest, coastal forest on limestone soil. **Sabah**: **Banggi and Balambangan Islands.** Distribution: Philippines (Palawan).

###### Cross diagnosis.

Characterized by the combination of lack of spiral colour bands, the not-pinched periphery, the moderately convex whorls and lower surface of the shell.

#### *Geotrochus labuanensis*-group (*Geotrochus labuanensis*, *Geotrochus kinabaluensis*, *Geotrochus oedobasis*).

A group of very similar species, distinguished here on account of shell colour, aperture shape and the position of the peripheral keel relative to the shell height. Anatomical and molecular investigation is needed to further unravel the taxonomical structure of the group.

##### Geotrochus labuanensis

Taxon classificationAnimaliaStylommatophoraTrochomorphidae

(Pfeiffer, 1863)

Figure 83

Helix labuanensis Pfeiffer, 1863: 523. Nanina conicoides (Metcalfe) var. labuanensis (Pfeiffer) Tryon, 1886: 48. *Geotrochus labuanensis* (Pfeiffer) Solem, 1964: 27. Type from Malaysia, Sabah, Labuan.

###### Examined material from Sabah.

*Interior Province*. Crocker Range N.P., Gua Laing c. 12 km North of Keningau (leg. J.J. Vermeulen, V 1105). Sepulut valley, Batu Punggul (leg. J.J. Vermeulen, V 1980); Batu Temurung (leg. J.J. Vermeulen, V 8053); Bukit Tinagas, East end of Batu Punggul limestone (leg. J.J. Vermeulen & M. Schilthuizen, V 7637); Gua Pungiton (leg. J.J. Vermeulen & M. Schilthuizen, V 7562; leg. M. Schilthuizen, BOR/MOL 901); Gua Sanaron (leg. J.J. Vermeulen & M. Schilthuizen, V 7670; leg. M. Schilthuizen, BOR/MOL 905; leg. T.S. Liew, M. Schilthuizen & S. Chiba, BOR/MOL 4281, BOR/MOL 4316). *Sandakan Province*. Kinabatangan valley, Batu Keruak (leg. T.S. Liew & B. Elahan, BOR/MOL 1848, BOR/MOL 1899); Batu Mawas (leg. T.S. Liew & M. Schilthuizen, BOR/MOL 1955); Batu Pangi (leg. J.J. Vermeulen & M. Schilthuizen, V 9676, BOR/MOL 2434); Batu Tai (not Bod Tai) near Gomantong (leg. J.J. Vermeulen & M. Schilthuizen, V 9601; leg. M. Schilthuizen, BOR/MOL 2436); Batu Tomanggong Besar (leg. T.S. Liew & B. Elahan, BOR/MOL 2283); Batu Tomanggong Kecil (leg. M. Salverda & H. Van Oosten, BOR/MOL 2437); Gomantong Cave (leg. A. van Til, BOR/MOL 3281). Segama valley, North end of limestone ridge on East bank Tabin River (leg. J.J. Vermeulen & M. Schilthuizen, V 7764, BOR/MOL 902); Tabin Wildlife Reserve (leg. M. Schilthuizen, BOR/MOL 904, BOR/MOL 906). *Tawau Province*. Batu Baturong c. 50 km W.S.W. of Lahad Datu (leg. J.J. Vermeulen & H. Duistermaat, V 1851); North slope (leg. J.J. Vermeulen, V 7602). Danum Valley (leg. H.A. Rutjes, BOR/MOL 903); Gua Madai c. 40 km S.S.W. of Lahad Datu, N.E.-end (leg. J.J. Vermeulen, V 7711). Segama valley, hill N.W. of crossing road Sandakan-Lahad Datu with the Segama River (leg. J.J. Vermeulen & H. Duistermaat, V 1656); ‘Kirk’s Cave’ 8 km North of Lahad Datu (leg. J.J. Vermeulen, V 1210); limestone hill on North bank Segama River, near bridge of road Sandakan to Lahad Datu (leg. J.J. Vermeulen, V 7518); Sabahmas Cave (leg. J.J. Vermeulen, V 7470).

###### Description.

Shell medium-sized, rather thin, opaque, creamy white, greyish very pale yellowish brown, periphery whitish, with a narrow, rather vaguely outlined, pale to dark (ochre-)brown band immediately above it, and often a second, similar band immediately below it, shell low-conical with approx. flat sides, apex not or hardly protruding. Surface shiny. *Whorls*: Inner whorls moderately convex, outer whorls flat to slightly convex; suture impressed in the inner whorls, not so in the outer, coinciding with the periphery; last whorl acutely angular, slightly compressed at the periphery, slightly convex below the periphery. *Protoconch* 2 1/8–2 1/2 whorls, smooth. *Teleoconch*. Radial sculpture: above and below the periphery scattered inconspicuous growth lines, locally grading into inconspicuous riblets. Spiral sculpture: last whorl with a sharp, slightly pinched peripheral ridge; start of fifth whorl with 4–25 usually well-spaced, low and thin spiral threads, the 2–5 threads close to the periphery more distinct, the others very inconspicuous or absent; no spiral sculpture below the periphery. *Umbilicus* closed. *Peristome* thickened and reflexed, basal edge most strongly curved towards the columellar side, and often, to a somewhat lesser extent, towards the periphery; in between only slightly curved. *Dimensions*: Height 6.3–7.6 mm; width 14.5–17.8 mm; h/w 0.39–0.45; peripheral keel of the last whorl at 0.44–0.54 of the shell height, measured from the apex; diameters of the first 4 whorls 1.3–1.6 mm, 2.2–2.7 mm, 3.6–4.4 mm, 5.2–7.0 mm respectively; number of whorls 6 1/8–6 7/8, height aperture 3.0–3.6 mm; width aperture 7.4–9.2 mm.

###### Habitat in Sabah and distribution.

Primary and secondary forest on limestone soil. Lives on decaying wood. **Sabah: widespread, but scattered localities. More frequently found in the East part.** Endemic to Sabah.

###### Cross diagnosis.

Differs mainly from *Geotrochus kinabaluensis* and *Geotrochus oedobasis.* by the presence of a peripheral colour band (not always present), the slightly pinched periphery, and the less convex outer whorls. The position of the peripheral keel relative to the shell height is intermediate between *Geotrochus kinabaluensis* and *Geotrochus oedobasis*. The spiral sculpture above the periphery is often more widely spaced, and more unequal, with a few stronger threads close to the periphery and very inconspicuous ones, if any, elsewhere.

###### Remarks.

*Geotrochus labuanensis* is a lowland species, contrary to the other species in the group.

##### Geotrochus kinabaluensis

Taxon classificationAnimaliaStylommatophoraTrochomorphidae

(E.A. Smith, 1895)

Figure 84

Trochonanina kinabaluensis E.A. Smith, 1895: 105. Type from Malaysia, Sabah, Mount Kinabalu.
Trochonanina kinabaluensis E.A. Smith var. pallida E.A. Smith, 1895: 106. Type from Malaysia, Sabah, Mount Kinabalu.

###### Examined material from Sabah.

*Interior Province*. Crocker Range N.P., West of the km 10 marker on the road Tambunan-Ranau, Mahua Waterfall (leg. Suhaili, BOR/MOL 3372). *West Coast Province*. Kinabalu N.P., Poring Hot Springs, leg. M. Schilthuizen & P. Koomen, BOR/MOL 2933); Sayap-Nunuhon trail at 1152 m (leg. T.S. Liew, Dominik, J. Lapidin & Jasilin, BOR/MOL 4025); Serinsim-Nombuyokon trail at 5.6–6 km (leg. T.S. Liew, BOR/MOL 4026); Kiau-Spurs route at 2800 m (leg. T.S. Liew, J. Lapidin & Safrie, BOR/MOL 4027).

###### Description.

Shell medium-sized, rather thin, opaque, brown, sometimes a slightly paler brown just below the suture, periphery pale brown to dull white; shell low-conical with approx. flat sides, apex not or hardly protruding. Surface with a silky luster above the periphery, shiny below. *Whorls*: slightly convex; suture impressed in the inner whorls, not so in the outer, coinciding with the periphery; last whorl acutely angular, moderately compressed at the periphery, slightly convex below the periphery. *Protoconch* c. 2 1/8 whorls, smooth. *Teleoconch*. Radial sculpture: above and below the periphery scattered inconspicuous growth lines, locally grading into inconspicuous riblets; next to this a very fine (just visible at 40 times magnification) radial wrinkling of the shell surface is locally present. Spiral sculpture: last whorl with a sharp, slightly pinched peripheral ridge; start of fifth whorl with 15–17 somewhat spaced, low and thin spiral threads, the 2–5 threads close to the periphery slightly more distinct, the others inconspicuous; no spiral sculpture below the periphery. *Umbilicus* closed. *Peristome* thickened and reflexed, basal edge most strongly curved towards the columellar side, approx. straight or slightly concave towards the periphery. *Dimensions*: Height 7.4–10.0 mm; width 17.6–24.5 mm; h/w 0.41–0.42; peripheral keel of the last whorl at 0.40–0.45 of the shell height, measured from the apex; diameters of the first 4 whorls 1.5–1.6 mm, 2.4–2.6 mm, 3.4–4.6 mm, 5.8–6.5 mm respectively; number of whorls 6 1/2–6 3/4, height aperture 4.0–5.5 mm; width aperture 9.0–13.0 mm.

###### Habitat in Sabah and distribution.

Primary and secondary forest on sandstone and shale bedrock, 600–2800 m alt. Lives on decaying wood. **Sabah: Mount Kinabalu, Crocker Range.** Endemic to Sabah.

###### Cross diagnosis.

See under *Geotrochus labuanensis*. The limited material seen of *Geotrochus kinabaluensis* suggests that the shells are usually higher and wider than those of *Geotrochus labuanensis*, and that the aperture is usually larger.

###### Remarks.

The exact morphology of a very fine radial wrinkling of the supra-peripheral part of the shell needs to be investigated.

*Geotrochus kinabaluensis* is a submontane species, contrary to *Geotrochus labuanensis*.

##### Geotrochus oedobasis

Taxon classificationAnimaliaStylommatophoraTrochomorphidae

Vermeulen, Liew & Schilthuizen sp. n.

http://zoobank.org/203134B5-2AD4-4B07-BF30-581A483C5C44

Figure 85

###### Holotype.

Malaysia, Sabah, West Coast Province, Kinabalu N.P., Headquarters area (leg. M. Schilthuizen, RMNH.5003929).

###### Examined material from Sabah.

*Interior Province*. Crocker Range, Gn. Emas (leg. M. Schilthuizen, BOR/MOL 909); Gunung Trusmadi slopes, Gua Loloposon (leg. J.J. Vermeulen, V 13258; leg. M. Schilthuizen & P. Koomen, BOR/MOL 911); Gua Dawaras (leg. M. Schilthuizen, V 13522; leg. M. Schilthuizen & M. Suleiman, BOR/MOL 2418, BOR/MOL 2419). *West Coast Province*. Kinabalu N.P., Headquarters area (leg. J.J. Vermeulen, V 1191); Summit trail between 1350 and 1800 m (leg. M. Schilthuizen, BOR/MOL 908; leg. T.S. Liew, BOR/MOL 4400); Mesilau trail between 2040 and 2112 m (leg. T.S. Liew & J. Lapidin, BOR/MOL 4399, BOR/MOL 4404, BOR/MOL 4407; leg. T.S. Liew, BOR/MOL 4406, BOR/MOL 4408).

###### Description.

Shell medium-sized, rather thin, opaque, uniformly somewhat pale brown, periphery pale brown; shell low-conical with approx. flat or slightly concave sides, apex not or hardly protruding. Surface with a silky luster above the periphery, shiny below. *Whorls*: apical whirls slightly convex others approx. flat; suture somewhat impressed in the inner whorls, not so in the outer, coinciding with the periphery; last whorl acutely angular, (moderately) compressed at the periphery, moderately to distinctly convex below the periphery. *Protoconch* 2–2 1/8 whorls, smooth. *Teleoconch*. Radial sculpture: above and below the periphery scattered inconspicuous growth lines, locally grading into inconspicuous riblets; next to this a very fine (just visible at 40 times magnification) radial wrinkling of the shell surface is locally present. Spiral sculpture: last whorl with a sharp, slightly pinched peripheral ridge; start of fifth whorl with 19–23 somewhat spaced, low and thin spiral threads, the 2–5 threads close to the periphery slightly more distinct, the others inconspicuous; no spiral sculpture below the periphery. *Umbilicus* closed. *Peristome* thickened and reflexed, basal edge more or less evenly rounded from the columellar side to the periphery. *Dimensions*: Height 6.0–8.5 mm; width 13.5–18.0 mm; h/w 0.39–0.45; peripheral keel of the last whorl at 0.33–0.40 of the shell height, measured from the apex; diameters of the first 4 whorls 1.3–1.4 mm, 2.2–2.3 mm, 3.7–4.0 mm, 5.9–6.4 mm respectively; number of whorls 6–7 1/8, height aperture 3.5–5.2 mm; width aperture 7.0–10.0 mm. Shell medium-sized, thin, about opaque, uniformly pale yellowish brown.

###### Habitat in Sabah and distribution.

Forest on sandstone or limestone soil, also found in grassy roadside with forest nearby, alt. 900–2200 m. **Sabah: Mount Kinabalu, Mount Trusmadi.** Endemic to Sabah.

###### Cross diagnosis.

See under *Geotrochus labuanensis*. Differs from *Geotrochus kinabaluensis* by the evenly rounded basal peristome. Relative to the shell height, the peripheral keel is positioned closer to the apex of the shell. It makes the lower side of the shell appear more convex, and the upper side flatter than in *Geotrochus kinabaluensis*.

###### Etymology.

The name refers to the convex lower side of the shell [*oidos* (Gr.) = swelling; *basis* (Gr.) = base].

##### Geotrochus conicoides

Taxon classificationAnimaliaStylommatophoraTrochomorphidae

(Metcalfe, 1851)

Figure 86

Helix conicoides Metcalfe, 1851: 71. *Trochomorpha conicoides* (Metcalfe) Wallace, 1865: 407. *Nigritella conicoides* (Metcalfe) Godwin Austen, 1891: 42. *Dendrotrochus conicoides* (Metcalfe) Kobelt, 1897: 50. *Trochonanina conicoides* (Metcalfe) Von Martens, 1908: 284. *Eurybasis conicoides* (Metcalfe) Van Benthem Jutting, 1941: 23. *Geotrochus conicoides* (Metcalfe) Van Benthem Jutting, 1959: 143. Type from Borneo (unspecified).

###### Examined material from Sabah.

*West Coast Province*. Pulau Gaya, 8 km Northwest of Kota Kinabalu (leg. M. Schilthuizen, BOR/MOL 2937). Pulau Tiga (leg. UMS students, BOR/MOL 899). *Tawau Province*. Tawau Hills N.P., path up to Bukit Bombalai (leg. J.J. Vermeulen, V 13167).

###### Description.

Shell medium-sized, rather thin, opaque, uniformly pale to dark brown (some material from Sarawak a paler brown just below the suture), (low-)conical with almost flat to distinctly concave sides, apex slightly to distinctly protruding. Surface more or less dull or with a silky luster above the periphery, shiny below. *Whorls*: Apical whorls moderately convex, next whorls approx. flat to slightly convex, outer whorls often flat; suture impressed in the inner whorls, not so in the outer, coinciding with the periphery; last whorl acutely angular, (distinctly) compressed at the periphery, slightly to rather distinctly concave just below the periphery, slightly to moderately convex towards the umbilical region. *Protoconch* 1 7/8–2 1/2 whorls, smooth. *Teleoconch*. Radial sculpture: above and below the periphery inconspicuous growth lines, somewhat raised locally. Spiral sculpture: last whorl with a sharp, distinctly pinched peripheral ridge; start of fifth whorl with 5–17 rather densely placed to somewhat spaced, low and thin spiral threads, the 2–5 threads close to the periphery usually slightly more distinct, the others very inconspicuous or (almost) absent; no spiral sculpture below the periphery. *Umbilicus* closed. *Peristome* thickened and reflexed. *Dimensions*: Height 7.7–10.0 mm; width 16–19.8 mm; h/w 0.42–0.53; peripheral keel of the last whorl at 0.57–0.66 of the shell height, measured from the apex; diameters of the first 4 whorls 1.3–1.5 mm, 2.0–2.5 mm, 2.5–4.0 mm, 4.2–5.6 mm respectively; number of whorls 7–8, height aperture 2.8–3.8 mm; width aperture 8–9.5 mm.

**Figure 86–88.**
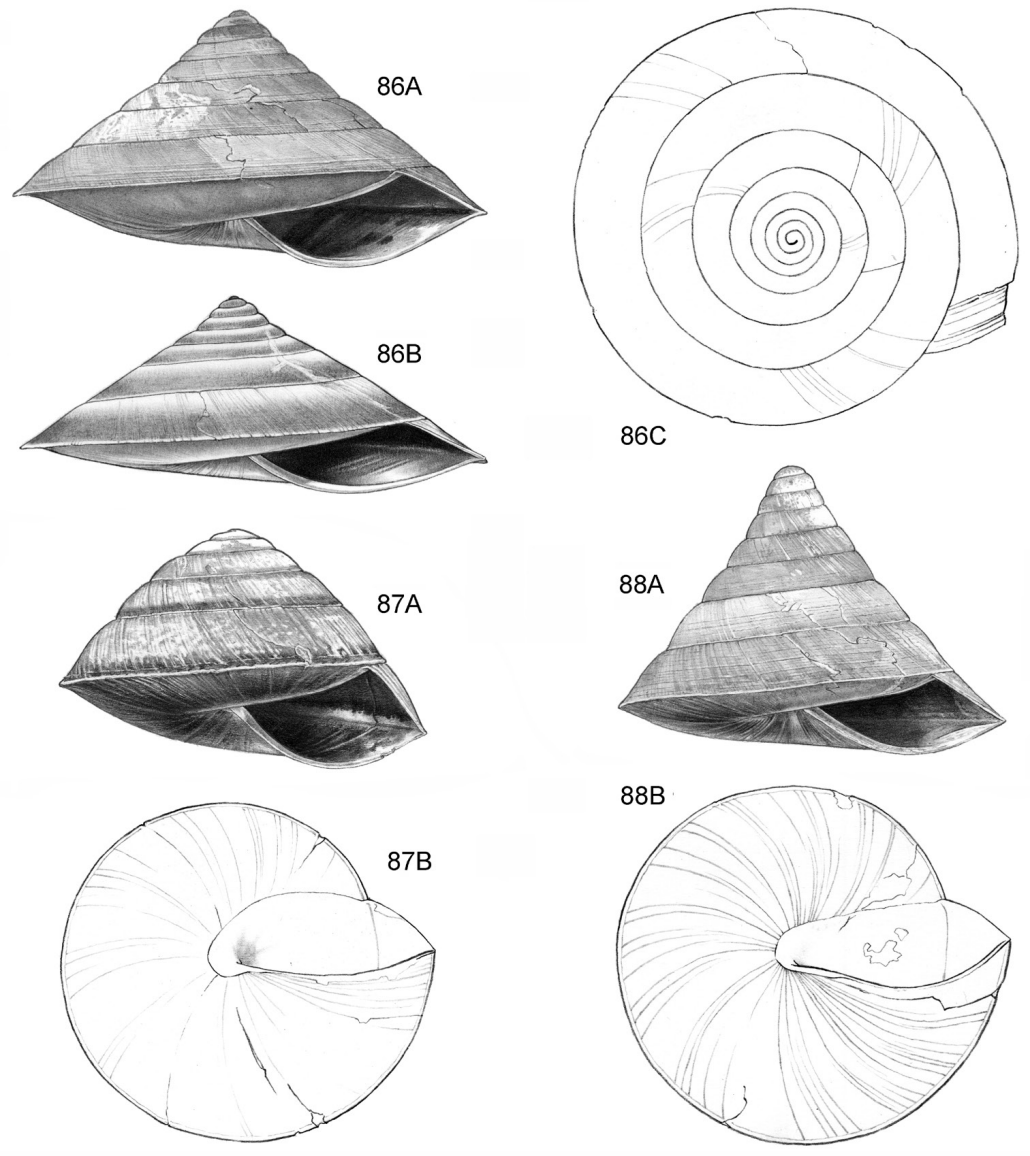
**86** *Geotrochus conicoides* (Metcalfe, 1851) **86A** Frontal view, shell 8.5 mm high **86B** Frontal view, shell 11.0 mm high **86C** Apical view (**86A, 86C** Malaysia, Sarawak, 4th Division, Niah Caves N.P., N and NW side of limestone massif, V 1573 **86B** Malaysia, Sabah, Tawau Province, Tawau Hills N.P., path up to Bukit Bombalai, V 13167) **87** *Geotrochus spilokeiria* sp. n. **87A** Frontal view, shell 8.5 mm high **87B** Umbilical view (Malaysia, Sabah, West Coast Province, Crocker Range N.P., Ulu Kimanis, along Keningau-Kimanis road, BOR/MOL 2784, holotype, aperture slightly reconstructed) **88** *Geotrochus scolops* sp. n. **88A** Frontal view, shell 10.0 mm high **88B** Umbilical view (Malaysia, Sabah, Tawau Province, Tawau Hills N.P., BOR/MOL 2432, holotype).

###### Habitat in Sabah and distribution.

Primary and old secondary forest on sandstone and volcanic bedrock, lowland conditions. Living on decaying wood. **Sabah, scattered localities: Labuan, Pulau Tiga; Pulau Gaya, Tawau Hills.** Also in Sarawak. Distribution: Sumatra. A record from Morotai, Moluccas (Van Benthem Jutting 1941: 23) is probably erroneous.

###### Cross diagnosis.

Generally characterized by the conical spire, but low-conical specimens occur. These approach the *Geotrochus labuanensis* group in general shape but have a more distinctly protruding apex and more distinctly concave sides. Also, relative to the shell height, the peripheral keel is positioned closer to the base of the shell, and the lower surface of the shell is often rather distinctly concave towards the periphery.

See also the remarks under *Geotrochus scolops*.

#### Group 1b. Suture between the whorls coinciding with the periphery. Shell 10–12.5 mm wide in adult specimens (check also *Geotrochus oedobasis*)

##### Geotrochus spilokeiria

Taxon classificationAnimaliaStylommatophoraTrochomorphidae

Vermeulen, Liew & Schilthuizen sp. n.

http://zoobank.org/648E599B-8DE4-45ED-9F64-DBFD0F938437

Figure 87

###### Holotype.

Malaysia, Sabah, West Coast Province, Crocker Range N.P., Ulu Kimanis, along Keningau-Kimanis road (BOR/MOL 2784).

###### Description.

Shell medium-sized, rather thin, opaque, above the periphery greyish with a somewhat vaguely outlined brown band just above the peripheral keel, and with an irregular pattern of white stains and spots which more or less follow the radial and spiral sculpture, periphery white, below the periphery dark brown, grading to white towards the periphery; shell conical with convex sides; apex not protruding. Surface shiny above the periphery, glossy below. *Whorls*: Apical whorls moderately convex, next whorls slightly convex, slightly shouldered, outer whorl almost flat; suture somewhat impressed in the apical whorls, not so in the outer whorls, coinciding with the periphery; last whorl acutely angular, slightly compressed at the periphery, slightly rounded below the periphery. *Protoconch* c. 1 5/8 whorls, smooth. *Teleoconch*. Radial sculpture: above the periphery rather distinct growth lines, raised at irregular intervals; below the periphery indistinct growth lines only. Spiral sculpture: last whorl with a sharp, pinched peripheral keel; above and close to this keel 1 thin, low, sharply outline spiral thread; above this 3–4 very low and wide, vaguely outlined ridges locally visible in tangential light; next to this radial and spiral sculpture oblique rows of very shallow, vaguely outlined indentations are locally visible; below the periphery locally with traces of a fine, shallow, vaguely outlined spiral striation. *Umbilicus* closed. *Peristome* not thickened, nor reflexed except on the columellar side (see remarks below). *Dimensions*: Height c. 8.5 mm; width c. 12.5 mm; h/w c. 0.68; diameters of the first 4 whorls c. 1.5 mm, c. 2.7 mm, c. 4.6 mm, c. 7.0 mm respectively; number of whorls c. 5 5/8, height aperture c. 3.5 mm; width aperture c. 7.0 mm.

###### Habitat in Sabah and distribution.

Disturbed primary forest on sandstone bedrock, c. 1400 m alt. **Sabah: Crocker Range only.** Endemic to Sabah.

###### Cross diagnosis.

Uniquely identified within *Geotrochus* by the brown, grey and white mottled shell. The convex sides of the shell, combined with the rapidly expanding whorls are also characteristic.

###### Remarks.

The only shell available is possibly sub-adult. The description of the aperture may need to be amended once more material becomes available.

The pattern of low, vaguely outlined spiral ridges and similar rows of oblique indentations do not occur in any other *Geotrochus* species.

###### Etymology.

The name refers to the dirty white apiral band [*spilos* (Gr.) = stained; *keiria* (Gr.) = bandage].

##### Geotrochus scolops

Taxon classificationAnimaliaStylommatophoraTrochomorphidae

Vermeulen, Liew & Schilthuizen sp. n.

http://zoobank.org/2EB67F35-1D06-4159-B31F-40B66BB0C6DC

Figure 88

###### Holotype.

Malaysia, Sabah, Tawau Province, Tawau Hills N.P. (leg. J.P. King, BOR/MOL 2432).

###### Description.

Shell medium-sized, rather thin, about opaque, brown, above the periphery with a very fine, slightly paler spiral striation, high-conical with concave sides; apex protruding. Surface slightly shiny above the periphery, glossy below. *Whorls*: Apical whorls moderately convex, other whorls slightly convex, slightly shouldered, last whorl almost flat; suture impressed in the inner whorls, not so in the outer, coinciding with the periphery; last whorl acutely angular, slightly compressed at the periphery, slightly rounded below the periphery. *Protoconch* c. 2 whorls, smooth. *Teleoconch*. Radial sculpture: above the periphery growth lines, slightly raised locally; below the periphery indistinct growth lines only. Spiral sculpture: last whorl with a sharp, somewhat pinched peripheral keel; start of fifth whorl with c. 3 thin, low, widely spaced spiral threads with an irregularly incised crest: 1 slightly more distinct close to the periphery, as well as 2 inconspicuous above these (penultimate whorl with c. 8 such threads, of which 3–4 are slightly more distinct); no spiral sculpture below the periphery. *Umbilicus* closed. *Peristome* slightly thickenened, but not reflexed except on the columellar side. *Dimensions*: Height c. 10.4 mm; width c. 12.5 mm; h/w 0.83–0.84; diameters of the first 4 whorls c. 1.1 mm, c. 1.7 mm, c. 2.2 mm, c. 3.0 mm respectively; number of whorls c. 8 3/8, height aperture c. 3 mm; width aperture c. 6.5 mm.

###### Habitat in Sabah and distribution.

Forest on sandstone or volcanic soil, lowland conditions. **Sabah: Tawau Hills National Park.** Endemic to Sabah.

###### Cross diagnosis.

Uniquely identified within *Geotrochus* by the high-conical shell with concave sides, and tapering towards the apex.

*Geotrochus conicoides* with a relatively high-conical shell are most similar, but still have a lower h/w-ratio: 0.57–0.66.

###### Etymology.

The name refers to the shape of the spire [*scolops* (Gr.) = pointed].

##### Geotrochus kitteli

Taxon classificationAnimaliaStylommatophoraTrochomorphidae

Vermeulen, Liew & Schilthuizen sp. n.

http://zoobank.org/AB073D02-62A6-4793-8420-BE7E44C87661

Figure 89

###### Holotype.

Malaysia, Sabah, West Coast Province, Kinabalu N.P., Headquarters area, Silau Silau (leg. K. Kittel, RMNH.5003930).

###### Examined material from Sabah.

*West Coast Province*, Kinabalu N.P., Sayap-Nunuhon trail at 1776 m (leg. T.S. Liew, Dominik, J. Lapidin & Jasilin, BOR/MOL 4109).

###### Description.

Shell rather small, rather thin, about opaque, yellowish brown, whorls slightly darker brown towards the periphery, moderately low-conical with approx. flat sides; apex not protruding. Surface dull or slightly shiny above the periphery, shiny below. *Whorls*: Apical whorls moderately convex, outer slightly convex, slightly shouldered; suture somewhat impressed, coinciding with the periphery; last whorl acutely angular, slightly compressed at the periphery, rounded below the periphery. *Protoconch* c. 2 1/8 whorls, smooth (see remark, below). *Teleoconch*. Radial sculpture: above the periphery rather distinct growth lines, raised at irregular intervals and wrinkling the shell surface, most conspicuously so below the suture, and giving the shell surface a somewhat rough appearance; below the periphery indistinct growth lines only. Spiral sculpture: last whorl with a sharp, somewhat pinched peripheral keel; start of fifth whorl with c. 15 thin, moderately spaced spiral threads: 1–2 slightly more distinct close to the periphery, as well as 13–14 inconspicuous above these; no spiral sculpture below the periphery. *Umbilicus* closed. *Peristome* thickened and reflexed. *Dimensions*: Height c. 5.5 mm; width c. 10 mm; h/w c. 0.55; diameters of the first 4 whorls c. 1.2 mm, c. 2.2 mm, c. 3.3 mm, c. 4.8 mm respectively; number of whorls c. 6 3/8, height aperture c. 3.5 mm; width aperture c. 5 mm.

**Figure 89–92.**
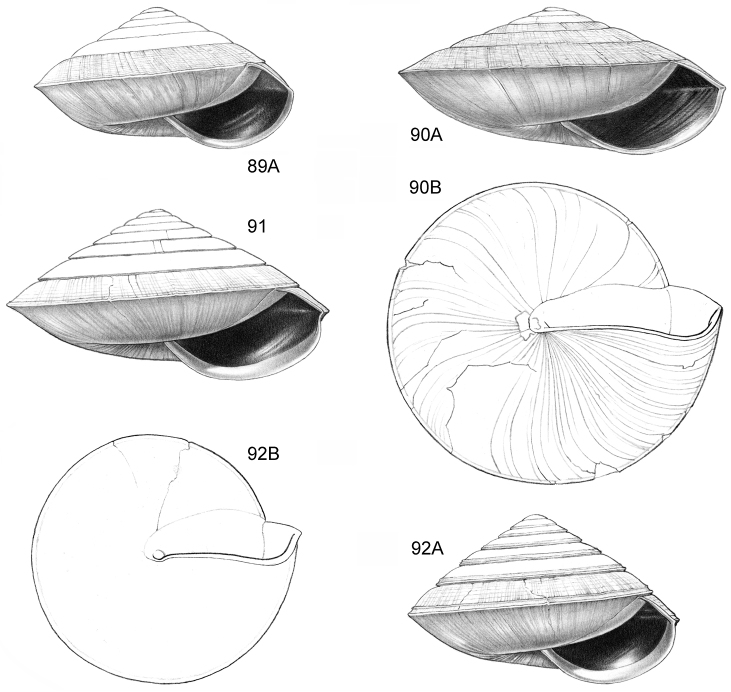
**89** *Geotrochus kitteli* sp. n. Frontal view, shell 5.5 mm high (Malaysia, Sabah, West Coast Province, Kinabalu N.P., Headquarters area, Silau Silau (RMNH.5003930, holotype) **90** *Geotrochus whiteheadi* (E.A. Smith, 1895) **90A** Frontal view, shell 5.5 mm high **90B** Umbilical view (Malaysia, Sabah, West Coast Province, Kinabalu N.P., Carson’s Falls, V 12710) **91** *Geotrochus subscalaris* sp. n. Frontal view, shell 7.0 mm high (Malaysia, Sabah, Sandakan Province, Kinabatangan valley, Batu Pangi, RMNH.5003931, holotype) **92** *Geotrochus meristotrochus* sp. n. **92A** Frontal view, shell 7.0 mm high **92B** Umbilical view (Malaysia, Sabah, Interior Province, Sepulut valley, Batu Punggul, RMNH.5003932, holotype).

###### Habitat in Sabah and distribution.

Mixed montane forest, 1500–1800 m alt. **Sabah: Mount Kinabalu only.** Endemic to Sabah.

###### Cross diagnosis.

In size and general shape most similar to *Geotrochus whiteheadi*, it differs by the higher ratio h/w, and by the more distinct growth lines above the periphery, which give the shell surface a rough appearance. It differs from *Trochomorpha rhysa* by its somewhat pinched peripheral keel and the not-nodular spiral sculpture above the periphery.

###### Remarks.

The single shell available is somewhat worn in the apical region. Traces of what may be radial riblets are present in the suture of the protoconch, close to the transition to the teleoconch.

###### Etymology.

Named after Mr. Klaus Kittel, Germany, who collected the type specimen.

##### Geotrochus whiteheadi

Taxon classificationAnimaliaStylommatophoraTrochomorphidae

(E.A. Smith, 1895)

Figure 90

Helix conicoides Metcalfe var. parva E.A. Smith, 1887: 132. *Trochonanina whiteheadi* E.A. Smith, 1895: 106. Type from Malaysia, Sabah.

###### Examined material from Sabah.

*West Coast Province.*Kinabalu N.P., Carson’s Falls (leg. J.J. Vermeulen, V 12710); Serinsim-Numbuyukon trail between 700 and 1680 m (leg. T.S. Liew, BOR/MOL 4024, BOR/MOL 4104); Monggis-Tambuyukon trail at 2080 m (leg. T.S. Liew, BOR/MOL 4110); Mesilau trail at 1948 m (leg. T.S. Liew & J. Lapidin, BOR/MOL 4393); Summit trail between 1700 and 1784 m (leg. T.S. Liew, BOR/MOL 4392; leg. T.S. Liew & Dominik, BOR/MOL 4396).

###### Description.

Shell rather small, rather thin, about opaque, pale corneous to pale brown, low-conical with approx. flat sides; apex not protruding. Surface with a silky luster above the periphery, shiny below. *Whorls*: Apical whorls moderately convex, outer slightly convex, slightly shouldered; suture impressed in the inner whorls, hardly so in the outer, coinciding with the periphery; last whorl acutely angular, (slightly) compressed at the periphery, moderately rounded below the periphery. *Protoconch* 2 1/8–2 1/4 whorls, smooth. *Teleoconch*. Radial sculpture: above the periphery growth lines, slightly raised locally; below the periphery indistinct growth lines only. Spiral sculpture: last whorl with a sharp, somewhat pinched peripheral keel; start of fifth whorl with c. 15 thin, moderately spaced spiral threads: 1–2 slightly more distinct close to the periphery, as well as 13–14 inconspicuous above these; no spiral sculpture below the periphery. *Umbilicus* closed. *Peristome* thickened and reflexed. *Dimensions*: Height 5.5–6.0 mm; width 12.3–12.5 mm; h/w 0.44–0.48; diameters of the first 4 whorls 1.2–1.4 mm, 2.1–2.4 mm, 3.4–3.8 mm, 5.2–5.8 mm respectively; number of whorls 6 1/4–6 3/8, height aperture 3.3–3.5 mm; width aperture c. 6.5 mm.

###### Habitat in Sabah and distribution.

Mixed montane forest, 700–2100 m alt. **Sabah: Mount Kinabalu only.** Endemic to Sabah.

#### Group 2. Suture slightly below the periphery in the last 4–5 whorls of adult specimens.

##### Geotrochus subscalaris

Taxon classificationAnimaliaStylommatophoraTrochomorphidae

Vermeulen, Liew & Schilthuizen sp. n.

http://zoobank.org/41FAD031-FC38-4CCF-9FDF-567E2EA07EF3

Figure 91

Geotrochus heraclea (not of E.A. Smith, 1895) Solem, 1964: 27.

###### Holotype.

Malaysia, Sabah, *Sandakan Province*, Kinabatangan valley, Batu Pangi (RMNH.5003931).

###### Examined material from Sabah.

Sandakan Province. Kinabatangan valley, Batu Mawas (leg. T.S. Liew & M. Schilthuizen, BOR/MOL 1956, BOR/MOL 2251, BOR/MOL 1991); Batu Materis (leg. M. Schilthuizen, BOR/MOL 2430, BOR/MOL 2426); Batu Keruak (leg. T.S. Liew & B. Elahan, BOR/MOL 1847); Batu Pangi (leg. J.J. Vermeulen & M. Schilthuizen, V 9634, BOR/MOL 2421); Batu Tai (not Bod Tai) near Gomantong (leg. J.J. Vermeulen & M. Schilthuizen, V 9597); Batu Tomanggong Besar (leg. M. Schilthuizen, BOR/MOL 2428, BOR/MOL 2429; leg. M. Schilthuizen, A. van Til & B. Elahan, BOR/MOL 2973; leg. T.S. Liew & B. Elahan, BOR/MOL 2284); Batu Tomanggong Kecil (leg. M. Salverda & H. van Oosten, BOR/MOL 2427); Batu Tulug (Batu Putih) along road Lahad Datu-Sandakan, North of bridge over Kinabatangan River (leg. J.J. Vermeulen & H. Duistermaat, V 1490); Gomantong Hill 30 km South of Sandakan (leg. J.J. Vermeulen & H. Duistermaat, V 1627; leg. M. Schilthuizen, BOR/MOL 894; leg. T.S. Liew & J.P. King, BOR/MOL 3668); Tandu Batu (leg. J.J. Vermeulen & M. Schilthuizen, V 9626); Unnamed hill near Sukau Police Station (leg. T.S. Liew & B. Elahan, BOR/MOL 2220). Segama valley, North end of limestone ridge on East bank Tabin River (leg. J.J. Vermeulen & M. Schilthuizen, V 7763). Tawai Mountains near Telupid (leg. J.J. Vermeulen, V 1259). *Tawau Province*. Batu Baturong c. 50 km W.S.W. of Lahad Datu (leg. J.J. Vermeulen & H. Duistermaat, V 1852). Danum Valley (leg. H.A. Rutjes, BOR/MOL 888, BOR/MOL 2936; leg. M. Schilthuizen, BOR/MOL 2934); Gua Madai c. 40 km S.S.W. of Lahad Datu (leg. J.J. Vermeulen & H. Duistermaat, V 1741). Segama valley, hill N.W. of crossing road Sandakan-Lahad Datu with the Segama River (leg. J.J. Vermeulen & H. Duistermaat, V 1657); ‘Kirk’s Cave’ 8 km North of Lahad Datu (leg. J.J. Vermeulen, V 1253); Sabahmas Cave (leg. J.J. Vermeulen, V 7469, BOR/MOL 897). Tabin Wildlife Reserve (leg. T. Kimsin & H.N. Chai, BOR/MOL 898). Semporna area, Segarong Hills, Batu Tengar, 25 km E.S.E. Of Kunak (leg. J.J. Vermeulen & H. Duistermaat, V 1816); Bukit Pababola, 25 km E.S.E. of Kunak (leg. J.J. Vermeulen & H. Duistermaat, V 1780).

###### Description.

Shell rather small, rather thin, about opaque, yellowish to pale brown, moderately low-conical with slightly concave to slightly convex sides; apex slightly to moderately protruding. Surface with a silky luster. *Whorls*: Apical whorls moderately convex, outer approx. flat to slightly convex; suture impressed, between the outer 4–5 whorls slightly below the periphery; last whorl acutely angular, compressed at the periphery, rounded below the periphery. *Protoconch* 2 1/8–2 1/4 whorls, smooth. *Teleoconch*. Radial sculpture: above the periphery some growth lines, locally grading into inconspicuous riblets; below the periphery indistinct growth lines only. Spiral sculpture: last whorl with a sharp, pinched peripheral keel; start of fifth whorl with 6–10 thin, widely spaced spiral threads: Usually 1–2 (slightly) more distinct close to the periphery, as well as 5–10 inconspicuous above these; no spiral sculpture below the periphery. *Umbilicus* closed. *Peristome* thickened and reflexed. *Dimensions*: Height 6.2–7.3 mm; width 11–13.0 mm, h/w 0.52–0.59; diameters of the first 4 whorls 1.1–1.4 mm, 1.9–2.4 mm, 2.7–3.6 mm, 4.0–4.8 mm respectively; number of whorls 6 1/4–8, height aperture 3.2–4.0 mm; width aperture 6.0–7.2 mm.

###### Habitat in Sabah and distribution.

Primary and secondary forest and more degraded vegetation types on limestone soil, up to 200 m alt. **Sabah: East coast.** Endemic to Sabah.

###### Cross diagnosis.

Most similar to *Geotrochus whiteheadi*, differs by the suture which is situated slightly below the periphery in the outer whorls.

###### Etymology.

The name refers to the slightly protruding peripheral keel, creating a small notch in between the whorls in lateral view [*sub-scalaris* (L) = a little resembling a ladder].

##### Geotrochus meristotrochus

Taxon classificationAnimaliaStylommatophoraTrochomorphidae

Vermeulen, Liew & Schilthuizen sp. n.

http://zoobank.org/A3969A3E-5C3D-4A25-BAE8-13E53AAD5FD8

Figure 92

###### Holotype.

Malaysia, Sabah, Interior Province, Sepulut valley, Batu Punggul (leg. J.J. Vermeulen, RMNH.5003932).

###### Examined material from Sabah.

*Interior Province*. Upper Padas valley, Long Pa Sia (leg. T.S. Liew & Meckson, BOR/MOL 4343). Crocker Range N.P., near the km 59 marker on the road Kota Kinabalu-Tambunan (leg. J.J. Vermeulen, V 1186); West of the km 10 marker on the road Tambunan-Ranau, Mahua Waterfall (leg. J.J. Vermeulen & M. Schilthuizen, V 9743; leg. J. Schilthuizen, BOR/MOL 896; leg. M. Schilthuizen, BOR/MOL 2420, BOR/MOL 2423, BOR/MOL 2424, BOR/MOL 3143, BOR/MOL 3345). Gunung Trusmadi slopes, Gua Loloposon (leg. J.J. Vermeulen, V 13236). Pinangah valley, Batu Urun (= Bukit Sinobang) (leg. J.J. Vermeulen, V 8008, V 1163, BOR/MOL 891); Pun Batu c. 30 km West of Sepulut (leg. J.J. Vermeulen, V 1302); Batu Punggul (leg. J.J. Vermeulen, V 1979; leg. T.S. Liew, M. Schilthuizen & S. Chiba, BOR/MOL 4317); Batu Pungiton (leg. M. Schilthuizen, BOR/MOL 890); Batu Temurung (leg. J.J. Vermeulen, V 8054, BOR/MOL 892; leg. M. Schilthuizen, BOR/MOL 893); Gua Sanaron (leg. J.J. Vermeulen & M. Schilthuizen, V 8073; leg. T.S. Liew, M. Schilthuizen & S. Chiba, BOR/MOL 4280; leg. M. Schilthuizen, BOR/MOL 887); Kampung Labang (leg. J.J. Vermeulen & M. Schilthuizen, V 7727, BOR/MOL 895). *Sandakan Province*. Beluran, Meliau Range (leg. T.S. Liew, M. Schilthuizen & A. van Til, BOR/MOL 3207, BOR/MOL 3208, BOR/MOL 3209, BOR/MOL 3210). *Tawau Province*. Batu Baturong, North slope (leg. J.J. Vermeulen, V 7603). Segama valley, limestone hill on North bank Segama River, near bridge of road Sandakan to Lahad Datu (leg. J.J. Vermeulen, V 7517). Tawau Hills Park (leg. J.P. King, BOR/MOL 2425; leg. T.S. Liew, J.J. Vermeulen & M. Schilthuizen, BOR/MOL 4272). *West Coast Province*. Kinabalu N.P., Serinsim-Numbuyukon trail between 548 and 1204 m (leg. T.S. Liew, BOR/MOL 4474, BOR/MOL 4475, BOR/MOL 4477). Kota Kinabalu, Kiansom (leg. M. Schilthuizen, BOR/MOL 889, BOR/MOL 2935; leg. UMS Tropical Malacology Course participants, BOR/MOL 3481).

###### Description.

Shell rather small, rather thin, about opaque, yellowish to pale brown, moderately low-conical with flat to slightly convex sides; apex slightly protruding or not. Surface with a silky luster. *Whorls*: Apical whorls moderately convex, outer slightly convex; suture impressed, between the outer 4–5 whorls slightly below the periphery; last whorl acutely angular, slightly compressed at the periphery, rounded below the periphery. *Protoconch* 2 1/8–2 1/2 whorls, smooth. *Teleoconch*. Radial sculpture: above the periphery some growth lines, locally grading into inconspicuous riblets; below the periphery indistinct growth lines only. Spiral sculpture: last whorl with a sharp, slightly pinched peripheral keel; start of fifth whorl with 6–9 thin, widely spaced spiral threads: 1–2 distinct close to the periphery, as well as 4–8 inconspicuous ones above these; no spiral sculpture below the periphery. *Umbilicus* closed. *Peristome* thickened and reflexed. *Dimensions*: Height 6.0–7.0 mm; width 9.3–10.8 mm; h/w 0.59–0.67; diameters of the first 4 whorls 1.0–1.4 mm, 1.8–2.2 mm, 2.7–3.4 mm, 4.0–5.0 mm respectively; number of whorls 6 3/4–7 3/8, height aperture 2.8–3.5 mm; width aperture 4.8–5.5 mm.

###### Habitat in Sabah and distribution.

Primary, secondary forest and abandoned agricultural land on limestone and sandstone soil, up to 1300 m alt. **Sabah: West part, East coast (lower Segama River valley and further South).** Endemic to Sabah.

###### Cross diagnosis.

Shell less wide than *Geotrochus subscalaris*, compare shell width and aperture width of both species. Usually, the h/w-ratio is also higher than in *Geotrochus subscalaris*, but here a slight overlap exists.

###### Etymology.

The name refers to the slightly protruding peripheral keel, creating a small notch in between the whorls in lateral view [*meristos* (Gr.) = divided; *trokhos* (Gr.) = wheel, often used for the gastropod spire].
